# Establishment of a CRISPR/Cas9-Based Genome Editing System in *Macrobrachium rosenbergii*

**DOI:** 10.3390/ani16010013

**Published:** 2025-12-19

**Authors:** Junjun Yan, Siyu Qian, Guo Li, Yujie Liu, Liqian Zhou, Tiantian Ye, Cui Liu, Jilun Meng, Yukun Jie, Zhimin Gu

**Affiliations:** 1 Xianghu Laboratory, Hangzhou 311231, China; yanjunjun@xhlab.ac.cn (J.Y.);; 2 College of Biological and Environmental Sciences, Zhejiang Wanli University, Ningbo 315100, China; 3 School of Marine Biology and Fisheries, Hainan University, 58 People Road, Haikou 570228, China

**Keywords:** *Macrobrachium rosenbergii*, CRISPR/Cas9, genome editing, breeding

## Abstract

The giant freshwater prawn *Macrobrachium rosenbergii* is a globally farmed decapod species of high economic and nutritional value, but its genetic improvement has been constrained by the lack of an efficient genome editing workflow. Here, we optimized a CRISPR/Cas9 microinjection system for targeted mutagenesis in *M. rosenbergii* embryos. A controlled mating-based approach enabled routine collection of one-cell embryos, and developmental staging identified 0.5–2 h post-fertilization as the optimal injection window. Optimization of the needle tip (~1 μm) minimized mechanical damage, lowering early embryo mortality to ~10%. Using this system, the eye-development gene *MrPAX6* and the sex-related gene *MrIAG* were successfully edited. *MrPAX6* editing produced a 46.9% mutation rate and an average 50% reduction in eye pigment area. *MrIAG* editing achieved an 84% mutation rate, while post-injection survival to hatching was approximately 35%. These results establish an efficient and reproducible CRISPR/Cas9 editing system in *M. rosenbergii*, providing a foundation for functional genomics and genetic improvement in this commercially important prawn species.

## Simple Summary

The giant freshwater prawn *Macrobrachium rosenbergii* is an important farmed species, but progress in breeding better strains has been limited because gene function is hard to study in this animal. In this work, we developed a practical method to edit genes by injecting a gene-editing tool into very early embryos. We identified how to collect fertilized eggs, the best time to inject them, and the needle design that keeps most embryos alive. Using this method, we successfully disrupted a gene needed for eye development and another that controls male traits. This system provides a foundation for understanding key genes and may eventually support the creation of improved prawn lines for aquaculture.

## 1. Introduction

The giant freshwater prawn (*Macrobrachium rosenbergii*), belonging to the order Decapoda and family Palaemonidae, is one of the most economically important crustacean species in global aquaculture [1]. Native to the Indo-West Pacific region, *M. rosenbergii* is widely cultured across Asia, Africa, and South America due to its rapid growth, high nutritional value, and strong market demand [2,3]. In 2023, global production of *M. rosenbergii* reached 313,756 tons, with China contributing over half of the total output [4]. The species gained prominence as an alternative to marine shrimp in the mid-nineties, particularly after outbreaks of white spot syndrome virus (WSSV) severely impacted marine shrimp farming in the South and Southeast Asian countries [5]. However, despite extensive expansion of aquaculture practices and efforts in selective breeding and monosex culture, production yields of *M. rosenbergii* per area have shown no marked increase over the past decade [6,7,8]. Factors such as inbreeding, growth heterogeneity, and disease outbreaks have collectively constrained further productivity improvement [9,10,11]. Monosex culture, particularly the production of all-male populations, is increasingly prioritized in *M. rosenbergii* aquaculture owing to the faster growth, larger size, and higher commercial value of males [12]. In addition, targeted manipulation of genes associated with growth, disease resistance, and biosafety represents a promising direction for accelerating strain improvement [13]. Since traditional breeding in *M. rosenbergii* is limited by long generation times and complex traits [2], more advanced genetic improvement strategies are needed to enhance the productivity and sustainability of its aquaculture.

Originally discovered as an adaptive immune system in bacteria and archaea, the clustered regularly interspaced short palindromic repeat (CRISPR)/CRISPR-associated protein 9 (Cas9) mechanism enables sequence-specific recognition and cleavage of foreign DNA guided by small RNA molecules [14,15]. By harnessing this naturally occurring defense mechanism, researchers have developed the CRISPR/Cas9 system into a powerful and versatile genome-editing platform capable of precise genetic modification across diverse organisms [16,17]. In recent years, the CRISPR/Cas9 system has also found increasing applications in aquatic animals [18,19]. Studies in fish have demonstrated efficient CRISPR/Cas9 editing in species such as zebrafish [20,21], Nile tilapia [22], crucian carp [23], amphitriploid gibel carp [24], and blunt snout bream [25], enabling functional exploration of genes related to growth, metabolism, and intermuscular bone formation. Unlike fish, crustacean embryos pose unique technical barriers to genome editing, including rapid chorion hardening, yolk-rich cytoplasm, and the lack of artificial fertilization, rendering CRISPR/Cas9 delivery far more difficult than in fish. In recent years, early applications in crustaceans, such as *Daphnia magna* [26,27], *Exopalaemon carinicauda* [28,29], *Neocaridina denticulata sinensis* [30], *Neocaridina heteropoda* [31], *Eriocheir sinensis* [31], *Macrobrachium nipponense* [32], and *Penaeus monodon* [33], have shown that CRISPR/Cas9 can also induce gene mutations and facilitate functional genomics in these non-model aquatic invertebrates.

A recent study reported the successful use of CRISPR/Cas9-mediated gene editing in *M. rosenbergii* embryos through microinjection [34], demonstrating the feasibility of genome modification in this species. However, there is still a lack of a standardized and high-efficiency protocol for *M. rosenbergii* embryo microinjection and genome editing. The aim of this study was to establish and validate a practical and reproducible CRISPR/Cas9-based genome editing workflow for *M. rosenbergii*, through systematic optimization of embryo collection, developmental staging, and microinjection parameters. To demonstrate the feasibility of this workflow, we selected two biologically important *M. rosenbergii* genes, Paired box protein 6 (*MrPAX6*), involved in eye development [35,36], and insulin-like androgenic gland hormone (*MrIAG*), a key regulator of male differentiation [12,37], as targets for functional disruption. This study reports the first CRISPR/Cas9-mediated *MrIAG* editing in *M. rosenbergii*, provides a platform for *M. rosenbergii* gene function analysis, and paves the way for future applications in genetic improvement and breeding of this economically important species.

## 2. Materials and Methods

### 2.1. M. rosenbergii Rearing, Mating and Embryo Collection

Adult *M. rosenbergii* broodstock were obtained from a hatchery located in Huzhou, China (Zhejiang Nanyu Agricultural Technology Development Co., Ltd.) and maintained in indoor aerated tanks at 28 ± 1 °C under a 14 h light/10 h dark photoperiod. To obtain freshly fertilized embryos, newly molted, gonadally mature but not yet spawned females were selected each morning and paired with vigorous males (males with intact pereopods, active swimming behavior, and a hard exoskeleton) at a female-to-male ratio of 2:1 in small mating tanks (28 °C, continuous aeration). After ~6 h of mating, fertilized eggs were collected from the pleopods of the females between 14:00 and 18:00. Because females brood eggs as adhesive clumps, to dissociate egg clumps into single embryos, a mild trypsin treatment was applied. Trypsin is commonly used to disrupt protein-based adhesion through cleavage at lysine and arginine residues [38], and prawn egg clumps are known to be bound by a glycoprotein-rich adhesive matrix [39]. Low-concentration trypsin likely facilitates dissociation by partially digesting this matrix. This procedure did not affect embryo viability or development in our experiments (resulted in >99% intact embryos). Briefly, collected egg masses were treated with 50 mL trypsin (1 mg/mL, sterile freshwater) for 5 min at room temperature to dissociate them into single embryos. Trypsin activity was stopped by adding an equal volume of medium containing 1% fetal bovine serum (FBS), followed by two washes with fresh medium. Following trypsin dissociation, embryos were examined under a stereomicroscope. Only healthy, uniformly shaped embryos without signs of mechanical damage were selected and transferred into 10 mL Petri dishes, and maintained at 28 °C before microinjection.

### 2.2. Embryonic Developmental Staging

*M. rosenbergii* embryos were placed in 10 mL Petri dishes containing sterilized fresh seawater (salinity 13‰, supplemented with 100 U/mL penicillin and 100 μg/mL streptomycin), and incubated at 28 °C on a horizontal shaker set at 20 rpm to ensure gentle circulation and oxygenation. Embryos were observed daily under a stereomicroscope at 116× magnification (MZX81, Mshot, Guangzhou, China), and images were captured using a digital CCD camera (MSX11, Mshot, China; resolution 5280 × 3956). Embryonic stages were categorized into fertilized egg, early nuclear division, morula, blastula, gastrula, nauplius, zoea, and hatching larva based on morphological characteristics such as nuclear division, blastomere formation, gastrulation, eye pigmentation, and appendage development.

### 2.3. Design and Preparation of CRISPR/Cas9 Components

The coding sequences of *MrPAX6* (GenBank accession number: OP292287.1) and *MrIAG* (GenBank accession number: FJ409645.1) were retrieved from NCBI and aligned to the latest *M. rosenbergii* genome assembly (GCA_040412425.1) to determine genomic structures. Single guide RNAs (sgRNAs) were designed to target *MrPAX6* or *MrIAG* using CRISPOR (http://crispor.tefor.net/, accessed on 17 May 2024), minimizing potential off-target sites (listed in Table 1). The full-length sgRNAs were synthesized by GenScript (Nanjing, China) with three consecutive 2′-O-methyl-3′-phosphorothioate modifications at both the 5′ and 3′ termini to enhance resistance to nuclease degradation and increase stability, and Cas9 protein was purchased from New England Biolabs (NEB, M0646T, Ipswich, MA, USA). Both sgRNAs and Cas9 protein were stored at −80 °C until use. Prior to injection, Cas9 protein and sgRNA were mixed at a molar ratio of 1:1.4 (Cas9 protein, 224 ng/μL; sgRNA, 76 ng/μL) and incubated at room temperature for 5 min to allow ribonucleoprotein (RNP) complex formation. The freshly prepared RNP mixture was then immediately used for embryo microinjection.

### 2.4. Embryo Microinjection and Incubation

Before injection, separated embryos at or before the one-cell stage were arranged and immobilized in grooves preformed in a 3% agarose plate to facilitate stable positioning during microinjection. Microinjection was performed using a Digital Air Pressure Microinjector (DMP-400, Micrology Precision Instruments, Wuhan, China). Glass capillaries (outer diameter 1.0 mm, inner diameter 0.5 mm) were used to prepare injection needles. Needles were pulled using a programmable horizontal micropipette puller (HL-1000, Micrology Precision Instruments, Wuhan, China) and subsequently polished with a microgrinder (PG-22C, Micrology Precision Instruments, Wuhan, China) to obtain tip openings of approximately 1 μm, 2 μm, or 3 μm. Each needle was backfilled with the Cas9-sgRNA RNP mixture immediately before injection. Since early *M. rosenbergii* embryos at the one-cell stage appear uniformly yellow and are fully yolk-filled, without a morphologically distinguishable animal or vegetal pole. During microinjection, the needle was inserted directly into the yolk mass, and the injection was performed as close as possible to the presumptive nuclear region. For needle-tip optimization tests, each tip-size group (1 μm, 2 μm, 3 μm) included three biological replicates, each containing approximately 180 embryos. For *MrPAX6* editing, 352 embryos were injected and divided into three culture dishes. For *MrIAG* editing, embryos were distributed into three dishes, with ~200 embryos per dish, and each dish was treated as an independent replicate. For each injection experiment, an equal number of uninjected embryos collected at the same developmental stage and from the same batch of broodstock were maintained in parallel as wild-type controls. These embryos were cultured identically and used for survival rate comparisons and phenotypic assessment. Following injection (~500 pL per embryo), both injected and uninjected control embryos were transferred into 10 cm Petri dishes containing sterilized fresh seawater (salinity 13‰, supplemented with 100 U/mL penicillin and 100 μg/mL streptomycin) and incubated at 28 °C with gentle aeration on a horizontal shaker. The culture water was changed once a day. Survival rates were calculated as the percentage of embryos that remained viable relative to the total number of embryos injected.

### 2.5. DNA Extraction, PCR Amplification, and Sequencing Analysis

At 8 days post-fertilization (dpf), 30 surviving injected embryos (10 per dish) and uninjected wild-type embryos were randomly selected for genomic DNA extraction using the TIANamp Marine Animal DNA Kit (Tiangen, Beijing, China). The *MrPAX6* and *MrIAG* target regions were amplified using gene-specific primers listed in Table 1, generating expected product size of 729 bp and 434 bp, respectively. PCR amplification was performed using PrimeSTAR Max DNA Polymerase (Takara, Kyoto, Japan) in a 25 μL reaction containing 12.5 μL 2× Premix, 0.4 μM forward primer, 0.4 μM reverse primer, and ~50 ng genomic DNA. The thermal cycling program was: 98 °C for 2 min (initial denaturation); 35 cycles of: 98 °C for 10 s (denaturation), 55 °C for 15 s (annealing), 72 °C for 30 s (extension); 72 °C for 5 min (final extension). PCR products were purified and Sanger sequenced by Tsingke Biotechnology Co., Ltd. (Beijing, China) using an ABI 3730XL DNA Analyzer with standard BigDye Terminator v3.1 chemistry. Mutation efficiency was calculated based on sequence alignment using the TIDE online tool (http://shinyapps.datacurators.nl/tide/, accessed on 19 June 2024). Briefly, mutation efficiency was calculated as the sum of all indel frequencies above background noise relative to the wild-type reference trace. Default settings were used, including decomposition window size and indel detection up to ±20 bp.

### 2.6. Phenotypic Observation and Data Analysis

For *MrPAX6*-edited embryos, eye pigmentation was assessed at 8 dpf using ImageJ software (version: 1.53k) to quantify eye spot area. Briefly, fifty wild-type and fifty injected embryos were randomly selected from the surviving population at 8 dpf, and were further photographed under identical microscope settings. A scale bar from each image was first calibrated in the ImageJ software, allowing pixel measurements to be automatically converted to micrometers. For each embryo, only one eye was measured, and the pigmented eye region was manually selected to calculate its area (reported in μm^2^). Statistical significance was performed using a two-sample Student’s *t*-test under the assumption of equal variances [40], with *p*-value < 0.05 considered significant. For *MrIAG*-edited embryos, mortality and developmental progress were recorded daily until hatching, and editing efficiency was determined by sequencing analysis as described above.

## 3. Results

### 3.1. A Controllable and Convenient Strategy for Collecting One-Cell Stage Fertilized Eggs in M. rosenbergii

**Figure 1:**
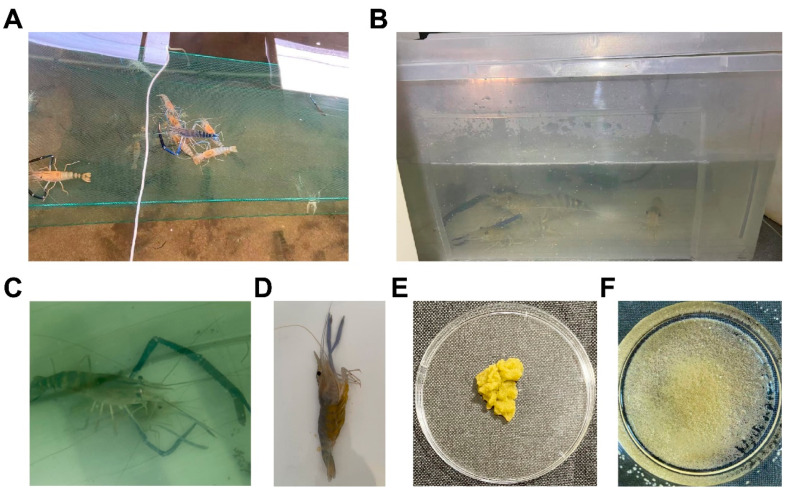
Controlled workflow for collecting one-cell-stage embryos of *M. rosenbergii*. (**A**) Several freshly molted, gonad-ripe females congregated on nylon nets in the broodstock pond, attracting males for mating. (**B**) Two selected females and one vigorous male were placed in a small indoor aerated tank with controlled temperature (28 °C) to facilitate mating. (**C**) A male and female *M. rosenbergii* engaged in copulation. (**D**) A female immediately after spawning, with some fertilized eggs still visible near the oviduct opening located at the base of the third pair of pereiopods, indicating that spawning has just occurred. (**E**) The fertilized egg mass was carefully removed from the female pleopods. (**F**) Dispersed single fertilized embryos were obtained after enzymatic treatment with 1 mg/mL trypsin for 5 min at room temperature.

We found that females molt before mating and move from the pond bottom onto nylon nets to avoid conspecific attacks (Figure 1A). Based on this behavior, newly molted, gonadally mature but not yet spawned females were selected each morning and paired with vigorous males at a female-to-male ratio of 2:1 in small indoor tanks (28 °C, aerated) (Figure 1B). After approximately 6 h, females typically have mated and begin carrying eggs (Figure 1C,D), allowing us to collect one-cell-stage embryos between 14:00 and 18:00 on the same day.

On average, each *M. rosenbergii* female carries about 50,000 eggs, while approximately 5000 are sufficient for microinjection. To minimize interference with normal reproduction, roughly 8–10% of the egg mass was carefully removed for experimental use (Figure 1D,E), after which the broodstock were returned to the pond, ensuring that the hatchery’s breeding operations remained unaffected. Trypsin digestion was then employed to dissociate the egg mass into single embryos (Figure 1F).

### 3.2. Developmental Staging and Optimal Microinjection Collection Window in M. rosenbergii Embryos

**Figure 2:**
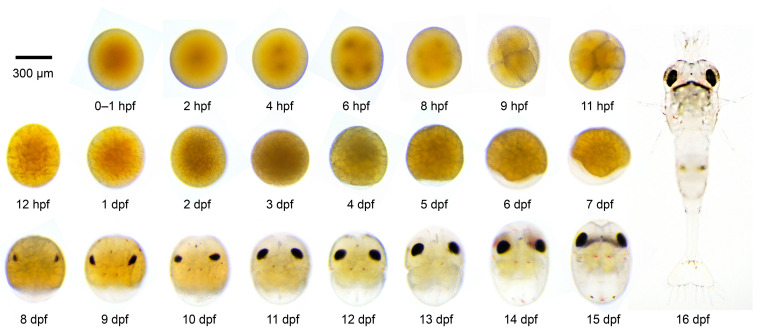
Embryonic development stages of *M. rosenbergii*. 0–1 h post-fertilization (hpf), newly fertilized egg. 2 hpf, visible nucleus formation. 4 hpf, appearance of two nuclei. 6 hpf, four nuclei present. 8 hpf, four blastomeres formed. 9–12 hpf, morula stage. 1–2 days post-fertilization (dpf), blastula stage. 3 dpf, gastrula stage. 4–7 dpf, nauplius stage. 8–15 dpf, zoea stage. 16 dpf, hatching larvae. Scale bars are 300 μm.

Newly fertilized eggs were slightly oval to spherical, measuring 500–580 μm in diameter, with no visible nucleus (Figure 2). At ~2 h post-fertilization (hpf), a single nucleus appeared and underwent successive divisions without cleavage furrows, defining the early nuclear division stage (Figure 2). Clear blastomeres formed at ~8 hpf (4-cell) and continued dividing into 8 (~9 hpf), 16 (~11 hpf), and 32 (~12 hpf) cells, corresponding to the morula stage (Figure 2). From 1–2 dpf, embryos entered the blastula stage, followed by gastrulation at 3 dpf, when the cell mass condensed toward one pole and a transparent region appeared and progressively enlarged (Figure 2). By 4 dpf, embryos entered the nauplius stage, followed by progressive development until 8 dpf, when eye pigmentation initiated and distinct eye spots became visible, marking the transition to the zoea stage (Figure 2). During this period, appendages gradually formed, the heartbeat became observable, yolk reserves were progressively consumed, and larval morphology gradually became apparent. By 16 dpf, over 95% of larvae hatched (Figure 2).

We found that newly fertilized *M. rosenbergii* eggs were too fragile for microinjection, nearly all embryos ruptured upon needle insertion, and survival was close to 0%, whereas the chorion began to harden sufficiently for needle penetration at ~0.5 hpf. However, after approximately 2 hpf, the chorion begins to undergo noticeable hardening, and the optimal microinjection needle could barely penetrate the embryo membrane, resulting in near-zero successful injections. Thus, the optimal injection window for *M. rosenbergii* embryos was determined to be 0.5–2 hpf.

### 3.3. Optimization of Microinjection Needle for M. rosenbergii Embryos

During repeated injections, we observed that the geometry of the microinjection needle, particularly the diameter of the needle tip opening, had a critical influence on post-injection embryo survival. *M. rosenbergii* fertilized eggs are heavily yolk-loaded, possess relatively high internal osmotic pressure, and undergo progressive chorion hardening after fertilization. As a result, needles with a tip that is too wide cause embryo rupture, whereas excessively fine or long tips fail to penetrate the chorion efficiently, resulting in low injection success.

**Figure 3:**
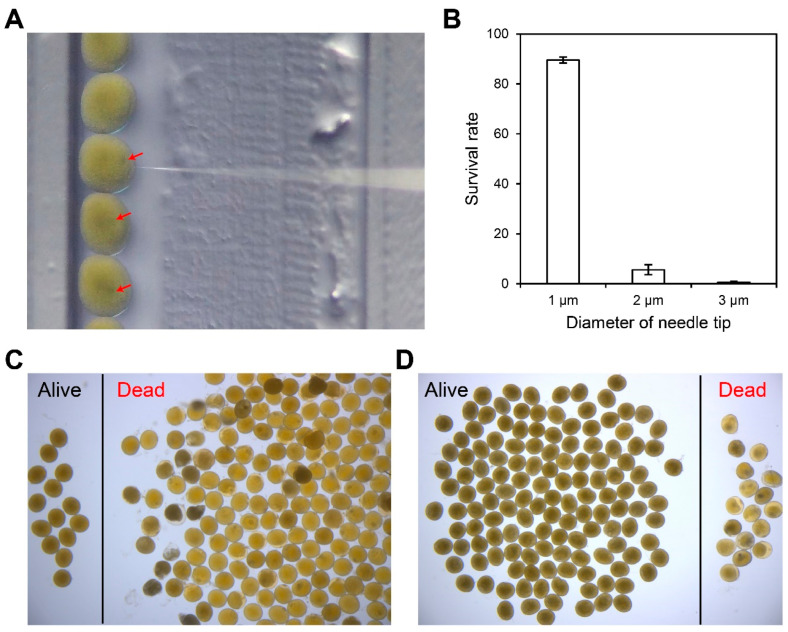
Optimization of microinjection needle tip diameter for *M. rosenbergii* embryos. (**A**) Schematic view of microinjection using a needle with a 1 μm tip opening. The red arrow indicates the injected solution diffusing within the embryo. (**B**) Embryo survival rates at 24 h post-injection using needles with different tip openings (1 μm, 2 μm, and 3 μm). Each treatment included three replicates with approximately 180 embryos per group. (**C**) Injection outcome using a 2 μm needle tip. (**D**) Injection outcome using a 1 μm needle tip.

To systematically evaluate the effect of needle tip opening size, embryos were injected using glass needles with tip diameters of approximately 1 μm, 2 μm, or 3 μm. Needles with a 1 μm tip penetrated the chorion smoothly, caused no visible structural damage, and allowed the injected solution to disperse rapidly within the embryo (Figure 3A). In contrast, 2 μm and 3 μm tips frequently resulted in yolk leakage upon insertion, and more than 90% of embryos died (94.4 ± 2.0% for the 2-μm-tip group and 99.5 ± 0.5% for the 3-μm-tip group) within 24 h post-injection (Figure 3B,C). When using 1 μm tips, embryo mortality was reduced to ~10% at 24 hpf, representing the highest survival rate among all tested conditions (Figure 3B,D). Based on these results, a needle tip diameter of approximately 1 μm was selected as the optimal specification for microinjection into *M. rosenbergii* fertilized eggs.

### 3.4. CRISPR/Cas9-Mediated Targeted Mutagenesis of MrPAX6 in M. rosenbergii

**Figure 4:**
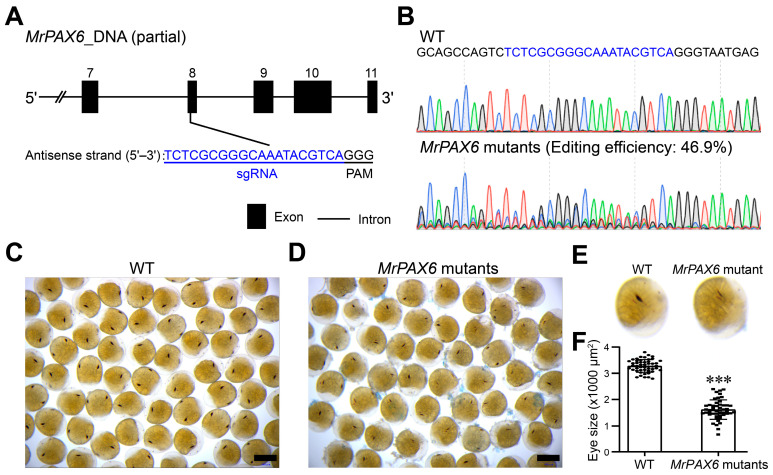
CRISPR/Cas9-mediated targeted mutagenesis of *MrPAX6* and resulting eye pigmentation phenotypes in *M. rosenbergii* embryos. (**A**) Partial DNA sequence of *MrPAX6* showing the sgRNA target site located on the antisense strand (highlighted in blue). (**B**) Representative Sanger sequencing chromatograms of wild-type (WT) and *MrPAX6*-edited embryos around the sgRNA target region (highlighted in blue), showing overlapping peaks in mutants. (**C**) Wild-type embryos at 8 dpf. Scale bars are 400 μm. (**D**) *MrPAX6*-mutant embryos at 8 dpf exhibiting reduced eye pigmentation. Scale bars are 400 μm. (**E**) Enlarged pictures of representative embryos from (**C**,**D**), showing clearly visible differences in pigment spot size. (**F**) Quantitative comparison of eye spot area between wild-type and *MrPAX6*-edited embryos (*n* = 50 per group). Error bars represent SD; *** *p*-value < 0.001.

To establish a proof-of-concept genome editing system in *M. rosenbergii*, we selected *MrPAX6* as a visible phenotypic marker gene for CRISPR/Cas9 mutagenesis. Genomic analysis revealed that *MrPAX6* spans 181,053 bp in the *M. rosenbergii* genome and is composed of 11 exons and 10 introns (sequence provided in Supplementary Materials). The coding sequence of *MrPAX6* encodes a 581-aa protein, and the CRISPR/Cas9 sgRNA was designed to target the antisense strand within exon 8 (Figure 4A).

A total of 352 embryos were injected across three dishes. Nearly 50% of embryos died within the first four days post-injection, after which survival stabilized. By 8 dpf, when eye pigmentation became clearly visible, 162 embryos remained alive. Genomic DNA extracted from 30 randomly selected embryos (10 per dish) revealed a *MrPAX6* editing efficiency of 46.9% (Figure 4B). The mean eye spot area of wild-type embryos was 3281.97 μm^2^, whereas *MrPAX6*-edited embryos showed a significantly reduced mean area of 1620.11 μm^2^, corresponding to ~50% of the wild-type size (Figure 4C–F). Thus, *MrPAX6* editing has resulted in markedly smaller eye spots relative to wild-type embryos.

### 3.5. CRISPR/Cas9 Targeted Editing of the Sex-Related Gene MrIAG in M. rosenbergii

**Figure 5:**
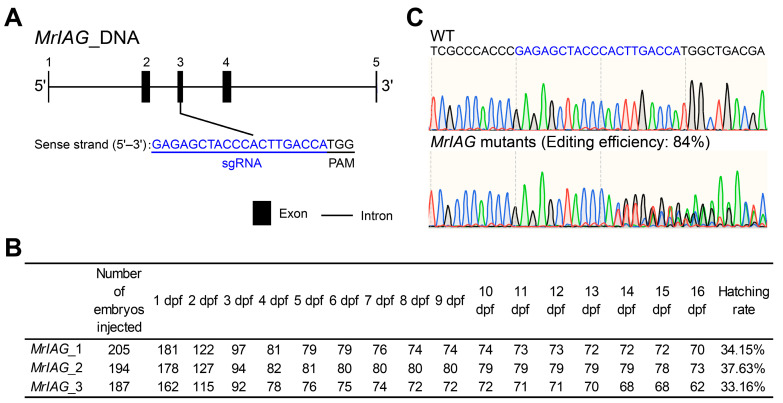
CRISPR/Cas9-mediated targeted editing of *MrIAG* in *M. rosenbergii* embryos. (**A**) Partial DNA sequence of *MrIAG* showing the sgRNA target site located on the sense strand (highlighted in blue). (**B**) Daily survival rate of embryos following CRISPR/Cas9 injection targeting *MrIAG*. Data are shown from three independent injection groups (~200 embryos per group). (**C**) Representative Sanger sequencing chromatograms of wild-type and *MrIAG*-edited embryos around the sgRNA target region (highlighted in blue), showing overlapping peaks in edited groups.

The genomic structure of *MrIAG* in *M. rosenbergii* was resolved as a 6704 bp locus comprising 5 exons and 4 introns (sequence provided in Supplementary Materials). The coding sequence of *MrIAG* encodes a 173-aa protein, and the sgRNA was designed to target exon 3 (Figure 5A). Embryos were injected across three dishes, with ~200 embryos per dish. Daily monitoring showed that approximately half of the injected embryos died during the first 4 days post-injection, after which survival stabilized (Figure 5B). By day 8 post-fertilization, 30 embryos (10 per dish) were randomly selected for genomic DNA extraction and sequencing, which revealed an *MrIAG* editing efficiency of 84% (Figure 5C).

## 4. Discussion

*PAX6* is a highly conserved transcription factor that plays a central role in eye development across metazoans. Loss-of-function mutations in *PAX6* result in severe eye malformations in vertebrates, insects, and other invertebrates [41,42]. It should be noted that *MrPAX6* editing in *M. rosenbergii* embryos resulted in a spectrum of eye pigmentation phenotypes, where some embryos exhibited eye spots reduced to ~20% of the wild-type size, while others retained approximately two-thirds (Figure 4F). This variation suggests differences in editing extent among individual embryos, likely due to mosaicism or variable DNA repair outcomes. Nevertheless, the average phenotype (~50% eye pigment reduction) was generally in agreement with the 46.9% mutation frequency detected by sequencing, supporting the overall accuracy of the editing and phenotyping strategy.

A pronounced increase in embryo mortality occurred during 2–3 dpf, corresponding to the blastula-to-gastrula transition when the cell mass begins to invaginate and a transparent region expands. At this stage, the embryo surface undergoes localized structural weakening, making it particularly sensitive to prior injection-induced damage. If the microinjection site coincides with the invagination zone, the local damage to the chorion may lead to an imbalance in internal and external pressure, resulting in embryo death. After ~5 dpf, when nearly all embryos had entered the nauplius stage and became morphologically stable, mortality dropped sharply. This developmental sensitivity also aligns with our finding that a ~1 μm needle tip for microinjection greatly improved survival by minimizing mechanical injury during early embryogenesis. Overall, the embryonic development of *M. rosenbergii* closely resembles that reported for other *Macrobrachium* species [32,43], suggesting that our observations may serve as a practical reference for optimizing embryo microinjection and genome-editing procedures in other *Macrobrachium* prawns.

In addition to microinjection, several emerging CRISPR/Cas9 delivery strategies have been explored in crustaceans. Nanocarrier-based approaches, such as PEI-coated single-walled carbon nanotubes in *Litopenaeus vannamei* [44], and cell-penetrating peptide-modified nanocarriers in *Penaeus monodon* [33], have demonstrated the feasibility of batch and noninvasive delivery of gene-editing components. Recent work has also shown that crustacean exosomes efficiently transport nucleic acids, suggesting their potential as natural vectors for RNA or RNP delivery [45]. Although promising, these systems remain under optimization, and their delivery efficiency, embryo permeability, and species-specific compatibility still require systematic evaluation in *M. rosenbergii*. Microinjection was therefore chosen in this study because it currently provides the most precise and controllable delivery into one-cell embryos, enabling accurate timing and direct visualization of successful injection. Nonetheless, integrating nanocarrier- or exosome-based systems in the future may allow scalable, high-throughput genome editing and expand the applicability of CRISPR/Cas9 technologies beyond early embryos in *M. rosenbergii*.

Although the pooled sequencing results indicate a relatively high overall editing efficiency, they do not capture variation among individual embryos, which may exhibit different mutation rates and degrees of mosaicism. In addition, potential off-target effects were also not assessed in this study, future work should include off-target validation using deep sequencing to assess genome-wide editing specificity in *M. rosenbergii*. In addition, the post-injection survival rate remained low (approximately 30%), highlighting the methodological limitations of microinjection. Less invasive delivery platforms, such as nanocarrier-based systems, may help reduce embryo damage and improve survival in future studies.

Previous RNAi studies demonstrated that silencing *MrIAG* in male *M. rosenbergii* can induce partial or complete sex reversal and generate neo-females, confirming that *MrIAG* functions as a key regulator in the male sex-determining pathway [37,46,47]. However, RNAi is transient and non-heritable, making it unsuitable for stable breeding applications. By contrast, CRISPR/Cas9 knockout enables permanent and inheritable *IAG* disruption, providing a genetic basis for monosex seed production. This concept has already been validated in the ridgetail white prawn *Exopalaemon carinicauda*, where CRISPR/Cas9-mediated *IAG* knockout caused full male-to-female sex reversal and the mutation was transmitted to the next generation, allowing monosex progeny to be produced through controlled crosses [28]. Because males grow faster and reach larger sizes than females, an all-male culture is economically advantageous in *M. rosenbergii* farming. Stable *MrIAG* knockout in *M. rosenbergii* therefore offers a practical route to generate sex-reversed broodstock without repeated RNAi treatments or androgenic gland removal and provides a genetic platform for both commercial monosex production and fundamental studies of sex determination. It is important to note that the present study demonstrates gene editing only at the embryonic level. Future studies involving long-term rearing of edited individuals, reproductive assessments, and genetic transmission analyses will be essential to evaluate the feasibility of establishing stable *MrIAG*-null broodstock for breeding applications in *M. rosenbergii*.

## 5. Conclusions

Genome editing represents a promising approach for functional genomics and trait-oriented breeding in *M. rosenbergii*. The findings in this study highlight the potential of genome editing to advance sex-control strategies and accelerate genetic improvement in this prawn species. Future work will focus on establishing heritable gene-edited lines, verifying long-term functional consequences of *MrIAG* knockout, and further improving editing efficiency through advanced tools and less invasive delivery platforms.

## Figures and Tables

**Table 1:** Primer/gRNA used in this study.

Sequence Name	Sequence (5′-3′)
*MrPAX6* gRNA	TCTCGCGGGCAAATACGTCA
*MrPAX6* forward primer	TGGGTCTTACCCTTGATCCT
*MrPAX6* reverse primer	AGTAACAATGAACGTGACGC
*MrIAG* gRNA	GAGAGCTACCCACTTGACCA
*MrIAG* forward primer	CATACTATCACGTTAGCGCG
*MrIAG* reverse primer	CCTAATTGGGTGACAACTGCGA

## Data Availability

The original contributions presented in this study are included in the article/Supplementary Materials. Further inquiries can be directed to the corresponding author.

## Supplementary Materials

The following supporting information can be downloaded at: https://www.mdpi.com/article/10.3390/ani16010013/s1, File S1: The cDNA and DNA sequence of *MrPAX6* and *MrIAG*.
