# Supplementary material for: Establishment of a CRISPR/Cas9-Based Genome Editing System in Macrobrachium rosenbergii
Source: Animals (Basel). 2025 Dec 19;16(1):13. doi: 10.3390/ani16010013 (PMC12784933; doi:10.3390/ani16010013)
Supplement: Supplementary file 1 [file animals-16-00013-s001.zip › animals-4012998-supplementary.docx]

>*MrPAX6*_cDNA

ATGTTGTCCCCAGTAGACGCCGGGCCACATGGACCTCATGGCCCGCATGGTCACCACCCGTGGGGCCCCACCACTTTGGACGACATGTCGCATAAGGACGTGCTCGATTACTCACTGGGACCCTCGGGTGGTCACAGCGGGGTGAACCAACTCGGAGGCGTTTTCGTGTCTGGGCGCCCCCTGCCCGATACCACGAGGCAGAAGATCATCGAGTTGGCACACTCGGGGGCCAGGCCTTGCGACATCTCCAGGATTCTACAGGTCTCTAACGGCTGCGTCTCCAAAATACTGGGCAGATACTACGAAACGGGTTCGATCCGTTCCAGAGCAATTGGGGGATCCAAACCCCGCGTCGCAACGGCGGAGGTCGTAGCCAAGATCTCTCAGTTCAAGAGGGAATGTCCTTCCATCTTCGCCTGGGAGATCAGAGACCGACTGCTCTCCGAAGGCGTCTGCTCTTCGGACAATATCCCAAGCGTGTCGTCCATCAACCGCGTCCTCCGTAACATCAACGCGAAGGAAAGCAGCGGCAGCGGCTCAGGGGGCGCACCGGTCCCTGGCGGACCCCCTGGTGGGGGAGGAGGTGGCGGAGGAACGGGCGGAGGAGGAGGAGCAGGAGGAGCAGGAGGAGGAGGTAGCGCCGTGGCTACTGCAGGAGCTGGAGGCGGTGGTGCCGGCGTCGGAGGGGGCCAGACGATGGGTATCGGAGGCGGGAACTCCACCAGCAGCAGTAATTCGAACACGCCTACCAGCACCCCGACCCAGGACCCCATGTACGATAAACTCCGCCTCCTGAATGGTCAGTCGACTTGGCCCAGATCACAATGGTACAGCGGACCAGGCAACGATATCACCTCCTTGCCCGTGGATTCCCCGCAGTCGGCTCCCTGCGCTCAGCAAGATATCGTCAAGAAGAGTGACGCCGGTGAACTTAGCGAAGACAGCAACGCTGGCAGCAGTGAAAATAGCAACAGCGAAGACCAGGCGAGGCTCCACCTGAAGAGGAAGCTCCAGAGAAACAGGACCTCCTTCACCAACGATCAGATTGACAGCTTGGAAAAAGAGTTTGAGAGAACTCATTACCCTGACGTATTTGCCCGCGAGAGACTGGCTGCAAAGATAGGCCTGCCGGAAGCTAGGATACAGGTGTGGTTCAGCAACCGAAGAGCCAAGTGGCGGAGGGAGGAGAAGCTCCGGAACCAGCGGAGAGCGGCTGAGGGCGTCGCTCCTTCTTCGCCCACGAGAATCATCAACAACTTCACGCCCTCGCCTATGTATACCCCGCTGCCTCCACCGCCCATGTCCGTCGCCGACACTTATGGGTCTATGGCAGGCGGAGGTGGATTTGGCATGGCATCGAGCGTGGGCGTAGGGCCAGGTGTGGGCGTGGTGGCCTCAAGCCCTAATTGCTTACCCCAGCAGCAGCCGCCAACCATCGTTCACCCAGCGGGGACAACGAGCGTGGGACTGGGTCGAGACCATTCTCATAACGCCCACAACCCTTACATGAGTCGGTCTTACGACTCCTTGTACACCCACGCCCGCGCCTCACCCACTTGCCCCCCCATGCTATATCACCCGGCTTCGCACCACCCTCACCAGAACCCCCACGCCCCCCACGAGTACAACACGCCCACACCACCCAATTCACAAGCCGGTCTTCTCTCCCCCGGTGTGTCAGTGCCTGTGGCGGTGCCGGGTCAGCACCACGACATGAACGCCCAATACTGGACTCGATTGCAGTAG

>*MrPAX6*_DNA (Antisense strand; exons are indicated by yellow background shading)

CTACTGCAATCGAGTCCAGTATTGGGCGTTCATGTCGTGGTGCTGACCCGGCACCGCCACAGGCACTGACACACCGGGGGAGAGAAGACCTGCAATTCCAAATTTAGATTTTAAATAAATACGAGACAAATTTTTAAGTAGAATTTCTAGTCAGAATCTACTTAAAGAACCGCTTATAAAGGTTGATGAAGAGCAACCATTTATTCCTATTCATGTTGGAAACTCATTCAACCCTCTTTGTATTGCATAATTCAATTAGTAAATCAAAATCAGTATTTGCTATAATTAATTGATTATATTAAGATAGTTAATTGATTATATTAAGCAGTGACCATCAAACAAGCCTTTCAAAAAATAGTCTCAACACTGGCTACTTGATACCTTCAGAAAGACGAAGAGAAGCTTACCGGCTTGTGAATTGGGTGGTGTGGGCGTGTTGTACTCGTGGGGGGCGTGGGGGTTCTGGTGAGGGTGGTGCGAAGCCGGGTGATATAGCATGGGAGGGCAAGTGGGTGAGGCGCGGGCGTGGGTGTACAAGGAGTCGTAAGACCGACTCATGTAAGGGTTGTGGGCGTTATGAGAATGGTCTCGACCCAGTCCCACGCCCGTTGTCCCCGCTGGGTGAACGATGGTTGGCGGCTGCTGCTGGGGTAAGCAGTTAGGGCTTGAGGCCACCACGCCCACACCTGGCCCTACGCCCACGCTCGATGCCATGCCAAATCCACCTCCGCCTGCCATAGACCTGAGATTCGGTAGGATAAATTAGAGAAGAAATTGATTATCAAAACGACATTTCTTTATGGAATAAAGAAAAGAATTTGTACGAGAAACAGATCAAATCCTTCTTGGTGCAGTGAATTTTTAAATCAATAATTGCATCATGAAATCCCTGTTATCATTGCTCATTGGCTCGCAAAGGCTCACCCATAAGTGTCGGCGACGGACATGGGCGGTGGAGGCAGCGGGGTATACATAGGCGAGGGCGTGAAGTTATTGATGATTCTCGTGGGCGAAGAAGGAGCGACGCCCTCAGCCGCTCTCCGCTGGTTCCGGAGCTTCTCCTCCCTCCGCCACTTGGCTCTTCGGTTGCTGAACCACACCTGTAGAAGAAGAAGAATAAGAAGAAGAAGAAGAAGAAGAAGAAGATGAATGACTGTGATTGTTGAGGCAAATGGGTCTTACCCTTGATCCTTTGACTTCATCACTTTATTTTAATCTCTGTTTTCTGTTCTAGATCTGATAAAACAGCTGGGATTAATCTGATCATATTTCATAATTCTTCCACTACCTTTGCCTGCAAATACATATATTGGCTTGAAAAGGAGGAAGGGGATATCTTCGACTTAAATTCAAAAAGGAATCGCACAAAATTGTAATCGTCAATTACTTAAACTTAGGCTTACAATGGAAAGATTAGTTTTCTTTCCCACAAACAATCCAGTGAATCATGTTTTGATAACGTGAACTAATATCCCAGTAAAATCAACAACAGTTATGAATTTAGGTTAACTAAGATACATCGCAGGAGAGAGAGAGAGAGAGAGAGAGAGAGAGAGAGAGAGAGAGAGAGAGAGAGAGAGAGAGAGAAGAATCTTTTTCTTACCTGTATCCTAGCTTCCGGCAGGCCTATCTTTGCAGCCAGTCTCTCGCGGGCAAATACGTCAGGGTAATGAGTTCTCTCAAACTCTGAAAATAGAAATAAGTAATATTGCAAGTTAGCCCTTATAAATCCTAAAGTAATTTTTTCTAGATTATATAAAAAATCTGCCTTAAGTGATAAACAAGAGGAAAATTCTACTTGTATAGTCTGGGAAAACACAAAGAAACACTAACGTTTTAAATATCCATAAGTAACAAGTTTCTTTGATGGAAAATTAGCTAGCGTCACGTTCATTGTTACTGACTACAATTCATGAGAGTAACATAATGTAAATGATTCCTTATTGAGTATCATTTAAGCTCTTGAAAAATCATTACAGTGAAAAGGTTAATTTACACACACATATGTATATATATTATATATATATATATATATATATATATATATATATATATATATATATATATCTATATTTCAATATATATACATATATATATATATATATATATATATATGTATATGTAGATATTCATATATACATATATATGTGTATTTGTATATATATATATATATATATATATATATATATATATATATGCATATATATAGTTTTTACCTTGCCAATCTTTATTCGAGAATGTCCACCCTGCTAGTCTTCTGTTACGGGCTGCTCGATGAGCAAGAGCCCGTGCTGGCATAAAGCCAACTCAGTCAACAACAACAACTATTCTTTTAACTTCTTCCTTTCCCGCATCACTCTTGCCAAAATGTGTATATTTTGCCTTCACTCTTTAATTCCGTCACACTCACAACCATTTGTTTATTTGGATTTTCACTTTTACCTTTTGTCACTCTTTCCTGCAGCCAAATCACTCTCACCTTTTTCCAGGCTGTCAATCTGATCGTTGGTGAAGGAGGTCCTGTTTCTCTGGAGCTTCCTCTTCAGGTGGAGCCTCGCCTGGTCTTCGCTGTTGCTGTTTTCACTGCTGCCAGCGTTGCTGTCTTCGCTAAGTTCACCGGCGTCACCTGCGGTTGAAGAATAATTAACTGGTGAGAAAACTTTGAAAGCATTGGTAAGGGTTACAGTGTACATGGATTTGGATATACAACAGAAATAAACTTTATGTGAATAATGAATGTCAATCATCAGGTAATAATTATTAGATGGGAATGCTAAGTTCCCTCTCATGGCCTTAGGAAATGAAAGTGGGTTTTTATAATGGGCTACAATTTCAGCATCAGAAACAAACATAGCAAGTGTATTCAGGTTGAGAGCATATTTTTTCAAATGGTTAACCGACTGTAAAGCATTTAACTTACGAAAAATCATATCTTTGTGTGTGCGCGTGTGTGTGTGTTTGCGGAATACTTCAAAAACAGTTGAACAAACTTTGGTGAAACTTGAATGAGATAGTCCTTTTGTAACTTTATAAAGATAATCAGCTTTCGAACGCAAATCGGACCAGTAGTGAGATGATTTCCTTTGCAGCAAAGTCAGTTCTGAGCTGCTTTCCCCTAAAAGTTAATCATCTATTCTAAAGAGAGCGTAGGGAAAATATTTTCCAAGTTTCATCCAACTCTGCTTAGAAGTTCTTGAGAAGTCTTGTTGAAACACATTAATATAAATGAAATAAATTCAATACTACGCCAACCAGCAGGTCATTATTTTCACTGTGCAGTGAGGAAGCTAGTTTCATTTCAGCAAGTGTCTGTGGACTTTTAAATCTGCCTTGCACTTGGTAACTGGTAAGGACAGACAATGCTTGCAAAGTGTAAGTAAATGAACGCTCTTAGATAAACAGTAAATGAACAAATCCCCAGTTGCTAAATGAGCAACGAGAAAATCTGAAATCGGTGACATGGCAGACAAAGGACAATAATTGTCCTTTTTTTCGCCAAGGGAAGAAGCTGACGCCACAGGAGCTATATATGCAAATAAAGTATAGATGACAGAATATGTAAACCGAGTAATAAGCGTGGTAAACGAGCGAAGCACCGCTACTCTAAGGATGACAAAGAGCAATATTAAGGAAGGAAGCCACGCCCCCTTTTGCGTTTCATTCACACTTTATTATGACATGATGCCGGAAACTGCCAGATGCGGGATGCCTCCGCCACCTCTGCCGACCGACGTCACCCTCTACGGACACATCCGATGCGGTTTCGTTAATAATTTTGACGTCGCGGAATTTCGCCCAAAAGAGGAGAATTAGACTCGATTTATCACATCAATTTGACAACCGGGTTTTTTCTCTCTCTCCCGTCCAGATATGTAAATGAATCGTGAACTGGAATGGGAATGGAGACATAAAACAGCGGAGGTAAAATGGTAACTGGCATCTCTCATATGGAGATAAGACGTACATATTCCCAACGTGGCCAGTCAGGCAGAAGGCGCAGATAAGTCCAGATGCTTTCGGGAGAAGGGGGAATGGAAGATGATGATAGTGGAGAGGGGAGAGAAGGGGAATAGGCCCAAGCCAAGGAGGAAGAGAGGAATAGAGGGGAAATATTCCTGAGAATGGCAGATGAACACAACTTCAAGATTCTCGTAGATAACTGTCTGCTTGGAACTTGAAATGGAAGATGAAAAAGAACCTTTAGGAAATCCGTAAGAAAAGACAATAGCATCACCAAATGTCGACCAGTTTAAGAATCATATGAACTCACAGAGTGAACACAGATAAATGTCAGAAGAGGAGAGTCGTTGCTCTTTGGAAACACTAAAGAGAGAAGAGAATTTAGATTTTTCTAAAAGAATAGAACCATAAATGACTTTTGAGACTTCATATATATATATATATATATTTGCACATATTGTTCATTTAACATAACCAACTAAGTTCTATATATATATATATATTATAAATATTCGTCCTTATCAACTCAATTTTTCTTGACGTGCATTTACACACATATATATATATTATATACATGCATATATATGAAAGTAAAATTGATTTTTATTTATACTGTATATATATATTATTGTCGATACCATTATATTTGTTTTCTATCGTAATTATTCTGTCAAAATTGTTTTCAATTATTTTCCTTTTCACTTTTTACTTGAGGACGATTTGGCTTTTCTGATGATTTTTGTCACTAAAACTTACCCTCTTAATTCTCTTGACCGTCTTTCCTCTTAGCGAAAAACCTGAAAATATAGCTGGCTGCACTATTATAAACAACGCCAAAATCACACATATATACGAAAACAACAAAAACAATATATATAAAAATATAAACCAAACAATGACATATAACACATCCTCATGACGTGCATTTAAGCTGAAGTTACGTTCCTATGACTACTGAAAGCAAATTGATTTTTATTTTGCTTTCATTGTCAATACATTCATTGTGTCGTTGCCTGTAGAAATTGTTTTAATTTTCCACAGTGGTTTTTCTATTCGATCATTATGTACTATCAGATATAAACCAAATAACCTCTACGCTTAAAACAAAGCCAATATCGAGTCTGCCAAGGAGGGAATATTCATTTAGCTTCTCTCGTAGAAATTATTCTGCCGCAGTTGCATTTGCCGTGCATGCAGTGATTGTGCTTTCCTGGTCCTCTCTTTTGCAAGAGGCGGCCGTTAATGTTATGTGTTTATTAGTCGAGTAAATATTCACGAAAGTGGACTTCTTGCAACTCTTGCAAGCCCACAGTCGTGCTTGCTGTCAGGAGAACATTTACTGGACTTGTAAGAGTTACAGATTCTGTTTATGGTCATTTTAGTCCTCACTATACAGTATTGATGATGATGATGATTATGATGATGATCTTATCTTCTCTTCTGAAACTATGGCTGTCAAACCAAGCGCTGGGGAACTTTCGGCCATTCGGCTCTTGAGACAGTGAAATGAGGGAGTTGTGATGGTTGAACAGCAAGACAGAGATCCAGAAAATAAAGGGGATGAAATGCAAGGATCTAAAGGTGGAAGTGGGAGAAAACTCCATTCTTGCATTAAGAAGTAACAGTTAGTGAAGTTGGACAGCAAGACTGAAGAAAAGAAGAGGGAAGGAGGATAAAGTAAAAGGCTAAAAATTGGCTGCAGCTGTAGGAGCCGAAGGGACGCTGCAAACACCCTCTAGCAAAATGCATACAGTGCAGAATGTGAGGTGAACTGACGGCAATACCTCCCTACGGGGATGATGGTGATGATGGTGATAAAAATCTAGATAATTCCAGAAGCTAATTTTTTGTGCAATTAAAAAAAAATAACACTACACTGTGTATATCAATCGGAGATGAAGCGTGCTATAATTAGATAAACACTTAACATTATAATGATCTTTAATAATAAAATCCGAGAGGATCTGATCTTGCTTGTCCTCTCCCGGGTGGCAGTAGGGGAAAACATGACACAGCCACCCAACCCCTGCAAACCGGTTTGTCCGCCCGGGTGGGGGCGGGGTAGGTACGGGATCGATAGGGCAGTGGAGACAGCAGCGCCGGGTTCCAGCGCGCCAACAAGTTTGTATAATAATAACGGTTCAAAAAAGCTATCAGAACTTACGAATGAAATACCAGCTACATGGAATGGAAAGGCATTTTTAGTCCTATTAATGTAAAGACGTTAACTGAAGGCAAAACAGTCAAGTAATGAGGTCACACAGCTATGAAAATACTACCTCAGAATGTAGCGAGAAATGATGATTCGCTAAACGGTATACTAAAAACAGGTAAAAAATGCGACGAAGTTTCTTCGGCGCAATCGAGTTTTCTGTACAACGTATAATGCAGTACGAAACTCTCAGCCAAGGCCCATGAAACTTTCAGCCACAGCCCGGTGATGGCCTGTGTTGTTGGCACCTATAGCGGTGCCAGACGCATGATAATGGCTAACTTTAACCTTAAATAAAATAAAAACTACTGAGGATAGAGGGCTGCAATAAAAATAACAGTATATGATACGAGCAACTGTGATAACATACAACGGCTACTGAGACAATATAAACGGTGATTATTAAAGTCTTTTTTTATAAAAACTAAAAGGATTAATTTTTACCACATCTCCAGTGATCATGGTAAAAGAAAAATCACTGACCTTCATTTTAACAGTTTTATACGAGCAACTTTAGAATCGAGTAAGCTAAACCCTACTCTATCAATGGATTGTATAAAAATCTGTGGCGGGTATTATGATTCTTCATGGTTAGAACCATATCATCCAACATAAAATGGTAACTACAGACAACTATAATATGACCTATATATATATTATATATATTTATCCGGATATTATATATATATATATATAATATAATATATATATTACATATCAACATATATATATATAAATATATATATATATATATATTTATATGTGTGAGTATACACTTGTATATAGGCAAACATCAAAAAATCTTACTACATATAACTATATATATATATATATATATATATAGTATATATATATATATATATATAAATATATATATATATATATATATATATATATATATATATATATATATATATATATACTGTATATAGTAAGATTCAATATAAGAGGCGATAAAAGAAACCATTTGGAAACCATTAGGATTCAGGACACCTGCCAAAGATCCCTTTATAAATTCCGTTAACACACTAGTTCACTTAATATGACATCTGGAAATAAGTAACAAATGAAAGCCAACGACTCTCAGTTACCAGGATTACTGGACATTCAGCTATAGTTTATTGTGAGTTATGATAATTTAAACTACAATTATCACAGTTCACATTGTCGCCGCAGTCGTCTTATTTGTCGCTGTGCTTTTATATATATATATATATATATATATATATATATATATATATATATATATATATATATATATATATATATTATATTACACACACACACACACACACACACACATATATATATATATATATATATATATATATATATATATATATATATATATATATATATATATATATATATATATATACAATATATACAATGTGACTTCTACGAGTATTCTCCTTTGCCGTTTGGTGGAAATATCACAAGATACCGAAATACAATGGAAACATAATTGACAAAGCGGTATACTTTTAAAGCATTCATACCATCGATCGTTTTGCATCTAAAATACCCTATTTACACACCATTGAAAATAAAAACATCACACCTATATCACCTGGTAATTTCCTGGCAGTAAAGACACTGTATTCAGGTGTAAGAATCAAATTAATCCTTACGAGACACTGTCTTTTTCCGAAGCGCGCAAGAGTTCTGTTGTTGATAATTTTGATAAAAATCGGGAGCTCTCATTTTGGATTGAAATTACTGAAATGTTTATCACCAGTGTTAAATGCCCCAGGGACAAAGAGCTCCTGTCACAACAGAACTTGTGATGATAATCGTTTATTCGTAATTTTTGATAAAAACTAAGTTCAGTGCCTCTTCATTATTTTTCCTAGCAATAATTACTTAGAAAATATCCATGAATCACAATACGATGATATATAAAAACATATATTATAATATATAAAAAAACATATATTATACTTAGTTAAGAAGTTCTGAAGAAGCACAAACATCATTTAAATCAGTAGAATGACAAATACACCGCACGAACATCTCAGTATTTCTTTCCATACGGGCGTATACGTATGATATCGGACACATACAAGATACAAGACTTCAAGAGAAAACTTGTATTGGCTTCCGAGAAGCTGAAACAATAGTCTCCGTCTACCCCTTCTTTTTAGCCCCCCCCCCTCCACCCCAACACCTCCCGATTCTAGCATCTTGGAGGTAGGGGTTCGTGGGGGAGGGGGAGGGGGGGGGGGGAGAGGAGGTCCCAGCCCTCGAGGAACTGCCATCTACAAAACAGAAAGAGGAAAAAGTGGGAAAAGGAAGGTGGAAAAAGATGCCAAAGAAAGCTCGCTTTCCCTCACTTTCTCTCCTCACTCTCTCTCTCATCATGAAATATATAGATGATGAGACTGAAGGTGATAGAGCGTAAAAGAAAACGGAGATGGGGGAGGGAATGGGGGGAAACACTCCCCTTGGAAGGGGGGGACTAAAATTAACTCCTCAGCGTTTTTCTAATCTAGTAATAGAGGAGTCCCTGCCTTTGACTTTAATATTGGTAATTTAGTGACTTCTATCCCACTCCCCTTCCATGGGGAATGGTGGGGGTGATGGAGGGGGTGTGGGGAAACGGGGTGGGGGGCATGCAATGGATCTCGGCCTCCTTGCGGAGGGAGCACAGAGGTGGGAATGGCTTTTGACTCCCGCCAATGGCTCTTTAGATAACAGGAGGCGAGGGGGGATAATAGGCCCTCTGTGAACCTATCATAAGGGTTGAAATGGACCGGAGGACACTTTATTGTCACCCCCATAACGGTGATCGAAATGTCCATTAGAAGGACATTTCCTCATTTTTCACGCCTATTATACTACAACTTTATATTTCTTAAGGTTTTCTAAAGTCTCGTGAATCACTCTTTCTGACCTTAGAATATTTAGGTTTGCCTTTAATTCATATATATATATATATATATATATATATATATATATATATATATATATATATATATATATATATATATATAAATTATATATATATATATATATATGTATATATATATATATATATATCATATCTATCTATCTATCTATCTATATATATATATGCCTATATATATATTATATATATAAACCAAATCCTCTTATCCTCCAGGAGATATAAGCAATTATAATGATCAGTCTAACAAAGTACAAGTAATATCGGCAGCTGGGAGTAAAGAGCCTCAACGAAGAAAATAATTCTTCTAAGTTCCCTGTAAGCTCAAGTGAGACTGGAGGAGCAACGTAACTGCTGCTGAGGTCTCAGCAGGTAGACCTACAGTATACGCCAAACGGCTCGCAAACCCCGGAAACAACGACCCCACTGAAAATCTACCTTAGGAACGCTAAGCTCTTGTTTGCCAGAGCAATTATCAATCCAAGAGAGTGCCTTCAACTCGGGACTACCGCACTGTGAGGTTCACGTGAAACCGTGAAACCAACATACATACCACGGCCCTTACGTAACGTGGAAAAGGTGTTGTAATGGAAGGGCTTTATTATCTAGGTAGCACGAACTATTTGTAGTTTTGACGAGCTGGTGCTAAACCTTCTATGTACGTATACGAACAAGCTGATTATGAGAAAGTTTTGGATGGATTGAATTCATCCTCGATTTAGCAGGTAAAAAGTGAATGCAGCGTTACCAACGTCTCCTTTTTTTTTTTATCTAGAGTCATTCTGTATCAGGAGAATCAAATCAGCTCATTGAAAAAAATTTAAAATCAAACTCATTTCGATGAAACCTTTGACTAAGACCCAAGAAGATAAGGTGAGTTGAGTAGAGGTCGATTTTTCAACTCTCACGAGCAATACGTCACATGGTGCGCTCATCGGTAGCCGCTGCCCGGCCCTGGCAGTGTTACCGAAACGTGGAAATGTGGCCTCCGGTGTGGGAGCTTTGATGCACAAGACGATCGTGTGCAGAAGTGGCATTTCCATTTTACGGTTTAACACACCAATTGTGGATAAAATTTCCGCACTCTTTTTAGGGGTTTATTGTGGTCAGATATATTATTTTCTTATATTATATGATTCACTGACCTATATTCTCTGTCATTTGCATATTTACACAGTATATATGTTTGTATGATAATGACTGAAGAGAGAGCCATCAAAGAATGGTTGTTTACGCCGAACCATAAAGCAGCAGAGGTCGATCGAGCGATGTCTTGATTGACACGTAGAATGCAGACGACATCAAAGTGACAGCTTTAGGTTTCCATTTCATTAAGTTAACTAAATCTATTTAAAATTGTTTTAATAAGATTGTGATTAACTTGACAAGTGAAGTTTACTGAAAGTGAACTTTAGACTGTGAAGGAAAGCATATATCGTTTTATATACGGTAGATTTTACCGATGATTGAACGAATTTAAAATTTAGGAGAACGTGACAGTTAGTGGTGTGGATATGTGGCAACACTGCGGGCCGCCCGCTGGCCAAACCAGTACCTTGATCCCCTTGCTCGAGACGAGAGCAAGGCGGGAGTTTTCACAAATTTGTGGCAATCCTTGAGGTTTTTGAATCGAGTCCGTTGTTGCCTTGGTGATATGCTATTTATGGATGCTGTAACACAGAAAGGGACAATGTTGGAAGTAAAAAAGGTTAGCTTTTACCGTTTCCCTAAATGGGTAGGAAGTAGATGAACATTTGCGGAAGTAGTGACCATATAAACACCAAAAAACGTAAGAGTGTGTTCGCTTCGTTTTCAACAAAGCGATTGCGTAAGAAGCCTCAGGCATGCATTGCTTAACTTCCGCCTGGCAAATGCAAGAAAACTTGAAGCTTGATGCCATACAGAGCCAGCGTTTCCGCTGCATGACAGTGCCGGTCTTGATTCAGGTATTTAACATCTTGACTCTACTTTGGTCTAGTCTACTGTTACATTAAATAAAACTGTGGATAAATATGTTAGAACTATAGGCTTGTATGAATACTTCTATCAATTATAGTGCATAAATATATCTTCCGCATGATTCCTAACGCAGGTAAACCATATTTGACTGGTTAAGAGTGATAAGTTTGAAATTAGGCTTAGACCTATGTGTAGTTAAGGTTTACTGCAAAGGGGATATACCAGTCAACACGAGAACACACACACACACACACACGAACATTTTATTGCAATTGTTTTCATGTGCATGAAAAGGGCCTATAATTTGTCGAGGTAAAGAAGGGGACAGTTATTTTCAAGGGAAACAGAGAGAGAGAGAGTGACTGAGAATGAACCATAGGTTTATAAGTATAATTCTAGCAGTTAAAGAGCACATACATTATGTAACATCTTCCATTCTCTTCCAAATACAAATAAACCATGTTCGCTGCCTAAGATTGGCAAAATTTGAATTAAGCCTAGACCTATGTTTTTTTAGAACAACTGCAAAGAGAAAATGCTAATCAACACAAAGCGAGAGAGACTGGGCAAAATTTCACTAGAATTGTTTTCATGTGTATGAAATAGCCAGTTATTTGTTTGGTAAAGGAAGGGGAATGTTACTAGAGAGAGAGAAAGAGAGAGAGAGAGAGAGTGAGAGAGAGAGAGATTTTCTTGCATATGAATGCAGCACCTGTATTCATATGCCGTCACTGAGGACAGCCATCCTCCTGGCAGTCTGCGCGGTAACTCACCTTATCTTGCATCTTGCCTACGACATTACTTTTTCCTTTGCTTATACGGCAAATATATTGTTTCCTAGTGATCAGCTCAGTATCGCCATATAAAGCCTTACATAAGGCTCTCAATAATGTTATTGTTATAAGTGCGGTTGCCTGGGATATTTTTTCATTGTTGGGAATGGATTTGTCCCCGATTGTCACTGATATTTGTAGAGTAGCTTTGCTTTCTTGCCTTTGCTTCAGGGAGACTGGTAATTCTTGTTGCTTTAATTAGTTCCATTAGTTTATCAGTACTATTTATAATACTTGGCTTACCAAACTCCGTATCATCTACAAGTGTCCCTTCAAGAAAATGAATACATTTTATACATTTATTCATGCCACACACACTTAATATTATTACATAAATACACACACACACACACATATATATATATATATATATATATATATATATATATATATATATATATGTGTGTGTGTGTGTGTGTGTGTGTGTGTGTTTGTGAGGATGTGTCTATTTATGTATGTGTTTCTGAAGGAGAATGTTAATCTTAGTATCAAGAAAAGCAAGTTGGCCGTATACGCATCACAAAATTTTGATTAGGAAAAAAGTGAATATTAAAATGCAGCACAGGAATCTTAGTTCTATTTTGAACCCGGATTTACGATGGAAATGCAAACGAGAACACGAAGTATATTAGACAAGGACCCCACCAATAATAAGGATTGAGAACAGTAAAGTCATTTAATCACTGTTCGATAAATAAAATGTTTGTAATTAGCATGATATATATTTACAGCTGCAGTGACATTATACTCTGTGCATTGTTAGTCATAGATTTTCTTTAACTGGAGATTTTTAACTTCTCTGAAAGCACTGTTAATGACCCAGCAATAAAAATTCCTTCAGTATGAGTACAAGGGGCCTTTAGGACATATGGTGATAATTGCATACAGTGAATGTTGATGGTGATGTTGGATATCTGGGGTTGAGGAAGAGTCTTGTTGGTGGAGTGGGGTGAGGGAGAAGGTTATTGTGGAGTGGGGTTGAGGGATAGGGTTGTTGGTGGAGTGGGGGGGTTGAGGGATAGGGTTGTTAGTGGAGTGGAGTGGAGGGAGAGGGTCATTGGAGTAGGGTTGAGAGATTTGGTTGATGGAGGAGTGGGGTTGAGGGATAGGGTTGTTGGTGATGGGTTGAGGGATATGGTTGTTGGTGGAGTGGGGTTGGGATATAGGGTTGTTGGTGGAATGGAGTTGAGAGATTTGGTTGTTGGTGGAGTGGGGTCGAGGAATAGGGTTGTTGGTGGAGTGAGGTTGAGGGAGAGGGTTGTTGGTGGAGTGGAGTTGAGGGATTTGGTTGTTGTTGGAGTGGGATCGAGGGAGAGGGTTGTTGGTGGAGTGGGGTTGAGGGATAGGGTTTGTTGGTGGAGTGGGTTTGAGAGATTGGGTTGTTGGTGGAGTTTTGTTGAGGGTAGGGTTGTTGGTGGAATGGGGTTGAGGCATTGGGTTGTTGTTAGAATGGGTTTGAGGGAGTTTGTTGTTGGAGTGGGGTTGAGGGATAGGGTTGTTGGTAGAGTGGGCTTGAGGGAGAGGGTTGTTGGTGGTGTGGGGTTGAGGGAGAGGGTTGTTGGTGGAGTGGGGTTGAGGGATTGGGTTGTTGGTGGAGTGGGGTAGAGGAATGGGGTTGTTGGTGGAGTTTGGTTGAGGGATAGGGTTGTTGGTGGAGTTTGGTTGAGGGATAGTGTTGTAGGTGGAGTAGGGTTGAGGGATAGGGTTGTTGGTGGAGTTAGGTTGAGGGATAGTGTTGTAGGTGGAGTAGGGTTGAGGGATAGGGTTGTTAGTGGAGTGCAGTTGAGGGAGAGGATTATTGGAGTAGGGTTGAGGGATTTGGTTGATGGTGGAGTGGGGTTGAGGGATAGGGTTGTTGGTGGAGTGGGGTTGAGGGAGAAGGTTGTTGGTGGTGTAGGGTTGAGGGAGAGGGTTGTTGGTGGAGTGGGGTTGAGGGATTGGATTGTTGGTGGAGTGAGGTTGAGGGATAGGGTTGCTGGTGTAGCAGGGTTGAGAGATTGGGTTGTTGGTGGAATTTAGTTGATGGGTTTGTTTGTTGGTGAAGTGGGGCTGAGGGAGAGGGTTGTTGGTGGAGATTGGTTGAGGGATAGGGTTGTTGGTGGTGTGGTTGAGGCATTGGGTTGTTGGTGGAGTAGGGTTGAGGGAGAGGGTTGTTGGTGGAGTGGGGTTGAGGGATAGGGTTGTCGGTGGAGTGGGGTTGAGAGATTGGGTTGTTGGTGGAGTTTGGTTGAGGGATTGGGTTGTTGGTGGAGTGGGGTTGAGGGAGAAGGTTGTTAGTGGATGTTGGTTGAGGGATTGGGTTGTTGGTGGAGTGGGGTTGAGAGATTGCGTTGTTGGTGGAGTGAGGTTGAGGGATAAGGTTGTTGGTGGAGTGGGGTTGAGGGATTGGGTTGTTGGTTGAGTGGGGTTGAGGGATAGGGTTGTTGGTGGTGTGCAGGTGAGGGGGAGGGTTGTTGGTGGAGTGGGGTTGAGGGATTGGGTTATTGGTGAGTAGGGTTGAGGGATAGGGTTGTTGGTGGACTGAGATTGAGGCATTGGGTTGTTAGTAGAATGGGGTTGAGGGATTTGGTTGTTGGTGGAGTCGGGGTGAGGGATAGGGCTGTTAGTGGAGGTTGGTTGAGGCATTGGGTTGTTGGTGGAGTGGGGTTGAGGGAGAGGGTTTTTGGTGGAGTGGGGTTGAGGGATTGGGTTGTTGGTGGAGTTTTGTTGAGGGATTTGGTTGTTGGTGAAGTGGGGTTGAGGGATTGGGTTGTTAGTGGAGCGTGTTTGAGCGATTGGATTGTTAGTGGAGTGAGGTTGAAGGAGAGGGTTGTTGGTGGAGTGGGGTGGAGGCATTGGGTTATAGGGTTGTTGGTGGAGTGGGGTTGAGGGATTTGGTTGGTGTACTGGGGATGAGGGACAGGGTTGTTGAAGGAGTGGGGTTGAGGGAGAGGGTTGTTGATGGAGTGGGGTTGAGGGATAAGTTTGTTGGTGGTGGGGTTGAGGGATTTGGTTGTTGGTGGATTTGGGTTGGGATCTTAGGGTTGTTGGTGGAATGGGGTTGAGAGATTTGGTTGTTGGTGGAGTGGGGTTGAGGAATAGGGTTGGTGGAGTGGGGTTGAGGGATTGGGTTGTTGGTGGAGTGGGGTTGAGAGATTGGGTTGTTGGTGGATTGGGGTTGAGGGATAGGGTTGTTGGTGCAGTGGGGTTGAGGGATAGGGTTGTTGGTGGAGTGGGGTTGAGGGATAGGGTTGTTGGTGGTGTGTGGTTGAGGGAGAGGGTTGGTGGAGTGGGGTTGAGGGATTGGGTTGCTGGTGGAGTAGGGTTGAGGGATAGGGTTGTTGGTGGACTGGGGTATAGGCATTGGGTGTTGGTAGAGTGGGGTTGAGGGATTTGGTTGTTGGTGGAGTCGGGTTGAGGGATAGGGCTGTTAGTGAAGGTTGGTTGAGGCATTGGGTTGTTGGTGGAGTGGGGTTGAGGGAGAGGGTTTTTGATGGAGTGGGGTTGAGGGATAGGGTTGTTGGAGGAGTTTTGTTGAGGGATTTGGTTGTTGGTGGAGTGAGGTTGAGGGATTTGGTTGTTAGTAGAGTGTGTTTGAGGGATTGGATTGTTGGTGGAGTGTGGTTGAAGGATAGGGTTGTTGGTGGAGTGGGGTGGAGGCATTGGGTTCCTGGTGGACTGGGGTTGAGGGATTTGTTTGTTTGTGGAGTGGGGTTGAGGCATTGGGTAGTTGGTGGAGTCAGGTTGAGGCAGAGGGTTGTTGGTGGACTGGGGTTGAGGGATAGGGTTGTTGGTGGGGTGGGGATGAGGGACTGGGTTGTTGGTGGAGTGGGTTTGAGGGATAGGTTTTGTTGGTGGAGTGGTGTTGAGAGATTGGATTGTTGGTGGAGAGGGGTTGAGGGATAGGGTTACTGGTGGAGTTTGGTTGAGTGATAGGGTTGTTGATGGAGTGGGGTTGAGGGATTTGGTTGGTGTAGTGGGGATGAGGGATAGGGTTGTTGGTGGAGTGGGGTTGAGGGAGAGGGTTGTTGGCGGAGCGGGGTTGAGGGATAAGGTTGTTGGTGGAGTGGTGTTGATGGACTGGGTTGGTGGAGTGGGGTTGAGGGAGGGAGTTGTTGGTGGAGTGGGGTTGAGGGATAGGGTTGTTGGTGGAGGGGTGTTGTGGGTGGTTGGAGGGGTGGCATTAGAGGGTGTCGTTTGTGAGACACCGAGAGGGCAACATTTTGCATATCGAAGACAGACAATAGTGACCATCATCAAAAGGACGTGGAGCCTTTATTTTCTTCATAATCATCCAAGAGAGTCGAACAAGAAGAGGACGAGGAAAAAAAGAAGAAGAAGAAGAAGAAGCAGAAGAAATAGATTATACCTCGCACTGAGTGAAATGCCAATGTTTATCTGTACCTCATTATTAGACGTCACGATTCGATTCGCATTAAAGAATAATGAGTTAAGCAAGAAGATAAACGAGTAAGCTTTGGCGTCCGAGCTTTCTTATCTTGGCTGCAGTTACTGTTACGAGATGTAGTCACGTTGAGTGTGAATTCTTGCATCAATCTCGCTGCACTTTGGGCTGAAGTAATCTCCGCCCCGAGCCTCCCAACTGTGGGATGCCCAAGGGTCTAGGTCCGACTTGTCCTTAAAAAAAAAGACCAATGGGAGCATCAAGGAAAGTGAATGCCGAATGCTTGTTGAAGAGGGAAACGACGTTCCGAAGTGCAAAGACGGCCGGACTGACTGACAGACAGAGAGACGGCACAGACGGAAGGAAAAGAAAGATGGAAGCAAGGCCTCTCGCCCAGCCTAATACCGAAGTCTTTCTCTAAGGCCCCCCAAAGGTTCCCTTTGTGTCGCCACGCATTATAGAGACTTAAAGGCGGTCCTGTGAGGGGCCCCTTCCACTGCCCAGTGCCCTCCCCACACGCTCCCCTTCCTTCTCGCTTAACACCTCGTCCTCCTCCTCCACCTTACTCCTCATCCTCCTCCTCCTCCTCCCAATCGACCCGATGCTAAACGAGAGAATTTATGAATATGGAGAGAAAATTCGCATTCAGTCGAGGCATTGGGTGTGAAATTAAAAGATAAAACGAACGATGTCCGCCCGAACTCCCCAAGAGAAGGAGCAGGAGGAGGGAGACGCTAGAGGAGGAGGAGGAGGTGTGGGAGGAGGAGGAGGAGGAGATGGAGGAAGGAGAAGAAGAAGAAAAGAAGAAGAAGAGTCAGGCACAATACAAACATCGATCACGGCCCTGCCATCCTATTCCTATTTCTCCTTCTCAAAGGTTCGTCCCAAGGAGGACCACCTTTCACTTTTTCGTATTTCTATGAAGTCCCATTTTCATAATACACAGCGACAAGAGCCCGGGCCAAAGGAAGGGGAGAAAAGAAGAAGAAGAGGAAGAAGAAAGGTTAATAAAACATCGAACTGGGGCACACAATGAGAAACAACACAAGCATCGGAAGCTATTCTCTCTCTCTCTCTCTATGTTCTCCTGTTAGAAAAAGATCTAATGCCAATATGAGTCACAGGTTCTCCTGTTAGAAAAGATCAATGCCAATATGAGTAATATATATAAAAGGAAATTGAAATTAGTACATACAAATATATATATATATATATATATATATATATATATATATATGTGTGTGTGTGTGTGTGTGTGTGTGTGTGTGTGTGTGTGTGTGCACGTACTGTATACATGTCCGTTGACAGAGAAATTAACCCTCTCCCATACTTTTCATTCGTGAATTACTTGTTGGTCTCAAAGAGTAGTGACTTTGACATTTTTCAAACGGTATTAGAGTACGGGAAAGTCAAAACTACTTATTTGTCATCAGTAGCATCATAAATAAACAACAAAACTAATAATAATAATAATAATAATAATAATAATAATAATAATAATAATAATAACAATATCATATTCAAAATTAGATATTAATAACGTATCCATGAGCCATGTTAGTTAAACCAGTGCAAGTAATGCAAGCACGATATATGTCAGTTATGTCCCAAATAACATAATATAAATTTATTGATCATAACATTGTGACGAAACTTTGTTTTTCTTGAAGTGCAAAATAAATACTCAATTAGCAATGCACAACCCAGTTACTTATTCAACTTTCTACGCTTACGAAAACAGTTGTTATAATATAGATAATATTTTCTAATGAAGGATTAGCGGTATATGATATTATATGCATCATATATATCCGAGATGACTTAAATCCCAGTAAAAGTTTGTACTCAAAACGGTTTCTTTTACATATAGGGAAGAGAGAGAGAGAGAGAGAGAGAGAGAGAGAGAGAGAGAGAGAGAGAGAGAGAGCCATAAATTCCATACAGGTAGAGAGAGAGAGAGACAGAGAGCTGAATTTTATAACATTCAGCTTTTTAAAATCATCTTGGCTCAGATCTATATGACTTACTATAACCAAAAATATATAAAGACATTTTATTCTTACATAAGGAAGGCAAATAGATACAAATGTATAAGAATGTACATTTGAGATGTGACAAATCAGCCTCTTCCGCAATGGGAAGTTGTTTATGCAGACAAGAAGCGCACATCAAGCTGGACATATTGCAAGTGGCATGGCATGTAATTTGTATCATTTTATATTGTTTTACGATGAACTAAGTGAAAAAACAAAAGAGCAAACTCAATGGTTTTTGTACTGTTTGCAGCGGGAAGTACTGTTTCAAAAACTTTTTTTTTTTTGTCAGCGAGAAATCCAACAAAAACTAAAGGAAAAGTGGGAATCGAAAAGAAATGAGATTAACATCTATGTCGCTACTATGAATTGAAGTATGCGTAAGAAAAATAATTTACAGGTGTATGTAGGGTGTGTACTGTACATACACATTGTGTACAAACGAACACAAGTGAGTGTGCAGAAGATAATCAAAGTCCATTAACGATAGTCATAGCAACAGCACCAGTAGAATTTCTGAGCTTTCGCAGCAACAACAGAAACATTACATAACAAATCATATTTGTCTAAACGCTCCTGCAGCTGTCTTGCTTAACACTTTCTCCATCTATTTTCCCCACCCACGACCCCCCTCTCTCTCTCTCTCTCTCTCTCTCTCTCTCTCTCTCTCTCTCTCTCATACATACACGAACGCATGAATACGTCAGATGGTTGTTTTCTTATAGCCTGGGACCAAATGGAATGCCAATGGCCATAAGCCGCTAAATACATGGATTATATATATTTACTTTTTACTTAAATGTCGACGATGATGGCAAAGCGCCATCAAGAGTAGATGGTAGAGCAATAATAGAAGTCTAAAATACGTGTTTCTTATATCAAAAGTTCTTATCAGTAACTAAAACAATGCCGTGAAACAATATCAAAAGTTCCTATCAATAACTGACACATTGCCAAGAAAACTACTTTGTGCTGTGATCGTATGACGATCTGTTCCCGTTAGCACAAGACCAAATTAACTGCAGATACCTACAGAACTTCAAGACTGGATTATCAATTTCAGTTAGCTACAGAGCTTAAACAGTGATAAGCAAACTAGTTGTTGCTATGTAGTACGCCAATGCTTTACGTTGCTGTCAGACTGTCCTCAAGACTTTATTGAGGAAATTGGGCAAGTCAGAAAACAGCAAAAGATTTAGTTCTGCCTTTTCAGCAAACAACAGAAATGCTGATGCACGCATATTCCCACCATTATTGTAATAGCCAGATTTATCAAGAAGGGGAAAATAACTAAACGACAGCGATTTATCGTAAATGAAAGCATAAAAACAGACTCGCCTTTCCTTTTAAAAGTCTAATAACATACGCGTAAAAAAATGAAATCCGTTTTCTATGAGAGTCGACGCCGCGCAAAGGGGGTAACATTTTTACTTGCATGGTATCGTGGCACGATCTTAATACAAGCGCGTGTGAAGCATGACTGCAAAGACCCGATAATGCTGTATTATGCTGACGAAGCACGGATGAGCTGGCAGCAAGTGTTGGGGTGAAAGGAAGGGGATGAGAGGGAAAAGAAGACCGAAGAAAGCGAAAGACAGATGCTTCGGGAAGAAGAATAAAACGAGGGGAAAGGGAATCATGTACAGGGAGTATGGAATAGATAGATGGAAAAGATACCTCGGAAAAGGGTCGGATAGGTGATAGGTAGAAACTGCTTGTAGAAAGAATTATGAGGTGAAACTTTGTAGTGATAATAATAACAGACAATGAAATATGGGAACAGACCATTCTAGTTTCCATCACTTGAGAAAAACTTAAACAAGAATGTTACATATGAGACAAAACCATTCTTTTCTGAAGACAAGAAAGGTTTGGAATTGAAATAGATAAAATTTAGAGCCAAAGACAATGTCTTAGAGATTAGGATATTAGTTGCTGAAAACTTTACTCGGTTATATATATATGTATATATATATATATTATATATATGTATATATATATTATATATATGTATATATATATATATATATATGTATATATATATATTATATATATATACACATATAATGTGCGTATGAGAAGTTATGCAAAAGTTTATAATCTGTGAATACTCGTCTATTGTATGCATTATATGTCACCTAAATATTTATGTCTCCCTAGTCACTGGCAAATTGAGAGAGAGGCAGGTCTATTAAGTGTACATGTACACACATCTCACTCTCTCTCTCTCTCTCTCTATATATATATATATAGAGAGAGAGAGAGAGAGAGTGAGATGTGTGTGCACGTACACTGAATACTTTTAATTGCCTTTGAAGTCTTTCTCTTGCCTATGTGTGTGTGTATATACACACACATATATATATATATATATATATATATATATATATATATATATATATTATATACACACATAGAGAGAATAGAGAGAGAGAGAGAGAGAGATAATTGATTGATAGATGGATAATGTCTGCGTAGACATTCAATATATGCAAAAGGGGTCGCTTTCTCTCGCACAACTAATGCATGGTGTATTCACACAAAAAAATAAAGAAAAGGTAAAAAACTGACATGAAATCAAATACGTGGCAATTAAAGAATCAGAAATCCATAACTCACTGGGGGGAGGGTGGGCGAGTTGGGGAAGGTTGGGGTGGTGGGGGGAGGGGATGATGGATAAAGAGAGAAAGCCAAACGAGATAAACAGGCGAAAAGGAAAGAGAAAGACTTCAAAGGCAATTAAAAAAGTGGCAATATCCGGCACGTGACCTCAGGGGCTGAAAAGGGAGGCGAATATTTAATCCCAAGGCGATCTCTCAGACTCACACGAGGCGGATCACAGTCGCAGGGAATTCCCCGTTTTTAATTTGTAACTGCTGCGCTTCCTTCATCTCCTACCCACCACCCCCCAACTCCCAAAACATCCTTGGATGGCCTCTTACCTCCTTCCTGTATCTGTCTGTCAGCTTGTCTGTCTATGTACATGCATGCACGCACACACATAGAAAAGTAGATATAATTGTCTCTTTCTCCTTTCTCTCCAACTAGATTATATATATATATATATATATATATAGTATGTGTATATATATATATATATTTATTAGAAAAGTAGATATAAATATCTCTTTCTTTCCTTTCTAACTCAACAACTATATATATATATATATATATATATATATATATATATATATATATTATATATTAACTTATAACTACATACATACGCTACTTAACTCTAGCTTGAAATGTCACCTTATAAATTTAATAATAACATATAAATATAATAATATAATAATAATAATAATAAGGAATGAGATCCTTGAGATCTTAACCGCAGACGGTTTTTCCCGCCATCTGGCCGCCCTTTGCTAAGATCATTCAAAAGTCTATGAAGCGTTAATTTCTTCATATAGGCTGGGCTTTACGGTCTTTTCATTCTAGCTCGACTTTGAAGAAAATTTATTTTAAATTAATTCTCGTGGTTGGTTTTTCTTTCTAGAGCAATGCTCATAATGAAAAGTTGTCACTATGTGAAAGAAGAAAGTATATGGAACTACTGATTCGAGCCTGCGCATTTTCAAATGACCGTATTTGCCACTTTCTAAAACTGTTATGTAGATACATGAAAATACTGATGTCACAACCAGACTGTAACTGGGACACGTCAAACAAATGATCCATTTCCTTATAGAAATGCTTGTGATGCAGTATGAAACGTCCAAATTAAGTTTCGAGCCTCGGCAACCGCAACAAAGAACCAATTTTTCAAACAAACCCGAGACACACTTGTCCGAACATCCGTGGAATTTACCTTTGAAGTGTCGTTCAATTTGCAGCAATTGAGCTGGATTACAAGTATCTCCTGTCAGTCCGGTATCACCCATCGTATCAGTGACAAAGAGGGAGAGATAAACATGGCAAAGACAGCGCCTGGCATTGTCAGGAGGGAACCATTAGAATGATATTAGGAGGGATGGTCGCTTCCCTCTAATATGGCGGTCTATTGTTCCGTTTTCTGCCCAACCGCCGGCTTTTCCGCTTTGTCCCGATGCGTGGCCTGCTTCTGACTTTGACTATGATGATTCGTAAATGATGATTCGTAAAATCAAATATATGGAAATAAGCTGTGAGAATTTTTTTCTAAAATATACTAAAAATCAAATATATGCAAATAAGCGAAAATGTAGATATCAATCACTGAATGGTGACAGGATCATTCTGAGCAATATCTCTTAGTAGTATCACTAACAAAAGTATAATTATGTACTATACTCATCGGCTTCTTGGCGAAAAGGGCAGGGCATATAAAGGTAGACAAACAACCAAAATTTAACAACAATAAAACAACACATTTATTCTGACAAAAAAATTTCCAGAAGTCAACAATTTGGTCAAACAGCCCGTGCCTCCACAAGGGAAGTTCATCACTGACGCATTAAGCCATGAACGAGTGGCACTTCATAGGGTACTTTCCTGGACCAGTGAAAAACACTAACGCGCCTCAGTAGCGATAAAGAAGGCAAAGATCAATCCTGGAACGGCTCAACCGCCGTGACAGATTAAAATTGACAAGTGGTTGTTTCTGCACATGACTGAAGGAAGTACGTTTGATATAATCAAAGTTAAACATTATTTTTCATGAGCAATATCTCTTAGTAGTAAAAACTCACAAAGAAAAAATAACAAAAAAAGTTGGATAATTATGTACTAGTCATCAACCTTTACACTTTGAAAATTTGTATGCATTTTGCATGTGATACGTGACTCAAAAAATATTCGATATATAAAATGTTCGTGCGGAAGTCAGTACATCAAATATAGACATTTTATATTTAATGGATATTTTCCGAAGGAGTGTCGAGCGAGGCAATAGAGAATTCTTAGAAGAAGCACGGATTAAAGGGGTAAATGGGAATTCTTTCAGTGGCTAGGTATAGTCCACAATCTCATGTGATACACTCAAAAAAAAAAATATTCGACTAAAAATGTTCGGCCATTTTTTATATTTACTTTTGAAAGAAACCATTTCAGTGGCAGCAATCTCCAAGTTTTCAGCAGCAACAGCAACTTCAGCAATGCCTCAAGTTCATCTTCAAAATCACAGGATACACGGATGGAAGAGAGAGAGAGACTGTAGAATAGGAAAATACGGAGCATTATTAGACTGCAAGAAATACATAGAGGAAAAAGGAGGAAGGGTATATCATCAGAAATCCACCAAGTGGGAGCATCAAAATATCGTTAACTACCGTAGAAAGTAATTCCCATTTTCTCACACGGCCTTTCCATCCTGCTAATGGGATGGTCACCCTGTTACTGCTCTCCCACTCCTGGAGTCTCTAGCCTATCCATGGCCCCCCACCGCTCATTGTTCTCATGTAAAAAAAATGAACAGAAAGTATCTGCAGGTTGGCTGAGAAACGATAAATCAGAATGGAACTAATGTGTCATTTGAAAAGAAGAATTTTGGGATGATGATTAAAATACAGCTGTGGCGTAAAAGTTTTGTTAGAGTAGAAATACCAATTTAGTGAACCAGGACCCACATCATCCCGCCAGAAAAGAAAAAATGAAAACAAACGAAATAAATAATACGCGCAGAGCAGCTCACAGCGCCATTTTACCAAAGGCTTCATAAGTCGATTATGAACTTTCTGACGAAGGCAGCGGTGTCTTCGCTACAAAGATTAATGAAGGAAACGCTGATAACTGATAAGCCATGATCGATGTGGTAAAAAAAGAAGTGAGCTTTGGCTTATTAGAAAAAAAAATTCCATTTTCTCAGTGACACTTCACTTTCCAATCATAGCTTCGACTTTCTGAAACCCGGAACGTCAAAGAAAGTTCATACTAATGTTTCGGGCTTTATGTTACTGGCTATTATTCCTAATCCATATTTATATCTCGTATGGGCGTTTGCAAATCAGACTCCAAGAATCATTTGTTTTTAGTGTCAAGTCTTTTGAATTTTTGAATGCTGTTATTTTGTTTGATTTCCACCTTTGTTACCATGATTCTAATCCCGTAGAGGGGTAGTGCCGTCAGTGCGATCACTTGGTGCACTGTTAGCATTACTAAAGGGTTTTCGCGGCGTCCTTTCATCCCCTAGCTTGCAACTTCTTTTTAGCCTTTTACTTTACCTCCATTCCCGCTTCCTGTCTTCATTCTTGCTGTCCAACCTCTCTAACGTTTAACTTCCTAGTGTAAGTGTGGGGGTTTCTATCAGTTCCCCTTTTATATCCTTGTATGCCCATCTCTTTTATTTTGTGGACTTCTTTTACATACTGTTTCCCCGTGGGGACCTCACACGGTGCACTGTATGCATTGCCTAAGGATCTTTGTAGCGTCCCTTCGGCTGCTAGCTGCAATCCCTTTCATTGCTTTTACTGTACCCCCGTCCATAATCACTTTCTTCCATCTTTCTTTCCACCCTCTTCCAACAATAGTTTTTTAGTGCAACCACGAGGTTTTCCTCCTGTTACACCTTTCAAACATCTTCTGCTCTAAATTTTCATTTTCGACGCTGAATGACCTCCACAGGCCCCAGCGCTTGGTCTTGGCCTAAATTCTATATTTCATTCTATTCTATTTACCTTACTATTACACAATTCCAGCTCCCTGTCTTTATCGTTTTCAGAACTTATTGGCCGAAAGTAACCCAGTGCTCGAGCTGCCAGCGCAAATTTTATAGATCAATCATAAATCAGCCATGGTTCCTCAACGACACCTCTTTTCGTGTAAGATCAGCTTTCCCAAGTAAGACCACTTAATCTAAATATCCCGCATACTGGGATTTGAGAAATGTCGAAGGTTTCCCAACAGGATATCGCGTACCGGCATAAATGATCTTGCAATCCCAAGGAAGGTAGCGAGGAAATTGAAACGTTCCCATATTCTGTTATGTACGCTATTTTCTTTCTAAAATGTCTTCCCCAACTCTCTCTCTCTCTCTCTCTTAATCAGATTTATTTAGCCACTTGTTATGACTATGATAGGTTTACAAAAATAGCATCAAAGATCTAAATCATGCCAAGCTATGTTTCCAAAACTCTTAAGTAAATTAATTAATTAAATAAAACGGTCGTTGCTCATTTGTAACACTGAATTTATGGTTTGGACCAAACACCATTTGAAAGCTTTCATTATACTGCACTGACTAACATAATATAATTTAAAGGTACTGATGACACTTTCTTTAGTAACAAAAATATTTTTGTCTTATTTTCTGTATTTATTTCTTGAGTATTGGAGTGATTACTAATTCAAGTTTTCCGTACAAGGTCTATTTTTTAAATTCTCCTTTCTTGTAATAGTAATCTTTGAGTTGAATTTTCCTTCATTCCGATTTTTCGCTTGGCTGTTTATTCTATAAAATAATTGTGGAATGCATTTGCATTTATTGTTGATTCATTGAACCTCCTGTTTTACATTTTGTCCTTGGTCTAATAATAGCTGATGAAGTAACTGTAGTTATTCTTTTCCCCTTTTTTAAATATATTAAATATATATATATATATATATATATATATATATATATATATATATATATATATATATATATATATATATATATATATGTATATATGTGTGTGTGTGTGTGTTTTTTTTCATTTTCTTCTCATCCAGTGATTCCCCTTTCCCCCCGCCTTTTTTTCTTCGTCTCCCTTCTCAATCCTGATAAAGCCATAAACTCCACAATGACTTCCTTCTTACCCCACTCGCGCGCACGCACGCGCAAATACACCCAAACGTTCACACACAAAACTGCTATAAACCGCGAAGACAGGACAGTGCTAGGCTCTCTATCCAGTAGATTCTAAATATAATATTCTCTCTCCCGCAAATTTCGACCTTATGGCCTCCGCAGCAAAGCTCGTCCTTTCGGCATCACAGACCTTCATGAGACAATAGTAACTGCCATTTAAGCCATAAGGGCGAGGTGCCCTTGACCCAACTGCGGATTATTCGACCTAATGGATCCCGGCGGGCGTCATCCTCGCCTCCCGAACTATTAAATTGAGTCATCAAGACGTTCCGCGTGTTTTTGACAGCTACTTCACTACAATAGCGCATGCGCAGTAGCCCACACTCGTGCACGCATGCGCGTTGAGTCTGTAATGCATATGTGAATTTTGTCACACCAAATCGACTTTGATGAAGATGTATGTGCACACGTCTGATATTACTTATTACTTCTGTAGTAAGTTTTAGATTTAGAAAAATAAAAGATAAATAAATAACGAAAACTTTCCACGTCTCCTGTTGTTGATTTAGTTGTGGATTAAGCTGGCCTTATGCCAACACGGGCTCCACCTCCTGGAGCGGAACGGAAATCCTTCAATTAAACACATATCACACAGAAGAAGTAGAAAGAACGTACATGACAGGAGATGTAATATCGTCTCTTTATAGTCCTCAGATTACCGCCAAACTAATTGTATTCTATGGAAAACATGATAAAAATATAGATAAACAGTTTATCATTTTCTCTCAAAAATTAGAACCAGAGAGCAGAATAAAATTCAAAACCAGGGGCAGAAGTATATCTGAAAAATTGCTATCAAGAAAGTGGAGGGAAAAAATGCTGATAAAGCACGAAAAGTAACTAGTAGTTCATCCATGTGTTCGGTTGCTCTGCTTTTTGACAAAATTTCTTTTAGGGGTGTTCTGTATTTTCTTCATTCAGTGGGATATTTTGCCCATATATATATATATATATATATATATATATATATATATATATATATATATATATATATATATATATATATACATACATATATATATATATATATATATATATATATATATGTCTGTATATATGTATAGTGTATAAATAGATTGATAAATATATATGTATATATACATATATACATATATATATATATATATATATATATATATATATATATATATATATACATACACATATACATAAAACAGGTATTTATACCTGACAGTAGACCAGATATCTGTCATACGCAGCTACCTGAGTTGTAAATGGTTCTTTTGTTCCAATATATTCCGAGAAATTCATCTCCAGAGGATAAAAACCGCAGAAAAAACTAACGCGACACATACTGATACGATCGATAAAATTAGGGTATTTTTTCGGCGATCAAAATAACGTTGACAAATTACTATTGAGTACGAATCAGTCTGAAGATATAAACAAACATCAACAAAAGTCAAAGCTCACGCAAAAATATGGTACTGGTTATCAGGTTTATGACCACTAAATCATAACCCTTAACAAGTTTTATCCGACGGCAGATAAGGTTTGCATTTTTACTGGCCACTTGTGTTATGTACGGCATGGTAGCAGGATGAAGCAATCATTAAATAAAACAGAAAGGATGGCAAAAAGCCGGTAGTTTAAATGACCAACAAAACCAAACTACCTCACATGTTAACACTGAGAGAAACTTTTGACATCAACTTGACGAAATCTCGCTTCTTAAAATCATGACCACAGCCTCCGACAAGATAAGAGTAAAAGAAAAAGAAAAGAAAATAAAAAGAATAACGCAGTTTGTGAAATGACTGAAGAAGACATTTAAATTGATGAGGAACTTCCTGTGGAGAAAAAAAAAAGACTAGGCAAAAAGGGTCAAAATAAAAAGTACTTCATTTCAAAACGGATTTTCGAGCTCTCTCTCTCTCTCTCTCTCTCTCTCTCTCTCTCTCTCTCTCTCTCTCTCTCTCTCTCTCTCTGTGGGTTCCCTAACAACACATGACCGAGATGACGTGTTATGAGGTCACGCCGAGTTGACCTAATCCGTTCCGGTTAATCCCTCGGCCCGTCTGAGTATTATTCATCCTCCCCCCATCGATCGAGGCGCTAAAGGGAGTGCAGAAATAAAGGAGGGGTGACTTCCGTCAGAAGGAGAAAGGGTAAATGGACACATATATATATGTGTATATATATATATATATATATATATATATATATATATATATATATATATATATATATATATATATGAACTTCTCAGTCCCAGAGGTCCTTCATTCCTCACACCGTTGGACTGTGGTACAGCCTCCCAGAGGATGTCGTGCAGTTGGAACTTCAGAAGTTTTTCCTAATAATTGATATCTTTCTGTATTTCCCATTACCTTCTGTTACTTCTTTCAATTTAAGTCAGTGGCCCCTTTATCTTGTTCCATATACATATACATATATATCTATATAATATATATACCTTATATATGCATATATATATATATATATGCATAGATATATATGCATATATATTGTATATATATATATATATATATATATATATATATATATATATATATATACACATACATATATAGTGTCCAAAAGACAAAGTTGTAATCTCAATACAAGTTTCTGAAGGACACAGACTTCTTGAATTGGATTCAGAAAGACAAAGGTTTGAATAAGATGCAATCTAAATTAGGTAGGCTAGAGAGAGGCATGTTTGAAAAGGGCCACAGGTCTGTACAAAAAGGGATGGACCAATGACACCAAATGATCCGAATCTGTGATGAAGAACATGGCGATAATTTTCATCTTGTCAAGGTTGGGAGTATCGGAACGTCATTGCTAATGAAAAACTTATCTGTAGTTTTCTCTCTCTCTCTCTCTCTCTCTCTCTCTCTCTCTCTCTCTCTCTCTCTCTCTCTCTCTCTCTCTCTCTCTCTCGTTAACCTTAATTTCAATCTTTAGTTTTGATAGAATTCGGTATCATCTCCAGTGCTATAAAGGTGTTAAGTATTGTTCGCAAGGCCTCTTCAGATCGTCTGTAAAATTTCTCGGAATATTTCAGAAACTTGCTTTTTAGGGTAAATTGCTAGTAGTGATGGTATTTGTTTATTTTCACGAAACAGTACGGTAATCAGGATTTAGACCGTTGCTGGTAAACATTTCAAGCTCTTTTTGTACCAGTAATGCATATATATATATATATATATATATATATATATATATATATATATATATATATATATATATATATATATTGTATATATATATATATGTGTGTGTGTGTGTGTGTGTGTGTGTGTATGTATGTATATTGTATGTATATGTACATATGAATATGTATATATATATATATATAAATGTCTGTATATATGCATAGCCTACATATATATATATATATATATATATATATATATATATATATATATATATATATATATATATATATATATATATATATATATATAATGTATAGACATACATATTATGTATCTATATATATGAACACCAAAAATGCACACACGTTTATTTGTGCTTAGGACGTCGTACGTACTGATCCACCCGACGGACGGACGCCGCAAATGACGAGACAAACAACCCAGCAGAGGAGAGGAGAGGAGAGCCGAGCCAAGGCAGCGCCACCAATTGCCTAAAAGCATAATGACGGCGTTTGGGCGGTAGACACATCGTAATAACAGAGTAAATTACAGGCCCTAAGTGCGGGGAATTACAAGAGGAATGCGACGATTACAGGCCGCGCGGACACCTCCCCTTCCCTCTTGTAACGGAGCTGGCACTCAAGATGACGTCGAGGGCCACTCGCTCGCACTTCAAAACTTCAAGAGCAACGGATTCAAGTGGGACTTCTGGAGCCGGGTATGGGTTGTTGGCGGCTGCTGTGACAGGAAGGGGGGAGAGGGGGGTGGCAGAAAAGGGTGACACGGTGACAGCCAAATGAAAAGATGTGAATCATGGCACAGAGAGAGAGAGAGAGAGAGAGAGAGAGAATTATGATTTAGTTCTAAACAGGTGTTAATGAAAAGATGTGAATCATGATACAAACACACACACACACACACACACACACACAGAAATGATGATATATTTATAAACAGGTATTGATTAAAAGATGTGAGTCATGGCAGAGAGAGATGATGTTATTATAAACAAGTGTTAAACAGGTGCACACCTGGACAAAAGACTGCGTATTTTAAACATCAGATGGAGATAAAAGTTTCAGTGTTATTTGTGGTGACTGATTATGACTGAAAGAAAATTAGAGCTGTTTACAGTTCGTGTAAGGTCTGTGAGTTGAAATTTTTTCTGTCAGTTTTCCACCTTTCGGGTGAAAAAAGTCACTATAGGTTTTGTAAGAAGGCGCAGGTAAAGTCACGACGGGTAGTAAGAATTCATTCCTGGAATCGGTTATGATACGTGTTACAATATAAAGAGGATATTATCAATATTCATATTTCATTTGAAATTACAGAGAGAGAGAGAGAGAGAGAGAGAGAGAGTTTTCGTATACCAGAGTCGAAAAAGTAAGAAAAAAAAAAATTAAGAACAGACACAAAACTGACCAGTCAACGAAATAAACAGATATTAACAAAATACGAAAAAGTATTAATATATACAATATATATATATATATATATATATATATATATATATATATATATATATATATATATATATGTGTGTGTGTGTGTGTGTGTGTGTGTGTGTGTGTGTACACTTTACATTCAAGGACGAGAAATTGCGCAAAGAACGTAAAAGAAAAATATGAAAAATCACATTATAAAATCGGTGTCCATAGCGTGTGACGTCGAAGCGTTCTCTGTGAGGGGAGCCTATTGTTATGCTAATATTCGTCTCTGTATTGGGGATTAAAAGCGGATTAGCGCGGGCGAGCGATTAATTGCCGTTGATACATAATATCTTAGAACTAATCATATTCCTTTATTAGGTAAAGAGCTCTTTTCATGGAGTAAACTTATTAAAAGAAATTGAAAAAATTCTCATATACTGTATAAATATATATATATATATATATATATTTGTATATATATATATATATATATATATATATATGTATGTATATATATATATATATATATATATATATATATATATATATTTATATACATATGTAAATATATGTATATTCATTTATATATAGGTCTATATATATGCATACATATACACACGCACATATATATATATATATATATATATATATATATATATATATATATATATATATATATATATATATATATATATAGGTTATATGTATGTGCGTGTGTGTACAACAAAAAGGAACGTATTTGGTCAGCCATTGATCTCAAACAGACACCAGAATTCCTTTTATTCCAGAGATTAGATTTAGTATGGATACGACCCTCTTAGAGGGAGAGAGAGAGAGAGAGAGAAAAAAAGAAAAAGGAAATCGAGGTAGGAGAGGAGTGAGAATCAGCATCAGTCTCATCTTCAAAGTCTTTCTCATCATTTTCATCTCTCGCAGGGGTCAACAGTTTCCGGCAAATATTTTCTTATTTGGATGTTTAAAACGGGGTTTCGGAACGATGAGGAGGGGGAAGGAGGGGTTTAGGTATTTGGGTGGGGTGGGGTGGGGGTAAGGGATGGCTGAAGGAGGGGAGGGAGGGGGGGAGGAGTGATTTTTAGTGCTACGCTAAGGCTCTTAATCCCACTTCCTGCAATGGAGCCGAGAACCATATGGTATCTCAAGGCCAGGTGGTGGGTGGGAGGTGGTAGGGCTGGGTTGCTAGTTGGGGGAGAGAGGGGAGTGAGTTCTTCAAAGAAATGTTTACTCCTCAGGAAAGCATGATATTAAATTTTCAAAAATCTTTTGATGATGTTAAGAAATCCGAGGGACTCCGGGAGACTTAAAATCCTCGTTCCTTATCTCTATCTATCTATCTATCTATCTATCTATCTATCTATCTATTTTGACATTAGTTGAGATGGTAGATTAAGAATATTATCTAAGTCTCATGTAAATTCTAGGAAGACAGGGTAATTGAAAAGTCGATGAAATTTATATATATTCTATATGCAACTTGAATATCAAATTAAACAAAACTGAATTTAACTATTCACTTATCATTTAACATGTCTTTCGCTCACTTCGAATAAGAGTCGTCTGTTATTTTTATTAGTGGTCTTCTTTGTAACCTACTTTATTGTAAGAGAAGGAAACAAAGCAAAGCCAGTGATAGGAAAACTGGAGGAGCGTGGAATAACTGAGAGGGGACAAAAGATAGCTTGTAAATGTTTCTTCTAAAGCATGACTTGTCCAACAAACTCAGATTGAAAATCAGTAAATTAATAGATTATAAACACACATGCGGTGGCACGCACACAACAACACAACCACAAACACTAACATTTATATATATATATATATATATATATATATATATATATATATATATATATATAAATCTCGATTAACAACAGCTTTTCCTAATAGGTGGGGCTTGAAAATTTAATAAAACAACAGCAAATTACATCTATTCAGCCGAAACATGAATGGACCCACTTTGTCGTGACCTTCAGTTGCATTAATAACTGCATATACCCAGAACAAGAAAAAGGCTCATTGCCAGTACATTGACTCACAAAATGAGAACATATATATATATATATATATATATATATATATATATATATATATATATATATATATATATATATACATATATATATATATATATATATATATATATATATATATATATATAATATTTTATATATATATATTCCATCTATATTTATGTTTTATATATATGTTCTTATATGATCTTATCAGAAGACCAAAGGTATTGATTTCAAATTGCTTGAAGTTCAGATAACTTAGTTACGCTTCCAAATTCAGCACGAACACCAGAATAATAAAAACATTCAAACATTCATTCATAGGAAACTTTGAAATAAATTGAAATACATAAAGACAAATTACTGTAATGCACAGTAACTCGAAAATGTGATACCTCCTGTAAGTTTTTCTTTTCATTCTACTTATCATACTAATTGAAGATTAACAAACTTTTACAGACAGCTGCAGTTCAAACAAAATACAGCAAAAGGAACGTAAACATATTCGAAGCCAGGCAGCGGTTCTGGGAGTAGAAAAAAAGAAAGGTATTATGACCAATGTATCAGAAAGGATTACAGGTATTAACGGTTTATAAGAACAACACGCGTTCGCTGTCGTATATCGCAGCGCTTCGAAACAGGAATTTACTCGTAAAGAATTTCCGAAAGGATATTTTCACATATTCAGCTTTATGAGAGAATATTCCTGCATGTCTGTCTATTTATTTTCTTCTTTTCACGAATGGGCAAATATGTGTACATAGAAATATAAATATATATATATATATATATATATATATATATATATATATATATATATATGTATATATATATATATATATATATATATATATATATATATATATATATATATATATATATATATATATATAATATATATATATATATATATGTCTGTGTGTGTGTATAAATAATATAAAATATATATATATATATATATATATATATATATATATATATATGTATATATATATATATATATTTATACATATACATATAAATATATATGTATGCATATATATATATATATATATATATGTATATATATACATACATACATACATACATATATATACATACAGCCAAAACGCCGACAATCAGCTTCTGATTACAACGGCAACTGAAGAGCCACGGGAATTTTGAATCTAAATTGCCATAGTCCGCCGAAAATCAAGAAAGAAGGAAATAAAAAAAAGAGGCATGGTCAATAGGCCTGGTAAGACTCTGGAATTACCTCAGCTTAAAAACTAATATCTAGAGCTTTCGCACACGCGTCACTCTTCTTCGTTTGCCTCCCATTGGCTTCGGCTACGGTTTCGTTTAATCGCGAGAGGACGAGTATTTTGAATCTGGAGAAATTAGAGTTTTCGGCAAATCCCTGCTCTTGTTTTTAAATTCATAGTAGCAGTAGCAGTTAATAGTCAAAAACACCATTATTTCTCGTATCACCTGTCAATCGCAATAATAACCCAATACTTAGTATAAGGATAATGCGAAAAGTAAATAACATTTTCTTTTCTCACTAATAGTGTACATCGCCAGAACCGCGTAACGTGTCGAATGTAATCAATAAGAACATCAGGACTCATGCTAGAACCCATTCCCGATGCTGTCAATCAAATCTCTTAATGAATAACAACAGGAGAGGAAAGTAGTTTGTGTAGCTTTTCCTATTTATGAATAAGAAAAAGTCACGTGTGTATATATTATTATAAAAATATATATATATATATATATATATATATATATATATATATATATATATATATATATATATATATATATATACAAATAATCAACACACAATCACGTGTGGAACAGAAATAAATTTTTGACTCACGTCGGGATCGAACCCAGGTTTCTATGGAATATATATATACATACACATATTATATATATATATATACAGTATATATATATATATATATATATATATATAGGCGCTATATATAAAACTATATATAATAGATGATAAACTCTCTGGATGACAGTCGATAAACTCTCTCTCTCTCTCTCTCTCTCTCTCTCTCTCTCTCTCTCTCTCTCTCACATACTCTTTCCTCTTTCTCCTCCCTAACACCCCCTCTACTCTCCCTCTCACTTTCTCTCATTTTCTCTCTCTCCCCTTTCCTGTTAAGATAGTTGCTTCAGTTAATTTGCCCAACATTTTTGACATTTTATATTTCACCCCTTCTCACCCCCATTCCTATCAGGGCAGAACTTGGACTTAAAGGGCATCGGGAGTGTGACTATTCATCACAGCAACCTCGAAAACTATGGATTAGACACTAATAGATGATGTTTTCGGTTATTTTTACATGTCACCCCTTCTCATCCAGACCTTTGGTGCCAGTGATGTCTTACCCCCACCCTACAGTATTCTTTTGCAGATTGTAAGTCATGTGTATACAGAAATAAGGTATGATAAACCCGTAGAGTTTTTTTAGTTACTAAGTCTACAGGCAGAGCCAACCCCGTTTCAGGGAAGCCCTCCCACCCCTACCCCCTTTGGTGCTAGTGATGTTTAACCGGCACATTATTCTGTTCCACATAGTAAGTCATATGTGTAACCAAATTTGGTTGAAATTGCTCAATACATTCCAGAGTTATGCTGGAACATACACACACACACATACATACATACACATTTATATATATATATATATATTTATATATATATATATATATATATATATATATATATATATATATATATATATATATATATATATGTATATATACAGAGGGGTGGCTGCAGTAAGCACCACCCTTTCAGTTTTTAACAGGTATTTTGGGATTGGGGCGCTAGTCCCTCACCCTACCCGAAGGCAACTGGTATTTCTTGCCGATCACCCATCTAAGTACTGACTAGACCCAGCGGTGCTTAACTTTGCTGACCTGGCGACCAGCGTTTTATATATACAGTATATATGTATGTATATATATATATTTATACATTTTAATATATGTACAGTTTATATATATATATATATAGAATGAGAGAGAGAGAGAGTGAGAGAGAGACAGAGAGAGAGAGAGAGTTTGTGAGTACCTTCTATAATCTGCGAGTTCTAGAGTAAGTTTTTCATTAATAATACAATATTTCCATAACTGCACATGCGCTGACTTGTTCAGATACTAATTAGGAATTTACGTTATAAAAAAGAACGATGGAAATATGTACGGTATGAAGACTAAGTCACATTAGACTATGCATATAAATTTGCATTTGTTTCACAAAATGTGGAAATCTAGAAGAAATTTGTAGAAAGAACAGTTTCATTGTGTCTGTATGTAAATGTCTTCGCACATGCGCGGTATGTGTTTATGTGTGTATTCTTAATCATCCCTCAAATTATAATAATTTGCGGCGTCTTTTAGAAGTTCCGCGAAGTTACTGAAACAATTGTGAGATTCACATTAAATGATAAATATGGTACTAACAATAATGCTTTACCTTTCTATATATTAGTTTCTCATTTTATCGTTTCTTTCGTTCATGGAACTCTGGAGATTAAAAACAAAATAGTAACACAGGTAGAGACCAGTTTAATTAATAATAATAATAATAATAATAATAATATTATTAATAATAATTATTAATATTAATAATAAACCAAACAAAAACTTCTTAGTTTTTTTATTAAAGAAAAACACATTAACTTGTTTACTTGTAAATATTATTACTTTTAAACTGATAAACCATTAAAAAACCTGTAAATGGGTTGTCAAGATCAACGTGTTTCTGAGAAAATTATTATTATTATTATTAAATTATTATTATTATTATTATATTGATTAACATTATTATTACTACTCCATTGTCAGTTAAACCTGTGGGTTGCCGATCAGTTTCTTGAAAATCAATAAGAATATCAGATTACTTGATTAACAATTCACATCACAGCGGTCTAGTTGCTTGGAAATGAAGAGAGAGAGAGAGAGAGAGAGAAATTATTATCATAAGTAACTTATAAGTAACTAACCTGACATAAAGTACGACGGTTATCAGTACTACAGTATTTTGTTGTTATGAATTATGGCTCTGCTGTCATTTACAAAAAGTCCCACCAGTTGTGGTTTGCTATGAGCGGAGATGGGATAGCAAGTGTTCGCTCTCTGAACGCAAATAGGAAAAGAATTTTACACAGTTGCAAACAAGTGTGTGTGTGTGTGTTAGAGAGAGAGAGAGAGAGAGAGAGAGAGACAGGCGGCGAGGAAGCCATTGATAGTGGCTGTACGTGGAACCCAATAAAAATTGTCGATGTAGAAAAAAGATATCTAACACAGATCAAAGATTTCCCTCGGGGACTCTCCCGAAGTCACAACACTCCTCCCCCCAAAACCCCTACCTCCCCTTAAATCCTCCGCCCTTCCTTACTAACCCCCCAAACCCACCCACACCCAGCCAGCATCCAAGACCAATCTTCAACCAACCACCCCTCCTTACCCTTATTCCCCTTCTCCCCCACCCCCACCGATTGAAGGTAGGGGTTAGGCCACCCGCCCCTTCCTTGCCCTGCCGTTCCCATGGACTATGATACAGATCGGTTTTATTAAAAACGGCGAACCCCGAGTCCTGCTACGCCAGCTTTTAGACAATTTTTAATTTGGTCGCTCGGGATGCTTCAAGAGGTAGGAGCTGGGGAGGAGGGAAGATCGAGAGAAGGAAGAAAAAATATATTCTGGGTAAAAGCTGGAAGAATTTATTGTGGAGGACAGGGATAAGAAGGACGCGATCCAAGAATAAAACAGAAAATTTGTTATGAGAGAGAGAGAGAGAGAGAGAGAGAGAGAGAGAGAGAGAGAGAGAGAGAGAGAGTAATGACTGAATGGCCCATGCAACATTGTTGGATCTTAGATGGCAAAAAAGTTAGAACTAAGTTAAATTTTACGACAAATTAACTGCAAAGGTAAGTAAAACTTTAAATATAACTTCAATAACTGTAAACCACAAAAATATAAAGTAACAACAGTCCCAATCATCCTGTTTCATAGGTAACCTAAAGAAAAAAATGATAAACACTACAGTATTGAGTGACAAATGTATAAATGATAATAAGAGAGCCCAACAATAATGTAGGTTGACAGATACAGACATAAATGTATTAACTGGCAGGTCAAATGAAGCTTTTTGTTAGATATGGAAACGGATAGGAGGTAACTAGATAACAGACATACCAGAAATCTAAAAAGACAAAAACCTTATTATTATTATTATTATTATTATTATTATTATTATTATTATTATTATTATTATTATTATTATTATTATTATTATTATTATTATTATTTGTATTATGATTATTATTGAATGTATGGCTATATTTTTAAAAGTAAAAAATGTTCCCTTTGAGAACGAAAAGGGAATCGCAGATGGCGAAAGATTCATGCTATCACTGAAGAAGCGGAAAAATCGGAAAAAGGTTTATATTTAAATATGTACGCGATATTTGTACAGTATGCTACGATTATTATTATTATTATTATTATTATTATTATTATTAATTATTATTATTATTATTATTATTATTATTATTATTATTATTATTTGTAGAGTGCATCGGACTCGCGATAACAGTAACGATGTTTCGTAATAAAAAAAAAACACGGACTCGACAAGCAAACATACATGCTAGGGTCTGTTTGGATTCTCGAGTCCACTGATACAAACCCGGAGGTTGTTTACATGTTTATTACCGAGAGTTAAGTAAAATCTAAGACATACTTGTGAATATTGCATATCATTTGCTTTTAATCACTCCTTAATTTATGGTCAAGTTAAACTGCAGCTAGTAAAACACATACACACACACACACACACACACACACATATATATATATATATATATATATATATATATATATATATATATATATATATATATATATATATAGAGAGAGAGAGAGAGAGAGAGAGAGAGAGAGAGAGAGAGAGAGAGAGAGAGGATAAGAATAAAAGTCTCTTAATTATGCTCTTTTGTAAATACTGTCAAGGCTGTGGCTTGATGGATTAGGCTTCCGTTCTCTCCTTTCTGAGTAGAAAATAAAATCAAATTTATGCTCATACATACATATACATATATGTGTATATATATAATACATGTATATATGTGTATACACACACATATATATATATATATATATATATATATATATATATATATATATATATTCTTAGAGAACAGACATTGTTTTACAGTGTCATAAATCTACAGAAAAAGACTCATGACCTAACAGACCTCTGAATAAAAGATGGAGAGATGCTCTCTGGCGCATCTCTCCATCTTTTATTCAGAGGTCTGTTAGGTCATGAGTCTTTTTCTATAGATTTATGACACTGTAAAATAATGTCTGTTCTCTAAAGAATGTGTATATATATATATATATATATATATATATATATATATATATATATATATATATGTATATATGTGTGTGTGTATGTATACACACACACACAAATGCGTGAATGCATTTCTCGGTTGATGAAGTGAGTATTTTGAAGATCTGCTAAACGAGGCAGAGTTGAATGATAAAAGTGGGAAAAGTTAATATCACTTTGAGAATGCCTATGCTTTCGTAGACTTATGAAGGGAAATCGAAATGTTGAAGAATGGAAAGACACTATGAGCTGATGGAATTACAAGTAAGATGCTGAGCTAGAACGGTTTCAGTGTGACAAAGTTGCTGACCAGAGTTTGTAAGGTAAGCATGAAGTATGGAAAGGTCCAGAATATGTGGGTTGTGGAATAATTGCTCGGCTGAATAAAGATAAAGGTGATTGTGGTAATTGCAATAGTTTGGAGGCATAAAACTGCTTAGTACAACAGGACTGGCTTTTGACTTTCTTGATATAGAAAGTTAGACAGATGACAGAAGGACTCACAGGGAAAGCAAAATGTGGATCCAAGCAAGGAACAGGTTGTGGGGATCGAGTGTTTGGTATGAAACATATGGGTGAGATGTTTGAAATTAAGGGAATAATGGGCATGTGTATTATGTGGATTTAGGAAAAGCTTATGGTACAATCGACAAAGAAGTATTCTGGAGGTAATTCAGACATAGGTGATGAATTGTTGAGAACGATTAAAAGTTTTTGTGATGCAATTAAGAATACATAAGAATATGAAAGACAGGACTGTTTTGGGGTAAAAGAAAAAAAAAATTGGTTTGGGAAAAGTTGCTTTATTTTTCATGTATGTTTACGGATGGAATGCTGTGAGGAGCTAGCAAAAGGACACTATAAGAAATTGCAAAGTAGTGGGATAAGAGTTGTGAATGGAGTGTGGACTGGTTATAACCTGCAGATGATACAATACTGGTCAAGGATAGTGAAAAGAAACTGCAAAAAACCTAGTGTATGCAAGGAGAGAAGGTTGAGAATAAATATGAGCAAGACTACAGTTAAAAGTGACATTGGAAACCGGGAAGATGGAGTCACGAATGTTGGTATGTATGGTGGAAGAATGAAAATGTCTGATTCGTACTGGTATCTAGGAGTAAATGTAATGGATGATGATAAGATGAGGAAAGAGGTAAGTCACAGAATGGGCGGAAGAAGAAAGTTAACAGGGTATAGTCACAGACTGGAAGACACTTGGAGTATATGAAGAGGTTGTTGAGCCAGTTCTCCTTTGTGGGAGTGAAGTTTGAATGTTGAATGCGAATAAAACAAATAAGGATGATGCTGTTGAAGCACAAGAGTTTTTGCAAAGATAAAGAAGGAAAGTGCAATGTGTGTTTAGGGTGATAGAAGTGCTATTGATGAGCCACAGTGTTATGGAAGTGTTCTTCTCGGGGACTTCATTCAAAATAGAACAGGAAAAGTATGAATGTGGCTGTTACTGTTTTTTATTATTTACTTTTAGGGGCCAACTTCTGTACACGGACAACACACATATACATACAGACATACATACATACAGACACACACAAATATACATATATATATGTACATGTATGTATATATACATACATATATGTGTGTGTATGTTTGTGTGTGCGAGCGTGTGTGTGTGTGTGTATGTATGTATGTATGTATGTATGTATGTATGCATGTCTGTATGTATATACGTATCCCGTGTATATAAAGTAGCTTCTAAAGAAAGTATATATATATATATATATATATATATATATATATATATATATATATATATATATATATATATATATATATATATATATATTCCTATAATATATATATATAATATATAAGTATATTTATATATATATATATTTCATATATATATATATATATATATATATATATATATATATATATATATATATATATATATATATATATATATATGTGTGGGCCACACTGCTATGTTTTTGTATATATACCATTGTGTGTGCACCTGTCCATGTATGTATGTAGCTGATGCCCGAAATATGTATGTCTAAATATGTATATGTGTTCTTATATTTCATACATATACATGAAGTGGCCTAAAAAAGTTTATATACATATTTATATATATATGTATATATGTCTGCATATATATGTGGTATCCGTATATATATATATATATATATATATATATATATATATATATATATATATATATATATATATATATAAGTACATATACATATACAAAGATACTTTGGACCCTTTCACCAGACAGTAAAGGTACGCGCAACGCGATTAAGGATTCGACTCACAGCGTTCAAATTACTGTGAACAGGTGCAAACGTCTGATGATTCACTATGAGTACCAGGTATAAAGTAGTGGGTAAATTAGGCAAAACAATTTTTCTTTCTCACTGACTAATAAGTCTAGTTTCTGTATTCAATGTTGAATCTACCGAGGGGCATACTAGTAATTCAGTTAACTGAGTAGTAATCAAGATATACTTAAGGTATGTGGTACCTTTTTCGTTCACCATAATTATACATTCAAAATAAAACCTGCTTCTAAGTTAAAAAAAAAAAAACACTAAAACTTCTTGCCAATACAAACTTTGCACCAGTTTCCAAGTGTCTAACAACAGTCAATTTTACCGATTCCAAGAACATTTCCCCATCATCATATACACTAAGAAAAAGATAGTACGTCCTTTCAGAGCTAGCCTGTCATGCATCGAAGGAAAAACTACTTAGTGATAAAATCGGCACTTGTGACTAGATGATCTCACCGAAGTACGAGAATACTCACTCTTCTTGACGATATCTTGCTGAGCGCAGGGAGCCGACTGCGGGGAATCCACGGGCAAGGAGGTGATATCGTTGCCTGGTCCGCTATACCTGCAGAAGGAAGTAATCCATATTTAGGGATTTCATCGTACCGTGAAAAACAAATTTATGCTAAGAAGCTCTAGAGCAGTTTCAAATGATTTAAAAAGTTGTAAAGTCGGGATCAAGAGAGAGAGAGGTATCCATGAAGAAAAATTGACGAGAAGAGAGAGAGCAGAAGAAGAGGGGAGAGAGAGAGAGAGGTTTCCATGAAGAAAAATTGACGAGAACGAAGCAGAAGAGAGACACGAAAAAATCCGTTAAATGAAGTGATGTGAAGAGAGAGAGAGATCAAATATATTTTACCATCTTTCACGTACATTAGCTGTCAGCGTATTCTAATAAAGTTTCATATATAAGCATAAAAATAGGGGAGAAATTATATAATTCAAACTGCTTGATGTTTGGCCCAATAGCTTTTATTTACTCTATAAAAGTGAGTCAAGAAATTATTTCATTAGGTTGACACTTATCTAAACCTTCGTCGATTGTTCACGTGGGCGTTAGTTTCTCTTTATTGAAAAATCTCTCATATAATTGAAGTCGATAATTGTTTTTGTTAAGATATCCGTAAAAATAAATTACGGATTTTAAATATATCTGTCATACTATGAAGTCTCGATTTCTCGATTTCAAAATATCGTTGCAAACGGTTTCCACTGAATCTTTTGAAATTCAGAATACGAAATGCAATCAATAATTTCCCCTTTCCCTCGCCAGGGTTTAAACCAGGTCGGGAGGGGAAAAGCATAGTAAATCATTCTATCTCCGACCTACGCTCGAAGCACGGTGGGAGGAAAGAAACTCTCCAGTTCATTTCCCATTCGGGTTTTCAAAGGATTTCAGAGGAAATTATCCTCACACACATTAGCAAATCGTGAAGTTAAGAGTTCATTATACGCTAGCTGTTACATTAGTACCATTACATCTGGCAATGTGACCAGTAAATTTCATATTTTGGGAAAATTCTCAATTTTCATTCAAACTTTCCCATGCATTGTCAAATTATGTTTGGAAAATGTGAGCCTCACCTCATCAAAATATTGTCTCAAGTCCTGTGATTTATATTCTGAGATAGGCAACATTCATTCAGACTATCACGTTTTTCATGAACCTTTTTTTTATAAATAATTTTTCCTCAGAGATGCAGTTCCCCCAGATAGTGTAATACCAACAGGAGTCGTAAAGATATCCGTTTTAATAGTTACTTCTTATTCTGTCATTGCCAACTCATGTAGTACATTCTACTTGACATTTTTCTAATTTCTCTTGTTGCTTCTTTCAGGGAAAAGGTTACTTTTTTCAGCCATAGTTTAAACCCTAATTGAACTAAGAATATAATCTGAATATTCGGATATCAGAAAATGTTATTTCTAATCTCAGTTTATCAAAGAGAAACAATTTTTTTTAACTAAAAGAAACCAAGAAGAGAGAATCTCCTATTTTAAGAAATTCAGAGAAATTAATTTCAAGTCATACGGTAAAAAGACTTAATGAAATAGGAAAATAATTCAGAGAAGCTTTGATAGTTCATGAGAAAAGAAAACTCGTTTCGTTTTGCATTGCCTCAAGAAAAAGTAAAAATGTGTAATACATAAAGTCGTCTGTCTTGCTTCACAAAATTAAGACTTTACATAAAGTCGTCTAACATGCTTCAGGTATCAAAATTAAGAGCTTCTGTAATATCAAAATTAATCAAAATTAAGAGTATTCTGTAATACATAAAGTCGTCTAACATGCTTCAGGTATCAAAATAAAATTAAAATTATTTTACGATGAACGAAGACCAAGAAAGCCATTTCACTTCTGTGGCAAATACGAGAAAAACTAAATCTTATCTTCTGAAAACAATAACAAACAAGTAAAATATGTGCCGAAGTTTCTTCGGCGCAGTCGAGTTTTCTGTACAGCGTATGTTGCTGTATGAGCCGCGGCCCATGAAACTTTAATCACGGCCCGGTGGTGGACTGATCTATAACGTTGCCAGAGCACGATCATGGCTAACTTTAACCTTAAATAAAATAAAAACTATTAAGGCTAGAGGGCTGCAATTTGGTATATTTGATGTTTTAAGATCTGAGGGCGGACAGAAAAAGTGCAGACGGACAGACAAAGCCATCTCAATAGTTTTTTTTGTACAGAAAACTAAAAATGGGCTAATCAAATCTCTACCTTATAGGCAAGGTTGCCAGTCAATCAGAAAGTAAGATGAAAAAACACATGGATTTAAACAAATTAAGAGGACAACTCAAATTGATTTTACTCTGAAGAGCCCAGGAAACAACGGAATATCTCCACTGAAGAGAACCTCGAAGTAAATATAAATATTTACTCAAAGATAAGAACGGTCAAGAAAGAAAATCAAACTAAGCCTTTCCCTTCTCGAGCCAAACTCAGCCTCGGGACTACATAAACACCTAACTTCTACATAAACGACTAACACTGTAATCAAAAGGGAAGACTGGATCCACCCACACCATTTTGTAAAGTTCTAGGTAATTTTTCATCCAATCTTCTCACGAGGGGAAAGGAAAAGAAAGGACAGAGGGTGGGAGGGTTGAAGGGAGTGGGGGGAAGGTGGTGGAGGAGGGGGGCGGCTGCCAGATGAAGGGGAGAAATATCCCCCTTTCCAAATTCGGTTCGCAGAAGAATGCTATTCTCGTTTCTCTAATTTCCATTCGATAGATAACAGTTTGTTTTGTCGTGAAAATATTCGCTTTAATTACATGAAAATCTATCGTTTTTATATGAACAGAGCAGTAAATAATGCTCGTACGAAATACTCGAAAGGAAAGCATCAGCCAAAGAGAGGACCAATGAAAACGAAGCCAAAAGAAAACGAACGAAAGTATTGTTTCGAATAATCGGGAGAAATCTCCCGGAGGGAGGGGGGAGGGAGGTGGAGGGGAGAAAGGAAGCAAGGCAAGGAGGTAGAGGAGTGTGAAGGGGAGGGGGGCAGAGTTCCCTGACTCCGTTCTAATAAGCAAAAAGCTCAGGTTTTACAGGAGAAACTAATCGAGTAATTGATTAAAGTGAGATTAAGCATGTCAACAAGACTATTTCTCATGGCGTGATAAGTCGCTCACTAATACAGCCTCACTCACTCAATGTCTCTCACTTTCCCCGAAGGATTTAACTTCAAGGAGCCACGTTTATGCTCCTTACGCTCGTACTCAGGATGGGGGACTTTTAGCTCCGTTTACTGTCGGTCGGTCTGTATGTCTGTCTGTCTGTCTGTCTGTTAACACTTCTTTGAGGAGGTGTAGTTTAATTTCCAATAACATAACGAATTTTTGCATATATAAATTTGGTAATAAATGCTTGAATGCATATATACAGAAAATTTAGTATGCATATATATATATATAGCATATATATATATATATATATATATATATATATATATATATATATATATATATATATATATATATATATATATATGTGTGTGTGTGTGTGTGTGTGTGTGTGTCGCTTTATCAAAATTCCAAATTTTAGCGAAGGAAAAGCTGAAAAGGACATTTCATATAATAAAACATGATAAATTGCAAAGAAAGAAAACAAAATTGCGTAGTTGTTTCTTTAGCCATTCTACTTTTTCTATTAATCTCTCTCTCTCTCTTTCTCTCTCTCTCTCTCTCTCTCTCTCTCTCGGCGAGAGTGAGGTGCATTAGGAAAATTGTTACCGCCAAGAAAAATGGTGTCCCGCCTTCAGCCATTCACGCACAAAATTTTTCACACCAATTTGTAACGTCCCTGAGGAATCAATTGCGATAATGAGAGAGAGAGGGAGAGAGAGAGAAAAGAAAACTGAAGCCTGGATGCAGAATGTACGAGTTACTGCTAATTAGGAATGTATCACAGAAAGAAAGAGGAAGGACATTGTGTTAGCGATAAAAGATAACAGGAGAATTAAAAGTCTGAAAAGGTGATAGTTAAAACGACTGACGGCGGCATATACACATGGAGGAAGATTATGAAATTCGGAAGTAAGTAAACGTCTAGTACCTAATTCATTTCCTATCAGATTTCAGTGCTCTCCGTCAAATCCTTTTTCAGAATGGTACGAGATGGCTATTACAGTACAACTTCATTTACCCGTTTTTTTTTTTAAATCTCTCTCTCTCTCTCTCTCTCTCTCTCTCTCTCTCTCTCTCTCTCTCTCTCTCTCTCTCTCTCATCATCAACGCTGTCCCTTCCTGTCCCTCCTCCCTCGTTTTGCAATCCCAATTGAAAATGTCTTATACTGTCTCCACCATCTGTCAATATTTCCAGCCCTTTGTTTAAGTCAATGACCCCTCGCCGTGAGTTGACAAAACACGCGCCCAATTGACCCCTTCCTAAACCAATTGTTTTCCTATAAAAAAAAGAAAGACAAAAGCCGACAATTCCCCCGTCAACCCCAACTTGCCTTCAAACAATACTTTTTAAAAGGAAACGTGGTTTACTATTTAACCCTCAGCGAATTTATTACCCTCTCATATGTCTTCGTTCGCTGTCTAAGCGATTCTGATCATTCACGGAATTTGTTCAAATGCTTGTAGACCTTTTAGCAACAAAGAAATTTAAGTTGACTGTTGCACTGTAATAGCCATCTCTTACCATTCTGGATGAGGTTTTGACTGACATCCTTAAAATCTGATAGGAATCAAATTCAGAAATCCTCTTTCCTAGGTCGGATTCGAACTCGCGCTGACTAAGAGTCGTTAGTTCATTATACGACTCGGAAGGATAAGAAGAGTGATGAAGTCATTGCAACCATTATGTAACTTAGAACACTTGAGAGTAATTAGTCGATTCTGTAATTAAGGATTTACCTCAATGGATTACACGATTGTATTATTACTGTATAACTGAATGGAATCTTCAATAGTGAAACAAGTCAGAGATGATATCCGATAGTTTGAAAAAAATTTCAACGTGAAATTGTTTTCTAGTTACCAAGAATTGTCTATCATGTGACTTTCATTTACCAACACCAAACTTTTGACAATAATTTATAAATCTAAAACGGTTTTAAATAAGAATTAGTAAACTGAGTTGAAATAGTTCTTGATGATAAATAGGTATTTTCAGCATAAAAAATACGACGCAAGTGGTTCAAGTATTCCTTATCTGGAAAACTGAAATCATTCCTTATTGAAGATGCATCTTCAGGCCATTTATTGCTCACAAATTATTTTCTATATTGTCCAAAAACGAGACATGTTGAAATAAAAAGTTTCTCTGGTGATTTCGTATCTTCCATTAATTGCACAGAATTTCAAGCTGATCAGAATAAAAGAGAAATCCTTAAGTGTCTTCCAATATCAGCAGACTATAAATAAAAAAATGAGTTTATTACTTTATCAGCCAACTATCTTGACTCTGTTGGTAAACTTAAAATGGACTTCTGAGTCACGGAATCAAATCTGTCACATGAAAGAAGAGTTAGAATCGTGAAAAAAGTGTATTGCATCTTAACAGACAATATACCCTTCCCCTTTCTTTTCTTACATATTGGCACTGTATTCAGTGCAAGCTTGGCTCTTTTATGCTTGTTTCGTACGCAAGTTTGTTTATTGTAGGTGAGAAAAATAAAAATTTAACTCACTGCATCCAAGCTTCCGAAAACTGGCCAGAGGAATCGTTGGTAATACTTAAACGGGGCTACTTAAATTTCTTTTGTAGTAATCATTTGAGATACTTCAAAGATTATTGCTATCATTTAGAAACACCTCTTTATAATAGATTACTTTACAACTATGTTCGTGAAATGAATCTTTCAATTCCTATTGTGGATATTACTTTTTTTGATCTACCTCTTCTTTGTAGTAATCATTTATTTAAAAAATAGTCCTTTCCTCTTAATGAGAATTGTATTCAGCTATCCGTGGCTTTAGAAACCTCTTTATAATTTTGTAAAACAATGTCATCACATGTCCAGTCTTTCAGAAATGTCATTTCAAAAAGGCTGGCAACAATCTCTGTAATTTTAAATGTCAGTATCCACGTTTGGGTGGAAAAATGAGGGCTCAAAGGTCAAACTCTTTCAAAACGATCATTGTTGCCATTAGCCGTGATGAATTTTGATTTGTCTTCAGTCATGAATAAATCAACAGATTTCGATTCAGATGGAAAAGCTAAAAAAAAGCACTGGGATAGTTAATGGGAGATTGAAAAGGGCAACTATAATAGACTAGGCACAAGTGGGACAGAGTAAAGGGTAAAAAAAAAAAAAAGTTAGCCCTTTCCTCTTTTCGAACGGTATAGAAGTAAAATTATGATATTCGTTTATCATTTTTTACCAACGGCAAAAGTACTAGCTTTAGCAATATCACTATTCAATCAGAAAAAGAAGTATAAACAAGAGAGAGTGAATTTGGTGATAGTTTTAGGCTTTTATATATTTTACTAAATATGTAAAACCAATGCACAGTTAAGAAGTTCATTCCTGCAGTCTTTGCAGCAAAATGCCATTTCAAAAATCGAGGTTGCCAATGACTCCAGGAACTCTGAAGATTACAAGTATACGAGCTGCCATGATGCACAAGGTTAAGTGCAGAAACGATGACAGACAGGGTGGAAAAAAAGAAGTAAATGCATTCACCTAAAGGCGGGGCAAATTCTCCATTTATTTTTTTGGGGCCTCGTCGGCCCTCTTTGACCGATGTTTGAATAATTGTTTCAACGCATAGTTAGGAAAATGATTCATCCTTTGATGAATACGAAAACTGATGCTCAGTCATGAATACAGAAATTAGGCTGAAATCAGACAGATTTCGATTCAGATTGGAAAAGAAAGGGTGAATATTAATCGACCGATATGAAGCCTGAAAAAAAGCCCTGGCATAGTTAATGGGAGATTGAAAATATATATATATATATATATATAATACACAAGTGGGACAGAGTAAATATAAATCAGATATAACTCAGTGGGCATACAGTTTTTGATACTCAGATATTTTTATCTCAATATAATATAGTGATATTTTGTCTGTTGAAATTAACACTTGTAACACACACAGTCTTGTATCTTTTGAAAGAGCACAATGAACAGTTGGGAAAAAATTTTCAGATGAAGAGAAATGAAAGAGAACGAGGAAAAATCAGATGAAGAGAGAGAGAGAGAGAGAGAGTTTTTCTGTCTAAATTTCTTTTTATATACTGACGGTACTCAACAGATTGAGCATTCCAGTTTCCATCTTTATTATTAATTTTCCCTTTACTTTTATTTATATGGTTAATATTACTTATGGAATTATACTCGACGAAACAAAAGTCAAAACAAATATCAATTAGATTTACATTTTTCTTTATTGTCTAAAAATAAACTATATAAGTAAGCAGCTAAGTGCATTAACACTTTTCAAATCTAATTTATAACGAGTAAGAACTAGTTAAAGTGCTGTCAGAATGAAAAGGATTTATAATTTTTTTTAATGACGCGGTAATTAACGATGATGGTTATTGAAACATCAAATTATTAGTAGGTTCGTAATGCAGGTGGATTATTGCGTGATTTGTCAGTGTCCCCTTATTGAACCTACTCTTATTCTGTCACGGGTGTGATTGAGACTAAACTATTCCGTGTTTATAGGATTCAAATCCTTCTTCACTGACGCCATAATTTCAAGTAAATGAGATGTAATTTGAATTTCATGAAAGGAACAATAACCGATGTTACTAAGTCGGACAGTTCCGGTAATGAAAGTCTGCAGATAACAAAATATTTTTGCAGGTTTTTTGTATTTTGACATTTGATGCAGTTTGCTTTAGAATTTCCCGTAATGTGCTGGACGCGTACCGGGAGAAGATATAAAATCTTGAAGAAGTAAGAATACTTATTTTACTCTGACAAGAAACGACAAATACTAAAAGCCTTCTTATTATGTCACGTATAAACAAAACAAAAAAAAAAAAACAAAAATCCGGGAAAATCCTGTAGCTATGCAAATGTTTTGAAATCAAATTAAATGCATATACCAAAAAAAAAAAAAAAAAAAGTATGTGTCTATGTCTTCAAAGTCTTGATGAGACTAAACTCCCAGCGCCCGCCGAGCATAAAAGACACGAAACGCCACGAACGGGACACGGAAGGATTCGATTCGTTTCTTACCATTGTGATCTGGGCCAAGTCGACTGACCATTCAGGAGGCGGAGTTTATCGTACATGGGGTCCTGGGTCGGGGTGCTGGTAGGCGTGTTCGAATTACTGCTGCTGGTGGAGTTCCCGCCTCCGATGCCCATCGTCTGGCCCCCTCCGACGCCGGCACCGCCGCCTCCAGCTCCTGCAGTAGCCACGGCGCTACCTCCTCCTCCTGCTCCTCCTCCTCCTCCTCCTCCGCCCGTTCCTCCGCCACCTCCTCCCCCACCAGGGGGTCCGCCAGGGACCGGTGCGCCCCCTGAGCCGCTGCCGCTGCTTTCCTTCGCGTTGATGTTACGGAGGACGCGGTTGATGGACGACACCTGCGGACACATCAAATTAAGAATTGAACTCACACTCCAACAAAGAATTAAAAAGTGTTCATAAATTGTAGATTAGTTTAACAAAGAACAAATAGAATGTTCACGAACTGGAGAATATATCAGTTAAAGAACTCAAAATGTTCATGGACTGAAGAATAGATCAGTTAAAAGAGGTTAAAGCATTAATGGACCTAAGAATAGATCAGTAGAAGAGAGATGAAAATGTCAGTGTGAAGAATAGAATAGAGATGAAAGTGTTCAGAGGTTAAAGAAAGTGTTCATGGAGTGAAGAATAGAACACTTAAGGAGAGATGAAAGTGTTCATGGAGTGAAGAATAGAACACTTAAGGAGAGATGAAAATGGAGTCATGGAATAGAACACTGAAAAGTGTTCATAGAACACTTGGGAGAGATGAAAATGTTCATGGAGTGAAGAACAGATCAGTTAAACAAAGATTAGACAGAATGTTTATAAAAAGCAAAGTCTTTAATTTCAGTGAAGAACAAAATAATACTGTGTTCACGTTTACCTGACGGAGAAATTATGAGATGCCTACAACTATTTAGATGAATGTACAACTACAGAGGCTTCACAGCTAGTCGCATAAGATGAAACAGCCACCACACCAGAAACGTTTGAAAAGTCAAAGAAAATTATTTGTATCTTCTACTCATGCAGCCGTTTTGGATAATCTGTGACCTTTAAAATTCAGAATCTTTTATCAGGTTCCCACACACATCTGTACCGCTGGCTGCCCGAGAGATAATACCAACTGAGAGATTTCCGAGTATTTGCCAAATTTCTATAAACCAAACATAATGCCCTTATACCCTTACATTTCTCTCTCTCTCTCTCTCCCTCTCTCTCTCTCTCTCTCTCTCTCTCTCTCTCTCTCTCTCTCTCTCTCTCTCTCTCAAATAAAACAACAAACAGCATCTCTATTCAGCAAACAATAATAATTGTTCTCCAAGTTCAAAAAACCACAGCCTCACTGCATCTATGGATTGCCTATGATTGTCCCAGTAACTAACCTTATCAATATCTGCCGAATATTTCTCATAGTCGTTTTTTTTTCAGTAAAGTTTTTTTTTTCTCTACAATTTACCTTACTCATCAAAAGTAAATGAAATCTAGATGGCGATCATAATCTGAGATTTTATCCTAAATTTCCATTGCCGCTATCATCAACCTGATTTACTTTTGGAGGTCATTCTTCCGTATTTCATTCTGCTCATTAGGCTGCATAATTTATTGGTTACAGTTTCCATATCATTCTCGTCTTGGGGCAAGTTTTAGAAGTCCTTCGGCATTATGATTAAATTCATGCGAGTCGGTATGATTCAATTTATTTTCGTTTTTCTCTAAAACATTCATGAAAATTCTGTGTCCTTGGAAAATGAAAGGCTGCAAAAAAAAAAAAAAACTAAAACAGGTTATATAATCCTGTACAATGCATAGACGTTAATACATTTATACAGTGCAATTTATTTCAACCATGTATAATAATCGTACCAGATACGACAAATTTTCCAACCGTGATCTCCCCTTCCTTCCCCACCCCCTCTCTCGTCCTAAGAACACCCCAATCGCTTCGCATTCACTATGACAATGCCCAAGTAAGGCATTCCCTTTCAATAACGCCCCTCGCTATTATTAAATAACAAGTGCAGAGTTACAAAACAATCGCCACACGACATCGCCCGAAGAAGCTGATTGGAACCTCTTTGTATGGCGCCGCTGACCCCCCGCGAGGCGCTCGAATCAACTGTGCGACCGCCGGTATTGTGCTGCCTATAATACCCACTGAGGGACTTCCCAGGGTATTATGAACAATCGTACACGGTATTCCCCAAACTCACTCTCTCTCTCTCTCTCTCTCTCTCTCTCTCTCTCTCTCTCTCTCTCTCTCTCTCCCCGACACAATATGTGTCTCCCGGATAAACTAACTCGGACGGCGCAAAGCTTCGTGATGTTTATTGAAACCCATACTTTGCATGTTTCCTAAATGCGTCGTTGTGTATTTGTGAATGTTGGACCGAAAAACACTTTGTAATGTTTCTTATAAATTTTTTTCCATCGAAAAATGATAGTAGTCATGACAAGTAATTGGCATTTTCCTCTTCCTCCTGAATATTGCAATTGATGCAATATTCTTTGCTGTAATTTTTTATTAATTTTCAAAACTCCTTTGACTTGTTAATACAGTTTCATTTCTCTTGTGAGTCCTGGTCTCACAAGGTTAGGCCTGTGAACTTGGTCAAAATGTTACTCATTATTTCTAAGTCTCTTGCGCTCAAGAGTCTCGTTCCCAATTATTCTATAGATTTCATTTAAATATTGTCTTTAATGTGTCGACACTGCACAAATTATTACGGACATAAGAAAAGAGACTTACGCATGAGACTGGAAACGTTTGTTCAACCATAACATTATTACAGCAAAATAATAAAGGGGAGAGAGAAGAGAGAGAGAGAGAGAGAGAGAGAGAGAGAGAGAGAGAGAGAGAGGCGGTCTAAAGAGGCAGCTACTCTCTTTGTCTTTGATAGTGTGGCAAAACAAAATAATAAAAGTTATTGATATATTCTTGTTTTGCTAACGTATCCAAAATACCACAATAGAAAGGCAAAAAAATCAGAAGAATTGTTTTAAACTCTGGGGAGACTTGAAAAAAGTAACTTTAACATGAAATTCACCTGAATAGTGAGCACGGCTTTATAGCCCATTAAGTTTGCCATTTCTTTTAATTTGTATTATGTTGCCTTCTTTCGTAAGGCAAGGGAAAATATATGAAATTATAAAAATCAAAGCAAATGTCATCCTAAAAGAATGAAAAAAATATGTATCATAAAAGGGAAAGCGATAAAACAACAATGAAAATGCTCGGGCAGTATTATATGCACCAGTTACGGGCACCAATATTCGATGCATTTATATAAAACAAATCGCCTTTGCCACCTATATTGTTCTCTAGGCCTACCTACCCACAACTCCCACTCCAATAAGGCCTGGAGGAGTCTTCCCCTTCCACCCCATCCCATGCTAGACTCCCCCCCAACACCTCCCACCCATGAAAACCAAGCCTCTTCTGTCATCTTTGTCTAAAGGAAACAAACGATATTGGAAAATGTCTGCAATATCGCCGATGCCAATATTGAAATCGTCTTTGACATTTCATCTTCGGTAAGAAGAGAGAGAGAGAGAGAGAAAGAGAGAGAGAGAGTCCTACGCCACTTTTATTAAAACTGAAAGGCATGATCCAAGGAGTACGGTAGCCACCCCCAGGCTGGGCGTGTGTGTGTGTCTCTCTCTCTCTCTCTCTCTCTCTCTCTCTCTCTCTCTCTTTGAAGAAAAAGAAGAAGCATCCGATATTTTCTAATAGGACGCATAAAAAGTCTCTTTGATGGCGAAGCCTACACCGACGCTGTAATCTGGATTAATTGGTTCAGTTTGTATCCAATTATCAAGAAAAACAGGGGGGGAGGAAGAGGGCAGAACGGCGATATGAAGGAGGTGGGGGGGGGGAGGTTTGAGGGGCGGGGGGATAATAATGATAAAATTAGTAATATATATACATTATTATGAAATAAGAAATGAAAATCTACAGTTATTTTCAGAACAATAATAGATTATTTTAGTCTTATTACATGGCGCGTAATGGAAACATCATTGCCATTTATGAGGGTCATAGCTTTTGGTAGAGACGTTTTCAGTGTTCCAGTGACCTAAGACGTAATGATATATTATAAAATCAGTAAAATCATCCGTTCGGAAATGTTTCAGGTTTTCTGCGGTCTTCTAACTGCTTGACCAAGTGATGATGGGACACTAGGTTTATCACCAAATGAAATAAATTATACATTACATAGAAAGTATTTATGTGACAGAAAATACGGCAAAGTTTGTGTGTGTGTGTTTTTGTGTTTACGAGTACGCATGTACGTGGTGAGCAGTGTTTATGAAGCAACACGTCTAGTCTTACTAATAATGTCTTATAAAGTATAAAATATCCAGTAAGAACACCATTTAAGACCTCTTGAAGGTGCTTTGACACCAATAGATACGTTGCGAGTCAGTTTACTAAATTTAAGTCATTATGTGAAAAAGAAACAAAAAGGCAGGATATTATGTAGATCCAAGTACAAAGACACCATTTTAACTTGAATAAGCAAAGTAACGTGTAAAATCCCTACAAAAAAGGCCTTGTGTGAAAACACGTATAGGCTAATTTCGCAAGATCGAAAAATCAGATTAATGAAGGCAAACTGGAAATCTTATAGAGGTTAAATAATAATGAAGCCAGGCATCACACCATATGCATCAAGATACCATCAGAGCCAAATATATGTCATAAAGTCGAACAAACGTAAAGCAGACTGGGAATGTCCCATCAGTAATAACAACAGAAAAATATTTCGAAGACAAGATCTTCATCGTCCATTCCCTGAAACTTCTAAATGAAGCTTAGAATCCTCGACCCAGTTCTACATTATTATAACAAGTAGCGAAGAACAATGCATTACCACCCTAAAACAATTCACCTTGTATTTTAATAATTTATTTTGTTTATGAAAAGTATTAATTTATATTTATATTTCTTAATTTAATAATAACTGATATCCTTATTCTTTATTTCCCATTGCCTTCTCGTACTTCTTCCTAATGAGCACCATATTCTTTGGAAGTTTGAATTTCAAATCAATGGCTCCTATGGGCTTGTTCCATATGAATAGGGTTCATCGTCTGAATAATAATAAAAATAACTTTTGTCCATTTTTACCTGTAACCTATCACTTCAGGCCTTCAGTTTGACAGGAGAACCAAATATTTCGATAGAATTATCGAGATGCGATAAACAAGGGAAGAAACGGAGAACAGAACAAATTACGATATTTAAGACAAGGACTCGATATTTTGAGCTCTTTAGGGTATTTAGAGTATTTACAAAGTGAAAAGTTCAGGAAAAAGTTAGATTCTGAACTAGTCGGGAAAACCTTAGTTTATCGAGATTTGAAAATAAAAGGGAGAATGAAAAATTGATAATTATGACGTTGAGAAATCCGCTTTCATTTCACAATTGATAGTAATAAAATGCGAAATTACATTAAAAACAAGAATCCATGATAAACTTTGATCAATAATCCCATATATATATATATATATATATATATATATATATATATATATATATATATATATATATAATATATATATATATATATATATTTATATATATTATATAAAGAGACAGAGAGAGAGATGTTTACTCTCACCTCCACGATACCTATGAAGTCACCTAGTTGGTATGTACCGTTCCGTTCCAAAGGCAGTGAAACTTGATAGGATGCTATAATAAATTGGAATAATCGAATAACCAGAAAACTCGTTACCAGCATCCCCAGGCTAGAGAACACACAGTTTTATGTGGAGACTTTTTATCAATATATATATAATTATGTATGTATATACACAGATATATATATATATATATATATATATATATATAAATATACATAAATAAATATATAGTATACATATATATATATATATATATATATATATATATATATATATATATATATATATATATATATATATATATATACTACGACGTTTGATATGTGTTGATACAGTGAAGAAGGGAAGATTACAATGGCTTTGACATAGCACCAGAAAAGTTGGAAGACCCAGACTTGCTGGAATAAGAACTATTGTTTGGTGGCAAGTCGAAATTTGCGGAAGACAAAGCACAGGAAAGGCATGAGTGGTGGAATTTCAGAGGTACCTTCGTTACACGATGTTGAAGGCGATGATGATATATTAACAGTTTCATGAATAAAGAACAGTAGTGAGTCTAAGCATTAAATGTATGTGTTAAGAGAGGGAGGAAAAATTGTGCTCTTACTTTCTCATTTATTGTTCTGCATAATAATAATAATAATAATAATAATAATAATAATAATAATAATAATAATTGGTTTTTAAATGTTATCATTTTAGTTTTCTGTAAGAGAAAACTATTGTGCCGGCTTTGCTGTCCGTCCGCACTTTATGCTGTCCGCACTTTTTTCTGTCCTCACTTTTTCCTGTCCGCCCTCAGATCTTAAAAACTATTAAGGCTATAGGGCTGTAAATTGGTCTGTTGATTATCCACCCTCCAATCGTCAACCATACAAAATTGCAGCCCTCTAGCCTCAGCAGTTTTGATTTTGTTTAAGGTTAAAGTTAGCCATAATCGTGCTACTAGCAACGATATAGGATATGCCACCACCGGGCCGCGGCTCATACAGGGTTATACCAAGACTACCGAAAGGTATAGATCTATTTTTGGTGGCCTTGATTATACGCTGTAGCGGCTGTACAGAAAGCTCGATTGCGCTGAAGAAACTTCGGCATATTTTTTACTTGTTTATTATTTTTACTAATATCCTAAATATTATTACTGTCTACTATTATTATGAATAATATTGTTACTATTAGTAATAATAGAAATTATTATTATTATTATTATTATTATTATTATTATTATTATTATTATTATTAAGAACAATTAGCTTTAAATGGAAAAATCAAGCTGAGTTAAGATTTAATAGTTATTTTTGTACACAAACTATAGAGAAGCAGTTTACTTACATAAAACAGTACAAATTAATCCTGTTCCTCGAATTTATTGAAAGACCTGAACACTGATTATCAATTACTTTGATTACATTAATTCCAGAGACATAAATACGGTCACTAACAAAATTTGAAAATTCGGACAGAATTCGGCTCTAAATGCATTTCGGAAAGACACTGAATAGTGAAAGCATCGACATAAGCAGTCTTCAGGAGTTAAATTTCTCTCTCTCTCTCTCTCTCTCTCTCTCTCATTTCTCTCTCTCTCTCTCTCTCTCTCTCTGGTGAAAGCATCAGTCCTCCGATAAAGTAATCTCTCAGGAGTTAAATTTCTCTCTCTCTCTCTCTCATTTCGGAAAGACACTGAATGGTGAAAGCATCGAGTCCACCGATAAAGTAGTCTTCAGGAGTTCTCTCTCTCTCTCTCTCTCTCTCTCTCTCTCTCTCTCTCTCTCTCTCTCTCTCTCTCTCATTTCATTTCGGAAGGCACTGAATGGTGAAAGCATCGTGTCCTCCGATATAAGTAGTCTTCAGAGAACCCTCTCTCTCTCTCTCTCTCTCTCTCTCTCTCTCTCTCTCTCTCTCTCTCTCTCTCTCTCTCTCTCTCTGAACTCCGGCTGCTGTAAATAAACTCAGAGGTCAATGTGCTGCGATTGCTTTAAAGGGCTTTCCACTGGCGATCAAAGCTTGCTTAATGTGAAACTCGTCAGCTGAGGAGGACTCGAGTTCTCTTCCCTCAACTTATTTTGTCTCTTCCCACGAAATACGCAGGACTTCAAAGTCTTCTCTTTCTGAACGTTATCCGTCTTTTAAAGGAATTTTCGTACTAAATGTACATTTCAAATAGATAAATTGCGTTTATAGTTTTTGACAGGTGATGTCTGTGTTTACGTATACATTAATTATGGACGTAAGGCATGTATGCATCATTGTCCTTATGCTTGTATGTATGCGCATACAGACAAGAAAACGCGATATAGGTTTTTATGTTTTGCTAATAAAAGTAGAATTTTAATGTATATGATTTTCTAATCTCAGTTTGTGTATATTTAGCTAATGGTACGTATCGTGAGATGAAACAATTCGCTCATTCAGAAACTCTTGGTGAAATAGTTGACAGATCAGGGGAAACTGAAAGTAATTTTTATGGAAATAAGCGAAGAGAGAGAGAGAGAGAGAGAGAGAGAGAGAGAGAGAGAGAGAATCATGGTGAAAATAAATTAGTTGGCAGGGAAGGAATACAATGAAAATAATTTTTATAGATATGAGCAGAGAGAGAGAGAAGAAGAAGAAGAGAGAATAAATCATGGTGAAAATAATTGACAGGGGAAGGAATATAGTGAAAATAATTTGTGTGATGTGCACAGAGAGAGAGAGAGTGAGAGAAGAAGAAGAAAGAGAGAGAAGTTTCCAATGTTTGTGTTTGTAACTTCAAGCGGATCGGGGGGAGCAATGGAGTTATTCACCCTTGCCAGTAATGGCTTTTAGGGTGTTCCAAACATTTTTTTTTATATCCCTTTACGACAGTTTAGACTTACCTAAGGTGATTAAGGTCACGATGAATGAGTTCCGTTTAATTACCTGGCCTGCCGCATCCCTACGCCCCCTTCATACCACTTTCATTGCTTAGCTTTACAGGAGAGGGGTGGGACCTCCCTACCAGGCCCACACGGACAGACTCCCCCCGCCCCTTACCTACCAAATGTTGTTACAATGAAGAGCGGAGATATTCCTAAACGGGTGATTCAAGTATGGAGTAACATTGGAAAAAACTCACTTTTTCTTCTTTTGGTAGACTGCCTCTTTGTCTCTCAATTAGTCAATCACTCCACGTGTTTATACATATACATACACACACACACACACAACACACACAACTAACTAAATATATATATGTGTATATATATATATATATATATATATATATATATATATATATATATATATATATATATATATATATATATATATATATATATATATATATATATATATATATATGTAGAATCTGCTGGTCACTTTTTACCCAGATACATATGTAATTGTAATAGCCACAATGCCCTCTCAACTTTCTCGAATTCTTCCCACTTTTTTTTTTGGATACGCTCGTCACTACAAAGCATTAAGATCCAAGTGCAAGAAATATGAAGAAATTCCGATGTGAGTTTCACTAACGCACATCAGAATTTTTCATATTTCTTGCATAGAATCGATTAACGTTGGTAACAAGCCCGTCAAAAAAAGTCGAAAGATCGAAAATTAGAAAATTACAATATTACATATATATATATATATATATATATATATATATATATATATATATATATATATATATACTGTATATATATATATATATACACACACTTATATATATATACATATATATATAAAATATATATATATATATATATATATATATATATATATATATATATATATATGTATATGTACATAAAATGAACTTTCGCTAGTCATGGTACCTTTATGAAGGACAGTAACAGCTAGCGGAAGCTCACTTTTAAATGCATATATATTTTCGTTAGACAATACAAGTACGACATGTGTACAGTGTCGTACTCAGGTAATTAAATGAAGAAACTCTACAATAATTAGCATTGTCTACTATATATATATATATATATATATATATATATATATATGTATATATATTATATATATATATATATATATATATATATATATATATATATATATATATATATGTGTGTGTGTGTGTGTGTGTGTATTGAAAAAGTGAGCTTTCATGGCCACAGTTAGCTTTGATATAATAACAGTGACTGTGAGTCACTTTCAAATAAATTTTCTATGTAAATTTCATATTGTTGTGGAATTCCCTTTGTCACACACACACACAAGCATGTACCCATACACTTACGAGCGCGCGCGCACACACGCACACACACACACTAATATATATATATATATATATATATATATTATATATATATATATATATATATACATATATACATATATAATATAGAAAACACCGTAATACATGATGTAAACATTCAAATGTATGAAACCGAAAATTAAAATTGTATTAATCAGGACATAAGATTAAGCAGAAACCCTCAGTAAAGAGCTGACAGGACGGCAACTAACAGAAATCTTCTTCTTCTTCGTTTAACGTGCTTTTTCCCATTTTTTATATGGGGTGAGCACGATGCCTTCTTTTGAAGGACTTTGATTTGGCGTTGGGGTAGGCCGTAGCCTCGATCGGCTGCCCTGCCTGACATCGCTTAGACCCGGTAGCGTATAGGTGCATGTATCGTACCAAATCCCCAGCTCCCTTTCTTCCAGCAGCGAGGAGAATTGAGCGATTAGGTTGACAGTTCGAGACGTGTGAAGTGTCTGGCAACTAACAGAAATAGATTAATGAAATTATATTAAACTCTCTCTCTCTCTCTCTCTCTCTCTCTCTCTCTCTCTCTCTCTCTCTCTCTCTCTCTCTCTCTCTCTCTCTCTCAGTATCGATGTTTCCAATTATCATTATATATAGACTCAATCCCCTCCTACAGAGATGCAATTAAAAATACAATTCCGAGTAGCTGGAAGTTTTAAAAGAAATCTTCGAAATGACCTATTATTACCAGCCTTGAATGAATACATGTTAATATGTCCCCCAGTGCCCAGGTGCAGCGAGTGAACGTAAAGCTTTCAGAATATTTCAAATATTCACCTCTCGTGGGATCATTACCTAGGGGTAGAAAATGATCAGTAGGATGTGACTAACCAAACCCAAGTTGAAGGGTCGGTGAGGCTCAAGAAACTACCCTTAGGAACAACGCAGTCAGACTAGTGTCCATTAACAATTACTTTTGGCAATTTAAAGATTTAGTAAAATTACTATGATATGGTTCATTAACTCCCACCTGTTTAAGTTTGAAAAGCAGGACCAAAGTCAATACCAATCATAAAAACTTCATGACTAGAATATAGAGGAAGCACATCTACTCTGACCAGAATTCGAACTTTGCATGCTGCGATGGGTTGATATCTGATTTGCAATGACGTTATCCATTCAGCCATCGATAATCTGTCAATTCCACAAATGAATTTTTCCTTTAAACATTAAGCCACGAATAACCCATAAACACGGAATTCGCGTATATTAGGAATAATGCTCACACGAAGGAAATCAGAATGGTAAACAATTCAGACCTATGCCTTGTAGGGTGGGCATGTGGGCGACAGTGGCATTATAAATTTAGCTATCAGGAGAGATAAGAACGAATTTCGACTGTGTGTTGCATATTCCTATACACTGGTTTCAATTATTGTTAATGTCAAGTTAATTCTACACGGATGGGTTATCTGTGGCCGGGTATCTGAATGTACAGAAAAATTTGTTTTTATGAAGTCTGAAATGTTGAGTGTTGTATAGTAAGTTATAGGTGCTACATATGAATTATCACTTGGAGAAAATAATAAAGGACTGTACCTTAAAAGCATTTATATACATATATATATGTGTATACATATATACACATATAGTTATATATATATATATATATATATATAAATATATATATATATATTAAATATATATATATATATATATATATATATATATATATATATATATATATATATATATATATATATTTAGTATATATATATTTATATACATAAATATATATATATATATAAAATTTTGTCATATACACCCGTGACATCTTTTACATTCATGAATATTAAGACACAAATATTGTTTAACGACCGATTCATTATACCTCGGGAACAACTTACATCCAAGGGGAATTACAACTGGTAGGTGCTCCGTCACCAGTAGGGTTCGAATCGCTGTCTGGTTGAGAAACAACTATGTACAATGAATTTTGACCACTGTACGATATATATATATATATATATATATATATATATATATATATATATATATATATATATATATATATATATATATATATTTATACACACACACACATACATACATATATATTTTTTATTTATGTGTATGTATATTGTATATATATATATATATGTGTGTGTGTGTGTGTGCTTTACTCAGTGTGTCCGTTTGGCCACATAAAAGAGAATCGGTAACATTTCAATAGTAATATAAAATGTTAATAACATTAAAATTGGATTCCATGACATGAAATACCTACTATAGAATCTTTTTCCGAAATCCTTATATTATTTGGACTTTGGTTTCAAGATCACTTCAGCTTTTCGTTAAAATTACTCATCCCAGCTGTATCAAAACTGTATTTGACTTTGCAGTGTTTAAACAGCGAATAAAAGTAAATGCTACCTAAACATATTTCTGAAGACTGAATATCAGTGAATAGTGAAATAACTTAACACTTTTCATTCTTTACAAACTAATTATGTCTTTTCTGTTCTTCATTTGTTCATAAGATATTTCTCCCTCGGCTGTTCATGAAGAACAAAATTCGTATTTGGCTTCTATGACGGGATCTTTGTCCACATATTTGGCGGCGTGGTCATTATATTATTATGACTTCCTGTATTCATTCAAAGAACACTCGACATTGCGACACAATTCGCGATAATACCCCAAAACCATAAACCGGAAACCTTGCACAACGAAACTTATCTTGAAGACTTTCATTCGCCGTCAATAAAACAGTTCCTTTAGAAGGTTTTGCTCTTTGTTGTACAGTTCCTATTTATGTGACGCCCAGGCGGTGTCTGTTTATGGAGCTATTGCAAAATCATGTAATGGGACAATTCATCAGTTTTGTATTTGAGAAGACATTTCTCTGTGCAAGACCTTTGCGTTAAAGCTGTTAAGGTATGAAACTGAATTTTTTTTTTTATGGAATGTAAATGAATGGAAACACTTGCTTTGTGAAGCTTGATCAAATCAAATCTGTTCCTCTGCCGGTTTCTGGATCACTTGTTTTATATATATTCCTCTTAATATAGCATTAGTACATGGCCCTGTTGTTATATTAATTATTATTATTATTATTATTATTATTATTATTATTATTATTATTATTGTTATTATTATTATTATGTTATTATTATTACTATTATTATTATTATTATTGTTTTCGGAAGAAGACCCTCTTTCAAACAAACTTCATTAAACAGGATAACCGCCTGGATGCCGTTGAGTTTCTATTGCAGTTTCTCAATCTCTCGAATGAGTTTCTTTGCAGCAGCGGGCAAATTATACACCAGCTGTCCAAAGTTCATTCGTCAAGGTATAAATGAACTTTGGACAGCTTTATATAATTTACCTGCAACTACAAAGAACCCACTCGAAAGATTAAGAAAGTGCAATACAAACTCAACGGCATCCAGACGGCCATTCTGTTTAATGAAGTTTATTATTATTATTATTATTATTATTATTATTATTATTAATAACGTTTAATGAAGCCCTCAATATTCATTAGTGTGTTACTCTACATTATTCAAGAGACAGCATTTCTAACTCACGCAGCAGCATTTGTGCTGTATATAATATCTATGGACTAAGTATTATATCTTCCATTAACCCATTAATTTCCCTACGAAACGCGTAGGATACTCCGTTCGTGTACCAAAGCTCGTTAAGTTCGTATGAGCCATTAAAAAAAGTTAAAAAGTATCTGCATCTATGAATCAAGCTCACAAGAAGTATTATAACTCATTGATCAGGAAAATGTCTCTGCGTGTGTTTTTGAGCACTTTCATCTCATTTCCTTCACGATTAACATAGTATGTACTTTGCTACAAATACCTCAATATACACCTACTTAAATACTTTTTATCTGTATAGAAATTATAGAAATTACTTTCTGATTAATTCTGTGGCACACAGGATTTGCACGTGGTCATAGATTATGAAGGGCGAATCTCTTAATAAATTTATTTTTGTAATGCATTTGACTAATTTTTCCATAGAAGGCCCATTCAGTGGCTCAAAAGGAATGACTCTCGCTGTACAAAAATCAAGCACATCAATTAAGCAAAAGATACTTTGGATATATTCTAATAGTCTTTTCGCCCAAGAATGGATTTTTATTTGCACCTTGTTAATTTATGGAAAGATAATCAAAACCAGGTGTAAAAAAGAAGGCAAATTTAACTTAAATATATCAGGCCAAATTAAAGCCTTATGTTCTTTAAAAACTGTTATTATTATTAGTACTACAATTATTATTATTATTATTATTATTATTATTATTAAGAATTGCCGTCCACTCTTTCTCTTTAACAAACTGGACATGAATTCTGTATCTGCTTATGGTCTCTTGCTGAAGAATAATTGAAATTTATTATTATCAATATTATTATCAGCAATGTTTCGGTGCAACTATTCATTATTATAACGTTTTATCTTGTAAATGTAACTTTTGTGTACTCTATTGTATTTACCTTGTCTATCTCTTTATATGACCCTGAACTAAAATAAAGAATATTATTGTCCTGACGTATGAATTTTATAACAAAAGACAGTAAATTACAAAGTGATCTCTGGTAGATGAGTCGTTGAGCGTCAAACGCAAAGAACAAACAGAGAGAAATAAAACAAACAAATATTCCCTTAAATCACCTCCTTCTTCGTTCCCTAATTCTCCCTCCCCGTGTGTGTGTATGTGTGTGTGTGTGTACACTTAACCCTGTCCCAGGGGACCGGCCAACTCTCATCCTTTTGTGGTTGTGGCCTTCCAAGAGACGGGAAGGGTGACGGGGACCACCGGGGATCAGCCATTGGTTCCCGCTGAGTGACCGCCTCCCTTATTGGTGAGGACCCCTGCCTCGCCACGGCTCACTGCGTCCTGATTGGCCAAGAAACCGAGCGTCTCGGGAGAATTTATCGTTCGATTGGCTAGAAATCCCCCAAGACAAGAGGTAATCACCGTTGATTGGCTCAGATATATATTTTCTTTTTTTTTTTAGTCTCCTGCCTTGGTTCGTCTAATCCTCCCCTTAAGGAGAAATGAGAGTGTGGGGGAGGGAGGGAGATGGGTGGTACAAGGGGGCCGCTTTACATGGGATGAGGAAGACAACGATCACCGGACAAACAAACATTGACAAGGTCACATCGCGGGTCAATATCAAGATAAAGAGATTAAATTGGTAGGAAATACTTAGGCAGCATCAAATTGAACTTGCATTTGAGAGAGAGAGAGAGAGAGAGAGAGAATCTAAGGCATATTGACCGAAAGTTTTCCTTTTTTCTTCCTCTTCCTTTTGAAACGAGAGACTGTGAAAAGGGGTGCGGTTAGAGAAAACAAAAGGCAGCGGATCCCTTAAGTGGGGAGTCATTAAGTGACACTTAACTCCGGCCACGCACTTTCAAAGGATGAAAGGAAGAGAGAGAGAAAAAAAATGAAATGAAATGAACGAAACAAAGAAAATAAACAATAATTACTTGGGCAGTTTGATAAAAACGGATTTTCATTGTTATTATTTTTTAATTGTGCGAGGTGTGTTAGCACAGTCTCTTGCTTTCAAATGAAGTGAGGGATTGTATAATCTTAAAAAATAATGTCAAGAAAAAAATAAATATTCGAAAAAATTTTACCTATAAATTTCATAAAAGGAACTGGTCATGTTCAGGTTCAACACAGCTGTGGTAAATAAGTATTACCATAACTGTAATACATTGTCCAATTTTAGATGACTATGACAACCCGCGTCATAATATATATAAACTGAATCAGGAACACATGTCTAAATAAAAGATTAGAGCGTATTTACGGTAATGAATTCAATAATACTGAAAGTACACTGTTATTTTTAGTCTAAGTGCAATTAAATACTTCGAGAATATGAGTCATGTATGACAATCAACCCCAAAGCCAAGAAAGGTTAAGTAACTAAGGTAAAGAAGAACTTATAAAGGAAGGGAGAATGAAAATAGAATAAGATAGAAAAAACGAAAAAAATTGCGTGCTGTAATGATTTATATATATATATATATATAATATATATATATATATATATATATATATATATATATATATATATATATATATACATTATATATATATATGTATTTATACTGTATATAATAAGCATTCAGCATTAGAAGGGCCTCTCGCCCAAAACTTTTGAAAATGACGTTTTCCGCCTGATTTTCTTTTATACATACTATTCTATTCCTGATCGCAAAATACTTCATCTCATACCAAATTCAACTTTGTATCTTGAAAAATAAAGAAATTAGAGCAGGCTGAACCAAAACTTTGATATATATATAATATATATATATATATATATATATATATATATATATATATATATATATATATATATAAAGAGAGAGATATATAGAGAGATAGAGAGACATACGTCTTGTATTTATATATTTGTTCATCTTCCTGATCATACGATAGCGGTATATAAATCTTTCTGCATATTTTGATACACGCATAGACGTCTTAACATTCACGAATCTATACATTTTGCACATATATATTTTCCTTTACTGAGGAACAGGTAATCATAAGCAGTAATGAATGCATAGTCTGTGTAGCCCACAAGTGTATAACCGTATCCATGTGCACAGGTAAGGCCTAATAATAAAAAGAATAATTTCCTCTAAAACCGAAATTCCGTTGTAATCGTCAAACATTAGAGTTGTTCTCTCTCTCTCTCTCTCTCTCTCTCTCTCTCTCTCTCTCTCTCTCTCTCTCTCTCTCTCTCTCTCATTATTTCATACTTTTTGCTTAATTAGGATTCTGAAAAATCTCCATCTAATAATTGTTCATTTGATTTTATAACCAATACACTTGGTTTTCTAATTTTTATTTTGCGTGTGTATTAAAATGCATTTATAGCAAATAAGGACATTTACAGGGGGTAGATCTGTTTCAGAATTTAAAGTAAAATATGGCACTCAGTCGTAGAAATCTGAGAGAGAGAGAGAGAAATGCTTAGAAAATATAAAATGTCCTTTATTGACAGAACATTCTCTTCTATTACTTGATAGTAAATAATATCAATATGTATTTGCTCATCGAAATTAAAAAAAAAAAATAGAAATTATAACAAGAGCTTTGCATTGTTTTCCTTTTGGAACGCTTAGACCGCCCTATCCAGAGTAATTTAATTTAACAGGAAAAAACCTCTTAGCATTATAAAAAAATTTTTACAATTGAGACTTACAGATATATATTCCCTACTATCTGTTAAAATGCCTGTATTTCATGTTTAAGTTATTCCCCAAAGTTCTTTTACGTTATCTGTTGTTTTCAGAGGATTCTATGTAATAAAAAATACATCCATTTATCTTGATTAAAGCTAGCAAACGCTTATAAGAATGTGATCCACGCAACCATCAATTGACAGACTATTAACAAAATCGTATATTAACTCGGCTGATAGTCAATCGATTTTCAAGCCAAACTCCTAAAAGATATTTGCATCTATTTCATAGACTTTTAATTAAATATTTATTTTTTTTATTTTATAAACAGATCGCGTGGATCGAAGCCTAACATTTATGAAGAAGCTGACTGTATACTGTATCTAATTCTCTCCTCACTAAACTTTAACAATAAATACAAAAGGATTTAAAATACAATTTAAAATACAAGATACAAACATTTCTCGAGATTAAAGAAATCTTGTGCCTCAACCAGAAGAGAAGAACAAGCAAACTCTCTCTCTCTCTCTCTCTCTCTCTCTCTCTCTCTCTCTCTCTCGTGTTCCAAGGGCCGCGGTTTCAGGTTACCCAAGGAGTGTCAGGGGAACAATGGCCGGCTGACCGCCATGTTGCCCACAAAACCAGCCCCCTTGGGAACTCAATTGTCGCTGTTGCGTGGCATTCCTCTAAATTCTCTCCTCCGCGTCTTCTCTCCTCGACTTTCCTTATCAGCTCTGCACTATCCCTTTTCCTTTTTCGTTGGTGATTTCGGTTTGGTTATTTCTCTTTTCCTGCTTGCGTTTTTTTTTTTTCATTCTTTAATCAATTTGTGGAGAAGGTCATGCGAATTCTTTTAATATCAAAAAACATATTTATGGAGAAGGTCATATATAATATATTTTATATATATATATATATATATATATATATATATATACACACACACACACACACATATATATATATATAAAAACATATATATATATATATATATATATATATATATATATATATATATATTATATATATTCATTCATATATATACAATGTGTGTGTGTGCGTGGGCTCTTGAGATCAGTTAGCTTTAAGACAAAAAAATAAATCATAAGCGAAAATGATTCATTTAGGACTAAATTGTGGAAAAGACAGAACAATGACACTGCTGTTCAAATTGACAGTTTTTTTTTCACATTATGGATGACCTCCGGCGTTTCATTATTTCTTTAAGATTTTGTCCTCTATCGTTATTGCCGATGTTACTCTTCGATTACGTATCCTAATTTTTGTTAACCTTATTACCTACGCCCATTGAAGTGGGCGTTTCTTAGGCGTACCGTTGACCTGTTGTGTATGTTCCGGAATTTTTAAATGCCGCTATGTTCGGTCTCTCTCTGAGATTGATGTTACTGTTAATGATTTTGTCATTGTTACTGCCTCCTACAGTGAACTGTATTGAATAACGTTAGCCAACCCATAAACTTTATTATTATCATTATTATGTGGTAGTAGCCGTCCATTCTTACCTGTTAACAATTTGTGGCTGCACACTGTATCTGTAGCCCATATAGTCTCAAGCCGATAAAAGTATTATTATTATTATTATTATTATTATTATTATTATTATTATTTATTATTATTATTATTATTATTATTATTATTCGCAATGTCTGAGCGTTACTGTTCAATATTTCATTTTATATACATGTAATGGACATGATTGTGTACTAGACTGAATCTTCCTTGTTTTTTTTAATATTGTTATTATTACTATTACCAAATCTCGCATCTAATAGGCAAGTCGTACGCGTGTTATTGCAGTTTCAGAAAATCTTACGCAACAAAAAAGCGATTCCAGGTTCCCAGACTTGTAAAATAAGGAAATGACTAAAAAGTACAAAGGAAATATCTACAATATTAACATTTGATCCCTTAACACTTTTTCGTAAGTTTCCCGAAGATGGAATTTCCAGCCGAGGTATTGAGGTTAGTACACTTTCCACGAACGGCAACTTCCAGAAATAGTTGATTCATAGTTTCTATGAATCTTTTTCATGAAGAAAAGCTAAGAAGGAAGATTTGTTATAGTCTTAAATATAACTAGAATTTAATCATTCTGGATTAGGGGAGCCAGAATGACAAAGTTATCTGCCACACAATTCTGTGAAAATAATGAAAAACATAAATCGCATAACAAAACTTTTTGAAAGAGAATGCACTATCAAAAATGATTTACCGTTGACCTGGTAGTTGTCTTGAGATTCCTCAGGGATACAGATTACCAGATTACAGTGAATGGTATTGAACTCTATATGCATTTGTTACAAAAGTGCATTGTAAGTGCTCTATTCCCAATAAAAAAAAATGCTTGGCTGTCTCCGTTTCTGCGTAACGTTATGACGAAGAATCTCACAGGAGAAAGAATGAAGAATAAGACGTTATTCACTTCAGTAATGATATTGTATCAATCTAGCACTAACATTGACATCTAAGGTTTATTTCCTATATATATCAAACGCCACTGAAAAACAGTTACAATTCCAGCAAACAATCGAAAACTCAGACATATACAGTAAATATGTTTCCAACTTACTACCCACTTGCACCTCGCCACTTCGCCTTAAGAAGTCAAATGTGAAGGTCTCACATATGTGCGAAAAACAACATAAAAAAAGAGAGGAAAAAAAAAAACGCACGTCACACCCGCAGGTTCTCAAAGGAGGATGAGACAAACAAAACTCTTTGAAGGAGAGCTTTGAAAATATTTACAGTTTAACCTTTAATATCTTAGTCCGGAAAAACACGCGCGTCACTCCAGCGGGCGCCTTCCTGGAAAGGGGAGATGCTACAAATTCTTGTTTGCGTTTCATCACGGTTTCTCTCAAGAAGTGTGTGTGTGTGTATATATATATATATATATATATATATATATATATATATATATATATATATATATATATATATATATATATATATATATATATAAAGATATATAAGAGAGAGATATATAGATATATAGAGAGTGCGTGTCTGCATATATATATATCGGCTGCATGCGTCATCTGGAAGAGAAGATATTTCTTATTGGGTTATACGTTATTTCTTAAAAGCCTCCTTTTTCTCTTTGATTTTTCATCTGGATATGTCATGGAGCCAGGTAGGTTAGGAGTATTTACTCGTGCTTTTCACACTGGAATGGATGTTATCGAGGATCGTGGATTTCCGTCAAGCATTCTGTCGGATAGTTGGGATGGAATTACACACACACTCTTTCTCTCTCTCCTATATATATATATATATATATATATATATATATATATATATATATATATATATATATATATATATACATACATATATACACATATGTATAGGTATGCATATAAATAAATAATATATATATATATATATATATATATATATATATATATATATATATATATATATATATATATAGGAAGATAGATAGATAGATATCACAGAAGCAAGTAATTAAATAACTTAAGATATAAACAACTCTAGGTATCTGACTGATGACGAATAGCTGGTTGGCGGGTCGAGATTACTATGCAGAATAATTTATTCGTTATTTCATAGTAAATTTTCGTTCACAGTGCAAAGCAGCTTTTAATCAAACCGCATATATCTGTGTAAGCAGCAATTCTTATGAGATCTGTTTCCGTTATAGAGGTTTCACTACCATAGAAACATCATTCCAAGTAGGCTATACTTTTGTGATAACCCTCCTTGAGACATGCTATTTTTACCATGTAACACTATTAAAATATATAGCTGAATTATATACAGTAAGGAAAGAAAAAGGAAACCGAAAAATCTTTCACGTTGACGAATTGTTTTCTTTTACCTTTGAAAATACGCGAAGAACGCTGCACGTAACAGCCATGTATGGAAACAATTTTTGATTAACTCGCAATTATGTCACATAAATTATCGATATTTTCAACAGTTGTTTTTGAAAAGTTCGGTGACGAACTGAAAACAGCAATGTCCCCCATTAATGAGCTCGACCATTTTGAGGATTTAACTACTGCTTGCTTGCTTTCTGAGAACTGTTTGTCCTTGGCAGACCAATTTGCTTGGTATCAGAAACAGGCTAGAAATAATCCTTAAATAATTTTATATTACACATAGACACACACATATATAAAATAAACTGTATATATGCATGTATATACATATAATATATATTTATATATATATATATATATATATGTATGTATATTAATATATATTTTATACAAATGAAAACCACTTTAAAATAATGGAGCATATATATATATATATAAATATATATATATATATATATATAATATTGATATACATATAATGTTGAAATTTCAGTAGGTGATCGTGACAGCATAATTAACACTGTGATCGAGATAGAAAAAGTAAAAAAGTCAATAAAAATCAATTAATTAAATATAATGCAATAAAATGAAAAGGAAAGGCAGATTATGAAACACTCATGCCCCAATATTATCGAAATTCAAATTAAGTTTCTATATCACCTTAAGTAGAAAATAAAAGAATCGATGTTCCCTCGTACAGTACCTTCATATCGCAAGGGACACAGGGGGAGTGTCTAAGAAAATCAATAAATAATCTAAACCATCTTCAAGATCTCAAAAAAAGAAAAAAAAAACATCTAATAGGAAATCCTAAAATTCTTGACATACCACTTTTTGCCCTTGGAAACTGGGCGGGACGGGGGAGAAAGGGGAGAAGGGGTGGGGAGAGGGGGAGGGAGGGGGTGGCAGGTGGGGGTCATCCGATAAGGGAGTTCTGCGGCAGATTGGTTTGACCGCCGAACCTTCCCACCTTACCTGGGAAGGTAGGAGCGGGGACCCTCTTGCCCGTGGCGCAGCTCTTGCGGAAAAAGGTTAATGAGTTATATCAGATTATACGCGATACCTGTTTTAATACGTTAATTCTCGGAGAGAAAAGAAAAGAAAGGGAGATATGGAGAATTGGCTCTCTCTCTCTCTCTCTCTCTCTCTCTCTCTCTCTCTCTCTCTCTCTCAACGTATTTATACTGTACAATCGCATAACCGTCGAGATGAAAACACACATTTATATACATATATATATATATATATATATATATATATATATATATATATATATATATATATATACATACACACATATATATAAAAAAGCGAAAACCACAGAAAAATAACAGACAGAAGACCCTGTGGTTTTTGCCTATTCTAAATCACATGCATCTTCTGTAATTTTTTAAGCATAGCACAGATTTATATATCCTACAACCTGTACTAGTATCTAGTGATGAAGGATTGGCGTTAAAGAACCAAGTAACTCATGTATCAGCTAGAAGACTTCCGTTAATAGCTAACACAAACAGTACACCGATCCCTGTTGAGCTTCTTCCTCTTAGCATATTCTCCTTGACAGACTGCTGTGTACTATTAACTTTAATGAAAAGTGACGTGGTATGAAAGACTGAGTGTACAATGACAGTTCATTGTATATATATATATATATATTATATATATATATATATATATATATATATATATATATATATATATATATATATATATATCATTTGTCTTTTCACTGCCAAAGGCTGTAGTCACAATGCTGCTTGGCTGCAAATGCCTCACAAACCGAAAATTCAAGATTCACTAATACAGCTGTTGCTGACTGCCTCTTTCGAAAACCTCACTGCTTAGTGATTATAATTTCTCTTGTCTTGTGAGAACCATACATGCCTTGGAGTGGCCATGTTTTGTATAACCATGCAGGTACACGATGTTAATGCAATTTGATGCTACGTATTCCCCATTTTTGTGCTTTAAAACTCTCTGAGTGATAGCAAGTTCCCAGAGAGTTGGGTATCTGGTGTTGTGCCATAATCTGTTGATAATACTTTTCTGTGTTTTCCGATACAGGCTTTATCAGTCTGTAGGGAACTTCGTCAGCGCTAGGGGCTGTGTCAATGCATGTTGTTAGTGTTGAAATAACTTTGTCCTAAGTTTATATTGGGATTTTGGGGAAGCTTCTGTTTTTTAAGATACACTTGAAAAGTTAAGTTAAATTTTTATTACCAAGGAGTTTGTGGCCTAAAGACTAAACACTTCTAACACTGGTAAAGGATTAAGCGTAAATTTTCGTGCTATTTCTTTCGTCTTCTTCCAAACAGTTGAAGATGGGGCCCTGCTATTAAAGAATGGAACAAAACAGATGTCCAAGATTGATGTCTAGGTGCTTTTAAAGCTGTGTAAAGCTGGATTCTGCATTTTTTAAATTTATAATTAAATTTTCTTAGATACGATGTCTGGGACATTGAAAATGGTCATCGTCTAAATAAATCTGTAGCTTTTGGAACCGATGGGATCATCTATAATTTGGAATTTATCATGCTTCACTATATTTTTACACTAAATGCAACTCGATTTCTAAAGCTTTCCAATGCACCGCCGCCGTGATCTTTGTGGTGGGGGTTATCATTAGTGTAAAAAATGACTGAGGAGTGGTCACTTGTAAGCCCTATGCCAATTGTACAGTACACACCAACTAAAAATCTACCCGGCAATTTGAGTTTGGGGTCAATGAATGGCAAGGTGCCTGTCTGAACGTGGAATGTTTTTTTTTTTTTTTTGGCTGTGTTTAAGAGTCCAACTTCTTCATGCCCTATTAAAGATGCTATGAGCAGCTCTTGTATCAGCCAAAACATCCTTCCCCCCGACAAGAGATGCCTACTACTGATGTCCCAAAATAATGCAAATGGTTGGGGTAGCTGTTTAAAGTTTGTCTTACTACTAAATTCTCTTGTGAGATGATGTTTTTATTTCCTTAGCAAGTATGTATGCATACATGCATACATACATACATATATATATATATAATAGTATATATATATTATACAGTATATAAATATATAAATAATTATATATATATACATATCTATATATGTATATATTATATATATATATATATATATATTTATGTATATCATATATATATATATATATACATATTTATATATATATATATATAAGTATATATATACATATATATATATATTTATATATATATATATATATTTAAATATAAATATTAATTAAATACAAATCGTGGTACCTTCATTCCGAAAGCTCTGTCACACAACCTACTCAGCAACAGAAAGGTACACAAGTAAATCACAGTCTCACTGTAGATCGTCAAAAGAAAAAAAGAAATGAAGAAAAGAATAAAAAATGAAAACAAAAAAAATCAAACCGAAATATGCCACCGGTTAACGAAGTGTGTTGTATAATCTTACATAACAACCATCCTTTTAACAATGGTGGCCCTGGTAAGGTTTCGGCGGGGCTATCGCGTTACACAGGGAAGAGAAATGCTTAAATAGTCTCTCCACTCGAGAGAAATAGTTCACTACGAGAGTCAGGGCGGCTGGAAACGTGAGAGCATCGAACTTCTTGGTTAATACGTCGGCGTAAAACTAGTAATGTCAGGTGGCAATTAAAAGTGAAGACATGATATTAAGTGAACAGTTCCTTTATCATTTATTTCCACGTATGTTTGCTTATATGTATGAATGCAAAATAGGAAAACTGATTGGCCAGTTTATTAAGTACACATTACATTAAGTGGCGTTTTTAGAATAAATAAACGAAGTTTAGTAGATAGTCCTGAAATCATTTACTGAATGTATATATGTATGTATGTTTCAATTCAAACAATGTATGTAGTATGTATGTACTTTCACACACACGCACACATATATATATAGTATATATATATACTATATATATACATATATATATATATATATATATATATATATATATATATATATACTCATATATATATATATATATATATATATATATATATATATATATATATATATATATATATATATATATATATATATATATATATATATATATATAATATTTAACACTGAACACCCACCATACAGGAATGCATATTTTCTCCAATAAATTTATTGAGAGCATCAACTAATTACTTATTGTACCGAGAATGAACGAGTATTGAGAGATTCTAATTTCTGAAGTCACATACTAAAAGTAAGAAGAGTGGGTTCTTTTAACAAGTATTTAATGTACCTTATTCAGCATAAAAGGTTTGATTTAAATGTGTTTCTAACTGTTTAATAGCCTATAATTATAACTGAAACCTTGATTTTTCTGTAAAAAAACGTTACTTTCATGATTTTTTTGTCCAAAATGTTACATTAATAATTAGGTGAAAATGTAACACACAGGTTCATACCATTAAGTTATGAGAAACTGTGTATGGTTCTCATTCTTCCGCAGTTAAGAAACGAATGTATAGTTGTGTATGGGTTACAGCCTCATCTTCAGTGCTTTGATAAATGATGTCACCTAACATAGAAACTTCCTCGAGGAAAGATTTAACTAAAAAAATACGTATTATTGATAGCTCTGTTACATAGCACTTAAGTGCAAGGACATGATTGTGTGGCCAACTTGCCATTCCTATAATTCAGCTCTGTTTTTTATTGCAACTGTAGGATTTTCTTGCAGTTCCACCTTCAGATCCTTGTATTTCATTTTATTGATTTTTTGGACCTCTTTACCTTGCTGTCCAACCACTCTAACTCCCCTTTTCATTGTCTTGAGCGCCGAATAGCCAAAAGTGCCACGGTGTTTGGCTTGAGAGCTTAAATTTCATAAACCAGTCGGTTTTATTCTGTACTGAGGACAAGATGGATATCACCTCTTGTCAATACGATATGGGGAGATTGCTCGGAATTGCACCCACAAAAAAAGAATGATTTATAAACTGACGGTGAAGAGAATGCTCGGAGTTGAACCCACACACAAAAAAAGAGACTATAAGTAATTTTACAAGCTGATGATATGAATAGATTGCCCAGAATTTCATTTACACACAAAAAATAAGAGACAAAGCAATTTTATAAGTTGACGTTATTAGGAGATTGCTCGGAATCCACACAAAAAAAAGAGACAGTAATTGTACAGGATTAGGGTATGAGGAATTTGCTCGGAATTGCACCCACACAAAAAAAGAAACAACGTAATTTTACGAGCTGACGATATGAGGGGATTGCTCAGAAACTCATCATCACGAAAAAGAGACAAGATAATTTTACAAGGTGAAGCTATGGGGAGATTGCTCGGAAGTTCACCCACACAAAAAAAAAGAGACAAGATAATTTTACAAGATGAGGACATTGGGAAATTGCCCGGAATTTCACCCACACAAGGAAAGAGACAAGGTAGTTTTACGAGCTGACGGTATGAAGAGATTGCTCGGAATTTCACCTACACACACACACACACACACAAATAAAACAAGGAGACTGGGTAATTTTGCAAGCTGACGATGAGATTGCTTGGAATTGTACCCACAAACACACAAAAGAGACAAAGTAAATTCACAAGTTGACGATATGAGGAGATTACTCGGAATTGCACCCAGGCACAAAAAACAAAAGACGAGCAATTTTACCAGCCGACGTCGACAGCGCAAAACCACAAAATGTTAGTTTTAACGGAGACGACTCGTACTTACGCTTGGAATATTGTCCGAAGAGCAGACGCCTTCGGAGAGCAGTCGGTCTCTGATCTCCCAGGCGAAGATGGAAGGACATTCCCTCTTGAACTGAGAGATCTTGGCTACGACCTCCGCCGTTGCGACGCGGGGTTTGGATCCCCCAATTGCTCTGGGACGGATCGAACCCGTTTCGTAGTATCTGGAATTCCAACAATGGGAAAAAAAAAAAAAATTAAATTCTTCAAAAACAAAAGCAGGTTCTACGAGCGTAAAAGGTAAAAGCAATATTATAAACAAACATTTAATTACGTATTTCAAATAAGATTAGTTATATTCCATGAATAATTAATTCTAAAAGTACATAATAAATATGGCTTAAAAATCAAGCCTTTGTGTTTTATTTTTTGGTTTTAACACCCTACTGGTCATTTCTTTCTACAAAACAAAAATTCTACATTACCAATGATCATTGACCAGTATTTTTCCAGTTTTACATAAATACATAAAGCGTATTGATAAGGCAAGATTCAATTTTTCGCAAACAAATGCTTTGTACCCAGCTTTCTCTGCATTATATGATCTCGAATTTTTATGCCATCAAAAGGCAGAAAAAGTATGTTGTTTTGTATACTTTGCAGTAGTTACAAGCCAACTCGATTTTTTGTTCTGCGTAAAAAAATGCACATACAAGAGTCATTATAACTGAGAATATTCGTGGTGATTTTAGTTAAATTGTGATGCACTTCATATTTATATCAAACCGAATGATTAATTTCTCAGCTTAAAATGGTCGAATTGCAAAGGAAAATTCTTTTTGACATCTAAACAGAATGGTTTTATCGGGATTCAACATTATTTTGAATGTTTATTGCATTCTCATTTATTGTTTTTTTCTCATGCATAATTTAGGTTGAAAATCTGCTAATCTTATTTTCAACAAAAGAAAAATCTTTTTCCAGCATATATCAATTTCTATTGAGGTTTATGCAACCTAATTAACAACACTGTATACTTGAAGTTCTTCAGTCTTACAAAGGTAATCGAAAAGTATTATCATATGCAATTAATTAACCTGTATGAACTAACATCTTCAAGATTCCCCATAGCCTTATCAGTTGTTGTTTGGAGGTAGAAGCATTACATTTAGTAACATAACATATTGGATTTAAAAACAAAACAAAGCAAACAACCTGAGTCATTTTGAAGGTAAACAGATTTGAAGAGTAGAAATGCCATCAGCTTTTGTCAGTTAATTTTACGACAACTAATCCTAAACAAAATATATAATCTGGCAAACTTCAAGAAGCATTTATGTATGAACAGTGTTTCATTATATAAAAATAAACTTCAAAAACAGCTTTCATCTTTTTGGGGTTCACTTATTCTGTTAGATCTCTTACAAGAAAGATATAACCACAACCTTCAGTAACCCAATTTGCCTTCTAAATCTGAGACAATAAGTCGACCTTATATTGAAACTCACATCTCAAACGCAACAGTGCAGCCATATTTGAAGAACAACCTTACCGTGAATATCCTTTTGAACTTCTGTTTTATTCTTCCACACGTTTGTAAATCTAAGGATTTCGATGCTGCAAAGGGTTAACTTTCAAAGGATGCGATTTATGGTCTTGCCAAAATAAATTCCATCAATTTACTCGCCGCGTATCTTTCAAAATCTTTAACAAAAACCTACATGCATATTTACTTGGCTTGAAATATGATTTTTCTTCGGTTTCCGATGCCATGTTAGAAATGAATACCTTATTTAGAAAGTCAAACCACATATAAATGATGAACATCTTGAACTAATCTCGCGCAACTGAGGGCAATATGATGGCATCTTCACCTTCTGAATTAAATTCAGTAAGAATCGAAACCTAGTCTTATAAAAAGTCGAGAGATTTCCCTAAAATTTCTCCAATTTTTCAAATGCCGTACCTCGAAGATAATATTATCTCTCCGTTGCTTTACTGCCTAAGATTATAATAAACTTCTTGAATACACGATAAGAAGTAACCAAGATTACACGCCTATAAATTTTCTTTCAATTTAATATAAACACTATCTTTATAAATCAATTTTCTTAGTCACTTAGGAAGGAATTACAGTTTTATAAGGAATAAATGCCAATTCACATCTGAATTAATGTTCAGCTTATGAAAGAGTATGCCTCCTCATAATATTAGGGATAGTTATGGTAGCCTTTAAAAGATTGCTAGGACAATCTCCCGTGAAGGTTTACTAGCTCACTAGCCCTTACAAGGTAATGTCTTCAATCATGGAATATATACAAGGAACATGTAAACATATATACATGTATTTATATCTGTTTATATGCAGTGCACAAATGCATAACCATAATTCGGTTAACATATAAGAATTTACAGTGCTAAGATCCAATTGAAATTCAACAGAATCGTGAAAATAACCCCCACCCCCTCCCCAAATGAGATATGATGGATCTGAATGTTTCTTACGTGTTGTTTCTATTTTTCCTTTTTAATTTTTAAGATTTTCTCCAATTAGAAGTTACTCAGACCCGTCTGAAAGCAAGTCTGACGATTTGAAAACAACTAGAAATTGTTATTGCTGATTTTTACTGACTGAATTTACTTTTACTTTGACCCTGAAATAAGACCCAAGAGGGGAGTAATATTTTTATAAGATAGTACTTAGCGTTAAGTGACGTAGATAATAAGAAGAGTTAATCGATAATATTAATAAAAAACTATTATAGTATGAAGTACAGCGTTCCACTTTCATAAACAGTTACATTTACTATCTGAGACATGTAACTGCCTATGAAACCATCGACATCTATATTAGAGGTCAGGGCAGAGGACGCGGGTAACTTCCTCATAGTTCTTTCTCTACATGACTTAAAATTCGAGAGAGAGAGAGAATCTGTCCATAAAGTTTAAGTGTATAAGTTTTGGATATAACTAACGGTGCGTTGCCCTAGTGTTAAAGCTAGTATTTACGTAAATCTTCATGAAATAATTGTCGACTATTCACAAAGAACAGAGAAAAATATTTTCATGTTGTGGCCAAAGTACGGATATTTGGAGTATTGTGAATGCTCTCAATGCTCTCTAGAGCCCATCTCATTTCAATCATTTTAATCTAGGTTTAAAAATTGAAACTTCTCATCTTAACCTTCGAGATTAAAGAAAGTAGTCAAGATCTTCACTCATTTTGATAACCCGTTCCCCACCACTCACCTGCCCAGTATTTTGGAGACGCAGCCGTTAGAGACCTGTAGAATCCTGGAGATGTCGCAAGGCCTGGCCCCCGAGTGTGCCAACTCGATGATCTTCTGCCTCGTGGTATCGGGCAGGGGGCGCCCAGACACGAAAACGCCTCCGAGTTGGTTCACCCCGCTGTGACCTGGAGTGTCAAAGAAGAAGAAGAATAAATAAAAAAAATACTCTTCGTTACGAGTAGTCAAAGAAGAAAAGGAATAGATAAACAAAACAAAAAAAAATACTTTTCGTTATCAGTAGTTAAAGAAGTAGAATGAATTTACAAAAAATACTTTTCGTTACGAGTGGTTCAAAAAGAAGAACACTAAAAAATAAAAAAGGCTTTTCGTTACGAGTGGTTCAAAAAGAAGGAGAAGAAGAAGAAGAAAAGACTTTTCGTTACGGGAGGTACAAAAAGAAAAAGAATAAAAATTAAAAAAAGACTTTTCGTTACGAGTGGTTCAAAAAGAAGAAGAATGAAAAACAAAAAAATACTTTTCGCTACGAGTGGTTCAAAAAGAAGAATAATAAATAAAAAAAAATACTCTTCGTTACAAGTGATTAAAGAAGAAGAATAAATTTACAAAAAATAATTTTCGTTGCGATTGGCTCAAAATGAAGAAGAATAAATAAATAAAAAAATACTCTTCGTTACGAGTAGTTAAAGATGAAGAATAAATTTGCCAAAATACTTTTCGTTAAGAGTAATTCAAAAAGAAGAATAAACAAAAATGCTTTTCCCTACGAGTAGTCACAGGAGAAGAAGAATAAATAAAAAAAAAGCCCTCGTTTCGAGGAGTCAAAGAAGAAGAAGAATAAATAAAAAAATACTCTATATTATGAGTAGACAAAGAAGAACAATAAATTGAAAAAATGCTCTTCGTTACGAGTGGTCAAAGAAGAAGAATAAATAAAAAAACACTACTCTTCGTTACGAGTGATTAAAGAAGAAGAAGAATAAATAAACAAAAAGGTGCCCTTTGTTACGAGTAGCTTGGCTGTGAAAATAAGAACAAAAATGTACATCAGAATCAGTGGTCAATAAAAAATTAAACATACAAATAAAAGTTAGTGCACTGGGTTTCTTGGCTTTTGAAGATACCGTAAAAACACACACATAAAGGCATCAGCCATACATTTGGTACATTTGGTTGGTTATAAATTCCAACTGAAAAAGTTTAGCTATTTGGATTCGTATCACTGACGTAATTAATAGACGCCACTTAATGAGGGCAATGATCTTGTCATTGTTAATGGTGATGCCTTTCTCATTCATTCAGAGTTCCATTAAATAACATGACGTAGTGAGACACACGGAATCCTAATCAGCACATGCTCATGTATCACTCACTGAAAGTGATACATTGCCTACTGAATTGATAAGTGCGATTCAGACGACACTTTAAATCTGAGAAGAAACAGGAGGGCAGAAAATAGAGATATGGATCTAAAACCTCAATGAAAAAGGAAACATGGTCTTTAACAGTAAATTAAGTAAACTTTAAAGAACGTTACGCGTGTCCAGTAACAACAGGTCGCATTTAATGCTCTTTTGAGGCAGGACAATAATTTCCCTCCCATAGTGGCATCTGCAGTTTCGTCATACAAGACACATCACATCAGCCGTAACACGCGGTTGGCGATCATTTCCACCGGCGACCCAAGATTCCTTTTCATCTATTAAATCCTTCAGATGTCCTCAAGTTTAATTGCAACAGCAGAGAAAAAACAATGGAAGAACCCCGGTAACTGATTCGACACTGTTTAACGAAGATAATTTGCTCAAAGGGCTACTTATTAAAGATTTCTTTGCGATGTAAATAGTAGGACTCTTAAGGGAAAAATTGAAGGGAAAGTTTTTATGAATAGATTAATAAAATTCGTCCTCCATAGTCTTCCATAACAACCATATAAATATAAATAAATATATATATTATATATAATATATATATATATATATATATATATATATATATATATATATATATATATATATATATATATATATATATATATATATATATATAAATAATTTAAATAAAAGCAGTCACCTCATTTTCAATCGTTAATTCAAAAGGCTTTGAAGACTACAGTAAGCTGCTGTAGCTCCTTTCGAGTTTTCCGCTGAAAAATATGCGTGAATACATTCCTGTTCTTCAGTGAGAATTATCGCATTCAAATTGGGGAACAAATAGGGCGGTAGAGAATCCCCCATTATGGAAGCATAGTTATGAAGAACTTCATCAGCTCATTTGAAATTTGAGGCCGTCACTTTCGCGGTGAAGACAGACACTTAAATTATCGATTTCTTTATACTTTTGGAAACGATTTCCGATGGAAGCCTAGAAAACTGAGATGGGAAAATGTCTCCTTACCAACGCTAAGACTAGACTTAGGTCAGAGGGGCAATAAGTTCAGTGTTTTATCCCCTACGCAATAATGGAGAGAAAAAAAAATTGGTCACAGGTAATCCTTATTCATGCCTGTGCTGAAGTAGACCAATTCTCACTCTGGTTAAGCGATTATTGAAGGTGAACAGTATATATATATATATATATATATATATATATATATATATATATATATATATATATATATATATATATATATATAAAGAGAGAAAGAGTCGTGCATGTGTGCTTGCTTAGCTAATTTCCAATCAAAATTCACGTCTAACTCGTTTTTGTGAGCTGAATCCATTTGCTCCGAAAAAAAATATTGCGTTAGAAATTAAATAAAGCACTGACGTTACACGAACAAGTGGTAAGATTTAGCTTTATTGTTAAAAATAACATGGCACGAAAAGAATAATGCGGTGTATATTGTTGGAAACCATGAGACAATCACCCATCTGCCATCAGCAGCAAACCACTGACAACTGCAATTTTTTCCTTGTTTTGGTAGATAGAAATAAAGTCATTATGATTCCTTTCTTACAGAACACCTTATAGCAGTGTATTTCTGGTAGATGAAATGCCATTCCATTAATCTAAAACCTTTGTTTGGACTGACATCTCCAATTACTACTGTCTATGTTTATCATTTTTGGCTAGTGATGGGAACCCTTTAACCTTATCTACCCTGCCGATGGTAATTGTCCTTTCGACGGGAGACTTTGGTTCTTTCAAAATCTCTCTAACTCTAATTTTGGATGGAATAAGTCATATTTGCGCTACTGTGTGAAAGAAGAGGCTAAACGTTAAATGACATTTTGATAAGCCGTAAAAAATGTGAAGCAAAGTCATTTCGAAATCATGTCTCTGAAAATGATCGTCATGATTTCTTATCGTTAATAGCCATTCCTGATACGGGACAAACAATTTGGAAATAGGCCCACAGGCATTACTAGGGGGTGTTCGCAGCATCCCTTCGGCCCCTAGCTGCACCCTTTTTCTTATCCTTTTACTTCACCTCCATTCCCTATTCCTTTCTTCAAGCTTGTTCTCCAACCTCTCTAACCATTACTCCTTAGCGCAACTATGGGGTTTTATCCCAGTTCCACCTTTAAATCCTTGTATTTCATCTCCTTTACTTTCTGGATTTCTTTATCTTGCTGTCCAACCACTCTAACTCCCTCTTTGCACTATCTACGGCGATAATGCCCCAGTTCTTGGTGTGACAGCCTAAATTTCATAAAATCAAATCAATTTGGGCATAGTTTAGTTTCCTGTTCTTTCCTTTTATCTGCAATGATGTCTGTAACCAAAACAGCACACTATACCATGGAATAGAAAAAAAAAATGCTGACGAAAGTGAACGATGGAATACAATATCATAAAACTATGCAAGGAGAAAAGGTCCTCTTTTCATTAAGTCATTTGGTTTCATTCAGCCCTTTCCTCAGTCGAATTCAATCAGTCCCTGGAAATATCAGATGGAAAGACTTGTAAAAATACTAGCAAGTTGTGCAAGCCAAACGTAACACCCATCCCTCTTCCCCACAGTTACATTCCATTACAAAATATGCTGCTTACAGTAATGTGAGATTATTGCAGAATTATATATTATGGTCATTCTGTCAAAATCGCTTTCACCTCCTTACAACAAACAAACCATGTCCCTAAAAGATAATAATAATAATAATAATAATAATAATAATAATAATAATAATAATAATAATAATAATAATAATAATAATAATAATAATTATTATTATTATTATTATTATTATTATTTCAAAATATAAAAACTGAAGTATATCTTTCTATCCTTACGAAGGACGCGAGGGTAGCTAAGGGATCAAGATGTAAGGCCTCTTCACGGTCGCCCCGGAAGTCCATCATTAATGATCTCATTGTAATTTGGCCTTTAGGATTATAATATTCCTAATCTCTGCTCACTTCCCCCACACTCCTATTGTAATCCCACTCTCAAGAAAGAGGACAGAACCCCGCCCCCTTCCCCCCCTCATCCCTTCAACAGAAGTTCTCTCTCATGGCCGGCTAAAGAAGTGCTGGTGTGTCTCGAACTGCACTTCACGGCGTTTCGAGGGGGGGAAGGGGAGCTGGGGGAAGGTTGGAGAGGAAGGAAAAACCTCCTTGGAAAATGCCCTTCCCAAAGCCTAAACCTCTAAAGGCTCTAACTCACTAACTATTACCCACAATCCTCTCCTATCGTGGGGACCTTAAATGTGGCTCTCAAGGCTAAGCCTTGTACCTGCTGCCCTTCGGGCGTTCTCGAGCGTGTGTGTGGTGTGTGTGTGTGTGTGCACGTGCATGTTTGGCGTGTGTGGGCGCGGGCAGGTGTCTGGTAGTTATCGTACATTAGAATATTAAAGATAATCCCGATACACATAAGATCGTAATTTTATCTTCCACTTGAGAAAAGATCCATCCCTTCTATTGTATCTTCAATTCACAGTGGTAAGTTTAACATATATACTACTATACTGATATATATGTATATATAACTGCAAAACTATATATATATATATTTAATATAATATATACAGTAAATATTGCTGTTCGCCCTTGATGTATTTATGAGTAATGAATCAGATGAAGATTTTTCATCACCAAATATAACGATACATACAGCGTCCCCATTAGGTATTTTTCCATGTATTATGCTACGTGGTCTTTATGTGTTGACAAAAATTTCAGACCGGGGGCATCAGTGCTACCTGTTTTGATGGTCAAGATTAAACTATGGGGAGAGCAGACGTTTTTCCTTTTCACAATCCTTTTTGTCTATGTTACTAAGTAAGTTTAATCCCCAGAACTTCGATTGATTGATCTAACCTTTGTATATCTGGATTCATCCTTCGTCAAAGTGCTTTATCTTTCAATACTTTATCTTCCACTTTATTTCAGTTTCCCTTGTTTCATTCTCCAGCCACCATTTTACGGTAGATGCTTTTGCGAAGTCTAGGTCGCCAAATTATTATATTGTCTAAGCTTCATCCAAATTATTCCAGTAAGACTAAGGGTTAGAGGTAAGCAAAGTATAATTCTTTATGCATGCTTTGCGTCTCGAATCCATTGTTTGGAAGAGCCGCTGGGATTTTGTATTTAATACCATCTTAACAGGTAGAGATTTTTCTGCGTCAAGTAGTTGGCGGCGTTTATCATATAGTCTTTATTCCTTGAGATATTCTCTGTTAAGGTTAATTACCCGTAACTGGCGATTGGTTAATATAAACAACTGTCTTTGAGGTTAATTCGTATATGACGGGTTATACGTGTGTCCTTAGCATGCAGGGCCGTGTAAAAATTATGTAAATCAAGTAATAAACTGATCAGCCAAGACATAATAATATATATATATATATATATATATATATATATATATATATATATATATATATATATATATATATATATATATATATATATATATATAGTTATGATTCAAGTATCAAGAATGGTTACGTTAAATGTGAAAGGCACATAAATTGGTCGTTCCAATTTTCTTCCCCCAACTGTAAAATGTCATTACCTAAACTTGCCATAAACGAGCGAGGAAGGAGAGGGGAAAATATTTCATTCAAGATTAAATTACAAAACTGATTTTTCCCCGAGAGAGGCGAACACGTAGCCTTTTTTCAGTTTATCAAGATCAGTTTCTTTTTCTTCTTTTTGTTCTTCTTTGTTTCGTGTTTTTTGTAGTCACTTGCATATCGGTCATTGTCTTGTTTGGAATTGGCATTGGCACTGAGAATTTAATTCCCTGAATGCAGTAAATGCGCATCGCATTCTATAAATAGGATTTAAAAACTAAGAACTCATGGGAATCGAACACCTGACTCGTGGTTTGAAATATAAAATTTAAAAAAAAGTAATTTTCTCTAAGGCAATAACTTGTGACTCCCGTAACGTAAGTCACTCTTCCTGTCATCTGCAAACTTGTACGTAAGCACACACACAAACGAGCAGACGCGCAAACACATGCTCGGACATTAAATCTGGAAAAAAAAGTCTTAAAACCCAATGTTACGGAACAGACATTAACTTTTGGCTTTGGAAAGAATCTCTTTCTGACGAGCGATAATATCCGATCGTATTGAAACAGATGCCATGATTCGAGTCCTGTGACACTCATATTGCTAATCAAATCAGTTTCAAGCTTTAATATAACTTAAGTATGTATAACCTACAGGCACTTAAACTTGTAAGTACAAATCAACTGCTTCAACTCCTTTTATTAGCCAGGGCAGCGTTTTACGTATCAGTAATTTGACTCTTACTGCAATAACAATGCTCGCAAAGGTTACTTGAAGAGATATTTTCAAGCCTACATGGAAGGACGTTGAATATATTTGTGTGTTTCCAGTAAATATCCAAAAAAGATCAGACACTTGCGTTGTTCTTTTGCAGTATCAATTTTTATTCATGGCGACTTTGACATAGCGGTTCAGATTTGCTCAAAAAGATTCGAGAGCTTCCCCTCCCTAAAACACAGGTGCCTGAAACAGAAGGCTGGAGAAACATCTGCCTTTTATGCGATGCGCGATATACATGTCCCTCACAGGCGCACATGTTACTTGTTCCTTTAAGCACATTAGAACAGAGATCTTCGTTTTTCATACTTACAACGACGCCTACGATGCAAAAGATGAAATCCTGAGCGAGAATAATGAAATTGTTAAGATGAACGACGACATGCCATAACGTTTATAAAGATGCTGCTGGCAGGAGCTTGTAGGAACCGATATAGTCTTGAATAACCACTACTTTGCCGTGATTGTTACAAACACTTATTCGACCACATCAAGATGAACGATTTGATTAAAAGCAATCAGACGTTATCGCTAAATTATTATCTTTTTCACAACAGGTTATATAATAATAATAATAATAATAATAATAATAATAATAATAATAATAATTATTATTATTATTATTATTATTATTATTATTTTTATTATTATTAATATAATATATATAACAAACAACTCCTCTTCTAAACAAGCTATATTAAACGGTAAACAATTCACATGTGCTGAGCTTATTCAAAATCGGTCTCACCACCTGGGAAAGCTCAGCGGATATCAACTAATAATTCTTTTGTTACCATCACTAACAAAAATGAGGAAAATTTTCACATGGAGCAAGCCCAGGCGACTGCTGCGATCAAATGCCTTAAAAAAAAAAAGTAAAGAACAGTGCTAAAACGACACTTCGTGAAAGTCCGCCACTCAAACAAAAGAAAAAAATGAAAAACCGAAGAAGCGCAGCAGCAAAGGCTCGCCAAAGTTCCCTCCCCACAATCACTTTCCGCGAATCACCTGCGAATGAGTCTCGGGGTTATTCTCCCAGCGGGGGACAACCGAGATAGATAACCCCAAACAAACAACAGTGTTCGAACCCCATAATTAGGAAGCTTTCGCAGCTGGGAGCTGCTCATGCGTTCGAAATATAAAGTTTTTTTTTTTTTTTTTAGTTACTTTCTCTCTTAACGCTTACAAGACGCAGAGGAAGGTAAACGTTATTGATTTATTTTTTCTGTCTCAAAACTAGTTAATGATAAACAATTTTTCAGGTAAAAATCAACCTCAACTCTACTGCTTTAAGCTTTTCAAAGCTGTCATTCAATTACTATTTCCTGAAAGCAAAATGATTTAATTTCCTCATAATACGAACATCGAACAATGGTTGCAGAGAAAAAAACATTAAAAAATTCATATAAAAAAGGAAAGTTGTTAATTAATGCTGTAACGAAGAAGGCAGTGTTCCAAAATAAACAACTTTTTGTTGTTATTGTTTTTTAACGGAGTAGGTATAGTTCATTGAACTAGGGCTTTTGCCTGCTGAAAGCTCTTGGGCTTGAAGGTGCTTGAATCTAATATCTCTTTCTCAAAATTAAATTCGATATAGATTATGATATATATCTTTCAGATAAAAAGGTCGAATAGCATTGGTATACACTATACACACATGTATTTATATATAAATAATATATATATATATATATATATATATATATATATATATATATATATATATATATATATATATATATATATATATGTATATATATAAACATATATAAATATATATATATATATATATATATATATATATATATATATATATATATATATATATATATATATATATATATATATATATATATATATATATATATATAAAAAAAGGTAGGAAAGAAATTGAGTACAAGAAGAGAGTTGAAAACCCCACTGAAAATCAAAATGACCACCGGACGGAGCACTAGTACCTGTGTACAATGTAACAGCGAAACGTCTGTCGCTGCTCTGAGGCAGACGTTCAAGCGCTGGCCAATAACAACGACAGACGCGAGCGAAATCAAACGAATTGTTCAAAGGCATTCGGACAATGAAAAGAGCGTTTATTCCTTCAGTATTACAACCAAAGGTGGCTACTTTTATGTGAATGACGAAACAAAAATAAATGAACCAAGTCTAAAGAAACAAAAACACGCATCTGTTCTTCTGAGCACTTGCGCACTGTCAGACAGCATTCCGCAGTTAATGGTAATATCATGATAGATGCACCTTATGTTTATTTCCAAAGAGTCACCTCTATACATCACTCTGATAAACCTGTTTGTTCATTTTGCCTCTCTGTACTATACTGATAAACACTATCGTGTTCAGATAAAAACTAAAAATGCAGCTAACTATTTTCTTTTAAAAGAATACACTTGCATCAAAAATATATAGGATTGCAAGTATGCACACCTCTTCGTCTTACATTCAAGTCTACATATGGAATTTGAGAAGCGTTTAAGATCTTTGGGATTTTTTTTACTTGATGAATTCCCATCGCGTTTTCACAAGGTTCATGGATCAGGAAGTGGTGTAGTTTGCAAGGTATGACTCAAATTCTCATGTATCACTAAGTGGTGTAGTTAGTGGGGTATGACTCAGATGTTCATAGATCAACAAGTGGTGTAGTTAGTGAAGTATGACTCAAATATTTATGGATCAGCAAGTGTTGTAGTTAGTGGAGTATAACTCAAATGTTCATGTATCAACAAGTGGTTTAAGTTAGTGGGGTATATGACTCAAATGTTCATGGATCAAAAGTGGTGTAGTTAGTGGGGTATAGGACTCAAATATTCATGGATCAACAAGTGGTGTAGTTAGTGAGGTATAACTCAAATGTTCACCGATCAACAAGTGGTGTAGTTAATGGGGTATGACTCAGTGTTCATGGATCAACAAGTGGAGTAAATAGTTATGACTTAAATGTTCATGGATCAACAAGTGGTGTAGATAGTGAGGTATGACTTAAATGTTCATGGATCAACAAGTGGTGTAGATAGTGAGGTATGACTTAAATGTTCATGGATTAACAAGTGGTGTAGATAGTGAGGTATGATTTAAATGTTCATGGATCAACAAGTGGTATAGTTGGTGATGTATGACTCAAATGCCCATGGATCAATAAGTGGTGTAGTTAGTGAGGTATGACTCAAGTGTCCATGGATCAACAAGTGGTGTAGTTAGTAGAGTATGGCTCAAATGTTCATGGATCAACAAGTGGTGTAGTTAGTGGGGTATGACTCAAATGTTCATGGATCAACAAGTAGTGTAGTTAGTGGAGTATGACTCAAATGTTCATGGATCAACAAGTGGTGTAGTTACTGGAGTATGACTCAAATATTCATGGATCAACAAGTGGTGTAGTTAGTGGAGTATGACTCAAATGTTTATGGATCAAAAGGTGGTGTAGTCACTGAAGTATGACTCAAATGTTCATGGTTCAACAAGTGGTGTAATTAGTGAAGTATGACTCAAATGTTCATGGATCAACAAGTGGTGTAGTTATTGGAGTATGACTCAGATGTTCATGGATCAACAAGTGGTGTAGTTAGTGAGGTATGACTCAAATATCCATGGATCGATAGGTGGTGTAGTATTCGAGCGAACATCTTTCGTATGCTAATATACCTGATCCAGTAGCAACTCTTACAGTTAACGCCAGTGTTTCAGCTGGTCGTGTGCACTAATGCTCAGTTATTGGTATTGAATAATCTTACTTCTAATAAAGTGTTTCATGTTGGTTTAACATACAGACAAGGGGTCGTTGGGTGTCAGAACCGGGTGTTTGTATTGTTTCACCCGGGCTTATTCGTAATATCAGCAGTTTTCATGTTGACCAGGATATGTCATTGCTTCAGACCACGTCCCTATTTCTTTTGCATTACAGTCTTTAAAAGTGAGAAAGGACTCTTTACTATTACGTGCATCTCAACTGGGTGTTCATGCTGAATTGTATTCTAATACTAATCATAATAGTTACGTTAAAAGGTCAGTTAAGTATGAGTATACCAATGAAAGTGCATTTTTGGAATCTTTGTCTCAATACCAGTTACCGGAGTTTATGGATAACAGACAATTGTACAGGGAAAGTCAGCGAAATTTTGTATAAATGGTCTTAGATGAGTACGATTTCGTGATGCCAATGTGAATTTCGATATATCTGTAGACAGATGTGAAAGGTTATTGCAAGCCAAGCAATAAATTGGAAGGGTGTTACTTGACACACGAGACAGCGATAGTAGTGTTCCCACGGATCAGCAGTTAAAGAGTTTTTCGAGGAGACACTTAACCAGTAAATGTTCAGGAAAGAGACTTTGCTGAGCTGGACACTAATGTATCATTTCCAGTCTTAGATGACCCGATTACAGCTTGCGAAGTGTCTCAACAGGTAACGAAAATGAAATCTGATAAAACATGTGGTCCAGATGTCTTAACTCCTGACAGTCTGAGGTTTTACCTATAACTTGGATTGTGACAATTGCATCTCGTTTTAATTGTATTTTTGTATCAAGACGTTGCCCCTTCACAGTGAACTTTTGCTCGACTTTTTGTCATTTTTAAACAAGGTTACCGATCATTGGTTCATGGTTATCGAGAAGTAAGAGTTATTAACTGTTTAGCTAAGCTATGTGATATGCACGGAGCATACTGCGTCGCCTTCGTCTATTGGGACTTAGCCAAGAGGAAAAAGTGGAAAGTATTTGTAACTTTTGTCGATTTTTCTCAAGCATGTCATAAAGTTCCAAGGAATGTGCTGTTTACTGTGTTGAAGACACTTGGATGTGGGGTCTTAGTGTTATTTGCTTTGATACCTATCTATCGAGATACACATAGTGCAATTGGCACAGCTGCTGTGAGTACCACAATAGGTGTGCGTAGGGTTCTGCTACATCTTGTCTGTTATTAGTCCTATTTGTTAACGACGTTATTGATATTATCAAACAGAACTGTGGTGTAGATTGGGTTCTTAACATGGCTTCATGTTAAGAATGTACTCATTCTTATGGACGATACTGTTATTTTATCTACTACAAGAACGATTATGGTTAAAAAGATTGATTTTTTAAACCAGTTTTGTGAGTCACATGGTATGGTAGTTAATGTAAAAAAACAAAATTCTTTGTTATTGGTGGTAATGAGACTGATAAACAACAGGAAGTTCGGGTCAATGCCATTATCGTCGAGCCCTGCGGTCACTAAGTGTATCTAGGTATTCCTTTTACATCTGATGGATCCATTTCCAGAGCTATTAGAATGCCCGCTCAACTGAAAATGTGTCATGCATTAAAATTTGTATCATGGGTCAACAAGTGGTGTAATTAGTGAGGTGTGACTCAAATGTCCTTGGATCAACAAGTGGTGTAGTTAGTGCGGTATGGCTCAAATGTTCATGGATCATCAAGTGGTGTAGTTAGTGAGGTATGACTCAAATGTTCATGGATCAACAAGTGACGTTGCCAGTGGGGTACGACTCCAACGTTCACGGATCAATAAGTGGCGTAGTTATATGACTCAAATGTTCACGGATCAGCAAGTGGGGTATGACTCAAATGTTCACGGATCAATAAGTTGTGTAATTAGTGAGGTACGACACAAACACCCATGGATCAGTAAGTAGCATAGTATGTATGTGTGTGTGTGTGTGTGTGGCATTTTACACAATTTGTCATGGATCAGCAATCGTTTTAGTTAGCGAGTAACAAGTCAATGTTTATCTGTCAGTAAGTTAGAACAATTGAAGTGGTTAACTAGTAAATGAAATCGGGAGAAAATACGCCAGGTGACGGTGAGATGAGGGAAGAGGTGAGTCACGGAATAGGAAAACTGGGGACTGCAACACATGTGCAGACGACCAGGAAGAGGCAAAACACTCACGGAAGCCCAGTTGGGAATGACCGTAGGAACTGCTAAAATTAACTCTTCTTTATGTAGATGGAGTGTGGAAGGTGAATGCAAATGAAGAAAAAATGGCTGAAGCTCTGGAGACTAACTCTTTGCATATTATAAGTAGCATAAGACCTGAAAGGCTGAGAAATGTGGAGGTACAGAGAAGTGACAAAACGTTAGTGTAACTGAAAGGATGGATCATGGTTTTAAAATAGTTTGGTCATGTGGGAAGCATGGATGATGATAGGTTGGCGAGAATAGCATTTAATTCTGGTGTAGGAAAGAAGGAGGACGAAAGGAAGACACTGAATGTGCCAGATGGATGGCATGAAAGAGGTATGTACCAGGAAGTTCAAGAAAAATAAATGGCGCAATGTGTATATGAGGTTTGAAGTGATGTTAATGAATGTTCTGTGCGCATCTACAAAGCAGCTATAATTGCGCAAGTTTTCTGTTCAGGAGGGTCGACAATTTAGAACTGACCGTTGCGCTGATATTTTTCTCTGGCTCCCCCTTGTAAGGACAACGGAATAATAATAATAATAATAATAATAATAATAATAATAATAATAATAATAATAATAATAGTTTGCTCTCTTCCTCTCGTTTTATAAGTTGGATACAAGAAATTGGGAAGAGCGATTGAGTAGATATGTAAGTTTTCTTAGAGAGAGAGAGAGAGAGAGAGAGAGAGGAATAAAAGTCCTTAGATCATACATTATCATTATCAAGTGAAGTGGTAAAAAGAACTTTAAAAGGGCAATCGAGACTAAAACGATGGATTATCATCAGGAGCAAAGGATTAGGAAGAGCAGGTGGCATGTAGATTATCAAAATATCACAAACACCTTAATTTTCTTGACTGCTTAAACTGTGACATCCCTCCAACATTAGCATTTAACTTTATATTCTTATATTTCTCTCCATATTATTCTTCTATTTTAAACTCAAAGCAATATGTTTTCTGGATTTTGGCATCTTGAGTCATCGGTTCGGATCTCCCTTAAACGTGAGAAAAGGGCACAGCCAGGGTTTGTGGTTATCTCTGCGGAGAAACTTTTCTTTATTATCATTGTTATTCAGAAAATGAACCCTATTCATATGGAACAAGCCCACAGGGGCCACTGACTTGAAAATCAAGCTCCCAAAGAACATGGTTTCATTGGGAAGAAGTAACAGAAAGTTATCTAGACTGGCCGCGTTTTGTCAGTATGCAGAATTAATAAACGGACGGAATTTACAGGATCTGGATCAGAATACAGATTTTTTTTTAAACGAACGGTATTTACAGGATCCGGATCAGAATTCAGACTTTTTAAAAAACGAATGGAATTTACAGGATCCGGATCAGAATTCAGACTTTTTAAAAAACGAATGGAATTTACAGGATCCCGATCTGAACCCAGACTTTAAAAAACGGATGGAATTTACAGGATCCGGATCAGAATCTAGATTTTTTTTTAATCCCCTCGTTCATTGTTATTATCACTGATGTGTATCTAGAGGAATCGACAGCTTCCTGATGTACGTAAAAGAAAACTGAATGTCCCAGACTGTTCTGTTTGCATATACGAGATCGTAAAAGAAAACGTAAATGATGAAGGCTTTTGGGTAACTATCCTAAACTTGACGTGGGTTTACAGTTTCTGATTACCCTTTCTAGTACGTCACATGTAGGGACAGAAAAAGGATGTGAGTTTGAGGCCTGCAACTTCACTCGTCGTAGATAATTTGCTTGTAACAGACAGAAGATCTCACTTTTTGGAAATATCCCTTCAGAAGGAAACGAGTAATAATAAAAGATAATGAAAATAATGTAAAAAGATTTACAATGGCCTACGAAAAGAAAGGAAGGAATAAAACGAAAAAAAACGGAATATATTAAACTTCATTGCATTTTCCTTGCTCGACTGTTTTCGACTTTTAATCCAAACAACAAAGACTAACAAAGTAACTAAAAATACAAAGAACATTGCGTATGTGCAAACTGTGTATATACCCATGACAAACAAAAGGCGAATAGGATACGTTGGCGACAAGCAAACATCGACAATAAAAAACTGACGCTAATTGTTTTTGTTTTCATTTTTCTCTTTTTGAAAATAGATTAAAGGACACAATGAATTCAGAGCGTGCTAACAAGACAAACCGACAAACAGACAGACAGAGAGACAGACAGACAGACAGAGACAGAAAGGATACGAAAATAAGCTCGAATTAATAAGAATCTGAATAGATATACAGTATATGTCCACGTCACACTTATTCCCTCTCCTAGCCACTCCCTTCTTCCCCCTTGGACTTATTTTTCGATCGGTATCACTAAATTTCGCTGTAATTTCCCACATGGAAATGGAGTTGCAGCTTCAGAAACGAAAACTGTTTTCAAATGAACAATTATTCATAACATAACGTCCTGATCATGTGTTTTAACTTTCATGGTTAAATGACCGGCGTTTGTTAGCTCTCAAATAGCAGTTGATATACAATTTATCGTTGGAGTAATCGGCAACATAGGTGAGTGTTGCCACACGTGTGTGCTGCATCTCATCCCTATTACGTTCTCGTATTTTTGTTTCCAGATTTTAAAAAAAAATATTGTATTTCGTAAGACTTTTCATCCCCATGCAATTCAATAACTTCAGGAGATGCACGAAAAGCCATGCATAACTTCCATAGAAATTTGTATGAGAAAGCAATGCGTTGTAGGATGATCTTGTTGGTACATGTTGCTCTTTTAAGTGTTAAACTTTGTCTCCTTGAATACAAACTTCATTGTTAACTAACGAAACTAAATTTCTTTCCCCATCATATTACGTACACAAGAAAGCATTTAAAAGCTATATATAGATCCAAACGTCATTACCTCTCTCTCTCTCTCTCTCTCTCTCTCTCTCTCTCTCTCTCTCTCTCTCTCTCTCTCTCTCTCTCTCTCTCTCCCCGCAGAATTCATAGAAAGAATGGCCTTCAATTACAATATTCAGTTCTCTCAATAACTCTTATATTTTAAGCTTTTGAAGGTTAAAACACGCACAGGCATTTTACAAGAGAGAACGAAGGAAAAAAAAAACATGAAATAAGTAACTATAATATAACCATATACAGTATATATGTACACACACATATAATCGATATGTAATGTGTGTATGTATATATGTATATAATCACCTTTTGTTATAAGCCATTCCCAAGGTAGGAATTCGATAATAGGTAATTTATATTTGGGTAGAAAATATATATATGTATATATATATATATATATATATATATATATATATATATATATATATATATATAAATATATATATATATATATATATATATATATATATATATATATATATATATATATATATATATATATATATATATATATATATATATCACATATAACAAACGAATACACGTATAACGGTCTCTAAGATAGAACTATTACGTAATTGCATGTCTTATCATGAGTTAAAGTTAGTACTGAAGATTTAAATTTATTTTACACAGGATACGGAAACAACTAATTAAAGTCTGGTGTTTGTTCAAAACTACGTAAAAATTCAAGGATTCAATATAAAAATTGTTCCATTTCATACCTCTCTCTCTCTCTCTCTCTCTCTCTCTCTCTCTCTCTCTCTCTCTCTCTCTCTCTCCTCTTCTTCACATACACGAACGCACAAACACAGCAATTACTTGCATTTTCATTTAGCCAAGCGGTATGCAAATGAGAGCGAGGAAATTCGTGTATGATTTGTATGAGCCACAAAATTAAAAGTATTTTCTCTTGTGTTTGCGTGTGTGTGTGTGTGCGCGCACACGGCTGTGAGTATGTGTGTGTGTGTACGCGCGAGTTTGTGTGTGTGTGCGAATGCTTATGTGCGGATGAGGTCCGCTGGGGAAAAAAAATCATTTGAATTTAGATTGTTCTGGATATCTCAAAATCCATAAACATTTACCATCCATTTCAAATGCAAAAGTCAAATAAAAATTGCCCTGTCAATCAGTGGTTAATGCGGGCGATGATGAATGTACACTTGTTATCGTGTGCCTGTTGTAGTTGTTCCTGTATTTAATCGCTTTATTTTTAATCTGTTAGGAATTAATTCAATTAATTTTGAAAGAGGGTGAAATTCCTCGTTTCTCAGTGGACTTTAGAATTAAATCCGAACACAACGTAAATGGCTTTCAGTAGAGATCTGAAAGGATACTGAATGATTATTTTGCCGCAACTTACTGCGCACGCGTTAGTCAATAACACCGAAATTTTCTTGATGTCTTAATGCTGTTGTAGCCACGGTTATCATTTTCCCGATCGTCGTGATTACACACAAAAGTAACCGTAACTGATGAGACATTTAAAACGTTCGTTCCACGGTATCTATTTTTTTTTCACTGTTCGTGTCCGTGTTTATTTTATTCTGATCCATCTTTATTGTCTATATCACAGTTACGTAGATTTGGGAGACTAGAGTATACCATTTATTGTCCTGTGCTAAAAAATATTAAATAAACATTATTTTGCGTTCGTGGACTCCTTTCATTTTTATCACCTCTTTGTGGAAGAGTAAGAATTAAAATATAATGAAATTTTTATCCGTAATGTAAACGCTTCTTATGTGAATGCCCAATTGGCTACCCCTATGAGAGCTGATAGTCAGCTCAGTGGTCTGGTTAAACTATTTTAATAATATGAGAATGGCTGAAGTTCGAACTGGGCTAAAGTTTCACCAACAGAGAAGTAATTCGATAAGATGAAGGTGACGGCTATAGGCATAACTTTCCCTTGCGTCTTAATATTTATATGTATGCAGTGCTTTAGTAGAATAGAACAGAATATAGAATTTTTGGCCTAAGGCCAGACGCTGAAAGGGAAATAGAGAGTAGGAAGGTTTGAAAGGTGTAACTGGAGTAGGACAGGGTGGAAACTAAGAGGGAAGAAAGACAATATGAACGGAGGTACAGTAAAAAGGAATGAAAGGGTTGTAGCTAGGGCCGATGAGACGCTGCAAAGAACTTTATGTACTACCTACAGTGCACCGCATAAGGTGCACTGACGGCACTACTCCCCTACGGAGGTAATGCAGTAATAATCTTTCAAAACTCATGAAAATATAAAGCAAAGGCCACATTTAGGCAGAAAAAAGAGACTCTTTCTCCATTGCCTTTGTCCATTGGCAGCAAACAACCTGTTCATAGGAATGATGATGATTGTAATGTAAGTATATAGGATCGTTTAAGAAGGCTGCGTGTTCTTCCGACACAGATTGGTCTTTGCTGTGTGAATATAGTAATTTATACAAAACACGTATAATAACGTGAATCAGTTCTTTTGACTTATTTATTTTGCTTTGTTTTCACTAGTTGCTCTACTAAAAACAGGCCTTGGATTCTTTTTAATGTATTTGGCATCCGGCAACTTTAGTGACTTAATAAAGTACAGATAGTGCGCAATGACGTTTTTATTCGCTGATTTAAAGGATCCCGTCATACCGATATTGTGAAAATTCATATGAAATTAGTATACGAAACTGTTTACCTGACACATGAGAATCTCTTAACACTTGCGCAAGTCCTTGATCCTTCGAGGAAGAAATTCTTCTTAGAAAGTAATTAAAACCTCCTCCCCGAAGAAGCATGCCTTTCTTACTACAACGCAAGCACGATAAATAAGCCTGTGAGGCTTAAACAGTAACAAGTATTACAAACAGGACATTTTTAGAAATTCACTGCCCAGTCTAGTAATCTACTAATTTCAAAACTTTGCGGCGAGGAATAGGGACACGCGATTATAAAGGAAAAAGAAAAATAGAAATCGAACCGTCCCCCCCCCGCCTCCCATCCCAAAAAAGGGATAAAATAAAACAATTAAATCACAAAACACGTCAGACCTGCACACCTGTAGTGACATCTGCCTCCCACTTCCATGTCAAGAGTGATTAGATAATGATCTTTTCACGCCACCAGTTATTTTACTCATAATTATAAGAGGATCCTCTTGCTGTCCCAACTTTTTTTTAACAGGTGCAACTGGTGGTAGATCGCTCGACCTAATGGAATTTTCTTGCCCATATCAGAGGGAAAAGTAAAAGGAAAGGTTGGCGGAATGGGCGGGGCTTACCTGTGAAGGAAGCGATAGGCTGTTGTGCCTCTTGGAGACAGGAAGGTTCTAAGAGGCAAGAAAACAATGAGCAGGAAGAGAGTTCCAAACCATGCTAGATGGAACACAGAAAGAGAAACAGATTGGGGGAGTCTGAGGATTACCGATTTCGAAAAATGAGGTGGTTCTAATAACTCAAAGTGGGAAAAAATTTTACAGTCGTTGGCACTGCTTCTGTTGGGTAGTCACGTTCTCATAATGATCGTCAAAATCATTCAGATACACACGACGCGATCATATGATTTCAATGCATGCAACACTTAGGGAGTCTCGTGAACTTGAGAGTAAGTTCCCTTCTCCAGCTGCCAAGATGAGGAGAGTAATTATTATCCTCTCTCCAGCATCTTTATTCACTTTGAAGATATTGCTCTGTTTCGCAAGATTTAATTCTTTATTATTTGATTTTATTACTTTTGCTACAATTTACTTTTAGTCCTTTTTGTTGCTTAATTTAAAAAATATAGGCATATTTACGGTTTGCGTGAATTTTGTGAATGTCGTTAGCAAGATTTGATTCATTAAAGGTTTAAATTTGGTCACCATTGGTTTTTCGTCGGGCTGTAGCATCAGAGCTGCATAATAAGTGGCAGTTAATAATACTGTTAAGTTTATGTGAATGAATATGAATGATCCAGACCAGTCTACTTTTGAGGTACTGTCATTTGTCCGGACTGACAATGACGATGACAGATAGATGTTCACAGTGACAACTCAATTAAGCTCCAGCCTCTGAAAAATATAAGACGAGACATCGTTTGAGATTTATGACGATGATTCGGCTGATTAGAAGCTCTCATTGAAAGCATAACACTCGTACATAAAACTCAATCGCGGGCCATTAAAACAAATAAGAACTTAATTTATATATAATGAAAAAGCCCCTTGGATGTCTTCATGTTCATTAAACTCTTCTCATAGTGTATTTCCCTGTCAGCCAAAAATAACGCAGTAACACTTTTAAGCAGAAAAAGCGCCCACAGAGCCCATACCCCTGCCAACGTCAAAACTCTGCTCAGATATCAAGTCAGACTTTCTTTGCAAATTTTGACGGAAATCTGGTAATCAAGGTCAATGATAATGGTGCGCAGGGAATCCACTCAGCAAATGGTCCCTGCTCCCCAAAGATATATTCCTTTCTCTCCAAAAATGTGATCCCTTGTTGGTAATCATGCGTAAAAATTCGTGCCTGACATTTTGCGAAATGATAAAAAATGAACAAAAAGAATAACCTCCTTGCCAGGAGGCACTTAACGATTTCTGTGAGGTGGAAAGTAATTTTCCACACGAATCTCGCAGGTTTTCACGTGTCAATCAGACGGCAAAAAAAAAAAAAAAAAGCTCCTCACAGATTATCAACTTTCTAGAACAATGTCTCAAATAGTACTTGAAAAAAGGGTAGAGAGTATTAGTGTTACAAAGAAACAGATTTATTTAATGTCCTAAACTTATAATAGGTAGCTCGATTTTCTTTGACGAAGATCGAGGCTTTCGAACGGAAACTATAACAAAGTATATATCAAAAGTTCGGAACAAGAAGCAACCTCGGATGCATTGCATTGAACTATGTCCAGCTTAACCCGTTAGATAAAGAGAGAGGGACATACACTTATCTCCAAATAATTTATGACAGTCATGCGCTTACAATCACATGCTCAGACATATAAAACAAACATATGTTTCCCATAAACATTTGGCCACACAAAATGTATCGACAAAGATAGGAAAACATGGTTCTCCAACTGGTCTAACAACTTGACACGTCGAAGCATATAGTCATCATACTCGTATTCTATTCCCGGAACATCAGATATTCAGGTGTTGTCCATCTGATCGTTTCTTTATATCATTCTCTTAACAGAATGTGGAATTCATTACCTTCTCCAATATTCCCGAGGCCTAATAACCTTAGGGTGTGAGCCCAGGTAACGTTAAATCAGTTAATCAGTCAAAGCTCCTCTTCAGTAAAGGTAAAATTATCGTAGAAGTGGAGCCGTTTCAAAGGTGGCGAATCCTCACTATTAATACTACTACTACTGTTAGCCACATTACTATGTGGACGAACTTTATAAAAATAAAGTGTGAACTCTCTCTCTCTCTCTCTCTCTCTCTCTCTCTCTCTCTCTCTCTCTCTCTCTCTCTCTCTCAATATGTATATATATATATGTGTGTATATATATACATATATATATATGTATGTATATATATACATATATATAAATACATACATACGTATCTAAGCGATGTCAGGCAGAGCAGCTGATCGAGACCACAGGTTTATAAATCAAAAGTCCTAAAGAAGGCATCATGCTTACCCATACAAAAATGGGAATAAAAGCACGTTAAAGAAGAAAAAGATATATATGTATTTATATAGTATATATATGTATATATATACGTGTATATATAAATATACACATACATATATATATATATATATATATATATATATATATATATATATATAATATATATATATATATATATATATATATATATATATATATATATTTATATATATAGGCTACATATATATATATATATATATATATATATATATATATATATATATATATATATATATATATATATATATATATATATATATATATATATATATATATTATATATACATACATACATACATACATATATATATATATATATATATATATATATATATATAAATATATATATATATATATATATATATATATATATATATATATATATATATATATATTCAGTATAGGTCTGTCTTGTAATCCAGGCGTATATTGCAACTCTGAAATCGAAATACTGAAAAATTTACCACATGACTGAAGGATAAGTGCATAAAAACTTATTATTTCTTATAAATATATATATATATATATATATATATATATGTGTGTGTGTGTGTGTGTGTGTATATATATATTATATATATATATATATATATATATATATATATATATATATATATATATATATATATATATACTATATATATACACTGTACACACACACTCACACACTCTCCAGTAGACTCTACAGCATATAAAGTATCCATATATAGATATGTATTTAAGCATGTGTGTTTACACTTGTGTGTCTTAGCACAGATGTATACACAAAAATACAATGACAGAGTCGAGCTTGGTAGTTCGGTGATTTGTGGCTTACTGCTTATGAATAAAATATCACGCTTTGATGAATGACTATGTATGCATATATGCATATATATGTATATATATGTGTGTGTATAAATATGTGTGCGCATATATATTATTTATAAATGAAATATCACGCGTTGATGAATGACTATGTATAATATACTGTACATAATCAGCGCGTGATATTTCATTCATAAATAACTGAATCTGAGTTCTGAACACAAATTCATGAAGTAATACTGTATTAATTAATGCTTCAGTATTAACACATATATATACCCATCAACATTAAATTCACTTTGCCTTTGAAATAACTTACAGCCAAAGCGAACTGTATATGATACATGCATCTACCCTGACCATGATTCACCCATATATCAATCTACAGAGGCACAGGTTCCAATCTTGACATGGGTAGATGCACTTATATATAATTCATCTCGGACATAAGTTATTTCCAGGGTAAATTGAATTCTGTTTTAATGGATTATTCGTGGCCAGTATTTGGTATAATAAAAACATTATTTGTGATATTATCTAATAATCATGTATGCATGTACGTATGTGTGTAGTATCACGTACAGACGTAGCGCAGTTAACAAGCACTAAAAGCATTAACAACAATCTTCAATCCAAAGAGCAGAAGAAAATAGCCCTCTTGCTAGGAAATCCCAAATGAACAGAAACTCTCTCACAGACAAACATAAAACACGAACACCGAACTGTCCCCCCGCCCTCCTCGAGGAAAAAAAAAAAAACAGAAAAAAAAGGTGAACAGAAAATGAATTGTAAAATTCGATACCGTCCTGGGAAATCAGAAATATCAGGTGACATTTAAGTTGATAAAATCAAAGTAACGAAAAAAGGAATAAAAAAAGAAGCAAAAAACAAGCCTCAAAACGTAAGAAGTGACTCTGAAGACGAATAAAAAGAACACATCAAAATAAACAAAAAGCGACACATCAGAGGGAGGCTGAATAATTAACTATTACATCACGTAATCATAAAATCAATGGCATCAAACAGCATTATAGATGCAGTCAAGTGAAAAGGGACATTAGTGTGGGCCCCTGCCAGGAGAGGAGGAGGAGGGCGAGGCTGCTGATGCTGCTGTTGCCACTTCTGCCGGAAAGGCCCAAAGGGCCTCGGGAAATCATATCCATCCCGCTCATCAATACCCTTATCCCCTACTCCTGACCCCCTTGACCTCAAAATCCCCACCCTCGAAACCCCCTTCCCCTCCTCACTCTAAACCCCTTAACACCAAAATCCCAGCCCTCCAAATCCCCCTTCCCCTCCCCACTCTAAACCCCTTAATCACAAAATCCCCACCCTCGAAACCCCCCTTTCCCCTTCTCACTTCAGACCCCTTAACCCTAAAATCCCCACCCCTTCCCTCCCCAACCTAAACGCCTTAGCCACAAAATCCCCACCCTCGAAACCCCCATTAACACCCTACTCCAAACCCCTTAACCATAAAATTCCCACCCTCGAAACCCTTCCCCTTCTCCTCTCCACTCTAAACCCCTTAACCACAAAATCCCCACCCTCCAACACCCTCCCCTTCCCCTCGCCACTCCAAACCCCTTAACCCCAAACCCAAAACCCTTAACCCCAAAATCCCACCCTCGAAACCCACCTTTCCCTTCCCACTCCAAACCCCTTAACCCCAAAATCCCCACTTTCGAAACACCTCCCCTTCCCTCCCACTCCAAACCCTTAGCCCCAAAATCCCCACTCCTTCGAAACCCTCCCTTCCCCTCCCTACTCCGATCCCCTTAACCCCAAAATCCCCACCCTCGAAATCCACCCTTCCCTTCCCCATTCCAAACCCCTTAAACACAAAATCTCCACCCTCCAACCCTTGCCCCCCAGACCACCCCCAAAATCCCTACCTTCCAATCCCTTCCCCCCCTCACAACCCCACTCCAACCCTTTAACCCCTTAAAGAATACAGATACCTAAGCCTTCAGTTCAGATTGTAAATAACGAAAGAAAGCTCAAAAGAATACAGATACCTAAGCCTTCAGTTCAGATTGTAAATAACGAAAGAAAGCTCAACGATTAGATTAAGCATCTTGAAGTACAGGCAGAGGGAGTCATCATTAAAGGAGTAATCATGATATTTCCTTTTCCTTGTTGTGAGAAACGTCCCTTGAGTTTCGCTGTACCAAATTTATATAACCGTTCAGCGTGATTACTGTTCATGATAAAATCACTAAACAGTAAAATTATGGGCCATATGTTTTGTATTAATGGTAAAATAATAATAATAATACTTCAATTAGATGACCACCGTCAGGATCTGTAAAGTGACAAGTTTTAAGTGCTCAGAAAAAAGTAAAGGTCTTGATGAGAATTAGAGAAAAAAAAACGTTAATTTTTAACCTGTATAACAGTTCTTAAAAGTAGAATGAAAAAAGCATTTTAATACCCTCTCCACTTTATCAGAGAACGAGAATATTCTTTTAAATCGAAGAGTAATAAGAGAATGTGAGGCAAGGGTCGAATAACATATAAGAGACAAGCACAACCGACATTAAAAATTAGGGAACGGGTCTTAATAGGTTAGAGCTTGTAGGAGGTTACCAGGAATGGAAGGAGAGAGAGAGAGAGAGAGAGAGAGAGAGAGAGAGAGAGCAGAGGAGACGGACATGGCCGTAGGAAGGCCCGCGGAGTGGCCGGTGCGTATATTGTGTTGATTATCTGCAGGTGCTGAGCTTCACGGCCGACTGACCCTAAATGAGGCTGTCAGGGCGAAGCACCCATGCTGCTGAAGGCTCCCTCACGCCCTCCCCTCTCCTACGCTTAGGTCCTATCCCTCCTCTCCCCTCCTCCTCCTCCTCCTTGCTCCTTCCTACTCCTGCTCCTCCGTCGCCTTCTACTCTTCCCCAACAACAAACCACTTCTCCTCCTGTCTCCCTTCTTCTTCCTCCTCCTCCTCCTCCTCCTCCTCCTCCTCCTCCTCCTCCTCCTCCTCTTTCTTCTTCCTTCTTGCCTTCCTCTCCCCGATACATCCTTGTTGTTTACTCTTTTGTTTACCCAGTGTACCTTGAGTTCTTGAGTTCCTCTTTTTGTTTATCGTTATTATTCGATACGCTTTCCTTTTCGTTTTCCTCTTGTTTCATTATATCAAACAGCCTGTCTACCTATGCATAAAAATTCTGTCTCTCTGTCAGTCCGTTCTGTCTGCCTTTCACTCCCCTCCCTGTTGCTTTGTCGCCCCTGAGGTTGGTCCTCACCTGAACAGGTCCTCCTACGGCTCCTCAACTCGCCTCTGAAAGCCACTTCCTTTCATTTGCGTTGTTTTAAAGTAAATAGCCATTCGCTCCTAAGATTTCTGCTTTCATTGCCAATTGTCGAACTCGTATGTTTAGAGTTAAGTATTCTAGTAATGTAGGATATTCGGGCCTGGCAAGTTGTCCGATGTAATTATTATCAGATTATTCTCTCATAGTTTCTTCTTCGTTTGAACTGTTAAAGTTATTTCATCAAAAATGATTAGGAGATTCATAAACACTGGTTATTTCCACGTCCTTTTATTCAATTTCTATCTTCAGGTTTCTTTTTTATTATTTTTGTAGCGTATCTCACTGTAAAGACACCTTTCCAGTTTTACGTGATTTATATGAAGATGGCTCTTTATTCCATATTTCCTTATCACCAAAAGCAAACTTTAACTTCAAAGTGATTTTCTTCACAGCCTTCTTTAGAATGAATGGCATTTTGTTGCTGAATCTGCAGGAAATGAATCATTCCGAGCCTAATATAAAGGAATCAAAATCATATGATTATGTTTTGTATTTTCCTACATGATAGGCTGCGTTTATAACTGGAACATCCGCAAGAAATATTCCCAGTAGTCGTAAGTAGTGGTAGTGTTGATAACGGCAGGAACGGTTTGATATTAGCTATAAGAACAACCATAATCATTGCAAGTGGGAAATATAAATAAAATGTGGGAAAAAATGGAAAATATATTTAAGTCGGTACAATCGGTTTCGAACAAACTAATAAAGACAGTTCCGAAAAACTTAAATTGTACCTTTTTATCACTGTGAAATAAATTGCTACCAGATCATCAAATAAAAGACCGACTAAATGCATTAGTCATGGATATCTACCAAATCTAATATGAGAGAGAGAGAGAGAGAGCATATTTCTTCAAGCCAACATAAATGCTGATAGTTTCTCGAATAATGCTGAGATGTGTCAAAATACATTTACTCGCCTAATTTTCGAAAATGGAGAAAAAAATAATTGAATGTAACTGTTACAGATCTTAATCATAAACAAAAATTATTTGAAGACGAACTAAAACATCGTAAATCTCATTAAATGCCAAAATAAAGTTAAATACACAAATAACTAAATATCTAGACTAAAAAAAAAGAGAGAGCTCTAAACTTACTATGTTTTAGAACAGAACAAGCAAGTTATATATGCACCTAAAAACCAGTGCGATTTATATTATTACAACTGGATGCCTGTAAGAAATCCATTCAGAAGCAGAAAATTTACCTTTTTTTCTAAGTTGTCAAGTATCTTATGATGTGGATTTCAGGTTTCTCAAGTCCTTGTTTCATGCCCTTCGCTGGGCCCCTCTTAAACTCCCGACCAATTTAAAAAAAAAAAATACAAAAAACTTACGCTTGCACGGTTCATAAAAATACATAAAGAAAATCTGAAACACGCAAAGACATACAGCATACAGCATTTGTCATACTCAAGTGCAAAGGAAAAATATATAGTCCCATAATAATTCAGTTATCGAAGAAAAATAATTATGAACCAACACAAGCTTCCACCATTCACCGTTGAGATAAAAAAAATGAATAAATTCAACGCTGAATAATATTGGAAATGGTTTTCTGCTCAGGCGTCAAAACCTTCTAAGAAATCATCAAACATACTTTAGAAGACACCATTTAGTGACTTTCTTTCCCTCTACCGCTATGCTTTGGATTTCTCTTGCTCCTACGTTTCCATGACTGCTAAACCTTCTCCTGATTTTCTTTTCATTGGCTTTTTCAGGCCAGGGATGGTTACGGCAGGCATAATCCGAAGGCAGGTGTAACGACAAAGAGTTTGTCCCATCAAAAACTGTCCTGGAAAAGTGTCCCCTAATTCTTAGAAACTAATTTGCAGCTACTATACCGGCAGGCCGAATCGTAAACCGGCTTGAAAGAAATATATTCATATCTTCCGAAGCCTAAAAAAAAAAAAAAAAAATTACGTCCGAATTTTCTCTAGAAAGAAAATATCAGAATAGTTTACGAACCCTATATCAGAATAGACTGATTTTGACTCTAGGATAACTTAAAATTAAATGTGACCTCCCTAATTTTACGGAGTTTGCCGTTAAGTATCATCATTTATAATAAACAGCTTCAAATAAATGGTATTACTACAACCTCTCTGAGCTCAGAAGAAGCGTTAAAATTCAGAACATGAAACTTAGTTATTATTTCATGTCAGAAAAATTCTGTCTTCAAGGTAGAACGAGGTAAAAGGATTTTTTTTTTAAACCAGATATTTTTCAAATAACAGAAAATTCTAAAGTTACATAATTTTATAAAGGATCATTCATGTAGCTCCTGTTAGGCGTCATTGCAACTTTAACTATGCTGTTATAGATCATCAAAAAATAATACGTCAACACGAAAATAATAATTTCTTCATAAACTAATGACTGAATGTAAACTGAAATGTCACTGCGCATGTTTATCATTTGAAGAACTGAAATTTAAAATGCCAAGGGATATGGAAAATTTCCTCTATAATAATAATAATAATAATAATAATAATAATAATAATAATAATAATAATAATAATAAACTCTCTTGAACTAGATAGTGGATGGAATGAAATTAGATGAATCTTTTTCATCAGCAAGCTTTATGACAGTTTTAGATACAGTTTTGAGGTAATCTCGTTTTGAGATAATCTCTGAGATATGTAAAAAAGTGAATTCAAGTTTATCTTAGCATAACACACGAACTGAGATCTTCCAAAACACAAATCAGACCAGAGATAAAATCTTCATAATTCCTAACTAAGAACTCATATCTTACTAAAACCACCCAAAAGAACTCGAAGCATATCTTCCTAAATTCAATGTCATTTTATATCTTTGTGAAAGTTAAGTTTACTTTAGTTCAGGAGATAGAAACGCATGAAAAAGCCTCATTAAATAAACTACCCCGACTAGGGGTTAAGCTATGAAGAAGAAGAAGGGGGGTGGGAGGAGAGGAGGAGAAGTTAAGGAACGCAACAAAAGTTTCTGAAGACTTAAAATAAACTACACTGGTGAAAACCTGCTGTTTCTTCAAGCCATCTCCTACAGTCATCCTCCTCCTCCACCTCTTCCTCCTCTCCAGCTAAGGCATTCCTCCCTCTTCTTCCCACTTACTCCTACATGACTACTGTAGTCTCGTCTCGTCACCTCTTGTCAGGGTGGGCTTGTCTTCCCTTCTCTTCCTAAGCGAGCATAGAGGGCGTCTGGTGGTCGGCCGTCGTCAACACACAGCTGAGTTTCAAACTACTCTAACACTCCTCACTTTTTAACGGTGAGATGTATCTCATTTTCCGTATGGGAGAGAGAGAGAGAGAGAGAGAGAGAGAGAGAGACTGGGCGTAATGTTATGAGAAGGAGAATGTGGACACAAGTAGATGCCATGAAACAGGAGGGGAGAGATAAGATGAAGGCTGCTAAACGTTAAATGTAATGTGAGTTCCAGGATGACTCTATGCACACATACGAGCATATTTACGAAAAACGCACATATATGTACTAAGTGACTAACAGGTAACAGTCAAACACATTTTAACGTTCTGTCCCTTGTCCTAGATAACAAACAAGACAAGCTTTAAAAAAAGTTGAACATAGTTTACCTCCGAAACAATCAAATAATATTGAATAGCATCTAGACATTCCAACGGTTCCAGAAGTATGTTATACAATACTTGCTGTATATTTTAGCGCTATAAAAGTTTCAATAATGCTAATTACTTTACAGAAAAGAGAATTTAAAGATGTTCAATAACTATATGTTCGATAATGTAATCATCTTGCAATGAAAGATTTTTTAGTTCATTTAACTTTAAAGTAATTAAACAAGCATTAGGGCCCTTGCGTTTATTTCATTATCTTTATAATTATCGGTAAAATATTTCCTTAGAACGTATTTGTATATCACTTTTTCATTGCGATTGGTTGGTTTCTAAGGGGATGCCTTGCGACAAAATCTGCTCTATTAGCCTAGCGAGAGATAAACTAATGAATAATAGAAAACGGAATTCTAAGTTTTGGCGTAGATTTTTAAATAATACTGCGCCAGTCGCCAATCATTTATTATTTTACTTATCTGCGATCTATGCTTTCTGTCTATTCAAATTCATGAATCCACTTTTTAAGTTGTTGTTTTTCCAATCAGCTTATTTTAAAATGTGTCGATTCTCGAAATCACTCTTTAGACCAGAGAGGGAGATGTTATCTGTATGCTTGTAAATGTAACTAGGTAGTTTGGTGAGCTGCCACCAGGTCGACGCTGCAAAACCTTGCGTTTATCTCCAGGTATCCGGGAACCACGGAGATTAGTTTGCCATTTAGTGTGTCCTGCGATTTTTTCGTTCATTTCTTCTTAATGGTACGTTGTGCGGCTTTTAATTGCGGAAATGGACTCAGGAATTCTTCAAGAAAATCTAGAGTAACATTCCACAGGTAAGGAAAGATTAATTTACCTATAACTTTGTGCGAGTCACACCAGTGTCGTGTTGTCAGCAGATACGTTTTCTTTGTTAACACGTCTCGTATTGTCAGCGTATTATTGCAGCTCTTCAAAGCAGTAGCGATTTCAGTTTTGAAATCAACATCATTAATCGTATATATGCCACAAATATAGAGTAAAAAGATCTTGCTCGCTTTATACAAAAATGTTTAGTATGTATAGTAGCCCATACGTTACTGGTTGTGTTAACGGCTCGTCTTTTGAACATGATGTTGGGCTAGTATTTCAGGACGACATAAACAGACCGTAAAAAATAACTATGTTATTCGATTCACCATGCAAAATAGTTGTATTTTTGTGCGTAACAAATCCTTGAGTTTAATTTATGTTTTCTTCAAACGATATGTCTCACAGGCTGGTGATAGCTAGCAAACGAAGTCAAGTAGCCTAAGCTAGACCTGTCTTTAAACCACTCATCTGGTAATATGCTTTGTTATTTAAACCATATTTAAGTACAAATATTATAATATACAAATTAATTTCGAATATATATATAAAAAATTTTATGATAAAAATGATCGAATCATCATGCCAAGGAAAAAATAGCCTATAAGAATGTTAGCAACAAGGAAAAAAATCTAGGCTATGCAAACGCTAGACCCATGCCTATAGATTTGGCATGAAGTTTTTTTGTTTGTCTTTTTAAGAACAAACAAGTTAAAATTACTTTCTTACGATAGTCCTTATTTAAAAATTCTCTCTAGGCATCGTTCCGAAGCCTCAGATTAGGAAGAAATGACCTTGCTAGCTTTTTCTGCCGTCGGTTTGTTTACAAGCCTTTCCAATACTTTATATTCAGGGTGGGTGGAAGGTAACTCCTTGTTTACTGTATGTTGATAACATATTAGTTAATGATTGAGTGTAGTGAAACAAGTTTTAGGTAGAATGCAGAGTAAGAGGTCAGGAATCCTTGTGGGGTGGGGGTGGTTGTCTCCAGGGGAGGAGGCAAAACCCACTGACCAGGCTAGGACACGGTGCCCAAGGTTAGGTTAGGTAAAAGTAAGTTTGGTTACGTTAATTCATGCTGAAATGCCAACTAGGCCTACAGTATTCCTGAACCCCTACCCTGCATTCGCTTCCATGTTTTAATGTGGACAGTAGAATGATAGAACTTGTCTGCAGTGTTAAAATTCCACATAGGCACCTGCAATTTCCACCAGTTCTGCAATCGCCTGAGGATAGCCTCGACAGAATTCAGCAGAAACTCAGGCGGTACGCTAGTGACGGCATGCCGAATGCGATCCTCCAGCCGACCCAGAGTTTTTGGTTTGGTACAATAAACCTCTTCCTCAGCCTAGCCACACAGGATAAAGCCCCATGGCGTGAGGTCAAGTCATGGCTTCTTGCTGGCCATTCATATGAACTACGACGACCCAGCTAACATCCCGGAAATTAGATGGCGAGCCACTCACGAACAGTATATAGCTGGTGCCTGTGCTGAATTTTAACACTGCAGACAAGATCTACCCTTCTGTTGTCGTTAGAACCAATGCAAAAATAAAGTTCTTAACATATTCTTAAGATAATTTCCATCCATCCTAGGAGAGATTAAAATGCAAAATTCTGCTTTAGACCTGAACAATTTTTCGCCGTTTTAGTTTTTTAAAATAATTAAAGGAAAGGAAAAAAATGTACTGCTTTAGTGTTTTTTCAATATAGCCGCCCGAGGTCACGCCCGGCAGGGAGATCGCCACTCCATGTTACTAACTCTATGCTTTAATCCTCCAGTATCAGCTAAACTCAAATCCTTATAGGAAACAATGTTAAAAATGATTGCGTCATAGACCAGGTTTCCTCTCCTACCAGTGACCTGGAAAATTAACCTTCTTTTCTGTTGAGAAGCCTATTATGTATTTTTTAAGTTACAATTTTGTTGCATTGCTAAGCTTTTAACCGAAACATTCAAGCCAGCGGATTTTGATTACGTTTTTATCGTCACATCTGAACATTTTCATTTTGGAATGTGTTGCTCGAAATCATTCACGCATCTGTTTGACCTGAGGAATTTCGGCTGTATGAATATTCGGAGTCTTTCGTTATCCAAATAAACGAAAACATCAGTGTTCTCCCTTTCTTCTTCTTCCGTGTAAGGACAAGCTATAACTTTGAACCCTTTTTGCTTGTATGTCTGTATAAATTTACGTTATCATTCTTGGGTTACAATATATTACAAGAATCTGTAAAACGCAATGCTCCAGGTTTCGTAAAAGAAAATGTATAAAGCATGGCTTTAAAGGGGGAACTGCATTCTTAAAGCAAAATCGAACAGAAATTCAAAATGCACCTGCTACCTCCAGCCAGTTCTCTTTGTTAAATTTGTGTCTTATTCCGGTTTGATTTACTCACACAATAATTTTCCTTGTTTTGCATCTTTTCAGTTTTTCACAGTCTTGGACCTTTCTACTCTTATTGCTCCCTCAACCCATACCAAATGTAATCATTCCTGTATTTCAATGAGAGAGAGAGAGAGAGAGAGAGAGAGAGAGAGAGAGAGAGAGAGAGAGAGAGAGAGTACTCAGGGTCACTTATGGAAATTTCCATAATGTGGAAAGAAAGCTGAGTTTCTCAACTGATTTTCAAATCCCACACTATAACCATCAGGGCATAACATTAATTTTTAACCTTGTTTTGTTAAGGTTTCGAATGATCAGTTTAACTATTTAATCTCATTTCCTCTCGGTTGTTCATAAGTCTTTGTTCGCATCAGACGTCAAGTCTGATCACGAATTATTAGTATATTCGTTTTTTATACAGTTTCTTAAATCTGTAGTTTTCTTGAATATGATTTAATTCTCTAATCGAATTCTTCTGCTGTGGCTATATTAAGGAGCCTTTTGGAAACGGAAATCAGGTTTAAGATTGTTACTGCAGTTAACGTATGCTGGTTTTGGAGACGGACAGTAGTTATAAGAAAGTTTGTGGCAGTAAACTGAAGCCCGCCTTGTGCTTTCACGGGCACTTGTTCCTACGAGCAGATGAAGCCTCAAAGGAATTTGGCGGTACAGCCATACTTAAGAAGGGAATCTTGAAACTTGAAACTGATGGCGATGGACATCGATATCGAGACTGTAATATGCCTTCACCGCTAAATTCCCGGGAGTTTTAATGCTAACGTTTCCAGAGAAATTAATCATTTCGTTTACTTAGAACTACTTGTAGGGATATCCAAGTTTGTCGTATGGCTTTTAAGTAATGAAAGAGTTAATGATATTTAGTTTGCTAAATTCATTGCATTTTGTCAAATCAGTATCAGCGGCTTGTAATACTCCATAATATATAAAAAATCCTTAGGAGTGGAACATTCATGCATCTTTCCTTACCGATTTGGTAATCTTACCATCGAACGTTGCATTTTACTAATGTCGACGTCTTATTCTCTTAAATTCATTTTAACTTCATCAGAAAAGGCCTTGGCAGTTGTGAAGAAACCCTATAACAGTAGATGGTCACTTTTGTGTATCGTCCATGTTTTTTTTTTTTTCGTGAATTGCAAAGGAAATTTGCCTCCGAAATCCTTTCGTCCAGAGCAATTCACACAGACTTATTGTAGCGAAGACCATTTTTGACGACAAAAGCATCAAGGATGGACAGTTCCAGTTCCTTGCAAAAGATGGCGCTAGAAAGCACCGTGTCTGTATATGTATAGCCTCTATTAACGCTTTTGTACCCAGCGATAGTTAGTTTATATATCTAACGTGTTTTTTTCTTTGTGGAGTGCATAGCGCCCTTTTACATTTTACTTTTGTACTTTTCGTTGTTTCTTTTTGCATCCTGGCTTTTCCTATTCAATGATTCTGTAAAAGTTACCTGCAACCGAATGCAGAATATTTTTTTATTTTTCTATTTTTATATTGCGGACCATTTATATTCTAGCTTGAACTTCGAAGCCGATCTTTTTATTTCTTTTTCTATCTAGACTTTTATTTGACTCGTTATTTCCCCAGCTAACAATTTCACTTTTTTTTTTTTTGCTTTGATGTTCCGACTTCAGTTCATTTCCAAAAACTAAAGTCCGCTACATTGTTACGACAAAAGGTTGTCACTCTTGTATTTCATCATTATTTGTTCGCCTTTCCTTTTAGCTCCGTTCTTTTCCGTTCACTCGAATAAAGATGCAACACTTGCCATCGATTTCGAAGACCTAAAACAATTGATAATTCCGGTACCGTGATATTAGTTTACTTAGTTGTTATCTAAGGGAACACGTGGATAAGTACTATCTAATGGAAGTCAGATAAAGGTTGCTATCTAATGTAGACCTTAGATAAAAGCTGCTAAGAGAGGACTCGGACAAAAGTTGCTATCTAAAGGAGGATTTGGATTAAGGTTGCTATCTAATAGGGAATTTGGATAAAGGTTGCTAATGTAATGGAAGACTTGGATAAATGATGCTATAAAAGATTCAATCTAATGGAGGACTAGGACAAAAGTTGCTTTTTAATGGAGGCCGTAGATAAAGGTTGCTGTCTAAAGGAAGCCTTTGAGAGAACTTAGATAAAAGTTTCTGTCTAATGGAGCCCTTAGATTAAGGTCGCTATTTTATAGAGGGCTTAGCTAAAGGCTGCAATCTAATGAAGGAGTTGGTTAAAAACTGCTGTCTAATGGAGGACTTCGAAGATTGCTATCTAATGGAGAGCTTGGATAAAAGTTGCTATCTACTGGAGAACTGGGATAAAAGTTGATATCTAATGGAGGCCTTAGATTAAGATTGCTATTTTATAGAGGACTTAGGTAAATGTCGCTATCTAATGGAGGAGTTGATTAAAGACTGCTATCTATGGAGGACCTCGAAGATTGCTATCTAACGGAGGACTTGGATAAAAGTTGCTATCTAATGGAGGCCTTAGATTAAGGTTGCTACCCAATGGAGGGCCTGGATAAAGGTTGCTGTCTAATTGAGGTCTTGCTTAAAAGCTGCTATCTAATGGAGCACTTCGATGTCATTTTAAATTCAGACCTGACAGCGCCAAAAAACTGGCCATGGAACTTCCTCTGAGCCAAACTAGTTGAACAGGTTTTATTAACTTCGTTCAGGGAAAGCAAAAAGCTGCTGATTAATCCAGCTATCGAAAAAAAAACCGCCAATCAGGTGATAAGAAAGCTTGCCGCAAGATGTCATAAAAAAAGACATCAGTTTAAAAAATATTTGTTCATTCTGACCTTCTGCAGAATAAACAGCAGCTTTCATCTAAATGATAAATTTAATATTTTGGGGATGGCGAGCCTACGGAGAAAGAACAACAGCATGGAAACAACCTTAGAAGGCATCGCTTGTTGTTTTTGGGGTTTCGGAAAATCAGAATCAAAATTTGAATTGGGGGAGAAACACTTTGCCGGTACACTGTCTACAACAGTTCAAACAATTGGGCGCATTGAAGTTTTTTAGGGCATCCGAAACCCATGTTATGCGTGAGTTAGAAAATAAAAATTTGCCATGGAAGATCTCAAGTATTAAGATTAAAACCCTGTACTGACCATTCGGGATCAAAAGACAAAATGAATCTGAAATATCGAGATTTCCTGAAAATTTCGTAAACACTATTCTCAACGTGACCATATACAATTGACACTTTCAGTGGAGTAGATTAATGTGAGAAAGCCAAGCAAGGTACAAACATGAACGACCTCTCACATCAGCGGGAAAAGACGATTCTAATATCACAATTGGTCAGTCCGCTGCCATGACATCATAATAACCCCAATTATCCTGTAGTGAAGTAGTCCGAGTTAAGACTCAGTTAAAATTAAATCACTGTGCAGAAACATATTGTTTTAATTTCGTTTTTATTATTTTTTTTTTATTCTGAACTCATTTTTGTTAAGGTAATCTTCAAGAAACTAATGCGAAAAAACAAATAAACTAGTTGGTAAGCGAGAAGGCAAGAAATCCTCTTGCTGGTAGCAGTTTCAAAAGACTTAAACGTACACAAAAGGAATTTGCCACATTCTAAAAGGGAAATAAATAAAAGCTAATGGCGAAATTTTAAACGTGATATTTATTCTCACCACCTTTGTTCTGAAAAAAGAGAAACCTAAAGTAAGCTTAAAAAAAAAAAAAAAAACTGGATGTTTATATGTCATCGTGCAAATGACGAAAAAACAACCTCTTGACGATGGTTGGCTCAGAAGACCCACAAAAATACTAATGTTTACTTTGGCGCGTTCCGGAGTTGGACATTACTCGAGTTGTTAAAACTGCAACAATTGTACTACCAAATCTTATGAATTTTGTGCTGCCCATTCGAGAAATTATTATTCAGCTAACTGGGTTCCAATACTCCTTCAGCTTCCAATACTCCACGCTTCCTTTTCGTTTCACTAAGTCTGCTGTATATATGACGTTATTACATCTCTCTTACGTCTGCCAAGAGTCTTGATTACCTTCATCTCAGACTTAGATTTAGGTTTTGGATCACGATTGAGGATTAAGTCGTTACCAAAACAAAGTCTTGAACAAGACAATTGTAAAAGATAAATTAGCGATTACAGTGAATATCTGTTCATGTTAATTACTCTCGTATTGGCATAACAGCTATTTACTAAGTGTCAAAAGACAAGCCAAATAAATGAACTACATGAAGCATGGCCATAATGCATGCAGTAAACAATTTCACGTCAATTAGACTAAACAACAATTGCTTCAACTTGGAATTTAAAGCAGGGGCGTTCGTAAACATTTGGGGCCCGGGGGGCACAGTCCTATTTGGGACCCCACTTATGGGGTGGGAGGCCAGCAAAACGAGCAAGAGCAGCTGGGGGGAATTTTAGAATATGAGCAATTGTAGGATCATTTTAAAGGCATTTGTAAATGCAAATTTCAGAATTTAGCTTGTCCTGTTATAGCAGCAATGGTTAAAATTCGCACTTTGTGGCCCCCCCTCACTTTGGGGCCCGGGGGGCGATTTGAAACAGCGCCCCCCCCCCTCAGGAACGCCACTGATTTAAAATAATGACGTGAGGGTACTGTGCTCTAGTATTAGGAACGAAGTAGAAACTTGATAATAAGATACAAGAGAGATTAACTAATAACTCACCTCTCCCACAGCTGGTTTCTATTTGAAACGTCACATGCAAGTTTTTGACAACGAATAACAATCAGAAGGGGTCTGGTATCATCTCACGAACCAATAAGTTATTTGTCTGTTTCTGAGAGCATTTGATGCACACTCGCGCATTTTATCCACTGCCTTTTGTGTATCTGCATTAAGCAGAGTGATTAAAAAAAAGGCAGTACATTAAACGCGTAAGTGTGAACCAAGTGCTCTCAAAACGTGAGTCACAAATTGAATGTATGAATACGTATTCTGTCAAGAAAAATAAAAAAAAAACTGTGAATATTTTCTCTCTCTCTCTCTCTCTCTCTCTCTCTCTCTCTCTCTCTTTTGTTTTCATTTTGTATTGCAGATTGAGGCCTTTTGCTAACCAGCAAAATTTTATAGATTTGTTCTAATAACTTTTTCACTTAAAATAAGGAGAGAGAGAGAGAGAGAGAGAGAGAGAAGAGAAGCAGCCATCTGCATAAGCATATTACTTCCTTTTCACAGAATTTTTCGTCCACAACATTGACTTTTCAAAAATTATTCAGTTTCGGCAATAAATTCATTTTTAAGCCTCTCAGTCTTCCTGTAAACTAAAATCAAATGCACCGGAGAAAAATCCCAGGAAGTGAACTTTGCGGCCATGAACTGGCGAAGCCGACGGGAAGACTAAAGCATTAGTTGAATGTTTTATCATTGTCGTTCGTCAGTCAGTTTTTAATCCTTATCCCGCAAAAGCCTAATTGACATCTTTCCGTGCAAAGGTCGGTGATGCAGATGACGAACGCATAACTCCCGTAGGGGGGCGGGGTAGCCGTCAGTGCACCTCACGACGTGCATCACTAAAGGTTCTTTGCAGCGTCCCTTCGGCTCCTAGCTGCAACCCCTTTCATTCCTATTGCTTTACCACCATTCACATTATCTTTCTTCCGTCTTGCGGTCTACCTCTCTTAACAATTACTATATAGTGCAACTGCGAGGTTTCCTTCCTGTTACACCTTTCAAACCTACCTACTCTCAATTTCCTTTCGAGAGCTGAATGGCCTCATAGGTCCCAGTGCTTGGCCTTTGGCCTAAACTCCATATTCCATTCCATTCCACGAACGCATAACCATGTTTAGATGAGGTGTTTTGGAAATAGGTATAAAACAGGGCAAGGAAGAGAGCAGCGCCGGACATTTCGAGTGATGTGACCATTCCGCCTCTTGACAAATGGAAAAACATAAAAGTCACAGGGAACTAAAGCAGGCAGTGAATTCCAAAGCCTGGTGGTGAAGGGAAATAAATGATAACTGAAAAGAGCAGTCCTGGGATTACTTACGTCCAAAATGAAAATGTCGAAGTGGCCGTAGTACCTGAACTCGGCCCATTCCCTTTACCGACCAAATCACATAGACGAACAGACTACACGCAAACTCACAATTAGACAAATAAATAAAGAAAAACAACTTGCTTGCTCGTCGCTTGACGTGGAGTAAGGGAGAAAAATAATCTCCAGACCTTCCAAAAAAGAGCGAGCGGGAAGTGGTGACACCACTGAAAACCATACACAGTCACCCGCAAAGAAAAAACCTAAAACAAAAGGAAAAACCCCATGCTTTGTCGTTGCTTCAGCTGGAGGAGCGGAGGAGGAGGAGGAGGAGGAGGAGGAGGAGGAGGAAGAGGAAGAGGAGAAGGAGGAGGAGGATGGGAGGAAGGAGATGAGCGGACACCATCAACCTCTTTAAGAAAGACTATCTCCCGAAGGAAACGAAAAATGCTAAATCCAATGGGGCTTGACCGAGCAAACTTGATTCACCCCAGAAAGGAAAATATATATTTTTTTTTTTTCAACCCGACGTCTTTTTTTTTTCCCCCTTTCCTTTTCCGCCCTTTTCCCCAACGCATCTTAGGGAGATTTCCTTCTTGCTCGAGACAAGAAGAAAATTTACATTCGAGAAACTTACCTCACACGATTTCTGCGGGAAGAAAAGAGGAAGAAGAGATGGTGAACAGAGAGAGAGAGAGAGAGTGGCGATGCCTTGTTTCCTCTGCAGTTAAGTGCGAGGGCGTGGACGGACTGTCTGATGGCGCAGCCAGTGATACATTATTAGAGTGGAGGTTATTTCAAGCCTCCTTATAAAGCAGACCTCGGTCGAGGAGCAGATATCGGCGCTGAATCAATGTAAAAATAGCTTTGGAGGGACACAGACATATGGGATTAGGCAGTATATCTATTCTAGCTGCGAGTCTTCAGAATAAAAATAAAACGCTTAAAATTACAGATTAACTCATTCCTTACAGAGCAAAAAGCCACCGCTCAACGCAACCTTCACCTTATTTGAGACCGAGAATAAAAACGACTTTCAAACGGGAATCAGTTATTGTCAACTCGAGAATGTCGATGCGTCTTGTTCTTTTGGATATCATTAGAAAGCTCGGCTACTAGCCTTTGAGTTAATGTGGGTCAGGCGCAGAAAATAGCTTTTCCAAATGAAAGGAACAAAGTATTTTTGGAGATTTCTGATGGTTTTATTTCTAAAGACGGCGATACCTCGAATAGATTTTTCTTGACGCACTTAAGAGGTTTTTTTTTTTTTTTTAGCCCGGTCTAAAATGCGCAAGGGTTGTCTACCTTTTGCATATCCCAAAATACTGAATAGTTTATTTTTGTCTATAAGAACATATGTTCAGTTACTTTATTAGGACATGATGTAAATGTCTTTTAAAACCTTTCAATAGATTTGAAGTACATCCCATTTTGACCCTCTCTCTCTCTCTCTCTCTCTCTCTCTCTCTCTCTCTCTCTCTCTCTCTCTCTCTCTCTCTCTCTCTCTCTCTCTCTCTCAGAACATTCTAAATGCGAAGGACCGAATATGGAAGTAGCTCTTGGCATAAAATTATCATAGGATCTTCCTTCATGATGCAAGACCCAGACAGGGCCTTTCTTTCTTGCAATTTCTGAGCCACGCCATACATGATGCACAAGGGGAGTACATTCTGTTGCAGAGAGGGTTTGTGGCAAAAATTCTGTTGATTCCTACTGGTCATCCATGTGATAGGTTTTCCCAGAATTTCCTCTCAAACTTGTGCGCAGCGATTGAAATGTATCGTAATTTTGTAAACTGATACAGTTTACAATTTGTGCATTATATATATATATATATTATATATATATATATATATATATATATTATATATATATATATATATATATATATATATATATATATATATATATATATATATATATATATATATATATATATATATATATATATATATATATATATATATATATATATAAACATGAAACTGTCAGTTCTATACGTGGTTTTGACCGAGAATGAACGGAGAAAAAATTCATGTGAAATATACTTTTACAAAAATTGAAAACAGAGATAATGCCAGCAGTAATAGAACTTGTAACTGGCAATAATAGTAATAATAATAATCATATGAATCAAAAATAATAATAATGGTTATCATTTTAGTTAGCTTATGTGTAAACAGCAATGAAAGAAAAATGATAAGAACCACAACATAAAACAGATGAACTATGAGACTTTACGACCAAGCAACAGAAAATGAACGAATGAGGTAATGTGTTGATTACCTGATAGACAGGATTAAAAGCGAAGCATGAACTTCTATTCCTCTTTAGCAAGCAAAATAATAAAAATAATACAAGGCATATCTGCACAAGAACAAGAAAAAAGAAGGTATAAATGCACGTAACAATATGGAAAAACTTTGCATCATCAACACTGTAAGATAACAAAGCCATAATAAGCGTTCATCATATTTAAGACGGTTAATGAGCCTCGAAAGAATACAGAGAATAATGGCGCGATAATGAATATTTAGACTGATCAGTGACACCTTGGGGGAGAAGGGGACTACTGCTAAGCTCACCATGATAATGATAAAGAAAACGAGATGATGGCTACATATTAATCTTGCTTCTAAATATTTTTGAGGACTTTTATTCCCGCTGTTTTTATCTCCTTCAAATAAACAGAAAAAACGAGTTTAGCTTGACTTCCGAAATCATTAGGCGTGGGATTTTGAAACTATTAATGTTAGCCAACGTACTTTTTTTATTTTTTTTCACCTCAAAGACGACCGAATTCTAAATTCCATTGTCCATTGTCCGGTCCGATCTTTATGTGCTGTCAAGTCTTCAACTAAATGAATAAATAAAAAATAAGTAAATAAATACAATTACTTAGGTACACTGGTTCCGTAGTTACCACAGCGAGATTACCACTTGTACTGAATCTATTCGCTTTCAGTTCAGTCGCTATCAAGACCGAACAAGGCATTCGAATCCGGTTTGCTATGCGGGTTTTAACTCTCCGCACCGGAATCAGTTTTAGAGTACCAGATGCTAGCTTTTGCGTTCATTCTCCCCACGTCGATTTCGTGCAATCAAAAATTCTTAGATTGTCACGAATAAAAAAAAAAAAAGTTATTCTCTAGTCTGTAATCGTGGCCGTCGCGCCATAGAATGGCTTTAGGTCCAAGGGTTTAAGGAACCTCCCTCCAGGTAATTGGTATAGAGAAGTAAAGATGTAAAGGCAATACTGACTAAACCAGACGTGCTAACAAACATAACTATAATAGAAATTGTAATGTTTAGGGAAAAATGAAAAGAGAAACTGTGTAGAAATTCCAAGATATTTCATCAACAACAGTCCTCCTCCTCCTCCTCCTCCTCCTCCTCCTCCTCCTCCTCCTCCTCCTCCTCCTCCTCCTCCTCCTCCTCCTCATCATCATCATCATCATCATCATCATCATCTTCATCATCATAACAATGATTCTGTTTTAATTTCAGCTCAATGTCTTACACAGGTACACAGGGCAGATGTAATGCAGTACAAAATTATAAATTTCGAAGGGGAGTGAAAGGTAAATCTCAAAAACAAACAAAACAACAACAGCAACAACAACAACAACAATAAAATAATAATAATAATAATAATAATAATAATAATAATAATAATAATAATAATAATAATAATACATAAGACGGCAGCGATGGGTTAGCTAACATTAGGATACAGAAAAGTTGCAGTAATATTTCTTAAGAATTAAAGCGATAAAGACGACATCATGGAAGGTAATATGCAACTCAAAGAGAGACAGCAGGGCGTGCACAACAGGACAGGTATTTCAACAGACGCCGCCCTGCAATGGGAGTAGATAACATTGCATGCAAAAAATCATAGTACTTGTACATATATATACAAAAGAATCGTGAGAACAAAATAAAAAAAAGCAATAAAAACAAACAAACATGCACAACAACTGAGGTCATCCGTGATGCGAAAGTGAAAGAAGTCAGTTACATAACAGATGTTTCATTATCTTATCTTTCCAACAGTTTGTGTCTTCTCGCTTCACTGTTTAAGAACTGTTTGATAAGCGGATTGCTTCTGTTTCTTTACCAGGGTGATCAGACTGGACGTGCCTGGCCTAACAATGGTTTTAAAATCGTCCTGGCGGTTTTCTGCGAACATCTGTGTGGCAGGATGGGAGCGAGGCGTGTTAAGGCGCCTCAGAATGTCTTTATGGAAAAAACGAATACGTTTCATAGCCTCTCGGGTCATGTTCGTCCATAGACAGTATAGCAGTAGGAGGGAAAGGGCAGCTACATGGGTTCTCGGTGACAAAATGAAAATGTTGCAGTCGTGTTGATAACCGCCCACACAATACATCCTATCTGTTCTGTGTCAGCTGTATCTGTTGAGTTGTCCGCGATAATGTCTTTCAGATAAGGGGAACTTTTGTACAAATTTCAGACGGTGATTCCCGAGGAAAATTTGTGGCTCTTCAGCGTGCTTGAGGATCTAGGGAGTAGCGACATGCACTGGGTCTTAGTTTCGTTGTATATAATACATTTATCTGCATACCTACGGTAGGTATCGACTGAAAGGGCGATTAGAACATCATCATCGGCTTGACAGAGATTTTTTATTTAGTCAACATCATCGGCTTAACAGAGATTTTTACATTCCGTTGCATCCGGCTGGGAATGAGTTCAGCTTGACATTCAGGGCATATATTATGTATGCGCATTAATTAACCAAATACAGGGAGAGAATGGCACATTCACGAGTTCCGTTTGAGAAACCGAAGTTGTTTGACAATACGTTACCCCATGGGACACAGAATTGTTGTGTGAGTACCAGCGACGTAATGTGGAAATTACTGTAAATACAGAGGTGAGCGTCTTTTTTTGAAGCTTCAAGAGGAGTCTCAGTACTTTATTCTGCCAAATGTTTTGCACACATCAACAAAACTTAAGAAAACAAAAGAAACTAATGATAGGTATTAGCTCAGAAATTCTTTCAGGATTTAGATGCAAGTGTCGGCTGAACTGAATGGCTTGCTTTAAATCCGAACTGGTTGTCTGCAGTATGTAGAAAGGGGAAAAGGCTTCACAATGACAGACTCAGACACCTTCGATGCGATTATAGGATTGCAATCGAGCGGTAGCTGACTGGGTCAGCTGCGTCTTTTAGCTTGATTTTGATTGACAGTATTGGGTGAACTAAAAGTACGAATTCTGGGAGACGCTGGTGAACTATGCATACACTGAATAATGCAGAAAACAAATTGTAAATTCCAGGGTGGAAAAACTTAAAAGTGTCTGCAGGATGACCATCACAACCCGTAGCTTTATTGTTAGGTAAACCCTGTATGGCATCGCCGATGTTACTCGGAGTAATACGATCGGCGAAATGAAATCGAATGCTATCAGTAAGGAGGTTATCTACTTCCTTTCAGGAGCCTTGATCGTTTATGCAATTCAGAACAGTGCGAAAATGATCGTCCCATATTCTCGCAATAGCACCGTCTCCGACACCTTCTTCTTCTACCCATGAGAGTCTGTTGGTCTTTTGAATTAGGAGCTGAATGTCAGTCGAGAGACGACGGTAATATCCAGGGTCTAATTTTATACTCGCTGCATCTGCTGTTAGTTGCTTTTCATTAAATCGACAGTGCCTCAGAGCAAATTTGAACTGTGCTCTTGCTTGTCTCATTAGCATTGCAAAGTGTCCTTCCCTCGGGCTGACATTTTGCTTCCACAGTAGAAATATTTCTCGTGAACGTGAATCTAGGTCTGTAAACAGGTCATTCCAGCCTGGTACGTAACGACAGTTACATTGGCGTAATCTAAAGGTATAGTTGCTTGCGTTCAGCAACTCGCTTATTCTACTTGAGAAAAAAAAAAAAAACTGGTGCCCATGAATAGGTATATACTTACCATTTATAACAGTTTTAAATTTTATATATATATATATATATATATATATATATATATATATATATATATATATATATATATATAACAAAAATACTAACGAAATATAAAATGAACAAGTAAATACAAACTAAAAGGGTGAGACGTAAAATAACGAAAAATTAAAAACACAAACATAAAAATGTGAAATGTAAACAAACTACCACTGTAAAATATTTAACGACCTAATTGATACCTACTATGAAATTACACGATAGCTAGTTGAATACCAGACGAGTTGTTGTTCAATTCCGGCTTCATCTTTTTAATAGTCAGCGACTCTGAAATTAAAAGGTCAGTCTGTTTGAACAAAAGACAGTACCCGAAAAATCCAGGTCAGTGAAAGGATGGTCCTGTGCTAAACTGTGTTCTCTAATGGCAGAAAAGGGTCGCTGACTATTAAAAGATGAAGCCGGATAACAACAACTATAGCTATCGTGTAGGTATCGTTAAATATTTTACTTTGTGATGATATTGTTTATTTCACTATGTTTGTATATTTTTAATTTTCGTTATATAGTTTGTATTTACTTGTTCATTATATTTCGTTATATATTTTTGCTAAAGATTTCTTATGACAATTCATTTTTATATATATATATATATATATATATATATATATATATATATATATATATATATATATATATATATATAATATATATATATATATGTATGTATATTTATATATATAATGTGTATGTATATATATATTATATATATATATATATATATATATATATATATATATTTATATATATATATATATATATATATATATATATATACTGTGTATATATATATATATATATATATATATATATATATATATATATATATATATATATATATATATATATATATATTTCCTTTCGTTCTCCGTCTTCAGCTTCCCTTTCCCCGTATTTTTCCCCAAACAGCGTAGGAGCCCAAAGGGATGGGGTAAAGAACGCCCCCCATTCCAGAATAACTTCATCTTAGACGAAAATAAGCGAGAGATTAAGCCGCAACGGAAATAAAACTCCATCGGCCAGATCAGGTGACTATGAATAAAGGTCATCTAAGAATAACCGGTTAAGATGATCTACGTTGGGGGCCCTGCAGGACATTCTTAGGTCTTCAGGAGGATACGCTTTCGGAGGACACAGTTCGCAATGGGGAGAAAGGAATGTTTTATGTCGGTATTCGCTGACATATTATGAAGCGTGTCTTAATAAATTCTGCTGTAAACAAGGATCTCTCTCTCTCTCTCTCTCTCTCTCTCTCTCTCTCTCTCTCTCTCTCTCTCTCTCTCTCTCTCTCTCTCTCTCTCACATATATATATATATATATATATATATATATATATATATATATATATATATATATATATATATATATATATATATATATATATAATACATATAAATAACACACACACACACATATATATATATATATATATATATATATATATATATATATATATATATATATATATATATATATATATATATAAATGTTCTGTTTATGTGTATGTATGTCTTTCAATCATTCGTACGTACATAATTGTTTGAGCATTATTAATACAAGAATCAAACAACAAGAGTTGTGAAATGATTCCGTATCCTCGTAAGATATTATTCGTTGAAGGGCATAACGTTTACCTGTTCTGTCAGTTGACATAAAGGCCGAAGGAACGGACATCAGAACGCCTTTAGCTAGCAAAACGGGTTATTAGAAAAGGAGTAGAAATAGAAAGTTGAATGATATTGCTAATGCCAAAAGTAGGAATGACCGTAATTTGACTGTTATTTTCAACCAGAACTACTACGGTGCGATGAACTTCTTCGTAGACTAACTGGTGGAAGAATTGCCAAAGGGAAAGACGAAATTTGTTATTTGATGAATATATAAGTGACTAGTCACACTCGTCATAAACGTGGAAAGACATCTTGTTAAGATGAATATGAAAATACCGATATATAAAAGGAAACCATTCCCACGCAAAATATTCCCTGGGGAAAATATGAGGATGAGAGAGAGAGAAAATAGGTGATCGTTGTGACAATTACGTAAGAATGAGAGAGAGAGAGAGAGAATTCAACCTGTGGTCATGAAAATAGAAAACCTTCTGCCTGACTTGATATAAAAAGCGCGACCTAGATTATTATAGTTGTGACCTACTCCGATGTGGCATGCCACCGCTCAACTCTTTTAGACGCTATAATACTTACGTATTTATATCTAGATGGGCAAGAAAGACAGAGATATAAATAAAAAACGATGCATTGTTATTACCCGATTTATCCCGCCTCTCCAGCTTCTATTGGCGATTTGTGAATGAAAGATTATACTAAAGAGGCCCATTTTAACAGTATCACTCACAGATGGCTCATTCAGAGGCATCCAGACTTTTTTTTTTTTACTTTTTATTTTTTTTTTATTTATTTATTTATTTATTTTTTGTTAGAAGGATCTCCACATAACCTCAGTGTTATTGGTTTCTTGACTTAGAGACCATTCTCTTGTCTTTGATAGAAATCTCAGCTTTCATGTTCTCTGCTCGTGCCTTCATGCACACATATGCATGCATTTGAAATTTATGAGGTTATAAGTACATTTTAAACACAGACATATATATATATATATATAATATATATATGTATATGTATATATATATATATATATATATATATATATATATATATATATATATATATATATATATATATATATATATATATATATATATATATATATATATATATATATATATATATACACACATGTCCCCAGGATAAGCTACAAATGTCGTTTAATATCAATTTGCTTTACCTTGGAAACAATATATGTATACCTCCTGAAGGAAAATTATAGTTGATAAGAAGCTCGAACCACCGAACACAGATATTTGAATGGGCTGTAAATATATCATAAGCGTCGTGAGTTCTCGTGTTCTGCGGATCGAGCCCGTGATATATACGAACTTCTTATCAACTATAATTTATATTCGATAAAACATATATACATATATTATTTCCGAGGTAGAACAGAATTGGACATTAATTATTGCTGATAATGCGTGTATATGAATCACAGTGATGTGATAAAAGTTCATTCGGCGTATACACACACACACACACACACACACACATATATATATATATATATATATATATATATATATATATATATATATATATATATATATATATATATATATATATATATATATATATATATCACATAAATATATGCATGTATATGTATGTGCTTAGATTTATCGGTTGTTTTCGCGTCTATAATTTTTTATATAATTTCATCCTCTATCAGCTACAATGTTTATAGGTAAATGATTATCATTTCTCTTCCTAGCAAAAAATATCTGGAAAAAACAAGACGAATTCAAATATGTCTATTGCCATTGCCTTCCAATATACTGTTCATTCATCGTTTCTGGAAAATGAAGCTAAATCAGAAATCTCACGTAATGCCAGGAAACCTCGCATAGATTTCCTGACCTCTGAGAAATGGTTAAAGAAAGCCGATACCAATCAGACATATTTTCACACTAAATGTTAACATCCTTGAAAATAAATAAACTTTTTTTTAACTTCCGAAAGGTTTTACAAGGAGCCGGGTAATTATGCCGGCTATGAATGCCGCTACTCAGAAATTATTTTATGAATTATCCGAAATCCTGACTATGTAGGCTTACTTTCCCTTCTCATGCGTTGTGTAAACACACCTCGGATCCTACCTTATGATGGTAGACCCATGATCAGTATATTCACATGATATTTATTCAACTTCATTGACAGAGAGAGAGAGAGAGAGAGAGAGAGAGAGAGAGAGAGAGAGAGAGCATGACAACACTATATTCACAAGGGCGTTGATGTAGAGGACCTCGGAAGGGGACAGAGGTGTAGAAAGGCCGTCTTTTGCACCCCAGGATGGGAGGCTTCATCATTAGGAGAAGGAGGAGGAGGTCGTAGGGAAGGAGCTAGAGGTGGGGAGGACTATGTGATGCAAGGAAGAGATTTGGATGCTGAGGGGGAGAGGGGGAGGGAAGGAGTCCATCCGTCCCAGCTGGTGGAGGAGGGAGCGGGAGGTACTCGTTCCCTAGCTACGTCCTCCTCCTCCGACCCCACGCACACACACACACTTCGAGTGGAAAATCGACCCCGACTGTTTCTTAGCCTAAGATTCCCTCCCTCCCTCTTACCCCTCCACTCCCCTCCATCCTACCCATGTCCCTCTCCTCCTCCTCCTCCTCCTCCTATCTGGGCCCCCCAACCTCATCCAACCCTGCTTCTTAGCGAGTCCCTCAGAACCTCATCTCATACCCCTGCAACACCTTCCCTAAAATGTATGCTGATTTATTCACCTCTTCCCTACTCGGGACGAACTTAATTACCCAATGCATTGTAGTGAATTTCCGAGCCCATTAATGATAACATCAAAAGTCATCCGAACGGACGGAATGAGGATACCCGAGAGACGCAAGAAGTATTCCGCATTTAATGCGATGCTTGCCCTTAAAAGTAACGACAGACAGAGAGACAAGGAAGCATAATAATAATTTCTTCTTCCTCTATTAATTTGCGTATGAAGCTGCAAAATTGAGCTCAGCCGCTTAATTCGTGAACTGTATCATATTTCGCAAATTCCCGTAACCTGATAATCCCTTGATCAAACAATAAAAAAAAAGATGAACAAAAGCGCACACACAAAGAACGCATAATAAGGCAATAAACAGTACAGACATCACAACATCATTACTTGAATAAATTTGATATTCAACACAAACATCAATCCAGAGCTTGTTGCATTATTTTTCTGATAACTCACGAGAGGTCTGCAACCACCGAGAGTATCTTGAGAGAGAGAGAGAGAGAGAGAGAGAGAGAGAGAGAGAGAGAGAATTACATGCAGATAACGATTTCTGTTTAGCAAACCAGTGATTATTATCATTATTGTTATCTGCAGTTGTAGTAATAATACAATATCTATGAGGAATGTACTTTCTGTTGCTCTTATGAACAGCAATAGCAGTAAGAGAATTATCGATTTATCAGAAAAGATAGCAGCAATTCTGATAATAAAAACAACCGTTTTAATTACTGTAATGCTAAAATGGAGCTCTGCAAAAGAAAAGGATTAAAAATTCATAAGCAAAAATTAAGAAAAGAAATTGAAATTATCAGCACTTGTTTACAACAGGAATAAAATACCCGGAGGGGAACTTTATAAAGGTACACTAAGAAAAGAAAGAAAAAAGTCTAAAAAGAGAGAGAGAGAGAGAAAAAAAAAAGACGGTAAAGAAGGTTGCTGGAACTCCCCATCGGTTTAACCCAGAGCCAGGGGGCAGTATTTTTCGGAGGCTGTAATTTATGAGTTCTTTCGTATGTAAATTTTTTGGTCAAAGAGGAGCCATCGTCGATATGTTTCCCTTGTCCAAGATATAGGTAGAATGGGCTACTTTATTCCGGGGAGATCTACGATGCATAAATTCCACCGGGCTGAAACAGTAGGCAATGTGAACTATCACGTTTCTTGCAGACGATTATCATAATGCTCAGGTGGAAGAGAGGGGGGCCGCCGTTCTGTTAACGCAACGTTACGTTGGACGTAAGATCGTGTCTGTTGGGTACAATAACAAATTCGAGTATATGAAAAGTGGGTTGTTGGCCAAATCTGATGACACTGCAAAGGACGTACAGAGTATAATTTGGTTTGCAATTACTGATGTTGGAAATTTTAGTAAACACCCATAGATAGATATATATATATATATATATATATATATATATATATATATATATATATATATATATATATATATATATATATATATATATATATATATATATATATATATATATATATGTAAAATTCTATGCATATGTTGTAATATCCCCAGTCAACTTTCTAATTTTTGTAATAGGTCAGAGAAAGATTAGATGCGCAATGATGTATGACAATACAAACAACTTGTACAAAAAGACTACTTCCTAATCCAAACTAAAGTAAAAGGACGAAGGCAAAGAAAAAAACAGAACTCGGGAAGAAAAGTGGCATCCTTTGATGGTTTTTTTTTTCGACATTTTTTTAATTCGATATTTTTCTCTGTTTCAGTTAAATTTCTTTAATATTTTAGTCGTTCAATCCAGTTTTTCTTCAGAATTAGAAAGATAATTATGTCGTGTTTACACTTGCTAGTTGTCATTTTACATCTTGCATGGAATAAGGCTCGACGGGTCTCCCAACCTACATGGAAAACAAACATTACAATTTTACGTCGTCCATGGAACAAGAACGGTGTACTTTTCCAGCCTCAGCGGAAAAAGGACACTGTATTCCTCCATGGAACAAGAACGGTGTACTTTTCCAGCCTCAGCGGAAAAAGGATAATGTATTACTCCATGGAACAAGAAGAGTGTACTTTTCCAGCCTTAGCGGAAAAAGAACAATGTATTCCTTCATGGGACAAGAACGGTGTTCTTTTCAGCCTCAAAGAAAAAAGGACACTGTATTCCTCAGTGGAACAAGAACAGTCTACTTTTCCAGCTTCAGAGGAAAAAGGACTGTATTCCTTCATGGAACAAGAACAGTGTACTTTTCCAGCCTCAGAGGAAAAAGGACACTGTATTCCTCCATGGAGCAAGCACGGTGTACTTTTCCAGCCTCAGAGGAAAAAGGACAATGTATTCCTCCATGGAACAAGAACGGTGTACTTTTCCAGCCTCAGAGGAAAAAGGACAATGTATTCCTCCATGGAACAAGAACGGTGTACTTTTCCAGCCTCAGAGGAAAAAGGACAATGTATTCCTCCATGGAACAAGAACGGTGTACTTTTCCAGCCTCAGAGGAAAAAGGACAATGTATTCCTCCATGGAACAAGAACGGTGTACTTTTCCAGCCTCAGAGGAAAAAGAACAATGTATTCCTCCATGGAACAAGAACGGTGTACTTTTCCAGCCTCAGAGGAAAAAGAACAATGTATTCCTCCATGGAACAAGAACGGTGTACTTTTCCAGCCTCAGAGGAAAAAGGACACTGTATTCCTCCATGGAACAAAAACGGTGTACTTTTCCAGCCTCAGAGTAAAAAGGACAGTGTATTTTTCCATGGAACAAGAACGGTGTACTTTTCCAGCCTCAGAGGAAAAAGGACAATGTATTCCTCCATGGAACAAGAACAGTGTACTTTTCCAGCCTCAGAGGAAAGAGGACCATGCATTCCTCCATGGCACAAGAACGGTGTACTTTTCCAGCCTCAGCAGGAAAACGACAATGTATTCATCCATCATCCATTGAACAAGAACGGTGTACTTTTCCAGCCTCAGCAAAAAAAAAAAAGACAATGTATTCCTCCATCATCTATGGAACAAGAATGGTGTACTTTTTCAGCCTCAGTGGAAAAAGGACAATGTTTTCCTCCATGGAACAAGAACTGTGTACTTTTCCAGGCTCAGCATAAAAAGGGCAATGCATTCCTCCATGAAACAAGAATGGTGTACTTTTCCCGCCTCAGCTGAAAAAGGACGGTGTATTCCTCCATGGAAAAAGAACGGTGTACTTTTCCAGCCTCGGTGGAATAAGGACAATGTATTCCTCCGTGGCACAAGAACAGTGTACTTTTCCAGCCTCAGCGGAAAAAGGACACTGTATTGTACACCATCATCAATTGAACAAGAACTGTGTACTTTTCAATCCTCAGAGGAAAAAGGACATTGTATTCCTCATGGAATGATAAAGGTGTACTTTTCCAGCCTCAGCGGAGAAAAGACAATGTATTCCTCCATCATCCATGGAACAAGAATGGTGTACTTTTCCAACCCAGTGGAAAAACACAATGTATTCCTCCTTGGGTCAGCAGCGGTGTCATGGAACAAGAACGGTGTACTTTTTCAGCCTCAGTGGAAAAAGGACTATGTATTCATCCATGGAACAAGAATGACGTACTTGTCCAGCCTCAGCGAAAAAAGGACAATGTACGAGTATTCCTCCCATGGAATAATAATGGTGTACTTTTCCAGCCTCAGTGGAAAAAGGACAACGTATTCCTCAATGGAACAAGAAAGGCCTACTTTTCCAGCCTCAGCAGAAAAAGGACAATGTATTCCTTCATCATCCGTGAAACAAGAACGGTGTACTTTTCCAGACTCTGCTGAAAAAGGACAATGTATCCCTCCATCTTCCATTGAAAAATAACTAATTTTCCTGCCTGAGGCAGAAAAAGGACAAAGTATTGCTCCATCCTCCAGCAAACAAGAAGGGTGTACTTTTCCAGCCTTAGCTGAAAAAGGACAATGTATTCCTCCATTGAACTAAAGCTGTGTACTTTTCAGGCTTCAGTGGAAAAAAGACAATTCATTCCTTCATCCTCCATGAAACAAGAAAGGTGTACTTTTCCAGCCTCAGCAGAAGAAGGACAATGCATTCCTCCATCCTTCCTGGGACAAGAACGTTGTACTTTTTCAGTCTACACTGAAAAGGGACCATGTATTCCTCCCTCCTCCATGGAACAAGAAAGGTGTACTTTTCCGGCCTCAGCAGAAAAAGGACATTGTATTCCTCCATCCTTCATGGAACAAGAGCGGTGTACTTATCCAGCCTGAGCAAAAGGACAGTGCATCCCTCCATCCTCCATGGTACAAGAACGGTGTACTTTTCTAGACTCCGCTGAAAAAGGACAATGTATCCCTCCATCCTCCATGGAACAAGAACTGTGTAGTTTTACAGACTCCACTGAAAAAGGACAATGTATTCCTCCATCCTTCATGGAACAAAAACTGTGTAATTTTCCAGCCTCAGCAGAAGAAGGACAATGCATTCCTTCATTCTCCATGGAACAAGAACGGTGTACTTTTCCAGCATCAGCGGAAAAAGGACAATTTATTCCTCCATGGAACAAGAATGGTGTGCTTTTCCAGCCTCAGCGGGAAAAGGACAGTGTATTCCTCCATCATCCATGGAACAAAAATGGTGTACTTTCCAGCCTCAGTGGAAAAAAGGACAGTGTATTCCTCCATCATCCATGGAACAAGAATGGTGTACTTTTCCAGCCTCAGGAGGAAAAGGACAATGTATTCCTCCATCATCCATGGAACAAGAATGGTGTACTTTCCAGCCTCAGCGGAAAAAGGTCAACATATTCCTCTGTCATCCATGGAACAAGAACTGTGTACTTTTCCAGCCTCAGCGCAAAAAGGACAATATATTCTTCCATCCTCCATAGAACAAGAATGATATACTTTTCCAGCCTCAGCAGAAAAAGGGCAATGTATTCCTCCATCCTTCATGGAACAAGAATAGTGTACTTTTCCAAACTCCACTGAAAAAGGACTATGTATTCATACTTCCTCCATGGAATAAGAACAGTGTACTTTTCCAGACTCTGCTGAAAAAGGATAATGTATTCCTCCATCCTCCATGGAACAAGAACAGTGTACTTTTTCAGCCTCAACAAAAAAGGGCCATGGATTCCTCTATCCTCCATGGAACAAGAACGGTGTACTTTTCCAGACTCTGCTGAAAAAGGACAATGTATTCCTGCATCCTCCATGAAACAAGAACGGTGTACTTTTCCAGCCTGATGGAATAAGGATAATGTACTCCTCAACCCTCCATGGAAAAAGAACGGTACGATGTACATTTCCAGGCTCTGCTTATAAAGGACAATGTAATTCATCCATCCTCCATGGACCAAGAACGGTGTACTTTTCTAGACTCAGTGAAAAAAGGATAATGTATTCTTCCATCCTCCATGAGACAAAAACTTTGTACTTTTCCAACCTCAGCTGAAAAAGGACAATGTATTCCTCCATCCTCCTTGTAAAAAAAACGTTGTACTTTTCTAGACTCAGTGAAAAAAGGACAATGTATTCTTCCATCCTCCATGGGACAAAAACTTTGTACTTTTCCAACCTCATCTGGAAAAGGACAATGTATTCCTCCATCCTCCTTGGAATAAGAACGGTGTACTTTTCTACCATTCTCCATGGTACAAGGACGGAGTACTCTTCCTTCCTCCTTGGAACAAGAATGACGTATTCTTCCTTCTTCTATGGAACAAAACTGATGTGCTCTCCCATACTCCACTGAAAAGACGTAGTATTTTTACGTCTTCCATTGGACAAAAAGAATGTACTCTTCCACCCACCATGGAACACGAACCCTGTAGTCTTCCAGGCTTGGTAGAAGAAGAACGTCAAGGACGAACCGAGAGAGACAGAGCTCTCTTCACGAATAACTGACGAATGCTTTCCTGGAACTCACAACACCCATTATTTACCCCGTCCTCCAGAGGAACGTTCACATCACATGCATTCTGGGTTCAAATGAAGAGAGTGGTACTCGTAACCAGTTCCTTGCGCTCTTTCGCCACTGGTAAGCAAGAAGAGCAGCTGTCAATGGCGAAGGCGCTCCAGAGCAGAGAGAGAGAGAGAGAGAGGAAATATTTCGTTTTCGTTCTTTTTAAATGAGTTTAATACATGTGCTACAAATGAAAGTTATATCACCCTTGTTGCTTGCCTTTCTCATGGATTTTATATAAAAAAAAGTGATCTGGGCTGGAGGAGAAGGTGTAATACTTGACAGACTTGAAATGGGCAGATGATGCTGTTTTAATCATCAAAACACCACAATTTTCACAAAGCTTTCGCACTAGATGGCATCACATATCAAGAGAGACTGGACACAAGATAAGTCTAAAAAAAACAAATGCAATGATTACTGATGAAATAACTCTAGATGTAGAAAGGATTAACGACGCTGAATCTTTCAACTATTTAGCTACACCTATATCCCAACACAGTTTCTGTTGAGTTGGTATTGAAAGGCTAAAAAGTGAAAATCAAACAATGGGCAGGATGAATAAGATTTGGAAATCATACATACTGAAAATTCATGTAACTGTAAGATTGTACGTTAGTCTAGTGAGTTCTATGTTGCTGTACGAACTTGAATCATGGTATGACATTGAAAATGTAAAAGATATTTTGATTTGATAAAAAAGCTTTATATGAATATTAGGAGTCAGATGGCAGGATAGAGTTAGACATGATACCAAAAGGGAATTTAAGGAAGTTCCATACGCAGATGAGATAATGATGAAAGGGAGATAGATATGGCTTAAAAACGTCCTTCGCACAACAGCTGGGAGAATATTACGTGATAAGTCAGTTGTTCTCCCGTGGGCACCAGAAGAGTTAGTGGCCCAGACCTACTAAGAGAAGGAAGGATGTAGATTTGAAATGAGATTTACGGAAGATAAAGGATAGGAAAGGATTTTCACAGTTTTTACGTCGCGCTGTTGTAGACGATACATATACAGTATGTATATATATATATATATATATATATATATATATATATATATATATATATATATATATTTATATATATACATACATATACATATACATATACATATACATATACTTCAGGACCAAATGATCTTTTTTTTCTAACTTCCTGTTTCTTAAGGATGACAGAAAATTATATATATACATATACATATACTTCAGGACCCAATGATCTTTTTTTTAACCTAAGGATGACAGAAAATTATATATATACATATACATATAAATGATCTTTTTCTAACTTCCTGTTTCTTAAGGATGACAGAAAATATATATTACATATAGTATGTATACACTTTCTGTCATCCGTAAGAAACAGGAAGTCATAAAAAAAAGATCACTAGGTCCTGAAGTACACCTTTACAAACCATTAAATTAAACCAACGACAAGATAAGGTCTCATAGCCAGATGTATGGTGGTTAAGAGTTAGGTGTCTCGACACTATCAGCAATCTGAACTTATAGAAAAAAACTTGTCTAGGATTCTGCATAGCATAAAATGGAAAAACTCACGAGAGTTAGTATATCAAAGGAAACTTTTAGTTTCGAGAAGTCACAAGAGAAATAAAAAAAAAAGAAAGGTTTGCCTGCAAGGAAAGTTTTTGATCTTGTAACCTAGATTATGAGCAATTGAAAGAAATTTTAGACTTTTGCGTATCCAGTAATCTTATTGGGCCGTTCAAGCGAATGTAACTTAATGTTAATTCTGTATTTTATCATTACCTTTATTTACATCGAATATGTGAAAAGTAAGAAATAATCTGAGAGACAGATGGTAGTTTATAAAAATTACTGCACACATCTTGGACAGGCAAATGGAGATAACAGGAAGACCGACATAACTAATAATAAAGAATATTAGAATACGAAAAATGTCAGCAGTGAGATCTTGCCTTCCTATTCAGACCGATGACATCCAAGAACAGTTCTGAATTTGCCGATTTTTGTTGTAGCCTTAACTTGAAGAAACTTCATTTCTTTTTTAAAAGCTAAAAAAAGGGGAAAAAATATATACAGTGCATTAACTTAGAAAATTATGAGATAGAAGACTGAAAAATACCATCATTCATTATCATTTGCTCTTTATCAAATTTAAAGCTAAAAAAACTTTATGGCGAACAAACACATATATACTTAGTGCCAAAAAAACTTAAGAAATGAAACTGCAATTAAATCCTAGAAAAAAGAAAATAGAAAAAAAGTTCAAGGAAAAGAAATCACACCCAAAATGAAGGGTCCAAATAAAAATCCTTATTGGTAAACCCCGAGGCCTTCTCCGGACACTCCAGCCCAAATAGACAAAAAGGTGCGGCGGGGAGTAACAACGCTCTCAACCTTGAAGTAGGGTCACACAAATGCAAGAAAAGTGATCACGGAATGGGAGGAGGGAAATGGAATCGGGTACACGGAGGGGAAAGGGAGCTGGGTGTTGAAAACGAGGGATAACAAAGGGGGAAATGGAGTTAAGGGGAAGTGTTGTCCTAGTAACGAACAGTGGTGTCGTGTCTGGCGTTTTAAAGTCATTTTGGTTAGAGAGAGAGAGAGAGAGAGAGAGACTGGAAAGGAAAAAGCGGAAGACGGTGGCATTATGGGGTGACGGAAAAGAGGAACGAGGAATTTTTGTATTTTAAATGTTGGACGGATACTGGCGAGGACAGAGCTTGAGAAAAGCAAATACATACGTTGCAGAGAGAAAATAAAAAGGAAGAGAGAGAGAGAGACAAACCTCACTTTACACTTCCTTTGACGACGCACCGTGTCAGTCCCTCCATTATAATGATTCTAGTAAAGTGGTAATAGTGGGAGGAATGAGAAGTAGGCGGAGGAGGGATGAGGGAGGTGGGAGGAGGGAGTAGAGTACTACAGTTATAAGCGTAATGGGCGGAGGAAGAACGAGGCAAACATCATGAGACTAAAACTTCCTTTTCTGCGCTTTTTTTTTCTTTTCTTTTTTTTTTTTTTTGCTTTGAATCTCCCCTATCCGCAGTCTTTGGAGTACACTACGTATTTCTCCTCTTCCTCCTCTCTCTCTCTCTCTCTCTCTCTCTCTCTCTCTCTCTCTCTCTCTCTCTCTCTCTCTCTCTGTTTAAAGAGTCTACCATAATTCTTAAAGAACGGATCTGCTTTTCAAGTTGACTCTAAGTGAGAGAAGGACAGAAGACCATGAACGTATTTACGCTAAATTCTTTCACTTCACTGCAGGCACGAACTGAGTTCGAACTGTCTTTCCAGCCTCGTTATCCACAGAATAAATAATGAATCAAGTTAACGGCTCTCCCCGTTTGTTGATCTGAGTAAAAAAAAAAAAACACGTTTACAGGGATTCTCTGATTATTTTAAAAGTAATGTGAGATTTGGTGAAAATGAACTGATACAATTGATGATTTTTTTTTTTTTTTACAAAATTAGTGGTGGTCATTACAGAAAAGATAACGATTTTCTCTTAAAAAAATGAATGATTAGGGGCTTGGCTTTTTACGATGCCGTCGAACTTCCTATGAATTATTTGAAGAAAAACCCGACTATGTACCAAGACCACTGACATTGCATCTTACAAATTGAATAAGCTTATTTCAGTTCGCGTTAGAGAATTAGTCTAAGTAATTGTTTTTCACCTTTTTCTTTCTTAATATTCTTATCAGTTAAGCTAGGCCCATCATGAATGTTAAACTATTGAACACACACACACACACACACACACACACACACACACTACAGACCTTACCCCCAAAATCTAAAGACATTTAAAAATCTTTAAATAATTTTTGAGGCTTCCTTTTAAATGTACGCATGACTTACAATGAGGGAGAAGTGGCCACTCTCTTACAGTTTTTTTTTATCGGACACTTACTGTGACTTTTTGACAAAACCGGCCTTAATTTATTTTTTTAATCCTTCGTATCCGTACATCCCTTGTCTTTGCATTTGCTAGGAATGACAATGACCTATATCTTATTGTATACTCTCTCTCGTATATATATATATATATATATATATATATATATATATATATATATATAAATGATTACTATTTGACTTTACTGTAGCAACACAGTTTGTGAAATCTACTAACAATAGGCCTCCCTACCAGCTCACTGAATCAACCAGAAACAACGTTGAAATATGTGATAAAATGTCGATTTATCTTTTCATTATCTTCTTACCACGTTACAATTTTAGTGCTTCATTACTTTATACAGAATCTTTACTACTTTACTAATAATCCCTTCCCCTCCAAAGGTAAACCCAATCAAACATTCTTTCCATTCTAGCTGCTCTAATTCAGTAAGAGTTGTAAGCTATTATTGGGCATTCTTTTTCTCCTAATTTCGAATATTTAGTTTCCAAGACTTCTCATTTACTCAATTTTCTTACCGTTGTTACTGTAAATTATTTTTCTTCTCTGTAAACAATCTCCCTCGTTTACCCTTTTAATGTTCGCTTCTTCAATTTCGTCCTCCATATCTTACGTATTGAACAACTTAAAGAGTATTGTTCTTCATTTAAGCGTTCATCTTTCGCATTTCCTTCTCTTCTAAATTTTTTGTTTAATCAATACCATAACAGTCCAGTTTTCGCCAGAATATTCGTATTTTCGTGGACATCATTATATTTATTTACATTGTGCCCATAATGATATTTCATTCATGCCTTTCTCCGTCTAGATTCATTAACGAATATCTATTCACGCTGCCTGAACAAGCCATAGCATTGCACATCATATAAATATACATATAACATTATATAAATTCATCCAGGGATCAAATAAATGAAACATGGCAATAACTGTGAAAATACAATGAATGAATAAAGAGACATGAGAAAAGCAAACTAAAAGAGAGGGAGATGACTTAAGGTACTACAAATCAAACATGTGACTGAATGAAAACGCAAAGAGAAACCAAGACAAAAAAAAAAAAAGAAAATACAGATTTACGAAAAACAACAAAAAATTGCAAAATGATCAGGTCAGCCTCACCATCCTCCCCTTCCCCCCTTGGCCACCAGGGGGGTATCGCTCCCTTACCCGAGGGTCCCAGTGAGTAATCGAGCACGTCTGAAATGGAAGAAAAAATGTATCAGTATCTTTGTCCATTTCTCTTAGAAGCAGAAAAACAGACACACAGCCAATAAATGTTGTACATGGATGGAAACTTTCAGTGAACTCATTTACTATTTAAAGATAGTCTTTTAAAACGATAGATGAATAATACCAAACAACGGGTTTCCAAATATATATAATATATACTGTATATATATATACACATATACATATATATATATATATATATATATATATATATATATATATATATATATATATGTGTGTGTGTGTGTGTGTGTGTATGTGTGTGTGTATGTATGTGTGTGTGTGTGTGTGTATATACACGTGTGTGTGAGTGGGGCGCGTGTGTGTCACCATGTAGAAAGACTCGAGATCGATCTCACGGGGTATAAACGTCTTTGGGTCCTTTTCCTGAAAAGAAAAACCCATTTAGCCAATAAAGGTGCTCAGTCGACTGTGGTAAGTTGAATGGCACATGTGGCCAGCAACCTCATCCTAAGTACGTCTTGCTGGGGACTGGAAGCGAAACACCCACCGGAAACACCTTAAAGAAGTGGCGTATAGTGGTGTGTGTGTGTGTGTATATGTATATATAAATATATATATATATATATATATATATATATATATATATATATATATATATATATATATATATATTTTATATATATACATACTGTATATATATATATATATATATATATATATATACATATATATATATATATATATATATGTATTTACATGTATATATAAACTTAATCATTTATTATCAGTCAAAATTCAGTATGAGAAAGATAATTTGTTACAGGAAAGATTATGATAATATATTAAGTTCGATACAGCGCAAGGCAAAACAAATTAGGGAATCAATATTGAATTGTTCCAATCATTTTCTTCTGACAAGCCGTATATCGTACAGAAACCCCACTAGTGCTTCTCCTAGCTAAGAGCATAACAGCCTTTTATTGAATTATAAAAGCCAATCAGCAAGCAGTCGCGAATTAATATCAAAATAGGCGAAGACTATAATATTATATTTTGTCAACTGTATTTAAGTTTCGGTTGGAATCCTATTGGATTAGTATGGCTCTTACTGACTTGGGGAACGGTCTTTGGAGTGTTTGGAATTATTTAAGGAACGCGCACACTTATGCTCGTATATGACATATCTTAATTCAAAGCATACATTTACTACCAAGCAGTATAAGAATTTTTATAAGCTAAAATATGGGTGACATTTATAAGAATTTTATCCTTTATATAACAGGAATGCTATGCATAAGAATATTGTAAGAATTATGTAAGCTATAACATGGTTGACATGTACAAGAATTTCATTAGTTAAAACATGGATGCCAAGTGTAAGGATTGTATATTCTATAAAATGGATGTCACGTATAAGAATTGTATATTTTTAATATGGATATCACTTGTACAATTGTATATTCTATAACATGGATGTCACGTATAAGAATTGTATATTCTATAATATGGATACCATTTGTGAGAATTGTATATTCTATAACATGGATGCCAATTATAAGGATTGTATGTTCTATAACAGGGATGCCACGTATATGAATTGTATATTCTATAACATGGATGCCACGTATAAAAATTGTATATTCTATAACAGGGATGCTACGTACATGAATTGTATATTCTATAACATGGATACCATGTGCGAGAAATGTATATTCTATAACATGGATGTCACATATAAGGATTATGTTTCCTCTAACATAGTTGCCACGTATAAGAATTGCATATTTTATAACATGGATGCCATGTGTAAGCATTTTATCAACTATAACAGAAGAGATTTATTTCTTCCACGGTTAGTCTTCGACGGCGGATATCGCATTGCAAATCGCAACAAAAAAAATGGCACCGTACTCAGAAATCATTTCGCAACAACAGACTTCTGAGCGACAAGACTGACCTTTAATTCCCTTCGTGATGGCGTAACTTTTTGAGTCATGTCAAAAATAGATTTGAACAAAATGTATTTCGCATTCAGTATCACTGCTCGTTCCTGTGGAGAAGAACGTTTAACTACTGTTAAAATTAACATAAATTTTGCTAGAACTTCCACTTGAAGTTCTTCAGTGTGTCATACGGTGCGTAATTCTTCTGCTGAAAAAGTAATTCAGGCATAAGCTTGCCAGGAACTGCAGAAAACACGACTTCAAGATTTCCTTCAAAGTCATTTCTTCAAAAAACAAAGCTGTAACAGCCTCATGTTTATTTATGAATCGAGCATGATTGCGTGTATAACCACCCCTGAAGGTGAAATGGGAAAAATAAATAAATAAAACACTTCAAAAACGTTATGGAAGACTCGTGCTTACGAATTTAAAAGCAATGTGGGAACGACTCCAAAAGCGTAAAACCAATTACTGAGAATGAAACCGAGAGTAACTGTGATCAGTATTAATCACTGTGACGTCATTATGCAACCGAGTATTGTGAAATGATATTTTCATGTCTGGAAAACTGGAAATCATATAAATAAAAAACGGTCGTAATGATACCCCTGTGCAGTTCTTAAAAGTATCAAGTTAATGTAATTTGCTGAGAAATATAAGATGAATTTCAATCTGTCGGGTTTCCGAGGTAATGATATTGCAATAGTTTCATACATACATTGATAAAACTCAATCTAACAACAGTCGTTTGGTCATAATTCAGTTTTTTTATGTTTATGCAGTGATTAGGAATACGGGAATTTAAAATCCAGAAGTATTTGATAGACCCCAGCCTGCTTTGTTTAATGTTAGTTCGTTTTATCCATGCTATCCTTATCTCAATTTACAAAAACTATGATAAACACTATATCCAGTTTTAATATGATTATTTTTATTGCATGGAAAAGACTTTAATGAATTTCTCAGGAACTTTAAGCACCTGACTTTGCCATTTAATTATTGTTGTTGAGAGTTTATATGTATCCCCGAGTAATGAAGACTGAAACAGTAGAGGCAAAAAACAGAACACGGTATATACATTACTGACATATCACATCAGACACCTTATAACCTTCTTTCCTTTAAGGGGAAGGAAAGTCTGTCGATTCCTTGTACCCACCCCGCTTCTTGTTTTCTTCGGGCCTGGGCTACAAGCGGGCAATAAGATCCCAGTCCCATGGACCTTTGTTATTAATAAAAAAAAAAAAAAATATATATATATATATATATATATATATATATATATATATATATATATATATATATATATATATAAAGAGTATGTTCTATACTTTGATTGTTCCCATATCTCACGGATACTGCAGATTCCAGATTACTTGACTGTTGGTACAGCAACACTTTTTTTTTTTCTCTTGCAATTCCTTTATTTAAAAAGCTCAGGGGACACTGTGCTGCAATATATTTCAAAGACGTTTCAAATTTTTCAAAAACCTTTTTTATAACATATGAGTACACTGACGCGACCGAAATATTATTCTTAAAGATTTGTCTGTCTCATACAGTTATGAGATTATATTATTATCATCATAATAATCATCATCATCTATGCATGCAGTTTCATATGGAAGAAACGAAAAGGGTCATTACCTTCGCAAATATTTCACAGAAAGGAATTCACGTAATGATTAAAAAGAATGAAATTCTACAACAACGGTTTACGTCAAAAGTTCCATGATTTCGAACAAAGGAATTCGTTGCATCGAGTAATTTTGCCAGTACAAAAAACATCAAAGATATGAAACATTAATACCACCATCTCTCTGGATATAAGCTTTTTACATTCTACATTGCTTTTTGGCATCACTGAATGAAAACTTTTTAAAATGCTGTAAATGAGACCAATTGTAAGTGAAGGTTACGCCTAATATTGTTAAGTACTCCTTATATTTTAAGGCTGATCACTGGTCTTATTTTCATCCCCAGTCATCATCATACTCAGTCGACTATAACACTTGCAGATCAACTGCAGGTCTTTGCTAAAGCTTTCAGGTATCTGCTTTACATCAGAGAAGACCAAATAAAGAAACTAGGCAGGCAAGGCAACAATCATTTAATAAGTACGGAGACACAATGCCTTTTAACGCTTATGACAAGCCATCGACACCATCGACAAATTTTTATTGTTATTCTACAGCAGAACAAAAAGTTACGAAATTTGTGAATAGGAAACGTATGATTAAGTAAGTCGAAGTAAATGTCAAAATAGATTTGAACAAGTCCAGACTCATTCATTACCAGCATGGAATGAGATGGATCTTGTTAAATATAAAATGGCATCACAATCTCCTAATTATCTCCGGCCTACATGCGGGCTATTGAGACGCATGTCTGGATTCATGCTGGCCTGCGTAACTCATTTATACATTTCCTCATCGATTATGCCAAAAAGATCTGAACACCCAAGTTTAGTGCACAATTCTAATTTCATGTTCTAAATATGAGACCAACATTTTGAACAAGAACCACTAATATATGCCAGCTCTGTTGAACAACCGCGGACAAGCCGTGAACTTATCCTTCTTTGTAGTATATTCATTAGATAAAAGAACAGCTGAATAAACAAAGAAAATATCACTGACGGGGAAAATTAGCAACTACTTTTCAGAATAGGGCTATGAACGATGACAGAGAATCACAATGTCAAAGTATTTATTTAGTATTCTGACTGAAGTTGGAAATAGCATGCAGGAATTTAGACTCATTTAAATTCCAACTACATTCATAAGCTGTAAAAGAATAAATACTATTTTACCTCACTCTGCGCAGCAAAACATTACATATTTACTGCCAATTTCAATAAGCTGTATTTGGGAATCATTACAAATAATATCATAAATAACGAACAGATTTAATTTAAACTTATTGCTTTTAAATAGAATCTGCAAACATAATTCAGTGATGAGTTTTTGTTTTTGTTTCTCTGAGCCATTACGGACAATATCCGTTGTCTAGTTTCGCACAAAAGCTTTCCAGCAATGTCGGCTGATTTTTTCGAAAATGCAGTAATTATCACTGAGAACTCTTTTGCTGGTATTATAATGTCTTCGTTCATTTTCCAAAATCATTCGGAACAACAAAGGAAATTGTTCTGAAACAAAGAATTGCTTTTATAAACTTTTAGTGTCAGTTTCTTTTCAAATATTATTGATTTGCCGCATTCTGATCAAAATGAGTGAATCAACGTCAACACGATGTGACGTTTCAGTTTCTGAATTTTATTTAGTGAACGGCTCCTTCTAAGACTTGGTACAGACTCCGTTTAATTAGTCGAGTATCGGACAAAGTTGAGGTAAAAGAAAGTCTTTTGCAAATATGATACGAAATGGGGTCCATCAGATTCAATGAAGTTACGCCCTGCTAATCACGTAGTGTCCGACGGAGGTATCAGTAATACTGTAACTGAAGATATATATACCTGTATATGTATATGTGTATATATATATATATATATATATATATATACATATCTATATATATATATATATATATATACATATATGTAGGTATATATATATATATACATATATATATATATATATATATATATATACTATATATATATATATATATATATATATATATATATATATATATATATATATATATATATATGTAATGTGAGTCTGTGTCTGTATGTGCATGCGTACACCAAGAGTAAACATGTACACAGAATTTAGGCACAAGGAATAAAATGGTTCACCAAGAATTTCTGTCCAAAAGCCGAAAATGGAAATTGGCGTAACCCGAGCGAATTTGGCAGGACACAATGGGAAAAGGGGAATGACGAGAGGATACTGAGGGGAAATAGCTGAGATATGGACGGCCGAGGGGAGAGTTGGGGGGAGAAGGGAATCGGACACAACACAAGGGTGAAGGTGATAAGAAGTGGAGAGAGAGAGAGAGAGAGAGAGAGAGAGAGAGAGAACGTTTTAAAAAGTTTGTATGATTAAAATTCATATGTAACAGAAGAGGAAATATCCTAAATTAAAATTGATTTTATATATGTATATATATGTGTGTATGTGTATATAGTTTATAAATAATGATATATGACATTTTGATATGTATATATATATATGATATCATTATAACATAATTTCATATCATTGATGTTTATTTTATTCCGGTTTAATAATATTGCACTAATAATTACTAAAAATTGATGATTGTTCCCACAAAACATTACTTATGCAGTAACAAAAAAAGAAAAAAGAGATAAAAGAGCAATATAGCAATAGCTTAATAAACTAAAAATGTGTAAATATTACAAAATGATTAGTAGTAAGAGCACTGAAGAATATGAGGGTGTACACAGACACACATACACAGGTGAATCATCTTCAGTTCAGCATTTCATCTGTGAATGTGACAGTGACCACCGTCTTATTTTTTCTCCGGAGCCATCCTTATTACGAAAAGAGGATTAATATATATATATATATATATATATATATATATATATAAAATATGTATATATACATATATAAATATGTATATATGTGTGTGTGAGTGTAACCACTGCTCATGATTAATAACAAATGAAAATAAATTACTGTGAAAAGGCCCTGTTAGAGTCAATAAAGGAACAGTTGAAAGCGAAAAAGGGCTGGAGGTCTTTGGTCAGACACGACGCACCCTGGCCAGGGCGGAAGAGGGGTGGGGTTTTGGGGGGAGGGTCCAAATAGGAGTTTCGATAGATAGGGATCAGAGAGTGTGAACAGGGGGTTGGCATCCAACGCGCCGCGTTATCTTGTCCCGTGGGAAGATTACGGCCTATAATCACCCTCGTGACATGATTGTTCCCCCATATTTTCCTTGTTTGGGTCGTTAGACACAGTGATTAGCAGATTACTATGACTTGACGGACGCATTTGATATGCGCAGTTGTTTGAACGAGCCTTCTTCACATTGCGCTGAATGCGCCTTTTGGAGATTTTCTTTCTTAATAAGAATTGATATTCTTACGATATCTGTTTTTGATGATGGAAGTTGATGATCAAGAACGATTTATTAACTTACATATTTAGCTTTTCCACTCAAACTTTCGGCTGCATGTCAACACAAAAACCTAAATTGCAATCACCTTACAGATCAAATAACAAAGTCATTTATTTATACGTCTACGAACATCAACAGCACCTAAAATGACAATGAAATTAGTTATGATAACAGATAAAATCGTACACTTATCCTTTATTATGATAATGCTACATGTATGCCAAGAGATCTTAAAGACCAGTGCTCTTATGATGGCGCAAGAGTAAACGATTAAGAGAGAAGGACTTTCTTATCATCCTTTCACCTTACCTTCTTTCTGCTTTCCCGTCTTTGCCCATGACCTGTGCTCATTTCTTTATATGTTTTTAGGTCCTAAGCGAGAAGCAGAAAGATTTGTGACTAAGGTTTGATCAAGAACACTTACGAAAATTCGAGGTCTTCTGAGCGAGTAGCAAGAAAGATTTATGACTAAGGTTTGATCAAGAACACTTACGAAAATTCGATATTTGCTAAGCAAACGGTAAAAAGAACATTGATAGCTAAGGATCACAAAGGAATCTACAAAAAAGAAAAAAAAAAAAAAAAAAAGCGTGCCAATCACTCAGATAATTAAAAATTATTTTTCTGCTGGTTTTCTGCACAAACAGAGAGAGAGAGAGAGAAGGATTTATTGTTGAACAAGAATCACCACGAGCGACACGTGTTAAAACGCACGCAGATTCATTTCCAAAAGGAACTTCTCACGAAAATAATTTTAACTCAACCTTTCCAAACCCTGTGGTCACTTATCTAAACCCTTTAATTTGATCTGAATCAATAGTTTACGAAATAAATTTAAAAATAACGAAATGAACACTTCAAATTCAAGACATTTTGTTACCCTATATTCAACATACCGATTCGTATTGTTGCGAGATCCTGTTAAGACGTTGGCTTGTTTATTAAATAAGTTCGCTGGCTTACCAAAGTGAACTCTGACTGGTCTGCAGAAAATAAACAAGTTCAGATTATGCATTATTTGCACTTATTCTGCCTTGCCATGTCACGTACATTCTTCTCATTCTCACAAGTTGCAGGAATATCTCATGGAAAAGGTCGAAAACCTATAACAAAAGTCTCTCTCTCTCTCTCTCTCTCTCTCTTCTCTCTCTCTCTCTCTCTCTCTCTCTCTCTCTCTCTCTCTTCTCCACCATCACAATCATTTGAGCCTAGCATCCGGGAAGGGAACTAGAGAGAAGGGGAGGAGGGGAAGGTTGTAAAGAGGTGAAGGCAGCAGGAGGGTGCGGGGAGAGAGAGAGAGAGAGAGAGAGAGAGAGAGAGAGAGAGAGAGAGAGAGAGAGAGTCCACAGGGTGACGTGGTGAGGTCCTGTAACACCACTACACCACCAGAGGTTTTAGTCGGATTAATGATAGCCGATGTTTTCCCCATCTGGAAGATTACGGTCACGAGATAAACCACTTCACGTTGGAGGTATTCCTTTGATTGTTATCAAGATATAGCGTCTTTGGGCTCGAGAGAGAGAGAGAGAGAGAGAGAGAGAGAGAGAGAGAGAGAGAGAGAGAGAGAGAGAGAGAGAGAGAGAGAGAGAGATTTAACATCGTCAATATCTAGACATTGCTGAATGGACAGATAATAGAAGCTCATTTTTAAAGTTGGAAATTCATGAGCAAACGAGGCAAACAAAGAAAGGAATAAATGCTGGCACAAAACTCTTGTAGATAATAGGACAAGAAAGAAAAAAACACACATGGTGGTCAGCGAAGCGAAAAATTGAAAATTATACAGTGAAAAACAGTAAACAAATACATAAAAAGAGTAAATATCAGGGTATAGCTTTAGCTTTTAGGATATATTTGACTTTCCTGTTTTTCATCTTTGATTTTCCTTGGTTATGAAACGGAGAGAGAGAATTGCTTAAATGGTTAAGTCCGAAGCTTTATCAAATCAGTTGCAGAGTGAAGTCAATTGACTGTGCAAGATAACCCAGCTGCTTCTGAAAAGGCGGCTTGTGTGATGTTATATTTATTTCCATCAAAAATAACGAATCGATCGAGGAGGGTTGGCAGTAAGTTACACGGTTTGATAACAGATCCTCATGTCGATGTCAAAAGACGAATTATTCCAATACTGAAGCAATATAATGTTTTGCTTGAAGGACCTTATCTGGCGACGACACGAACTCAAACGTATGAGGATCCCGTCCTCTGTAAATCTCTCTCCTTGAGACTCCTTCACCTGGAACTTCAAGTTTAAATCCACCTGGTTTCAGCAGCCCATTATCGTCTGCGGAATCTACAAAGGAAGCTGGGAAACTGGCTCGGTTTTGTTCGCTTCAGAGGGACATATACAATCACAGGGATGAGGAAGTGATTCTAAATTATATATATATATATATATATATATATATATATATATATATATATATATATATATATATATATATATATATATATACATACATATATGTGTATACATATATATTATACATAATATATATATATATATATATATATACATATATATATGTGTATGTATATATATATATATATATATATATATATATATATATATATATATATATATATATATATATATTGCTGAATAATGTAAGAATGCGAGAATCACAACAAGAAATAAACATTAAGTAAGTCAGCATGTCTAATAGAATAAAATTATACTGCAAGCGAAAGAAAACAGGTACACACACACACACACACACACACACACACATCCCTTCCCACTAGCGCCCATCTCCACCCCCCCCCCCACATCTACCCTCACTTTACAAAAAAGGATGAAGATAAATATCCAAAGGAAAATATATACCTGTTTGGGACTATTTCTGTGAGAGTCTAATCACATAAAATCCTATGATTATAGAACAGAGGGTGACCCCCGGTTGAGGGCGTCCTGTAGGGCGGGGGGGTGTAGGGTGTTAGTGGGCATGGGCAGGAGGGTAGGTTTAGAAGGGGTGGAAGGAGTCTGAGGTGTGCTTATTGTGAACTGGTTTATGAGATTAAGCAATTTACAATCCCTTCGTCCCCAAAACCAGGTACTTCCTTTGCTGGATTAACACGATTTTCCGGCATCAGAAGAAACGAAAATAAATATGCAATGAGAATATAAAACGTTCAAACAAAATAACAAAAGAATTGAGGCGAACTTGAGAAAATTGTCACTATTATTATTGTGACGATGGTGATGATACTCCCAATATTGTAAACAACACCAAACAAACAACTTTTGATAATTAGGCTTTTTTCCCACGCCACGGCAGAAAACTATCATTAAATTTACCACAACAAAATAAGTAAAAGGGAGAAAAAACCCGAATGAGATCAATCATTTTGTCAGTAATGAGGTCTCTAATGCGGGTACTTTTCGCTCCGCCAGAAAAAGAACTCGATTAATTTTACCCCAACTCGATGCCGTTACATGAAATGCAAGGGGGAAAAATGTCCTCTCTATGGTGCTGGTCAAAAATGTCGCCGCCTTTATTATAAGGACCCAGCGCTGCTAATAATCATTTGGGCCTCGGGGGTGGGAAAAAACCGGACTTATTAGCGAACCAAAATGGAGCATTTTACGGCATCCGTGGCAATTATCGAGTACAGACAAAACGCTTTATTTACAGTGCGCCGTCACCATTCGTTATAATAGCACGTGTCGTAAATATATATCGCTTTTTTTTTTGACGCTCGACGGACAGAAAAAGTCGTCGTTATTAATTGAGTCAATAATAACGCGGCTATTATTAATATTTAAACAGCGGCGTGTCAAGTCTGGGAAGTTAAGACGTCATTTCTTAGAAATGCAGGCTACAGACACCGAGTAATTATACTAAGTGATTAAAAATTTTATATAAAAAACATTCAGTGAAAGAAATATATGAACTTGTGTACTTTACAGAGTAATGATGGCATACAAATTTTATCTGTATGCCATATACTTTCTGAAACACATTTCGGTTCTGCTTGCTTTATAAAGCAGGTACTGTATATAGTATGAATGTACAAATGAACGCCTTTATTTTTGGTGGGAAAATCTTATAACTCAAACCAAAGTGTTCCAATGAACAGAAAAGAGAAAACAGTCCTCTTTCAAAAACTTATAAATCTTTCTCACAAGAAAACCATAGTGAGAAGGAATAAAGAAATGAAATATATAACTTTTTAAAAGATTATGTTCATGTCTTTCCAAAGATTTATTAAGTGCTAATCAGTGCTATAATTTCCTACTGAAGAGATCGAGGAACGTTGAATTTCAGAACAAAAAATCGAAATTTCAATTTAAATCTTTTGCAATGCGGAGAAAATGATTTCAATGGCAGAGAAGATATGGTGAAAAAGTTTGCAAATTTTCGAACGAGAGAAAAAAAGAGTATCTTGAATGAAATTAATTAATGGAGAAAGCGTTTTGTTTTCTTATATGCAAAATTAACAGTGATCCTTAACTTGAATCGACTCTTTATGAACCTGATGACAATTAGTCTTTACCATATAAATATGAAGACTACATGAAAGTATTACGAAGGAATGAAACGACCAGATTATATTGTATTATATTATATATTATATATATATATATATATATATATATATATATATATATATATATATATATATATATATTATATATATATATTTTTTTTTTTTTTTTTTTTTTTTTTTTTTTTCGGTTATTTATTACAGTCGTCCCCGCCAGAAAAGTAGGGTTCGATTCCCAACCGAGATGGGCTAAACTTGGGCCAAGCCCACTAAAACACACTATGCTTCTGTAAATTATGTACTTGGTAGTCAGATGATTATGATGGGTCACCATAGTGAAGAAGGGCATAAGGATAGCAGCTTCATTCAATATACAGTAAAAAAAAAAATACAGTATATATAAATATAAATAAATAAATAAATAAATACATAAATAAATATATATATGTGTATATTGTGTATATATATATATGTATATGACAGAGTGTGTGTGTGTGTGTAGTGTATGTATAGAGAGAGAGAGAGAGCGGCGTGCGTATCCTGTAAACATGAACATATTCACGTTCAAGCGACTGTATCATGGAGAGGTCCCAAAGGGGATTAAGCGAGGGGTTTTAGACTCCTTTTGTGAGGCGATGCTTGGCTGGAATGCGAGTTGCGATGAACCCGGGAAACGACGGCTCGTACCGCCCCATTCCGGGAGGGAGGGCGGAGGGGGATGTAGGTGGTGGGGGTAGGTGGTAGGGAAGTGGTATGGGAAGGGAGGGAAGCGGTAGGGTAATGATAGAGGAGGGGAGGGAAGGGGTAGGGGAATATGAGTTGAGAAGGGTACAGAAGGTATCTTTAGTATTGTTTTAATATCTGTTAATAATATATATATATATATATATATATATATATATATATATATATATATATATATATATATATATATATATATATATATATATATATAATTTATATATATATATATATATATATATATATATATGTATGTATTTTATATATATTATATTTATATAAAATATATAAAATATAAATATATATAATATATATATATATATATATATATATATATATATATATATATATATATATATATAAAGATAAATATGCTGGAGTTATATATTTATATCTAGTGTCGAACTATATATATAGATTTAAATATCATATACATTTCCATATTTTATTCATATACATATATATATATATACAATGATATATATATATATATATATATATATATATATATATATATATATATATATATATATATATATATACAGTATATATGTGTGTATGTGTACTAAACTAAAGTTGCTTTATCTAAAAAAAAAAGTATACTGTTATTGAGGTGGTAGTCAGTGCTAAGAGCAGCACACTGCTGCTGTAAAAATTGCCATTAGCTAGGGCTAGTAATGCACTGTGCCACTTGGTAGAGGAGCGCCCATTGGAATGTACTAGATTTTGTTAGCGGAAGTTATCATCACATAAATGGAGGATGAGCTTCTTCCATAAGGTTGATGACAGTCTCAGTTAATTTCCATAATATACGGGAAAAAACACACACACAACATTCTGACAGGTCTCTGTGGTGTAAAAGCCACCAAAAGTCCGGGAACATAGGGTTTGAAATGTTGGATGTCATGGTGCTGAGTAAGATCAAAATGAAAGGGCAGGGTGTTAAGACAAGGAGGAACTGGGTGCGACTGTATGGGGTGTTGCTGAGGCAGATAAGTCGTCAATATGAACGTAGAAAGTGGGGAAAGAGCACAAACAAGTTAATCCAAGGACTGTCAGAGGTACAGGTCAGTAGTACAGGGAAAACTGTGACCGTAAGTGGATGTGGTCCAGGAATGGGAAAAAGTGAGGCCGAAAGAGAAAGTTTTAGGGAGTGTCTGAATTTGTAATGGTTGAGTTTGGGAAAGAGAAAATGCGGTTGTGCTGTGTGATATTGATGCAATGGTAGGTGATAGCAATAGAGATGGCACACTTGGTAGATTTGGAGTCCATTTGTGAATAATGATGTCGTTTACAAATGTGCTCTGAAAAGGGCGTGATCGTTGGAAATACTTGGAAAAGAAAAACCACGAGTAAAAGAAATATTTGAATTGTATGGGACTGCAGAGAAGTTGGAAGATTAAACAGATGAACGTAGTGGTGAGAAGAGGGGAGGGTGTAATGTATAACTATTCATTATATTGCTAAAGAAAGGGGGAAAAATAGAATGTAGTTTTAGTTGAAGAGGAAGTGTTAAGGGTTCTGAATGTGACTATAAATGTAATTACAGTGAGTTTGATAAGGAATCAATGATAACAGCATATAAGGAGATCATCGGTGGCCAGTTGGACTATAGCTAGAGGTACCAAAGTGAAAAAAAAAGGTTATGATAAGGAGACAAAATATAAGGATGGGGCATTCAGTTAGTATGGGAATACTTGCAGGTAAACGAACGTAGGAAAGAGCAACAATAGAAGTAAATGTGGGATGATGATAAGAGTATTTCGTGAAAGAAAAAAAAACCCGCACTGGTTTAAGTGCAGTTGCCGACAAAGGCGATAATCATGAAGCAACACGCAAGAGGATGATGCTTTATATTCAGGAAGAGAGAGAGAGAGAGAGAGAGGAAGAAAGAGAGAGAATAAACACACCAGATAATTTAAGTAGACAATGCTGTATTATAGGAAAGTGAATGCCGAGTGAAAGGTTAATGAGCGAATGGATCTTAGAATAAAAGATTTGAATGAAGGAAGGCTTTCTGAAAGGAATGCACTGTTGGGTCGTTGTATAAAGAGCTGATGTATAAAGAGTAAAGAACTGAGATGCAAAACTAAAATGCAGTAAGTTGGAAAATAGAAAGGTGCAAAAAATTGACAGAGGTTTAATGGACATGCTGCAATATATTGGTGATCGAGTAACTGAATGGCATATCAAATGATAATGTTTATAAGTGCACATTAATCATCTGCAGAAAAGGTAAGGGGTTAGAGGTGAATATTGAGCTATGACATTACGTAGATGGGGTTATTACATTTCTGGTAGAGAAAGTCAAACTGATATCAGAAGGAATCATTGGGAAAGGGCAGCTTGAGTTTTTAGTCAAGGACGAGAATTTGTGTATCTCGTGTTCATCATGAAATGTTGTAAAAGACATTTAAGAGAAACGGAAAAGTTAGACATAGAGGAAGCTTGTGATAGAATTCAAAAGAGAATGGAGGGGGTTGAGGATGTGTGTTGAAGAGAGGAATTCATTGAGAGTAATTAGGGGCTTTTGTAATGAAAATAGACGTGTATGGCTAGTTCGGTGCATAGCCTTTTTGTAGCAGGAAGCTGCCGTTTTGACATTTCTTTTACTCCTTAAACTCAGGGCATTCTCTGTCACACTCTTTACATTCCAAATGGACACATATAGACAAGAATATACAGTATGTAAATATATATTTTTTTTATGTTAAATTTCCCATGGCTATGTAAAATTTTCATGGATACAGTGATGACAGGTCTTGGAAAGGAGAGATGTCGGTAGGAAGTCACAGGACCAGGGAAGGGACCGTTAATGAAGCTTGCAATAGTTGATGTTTGCTGATGATAGGGTTAATTGGTGATAGTGAAGAGAAACTGAAGAAAGTGGTAAAAGAGTTAAAGCTATTTCCAAAATGAGAAAGCAGAAAGCAAATATTGGTAAGCATATTGGTAAGAAAGTAGCAGGGTCGCTGCGAAACTTTTAGATGAGATGAGTAGCTACAGAAGCAAATGTTGTTGTGTATGAAAGGGATCATTTGGAAGCCACAGAGTTTAAAGGATATAAGTGGAACAAATAAAAAGAGCTTAAAAGGGTGAGAAATGCAGAGTTAAAATGAAGAAATGCTATATTTTTTATAGGTTAAAAAATCAATCAGGGTATTTTGAGGAAGTCTGTCACATGAAGAGAATGAAAGATACCAGGGTAGTGGAAAAGTGTGTTAATTAAACGATTCTATGAGGAGGGAGGAGAATGGAGGGCGATACAGTACTGAAGAGACTGAATGAGGAGGTACCGGAACTTGAGGACCTGAACTAACGTGCAGCTACACAGAGGTGAGTGGCGCACTATATATTTGTAGCAAGGAGTGCCAATGCACTAATGATACGCTTTTTGTGTAAAAATATGAAGCGGCACAAGCTGCCATTTTATATTTTTGTGTACAGGTTATTCATCCTTAATTTAGCAGAAAAATGCGAACGTTGTAATGCTTCCTTATTTGTTTCTATTTCCTATAATGAGAAAGTGATTATTTGGCAAATTATATATATATATATATATATATATATATATATATATATATATATATATATATATATATATATATATATATATATATATAATATGTGTGTGTGTGTGTGTGTGTGTATATATACATACACACATACACAGATATAGATATATATACGTTTAAATTTTTTTTTTTTTTTTTTAAATTCAAGCAGTTTCGCCTAAGAAGGAATGGATCCTGAGAAAAACAAAGTGGTGTGGTCACACAGCCAATGTCACATTCATACTCTAACTGATGAATCATGAATTATACACACACACACACACACACACACACATATATATATATATATATATATATATATATATATATATATATATATATTTATACTATATATATAGAAAATACATTTATATGTTTATATATAAATATATATATATATATATATATATATATATATATATTATATATACCTATATATATATATATATATATATATATATATATATATATATATATATATATATATATATATATATTATATATATATAATATATGTATGTATGAGTGTGTGTGTGCACGTTTACCTGTATTGACTTATTAAGTCTCTCTCCTCACTTAAATTACATGCTGAAATACATGACCATTTACTTTCAACACGTGCTACCACTGTTCGAGATAATTGTTAATATACTTTTTCCAAAGCCGAGTTACGGAGATATATAAAAATCAGTCAACGGCTTATCACTAGATATTTATCATAGACTTACTTGGCAACATGAGATAACGTAAGACTTACAGAAATACATTTCAGGCTACATGAAAACATAATATCAAAATACTGAGGATAAGCTAAGGCCCTTTTTGTCAGTGCAAATCTTAGTATATTGTTAAAAATACACTTATTCTTATTTCCTCATTGTCAGAGAAAAGAAAAATAAAAGATTTAAATATTCATGCCTAAGTTCATACTTACATACTTGCAATTCTAAACATCAAATATGAAAACATTCTCGCAAACTTACGCCAGTAATAACAATTACAACAATATCATTAGAATAATGCGCGTTCTAATTACAAGAAAATAACTCAATTACGGCCAAACAATAGCACATCACTGTCCCCAGGAAACAAACAAATCCCGCATCAAAAGAGAGAGAGAGAGAGAGAGAGAGAGAGAGAGAGAGAGAGAGTCATCTCCCCCTACGTGTCGACAGTGGCAAAGATTCAGATACAAAAGATAACGTAAGACTCATGAATAAAGATAAAGGGGAGGAGAGAGAGAGAGAGAGAGAGAGAGAGAGAGAGAGAGAGAGAGAGAGAGAGAGAGAGAGAGAGAGAGAACGTTCATATCGTACAGTGGTGCCCTTTTACTCTATCACCAAGACATGCTTTTGGATACCAGACGTACGATAATTTGCATGCCTCCATTCTTTTCGAATCTATGAAAATACCGAGGCCTCTGTATTCATGTTCTTTTATAAGATGCAAAGAGCCTTCGTTACCAACTCCTTAGACACATCTGAGTCTACGTAGCTTCTTTTTATGAAGGCAAACCAAGAAGCTATAAATGCAAAGGAAGCTGCTATATCTGGTGACACTCCGGGGAATGTAATAATTGTAATTCACACATCCACACATTCGTAGGAGATGCCGTATTTACTCTCTCTCTCTCTCTCTCTCATTAGTAACCCAATTCACATTTTATCGGTCAGGGTTGACTTGAAAGGCAAAGTGACAATGCAAATAGTTGCGTTTTATGCAGGATCATAAAAATGATGCATAAGGCTCATAAAGGCATTCGAGCGTCGCCGATGATCTCGGCACAAAGAAAAAAAAAAAAAAAAATCGCTCCATAATTTACATCACAGACAGGCACCCAGGTACGGCACTAGGATGTGACGACTCGTGGCTGCGTCGCCAGATAAAGCCTAACCAATAAATCAATCTCATTGAAAATATGATATATTTTTTTTCCGTTCGCTTAACACCAATTCTTGCCTCCTTCACAAATAGTTATTGTCTTGTATTGATTGTTCTTTTTCGACAAGGGATGAATGAGCTGTCTCAGTTCCCACTGGCATAAAACTGGTACTTGACTAAAATCATCCATACTTTTCACATTCGATATGAAGGCATCTGGGGATACCTAAATTTATATATAATATTTTCTACGATTTTTCTATATAATTTTTATTTTTCAGTGTGTGTGTGTGTGTGTGTCACAAGGCATAAAAACTGGTACTTGTTTAAACTACATCCATTCTTCCATTTATATATGGACAAATAACGTACAAAGATACTGTATACATACATATATAATGAAACAATTTTTTATATTATATATTTATATATATAACCCTATATATAATATAGAAAATATTTTACTAAATTAGAGGAAGGTTGTGTGTGTGTACTGATGTAATGCATATACATATAAAAATATATATAATAGAAAATAATATATGTACATATAATTATATATATATATATATATATATATATATATATATATACATATATATATAATGAAACAACTTTTGTCTTTTCTTTAAAAGCTTTAACCTCTATAATAAGACAATATTTTACTAGATTAGAGGATATTGTCTTAACTCAAAATATGCCCAATGCATCGACATTAAAAATAATTAAGAGAAAAAATATGTATATACATAATTTTATATATATACATATACATATATATATATATATATATATATATATATATATATATATATGTATATATATGCACATGTGTTTGTATGTATCTGTACGTTAGTACAAAGTCAGTTACTAATTCTCAACATGGTGCTTTGTATTTTCAAATATTACAACAAATAGTACACAAAGATGTCGAATTCAGTTTACCTTGAAAACAAAGCTTACAAACAAAAAGGGGATTATAATGATAACTGCAACTTCCTCAAAAAGGATTCGAACCCATGTCTTTATGATTGGTACGTAGGAGACAGTGACTTTGCACTTTTGATGGGAGAGTGTAATAAGTCACTGCCTCCCGTGGCAAACATCAAACGAAACGAGTACGAACCCTGAAAGGTGTATGAGTACAATTAAAATGACGCAGTTTTTGTGGATGTACATTACACTCAGGGGACGGTGACTTCCATAAATGATTGCGGACTTTATATATACATATGTATATACACATACATACACATACACATATATATATAATATAAATATATAAAAATATAATATTTATATATAAAATATAATTTTCATCTAATAAAAGTTAAATAATTTTGGCGTTTTTAGAGCCAATAAAAACGCTATAAATATATATTTATGGGCTATATATTATATTTATATATATATATATATATATATATATATATATATATATATATATATATATATATATATATATATGTATATATATATATATATATATATATATATATATATATATATATATATATATATATATATATATATATATATATATATCATAACTTGCTTGTCTGTCTAACTTTCATTAGTTATTTGCACCGATAAAGGTTCTGATTTGGTTTGATTAAATAAGAAACCTGTGGTTTCATTATTTTGGATAAATTCTTCAAAACAAATTTTTAAGCCCTGCGTAGCAATTCCAAATATATCAATCTTTAATCATTTTAATCATTTTTACAAATGTTATCTGATAAGTATACTGAGTTGTAATCTAACTTGCAGAGGTTGCATATGTTTTGATTAGAAATAAACACATACAAGCAGATATATATATATATATATATATATATATATATATATGTATATATATGTATATATATATACATTATATACACATATATATATATAATGTACGTATTATATATATACATATGAGTAAGCGTTATTGATTAATGATTGTCAGCTTATCAGAAATGCTTAAACAAGGTAGGATTGAAAAATTGTCAAAGTTTTGAGAAATGCTTAAACAAGGTAGAAAAATCTCTCTCTCTCTTGATGTGTTTGCTACTCTCTCTCTCTCTCTCTCTCTCTCTCTCTCTCATTTTAATGGTGACAGCTGTAGTCATCACCAGCTCTCACCGTTAATCTCCCCCATCCCCTTCATTCCCCCCTCCCCATAAGCAAAAACTCCCCTTCACCCCGTGGTCCCCATCCACGGGAACCCCCAACTGTGTCGGAGTCACAACTTGGTGCTCCATAGCGTGTGCATTTAACCATAAGTAACCTGAAATCGATTTTATTACTTCCGACCCTTGAAAAAATCGATGGCTGGCAACGGCGAACGCCACGACCGAGACGAAGGAAGGAAGGACTCCCCCATAGGACCCCCCTCCCCCCACAGGTTTCTCTCTTCCATGGCGAAGGAGGAGGGGGAGGGGGAGGAGGAGGAGGAGGTGGGGGAGGCAGAGGTGGTGATGGTGTGGTACTGGTGGTTAGAGGTGCCAAGAGATATTGGAGGCAGATGCTGATCTCTCTCTCTCTCTCTCTCTCTCTCTCTCTCTTATGGAGGAGGCATTCATGAGTTTGGCTGCAGCAGGTCATTGGATGGGAAAGGAATGTCTTTGGAGGAGTCCAGGCGAGGAAAGTCTACATGTACAGAAAAAGAGAAGTGGAGAAGATGCCTCTCTCTCTCTCTCTCTCTCTCTCTCTCGTTATTGTTTATAGAGTATATAACAAACCACACACACAACACACACATGTTATATATATATATATATGTATATATAACCACACACACAAGAGACACATGCCTGAGAGAGAGAGAGAGAGAGAAAGAGAGAATATATATGTATATATACATATATATAAATATATATATATATATACATACATACATATATATGCATATTTAAGATGAGATTACTAGTCGCATGCACTTTCCACAAGTGATTGACTCAATGCTTAGATGACCAAAGGCACAAAAGGATTTGAAGGGAACGCGGCCATGTATATCACTCCACATCGTAATTAAATCATACGGAAACCTATGTGTAATTTAAATGTGAGAGCATCCAAGAGCAAAGTTACGTCGTACTTCTACTGTCAGTATTTTTATCCATACATATGTAGACTATTTTTGCCAGTTTTATACGTCCTATACGTTCAGTTCTCGTGATAAACTAGTCTTGAAAACGTAAATTCTCGGAACTTTTCAAATCATCTCGGATTCCCCAGTAATCATGAAATACCGAGGTGAGTGACCCACTGTTCCATCTCTCGTCAGTGGTCATCGATGTTGCAATACAGATTGTTTCCAGGTGGCTTCTCTCTCTCTCTCTCTCTCTCTCTCTCTCTCTCTCTCTCTCTCTCTCTCTCTCTCTCGTCGTACATACGACAGCGCATACAATTCACACACTGTGAACCCGGATCTTGCGTATTAAAGCTATAGGTTGGGTAGAAAGAGACAGTAAAATTTACTATTATATATATATATATATATATATATATATATATATATATTATATATATATATATATATATATATATATATATATATATATATATGTACACTATATATATATATATATATATATTATATATAGTGTATATATATGTGTACATACTCATATATATATACGTACATATGTATATATAGCCTATGTATAAACAGATTAGAGTAAACAGAACCCTGCCATAATTCAATAATAATAACAATAATAATAATAATAATAATAATAATAATAATGATAGAATTATCAAATGTACGGGAGTGTCTTATTTGCGCGCAAGTAGTATTTCCTTCTACTATTTTTTAACGAAAGAATACATTTTTTTTCCAGGAAAACAGAGGTTGAAAGAAGAGATAACATAATAAAGACACAAACATTGAGACGATATTCGAAGTCTGGTGAGTACAAGATTTCTTTCCTATAAGACATCGAAAACAAAAAAATACAAATTTTAAATGAACGCAGAGAAATTAGTCCTTAAGAATATCATTATTTACATACATTCACATTTGAATATATATATATATATAATATATATATGTATATGTATCTAGATATATATATAAATGTATATATCATGTATATATATATATGTGTGCATGTATGCGTGCGTGCGTGTGTATGTTTTTCATATGAAACAAACATCAAAGTGCAAAAAGATGAAAATAAAATAAAGCTTTAAAAAGAAGTATTTCGTCAGAAAGTTTCGTCTAGTAAGGGATCTCATTAGGGCGGTTTATTATTGTCTCAAGGCACAAAATTGCAGCCAAGAGCGAGCCGGATGTAAAGAGTAATTACAAGTAAAAAATAAGTTAAAGATGTACAATAAAAAAAAAGTGATAATATTAAACGGTCGACAATTTCATACCTTGCATCCTTAAACTATATTCAGATGTCAATACAAAAGAGAAACAGTAAAAATCAGATTTTAAAAAACGCACACATAACGACTAGAAACGTGGATATATATTTTATAGTACATAAATGAATACATAAAGGCATTAAACACCCTTATATTCGTATTTGAAAGAGGAAAGAATGAGGTTCGCACAATTCGTCATATATAATGTTATATACAGTATATACTGTATATACATATATATGTATATATAGAGACACATATACACACACATACACATATATATGTATATATAGTGTGTATATATATATATATATATATATATATATATATATATATATTATTATATGCATATATATATATATATATATATATATATATATATATATATATATATATATATATATATATATATGGTAAGAACTTTGTGTGAATACACGTCCAGTTAATGATTATGTGACTAACAAGATAGAGATAACTAGATAATATATGTATATATATATGTATATATATACATATATACATATATATATGTATATACATATACATGTATTTTATATATATATATATTAGGCTATAGTGTATATATATATATATATATATATATATATTTTATGTATATACATATAGTATATATTATATATATATAATATATACATATATATATATATATATATATAAATTACACGTTGTGCTCCTCCTTTTCTAGTTTTATAAACATTTTAGCAGTATGATTTTCTTCACATGTCCATTTTCGGAAATGACTGAAGCATGCAGATGCCAGATAAGCTGACCGACAGAATATAAAATTAGACTGGTTTTCAAAACTCTGTTGAACCATCCCTAACAATCTTTTAAGAAAGACTCGGGGAAAACCAGAGTAAATCTATATAACAAAAGAAAATCTTGTTAATAACGCTTGACGTCAAAAACAAAGTTGTTTACAATGAAACTGAGAGGGAAAAACATTCAAATGAAGTGGTATAACAAAAGTGTATATAATAAATATAATATAATTAAGATGATATATTAAGAACCATAGTTACTGAGAAAATTATGCTTGCATTTTCCCCAAAAGTTTCCAGAGAATTATGGGGAAAATGCAAGCATAATTTTCTCAGTAACTACAATTTTTTTTTTTGTATCATATTATATTTAGTATATATATGTATTACAAAGGTGACATTTGGAAACTGAGAAATATCGCAGCCCCTTTTGAACTTGTCTGATTATGTTTAGCAGAGAAACAAAACAGAGCTTGACACTGCCTTGAATGACAAAATAATCATAATAATAATAATAATAATAATAATAATAATAATAATAATAATAATAATAATAATGCAACGATTAGTCGTTGATCTCTTGTTTCAATGAACGTTGGTGAACTGAAAATACAGAAGGCAATAAAGAGTTCCCGAGTCCAAAGCTAGAAGGGAAGTAGAAGCCAAAGCATTTGACTTAAGAGTTCACGAGGTTAAAAGTGCTAGAAAGCCAAACTATTAAGGAAAGAAAAGGCTTCTTGAATAGACAGATCGATTACCACCAGGAGAGTTCCCAACAGAGTCGGAAAATAAATGGAAAGAGATGTTCTGTTCACAACAAATTAGACACAGTTTATTTTAGGCTCACGCTTGGTTGTACCTTTCAAAGGCTGGTCACTGTTTTACTACAAGCTTTAGATAATGGCTAAAGCGAATTTTTCTAAACTACTTGTATGTCCTGTGCAGCCTCTGACGAACGCGGGAGATTTCGTCATAATTTCAAAAGCACATGGAAGGTTTGGGAGAGCATTAGGCGAATGAAATCTTATTTCTGAAAAAGATAAAGCACCCTAGTTACAATTATTAATAATTAACTGTTACCAATTCTACTTCACGCACGTCTAAGAAGGCTATTTTTAGGACAAAATGTTAGTATCATGTGTATACAAGAAAATATATATAAATATAAATATATATATATATATATATATATATATATATATATATATATATATATATATATATATATATATATATATATATATATATATATATATATATATATACATAGCAACTTATTACAACTTACAGTTTATTAACAAAAAACTTCGTTACTCAGATTTTAAATATATAAGATTTTAGTGCACACATCAGTTATTTTCCTTTATCAGAACAATAAAATTTGATCAAAATAGAACAAATTAAAGGAACAACTCCCCACACTGAAATTCAAATGAGAGAGAGAGAGAGTCCAATCAAGGCCAACCAACCCAATAGATAAGGCCGATAGCGAAGGGCGTCTCTCTGAATAGTTTCAGGTCTTTCAGAAGGTGAGGTGTGGGCACTTTTGCCGCTGTATATGGCCCACGCAGTTTATTTCGGTATTATCAGCTCCATCGACCAATGAGAGACAGTGCTTTACCTGAGTAAACACAACTGGCCAATCAGCGAAATCAGCGAAGTCTCTCTAACGAAACTTTCACCCACACTACCTAATGTAATACACGGTTGATTATTTCATGAGTATATTTAGCAGCAAGCTGATGAAATTTCTTTATCTGAGTTTTATTTTTTCTAAAAACGGACCCTATTGAAAGACTGCAACAGTGACGAAAATTTCGAAACAACACGACGGTGCAACGAAAAAGGAAATGTTGTCTGGCGAGCGCTGGCATCCAAAGCGGTGGGCGGAGTCAACGTCACCGCTGAGGCGGTTATCAAGGCCCGGCATCAGTAAACTTTACCATGACTCACAAAAGGGTCGTCGTTTCAACTTTTTCAATATGATGATGAGAGAACAATGCTTAAAGAATGTCGAGATGAGGACTGCGGTAAATGCTGGGCGAGTGTGAGATACAGCACCGCCCCCCTCCAACCCCCCCATTGAAATCACCTGCTTCTCTGCACCTCCATTCCGAAAGGCGGAAAGAGGATGGAATTAGAAGAAAGTAAGAAAATTCGCTTCCTTTGAGAAAATGAAGGATAATGATTGTAATTGCCGTGTTTTGCACGTTATTTTTGGCGATGGCGGCGTCATGCAAAGGGGTAATAGACGTCATCTTCCTTGGAAAATTACGTAATCGTTACAATTGTTCTTTGTGCTTAAGACGGTGACGCTAGAGGCAATTTGCTGGATAATAACACTGGATCCTGATAATAATATGAATATGAAGGCAAATGACAAATATAAATGACAACATGCGATAACAGGAAGATTGAAAAACATCATACAAACATACACATGTATATGCATACAGCTTGATTTTATACACAATTATTTGTAATCATGCATATGCCTTCTTTATTGCTATAAGAAATGACTAAATGAAAAAATAGGTGAGACAATTTAAATTCTGTGATTTTAATTAATTCACTAACTTTAGATAAATCCATAGTTAGCGTTTGATTACCGGTATAATTTTGCTAAAATAAATTTATTATTATTATTATTATTATTATTATTATTTACATGGCTGCAGTCTCAATCATATCAATAGAACGCAAGACAAAGAAAACAAGAAGAAATTATGACTAATGGTAAAAATAAATTTTTCAAAAAGGATGAAACCTTCTATCACATCCTACATCATTTGATACACGTGGCCACTCGAACATTTGGAAAACAAATAAACAAATAAATGAGTCGATAAATAAACAAAAAATTTTAAAAATGTTTTACGATACTAAGACACCAAATGTGAAATAACTTGTTTGATCGCCTTCCCCTTCATATGTGGTGTGCATTTATGTATAATATATTTACATAAATATATATATATATATATATATGTATATATATATGTATTTTTAGGGCATATATATATATATATATATATATATATATATATATATATATATATATATATATATATATATATATATATATATATGTGTGTGTGTGTGTATGTGTGCATTTGTATGTTTGTAGATAAAGATGTAAAAATTAAAAGTGAGAAGGCAGGACTTTTATGTATGATAAAATCGCTACAATAGCCTAACTATTTTATGAACTGTGAGTAATTTGTAAAGTAAGGGTTGCAACATGCCTCTGGTATTACTTGTTATTCTAGGCACAAGAACATTTCTAGATAGCCATATTCGTATGCATCAAAAACATAATAGCTCACGGAATTTCCAGGATTATACATAGCCTGTAGCCAGCAATAGAAAACATAAACATTTACAAATGTATGAAGCATTTGTAGGTATTTTATGTTTATGCTATGTATCTACGCCCATGTGCCTGTATTTGTTTGCTGAAATAGGGTTTGTAAAATTTTATAATAGTGTATATATACTGTATATATATACAGTATATATATAAATATATATATATGTATGTAATAGAAAATATATATATATATAAATATATATATATTTATATATATATATATATATATATATATATATATATATATATATATATATTTGTATATATATATATATATTTTAGAAATTTTAACATAAGCATCTAGTATTGTACGAGGCATTTCTATGACTGTCTATGTCATGTATCATATACATCTATCTTTATATATATATATATATATATATATATATATATATATATATATTATATATATATTCAGCACAAAAAAAACATAAGCATCTAGTATTGTACGAGGCATTTCTATGACTGTCTATGTCATGTATCTATGCCCAACTATCTTTATAGTCTGCAAAATCAGAGTTTGTGAGATATAATATATACTCGTCAATTTGTAGCCGTGATAAAAAAATACTTAAAACCAAATTTGCAAAGACGCTTTCTCGACTATGCAAAAATGTGAAGTTTCCATATTTTGTTTCTCTTCACTCCGTTATCAAAGATTCGGATAAAAACCAAACCATATTCTTGCTTCGTGTGTATGTGTGTGTGTGTGTGTCTGTGTTCGTGCGCGCTCGCGCTTCTCTGCGTACAATGAGTCATGCTCGCTTGTCCAATACAGCACAGGTTAGTGTCACAGAGGGCTGGGGCTTAGTCACGCAAGCAAACTTTATTGGACCAGCGACTGATAATTATCTCTTTCGTGTAAGTCGCGGAACACGTTGAATTATTATGCAAAAACTCGCCTGTAATTTATAGTGTTATGGATAAAAATGACCACCAGACACCGCCATTTCACATGCAAGAGAATACTTGCTATGAAATTACGATTCCGCCTTAAACCTGTAACGACACACTGCGATTTCGCTCTTCCTTTTTTTTTTTTTTCTTAGATTTTTGCTTTCTCCTAAGTTAAAAGAATAATAATAAAAAAAAACATTTAAAAATGCCAACTGCTTTTACACCAGAATTAAAATTCGATTGTGATACTGAATCATTTAAATTTGGACGGTAATCATATCAGTGAGCGCTGCATATTTGTTTCCCTTTTCAATCATATTCTCTAGTTTTATCAAAGAGAAATTTTAAATTTCAACTAAAATGGTCCGTGATTTTAAGGATACAGGGGGCACGGAAAATATTGTGATTCCATGACGAATTGCCTTGTATGTGAATACAATAAAATACATAGTGTAGGAATTAACAAGAAGCATTTATTAGGCTGGCAATGAAAAAGATAGTGAGAATACATACTATTTAATTACCAAAAAGTATTAAATTCACAAATCCAGACCTCCTGGACAAATGCTTTTGATTTTTAACCGTATTTTACTTGCGTCGTCATAAACATCTGATATTCGTTGAAAACTACAACATCACTGACCTAAACATTTCTTGCATAGGAAAGAAACAGGAAAGAAACCTGTATATGTAAGTGTTTTTCATGCTACTTATTGTTGTTCAGTACGAGTACACGTTTATTTAGTAATTAGCCATGAGTGTCAAAACTTGCAAAGCAACGTTCCTGAGATTGGAATGGCCGTATGTAGAAAAAGAGAAAGATATAGGCAGGAATAATAGATAGATAAATAAATAAAAGATACACGTAAAGAGAACAACATAAGGAAATACATGTACTAACTGTTGTTGACAAAAATGAAGTAAAAATTTAGGTGCACCTGGTAAAAACCCATTTTTAGTGAATCACGCCACAAAAACTCCGTACACATACATTTATACATAAGTGTTGATGATTACGCATGTATACATTTTTAATTTACGTGGATGATTTGATAATTACAAGGAAAATGTCAAATTAAAAACCTACAACATATCCTTATCTATGACTAATTTACATACGCTAACATTAGTTACACCATTATCGTTGACATTAGATTACGAGTGTAAGCTACATAATTTGTATTATTTTTATCATTTACACTCTCAAACTCTAAATAAGAACGTAGCACATAAGAGACACACAAGAAGAATTTATGAATAAAGATAAAATGTATGAAAAGGGGTGACGATGACCCAAATTCTCATTCTACCTCATCTTTTACATATTGTTATTACATCATCAGGAAAATAAGTAAATAAATTAATTCATTAAAAAATAAGAACAGTAAATAACTATAGATTTAGCTTCACCTCCAGTTTAAATCAAGCTATATTTTGAGATGCAGCGAAACGAAAGCAAAATTGAGCATTTAGTATAAAATCCTCTCTCTCTCTCTCTCTCTCTCTCTCTCTCTCTCTCTCTCTCTCTCTCTCTCTCTCTCTCTCTCTCTCTCTCTATATATATATATATATATATATATATATATATATATATATATATATATATATATATTACTGTGATTGTCTTTCCCTTGGCTGGAAAGTCACATAATATTCTTCAGAGGTTTGTTTCATTTGATTCCTTCAGTTAAATATGTTTTCATTTTACTAATGAATTTATACACACACATACAGTATATATATATATATATATATATATATATATATATATATATATATATATATATATATATATATATATATATATTATATATCCTATATAAATTCATTAGTAAAATGAAAAAATATTTAACTGAAGGAATCAAATGAAACAAACCTCGGAAGAATATCAAGTCACCTTCCAGCCAAGGTAAAGACAATCACAGTAATATTGAGAAAGGTTATTACGAACTGGACTCTGCTAGAACAACAGTTGGCTTTTAAGAAACTTGTATAGGTAAGGGCCTTCTTCCCAAATATAAGTGAATAAAGTGCTGTATCGACATATTGTCGATTATTTGAATAATTAAGGTCGTATTTAATATAATGACCATACTTAAAGTCATCGACTAATGGTGTGAGTCATTTGAAAATATTAAATTGGGGAAAGTTCTCTACATATATATAGAAGAAGACACCTCCTTTTAGCTTATATTATATTATGATTTTTAATTTACACGTAGACCTATCTATCTTGCTCAAGCAAAGCTAGATTAGATTAAGTGCCCTCAAAACGGGCTTTATTCCTTGTTTCTGGATACCTGTATCTCAAAAATGATTTTCATTTATTATGCTTAAAACACTGAATATTGATTATGCATATTTTTCAAGTGGCAGGATCAAGAAAGTGAAATTTACCAGAGCATAAAAACGAAACGACACACAGTCCGTGCTTATAGGAAAGCGACTAGATGGTTCTGAATAGCCATATTCAAATTCAGTTTTTTTTTTTACCCGAGTGTCCCAGTTCAGCAAAGGCATTTAGGTCTTTTGATAGAGAGAAATTCGAATACTTTCACCAACAGACGCTCATAGATGTTCTCTTTGAAATCACGAGAAAAATCAACATCAGAATGGTGAAGAGTGGTGAAAACTGGAACGCACACTTGCGCTATTTATTTCCTTTACATAAGCATATTTTTTCTAGCGTTGCTGTTACCAGCATTAGACATTAGCGCGAGTTTTAGTTGTTGTTCGTTCGAAACTGATAACAAACAGAAAGTGCCAATGCAAGCTTTATTTCTCACAGATTGATACATAATACAATATTGTATCTACTGTACAGTATATATATATATATATATATATATATATATATATATATATATATATGTATGTGCATGTGTATATTATATATATATATATATATATATATATATATATATATATATATATATATATATATATTAGAATAAATAGCATGCATACAGCCAGTTATAAGATTATTTCGAATAAGGAATGACAGTTTTGACTAACCAGTCCGGAAGCAGAAGATATATACAGTACATACAATATACTGTATATATATTTCCATATATATATATATATATATATATATATATATATATATATATATATATGCATTTAATACATCGTGAAACATACTTAAAAACGCCAAAAAAAAGCCACACTCTAGTTCCAGGATTACAAAAGCAAATTGAAAATAGAGCTAATTGTCTTTACATGCGTTTTGTACGATTACCAAGGAAGTTTCCCTTTCTTTAATATTTTCTTAGCATACTGGAACACTGGGTGTTTTGCTGTTGTGCTGTCTTGAGTGAGGTATTCAGCTACCTCAAGGAAATACGACCATTTAATACCTACGCTGCCGTGTGCTACTGAAGTATTACGCTAATTTTGCAGCAAATTGGTAATGACTTTTTTTTCGTATTGCTGTTAACATATAGATCAATTTTTTCGACTTTTCTCTCCGTTATCGCTTTTTTCCCGTATGCGCCTTCTATTCTGCATTATGATACTCCTTCATAATAATAATAATAATAATAATAATAATAATAATAATAATAATAATAATAATAATACTCGCGCTCATTTGATTTCTGATACTTAACTTCAACTTAATAATAATACTTAATTTCCAGTCCATTTCTCTTTTGGAGACTGGAGAGAGAGAGAGAGAGAGAGAGAGAGAGAGAGAGAGAGAGAGAGAGAGAGAGAGAGAGAGAGCCACATTTGACATGCCTCAGTCTCACAGCTAAAACCAATTTAAAACATATTCAGGTGTAATGATACAGAAAAACATATCTAAATATACTGTACTTATCTAAACCACATGCATACATTACCGCGCAGTTGGTATGAATTCACTATTGCAATTATACATGTAAATATTATATATATATATAATATATATATATATATATATATATATATATATATATATATATATATATACATGTATATGTATTAAATATATATATATATATATATATATATATATATATATATATATATACAGTATGTATATGTATGTATGTACATGTATATAGTTTAAATATGTATATATATATATATATATATATATACACACATATATATATATATATATATATATATATATATATAAACATAAACTAGCATCTAATCAAAGGTATAACTAGCTGGCTTGCAGTAGCCTAATTACGAAGACAATACTACTGGTCATTCTGTAGCAACCGTAATGATTATAAACATCCATGCCACTTAACACATCAGGAATGTTAATTGATCCGTCTACATTCACAATAATATCGCAATGATTTCGTTCATTATCACAATCATTATCGCTAATGATCCCCATGATAATTATTAAGTGCGGTAACAATAATAAAAGTCGCAGAAATAGTAATGTTGGTAATATTTCTAAATGTGGAGATGTCCATAGTACTATCAATTATATTATTGTCCTCAGTTAATGTTAATAAGTCGTCAAGAAATATAAAGAAAGGAACAATAGCTATACGCAGTGAAAAGTTAGCACTTAATGAATGGCAGTCCGTAGTAGTATATCAGAATCTCTTAACTCGACCAAAGATAGGGAACAACATATTTCAAGATTTTAATATTTAGAAACGTAAATATTCATTTACTTGTATATTTTAATTCATAGCCAGGCCATATCTCCCAAGTTTCAGTGCGTCGTCTTTACTTGGTTAAAACAGTAAATAGAGAGAGAGAGAATGGCAGAGAGAGAGAGAGAGAGAGAGAGAGAGAGGCAGCGAAGAGAGAGAGAGAGAGAGAAATGGCAGAAAAGAGAAAGAGCGAGAGAGAGAGAGAGAGAAAGAGAGAGAACTCTGGATGTCAGCACTGTAGGTTGCCATGACAACCATGTGACAATAGGGACGCCGCGAGGGTGTGCAAGTGTGCTGGGGGTGGGGGGGAGAGAGAGAGAGAGAGAGGGGTGAGAGGGAGGAGGTGGGAATGGGTGGGAGAGGTTTGTCGGTGTGCTAGAAGTTTTATCCAAGAGTAGTAAAGTTTCCTGCCCCCGTCCTACCCCGTCCCTGAGGCAGAGGTGGAGTCACAAATCATTGGGTTTGAAGCTGGATGAAATAGAGTAGAGGTGGGGATTGGGGGTTGGTAAGTGGGGTGAGTCTCGAACTTCTAAATAATTGCTTTATGAATCATGTGGTGAACCAACAAGAAGAGAGAGAGAGAGAGAGAGAGAGAGAGAGAGAGAGAGAGAGAGAGAGAGAGATACCATCAAAAAAAAAAAAAAAAAAAAAAAGATACTGTAAAACATCAGGGATACAAGTGAACAATCGAACAGCAAAGAGGAAATATATGAAACGGACATGAACAATTCCACTCAGTTAGTTTTCTAAAACCTTTAGACAAACTCGCATTTCCCATCGACACCTGAGGGGAGCATCGCGGAAAGAGAAATAAACAGCGAAAGAAGCAATGATAATAAAACCAAGGGGGTAAATATGAAATATGCGTATTTTCCGTCTTTCTTGGAGCGTCGCCAAGAGGAAAAGAATATGAGAGAGAGAGAGAGAGAGAGAGAGAGAGAGAGAGAGAGAGAGAGAGAGAGACTGGGACATCCAGACGGGGTGTAAGTTATTTATAAGCCTGGATGTAAATACAGCCCTGATGGGACTCCCCGGGTATGGATGGGTGGCGGTAGCGGACTCCAGGTGTGGGGAGGGGGAGGTTATAGCAGTCGGCGTCTTAACGATCATAGAGCGAGTCACAAATTGGATGGCACACTCACTGGCCCTCACATAATAGGCAACGGACGAGAGTGCCACATGCGAACGGAAACGGGGACGTACGTACGGGCGTGGAAGAAGAAACGTCCGCGAACGAAGGTGAACAGAAGTACGTTAGTTAATAAGGGAGAGAATATCGGTAGGAATAAGCGAGTGCGGCTGTTAACGAATGTTACGCGTGTTTTTCCTTAACATGATTTACATAAAGAAAATAGATATATATATTTTTTTAGGTAAAGAATAGCAAGTGGAAAAAACAAATATGGATAAACATTCATATATATATATATATATATATATATATATATTATATATATATATATATATATATATATATATATATATATATATATATATATATATATGTATGTATATACATATATATATATATGTTACACCACAAATATTGTTTGGTTTAAAATTCATTACTATACCTTGGGAATAAGATACACCTGAAGGAAACAATAAATGATAAGTGCTTCGTCAGCGGCAGGATTCGAACAGATGCCTGCTTTGGAGACAACGATGTTTTTCTATTTGGTGATCCTTATATACAAAAAATTGTTCTAGATGCAAATTATGATAAGGTGAAGCATATATCAGAAACTCATTTAATCTTTCTGATAATCTCTTCCTTCAAAAAATATATATACATGTATTATATTTACATATATGTATATATATGTGTGTATATATATATATATATATATATATATATATATATATATATATATATATATATATATATATATATATATATATATATATATATATATATGTATGTGTATGTGTGTGTGTCTAACAGCTCTCAAAGAGAACAGCGCTGAGAGGATAGATAGTATTTAACCGAAAAGTACCAATCCCTGTAACTGAAACACTACGGATATAGGATAAGTGCTAATGGTTTAAAATATTTTAGGGAGATGTTCCGTTTCAGAAAAAGGCGAGTCTAACTCATGTCCCTTTGTCCTAGTTAAAAAAATATTGCTTCAGTGTCTAAAGGCATACTTTCAGAATTAAATGTGAACAAAGAAGAAACTGGAGATGTTAGGCAACGAGAGTGAGAAAACAATTTGGGCTAAGGAGTAATGTCCTGTGTGGTGTCAACGGAGAATATTCAGATAAGAAGGTAAAAAGCTACAAATCCTGCAGAGAAGATTAAATTCACAGATGTAGCTTCAGAACAATGAAGATCTACGTTCAAATTTTCTCAACACGAGCATATGCCTTCATATGTTTGTTCCACATGAGAATGGATTAAAAATTACAGATGTATCGTGATTGAAAAATTCGGTATGCTAATACCACAACAGATTCGAATCTTGTAGCAAAAATATATTAATTGAACACTTGAAAGTTAACTATAACAAGATATTGCCGTGAACCCACACCAGCAAGAGATTTGAAACGTTGCATATTGCACGGAAAGGTTAGGGCAGTCTTGTATTCAAGCAAGTAACTGAACGAAGGTCTGTTTCTACTGTGGAAAAGAGAAAAATCTTAAATTAAGACAATTATACATCAAAAGTAAATGTTATCAGAAAGCTCTTTCAGAAACCTAGGAATATGTCACGACGTCAACTATAGTCTCCCATCTTTAGGGCGTGAGTACTTTTGGCCTAAGTCAGAGCAAATAACAAAAAAAAAAAAAATCAAGTTTTCAGGTGACAGGCCTATTACTGATTATCGGATAAAAGTAATAAATCATTCTCATAACAAATGAGGATCTAAATATACAGTTTTCAAGATTTTTTTTTCCAAAAACATCTTCGTTATAACGATAACAACAGACGCTGCTGCATAAGCATATATATTTATCATAGAATAATAATTATCATCATCATCATTATTATTTGCCATTATTTATCATTATCATTATTGCACTCTCATAATTATGGAGCCTCATGTAGTGGGATTATTAACCAAATTGTAACTTGAGCATCTACGAAAATATTAATCATTTCATATTGCGAAAATTTTATTGAACGCTAATTAACGGTCGTTTTTAACAAAGGTTTCAGTCAAAACTGACTATTTAGTAATAAGGTTGTATTCATAGAATTGCAAAATACCAAGTTTACTTCTAAGTTTCCAGGTAGATGAATTAAAATTAAATCCCATCTTCCCAATAAAAGTCTTGTTTTTGCGGTGTATTTGAATACGTAAGCGTATGTTTATGCCCGAATATTCAACAAAATATATTATCAATATTCTAAATGTATTGCATATTAAAAATGCTTAACTGTGCTGCGGGAGATATTATCAATATTTTAAATGTATTTTGAAGCGTCACATTAAAAAACTATGGATGACAAACCTGTCAATCATTTCATGGTGTATGAAGGATGGTGTGTGAAGGATGTGACGTATACGTCCTTCATAAATAACAGATCTTTGGTAAGGTCAGGCGCAAAATATTAATAAATAAATAAATTCCAAAGACACGTCACTTCCATTAACGAGTTCACAGGCGCAAATTTTCAAATGAACCTTGATACCACCTCCCTTATCATAAAAAAAAAAAAATATAATAAACAAGTTGACGAAGCAGTCATAAAAATAATAGTACCTGTGGGGTCGGTGACTCGTCATCAAAGTGATTTAAAAAATGTATGACTTTTTAAAGTACAGTTATTATATAAATCTTACAGCTGACCTTTTGAGCAATCGAATTTTACTTTTAAAGTCATAAACTCAAAATTGACGGGGGTGACAGGGAAAAAGATAGCGATCCTCCTATTTGTGTTCTTCCCGACTACAATGGTGTTCCTGTCTGTCTACAGACGTTGATCCCAAATGTGTTGACATAAGCCACGTGTACTTACGTCCTGACACAAGGCCATAAAAAAAAAAGAGTTTCGACCGTGCCTTAAAACTACAGTAATGTTTAGAGAGAGAGAGAGAGAGAGAGAGAGAGAGAGAGAGAGAGATTAATAAATTGACATCACAGCAGTTAAAGTTATCTACAGTATATGTAAGTAAATTTGGTCATGGAAACACACAGAGAGATGTAGATAGATTTATAGATAGATAGATAAATAGCGTCACTTTTATTAGAGAGAGAGAGAGAGAGAGATAGGACTTATTAATAAATTGATATCACAGCAGTCAAAGTCATCTACAGTAAATGTAAATTAATTTGGTCATGGAAACAGATAGTTAGAGAGATATAGATATCGACAGATAGACAGATAGATAGATAGACAGAGGCTAAAAGAATGCTATATATGAATAAGCTCAAGGGCCAAGGAATTTGCTCAGCAAATGGTCCAAAGATCAAATTATTTTTATCTACTGACAAACAAAATCCTTCCAGCATTGCACGTAGACAGGACTCACCATAGAGACGGTGAATCCTGAAAGGTATATCCCCTCTCCCCATCCCCCGTCATTTGCCTGATTTCCACAAGGCGATGGAGTGGTAAGACTGCATGCACCCTGCCCATTTTTTCCTGCATAAGGTATAAGTTTCCGAGCCTAACAGAATGCTGGATTTTGTGGAATAGCCGTATGTGCATCGCCGAATCTAAGGATGCTGTAACCTTAAGTAAGCAGTAATATCATTGCTGCTACTTCACATCATAATAATAATAATAATAATAATAATAATAATAATAATAATAATAAAATAATAATAATAATAATAATAATAATAATAATGATAAAATAAAGCTGTGATACATAATTCAAACGAAATTTATAAATCCCTCGTACCACATCGCTTCCAAAGCTAAATCTGTATGCTAAAGATCGTGTCGGAAATCGTACAACAAAACCACAAGGTAAAAAAAGGCCATGACCACAAATCCCCGGCGTGAGGGCTTCTCATCCCCTGCTCTCGCGCCAAGATCCTCCCTAACCCGCAGGAGATGAAGAGCCCTCGGGGACGCATCAGCGAAAGACCGCGACGGAGGGATTTCCCCCTTCGTGCCCGCGCCTCTGAAGGGCTTTACCAACACGTGTCTTTCCCTCCCTCTTCCGGTCTCGAGAGGCCAATTTGTCCGACCTTGATTCTGCACGCTCTTCCCTCTTCATCTGTTTCGGAGGCGTGATTGAAAAGGTCGCTACGACCCGCTTTCGAAAGGGTTATACGGCACTCGGCTGTGCAGATTTCATTCATAAGATTTTGGTAATGGGACTGAGGTGGATAAACTGCTCATAATTATGATACCTAGTTGCAATTTAAGTTTACTATAGATAAGGATGTATAAATAAGTGACACGTGAATGAAAGGAAGCAAATAAGTGACCAAAATGAGTAACTGATTGCGCTAGCAAACTGGAGTGACATCACATGTTATCGACGCACAGACTAAACGAAGGCAGAAAGTATAATCTAGATTAGCTTTTTCTTATTATATAAGAAAGGAAGAATCATCATCGTTGGTTTTTAATTAAAAATTATGTAAATAATAGTCAACGCCGTGAACACCAACAAAGTACCCAATCTATAACATTAGAAACTGTAATACTGCAGCATTCAACCCATTTTAACTTTTGCCAAAATAAAACAAGTACAAAATTCGCCGAAGTTTCTTCGGCGTAATCGAGTTTCCTGTACAGCCGCTACAGCGTATAATCAAGGCCACCGAAAATAGATCTAACTTTCGGTGGTCTCGGTATAATGCTGTATAAGCCGCGTCTCATGAAACTTTGACCACAGCCTGGTGGTGGCATATCCCATAATGTTGCCAGAAGCACGATTATGGCTAATTTTAACCTTAAATAAAATAAAAACCATTGAGGCTAGAGGGCTGAAATTTGGTATGTTTGATGATTGGAGGATGGGTGATCAACATACCTATTTGCAGCCCTCTAGCCTCAGTAGTTTTTAAGATCTGAGGGCGGATGGACGGGCAAAGCTGGCACAATAGTTTTCTTTTGCAGAAAACTAAAAAATAAGAACTTAATTATCAACCATGATTATGCCGACGTTGATAACAAGGTCAGCAGTTGCACAAGATATAATAATTAACCAATAAAATGATGTGTTCAGCTATCCTTAACCAATAAAAATCACTTCTTTAGTCTTCTAGAACAACGTGTAGCTCTCTCTCTCTCTCTCTCTCTCTCTCTCTCTCTCTCTCTCTCTCTCTCTCTCTGTAATCTATGGGACCCCTGAGTCCGAACGCGTGTTTAGGGAGCGTTTTAAAATTAATTAGCAGAGGTTTAGTGAACAAGTTGTTCGGAACATTAGCCGAGAGTCAACCCCTCCCTCTGTCCGTCCCTCGGGGGAGAGTGTGTGTGTGTGTTTGCTACGTAAGCTCCCGTTCCCTCTGTGAATCACGAACGCATCGGAAAAGCCGGTGATAGATGAATTCGCACAGACCGCCACCGAGTGACGAGTGACGTCACACAAACGCCAGTCCTGGTAGGCAAAGGAGCCTCGAGGCTTTCATGTAGCGCAAATGAATGAACACCGCATTATTAGGAAGGCGCTTTTGAATGAGGCGCGAGTGCCATAAAACAAACACCGCGAATCAATTAAGGCTTTGTTTATCGGATTCCTTGATTAGTTTGTAATTGAAGCGGATACGCCGACTATAAAGGCAAAGACTATGTCATAGCTTATACGATTTTACTCTTGTTTTATTACGTCTTTTTTTTTTTATTATTTCTCTGTCTTCTGCATCTGATGTGGTTGATTTCTTAGGGAAGGTTATGCTGGGAAAGTGAATCGCTGACTGTTTTTTTATTATTGCAGTATTAAGTTTCCACTGTTTAATCATATTTGTTTCGGGTGAAAATTAGCATTTTATCCCGTTTTGACCGTATAAAGTCATTCGGAAAATCCATCAATATGTGATGCGTATAAAATTAAAATTATTATCGTAATGAGTGTCCACTGTTTCTGATCTTGAGCAGAAATGGCTGGAAAAATTTGCAATGCCTTTTAAAACGCTACAGATCTTCAAATAATCTCTCTCTCTCTCTCTCTCTCTCTCTCTCTCTCTCTCTCTCTCTCTCTCTCTCTCTCTCTCTCTCTCTCTCTTTGTACTGTATGTGTGTGCGTATGAATGGCGAATTCCTTAAAACTCTGCTTCCCAGAACTGACAAGGTGTACCTTAAGCCCACCTGTGTTTCGAAAACATGTCCCCCTCAGGCACAGGAGCAGAAGGAAGAGGAGAAAGCAGATTTGTATGAGGAGGAAGGACTGTTATGTCGGCGGCTTTTGGAGCGCCTATATAGAGAGGATTATCCTCCTGTACTCTCACAATGGTATCGAATCATCTGCGTTTGTAGGATCGGCTAATGGTCCCCGGGTGGGACTGATATGTTGTTATTCCATGCGGTTGAGGACACTCCCAAGAAGTTTGGGAGCCACACTTGGGGGAGGTCTGGCCCAGCGGCGTCTTTCGTTGTCAGGCGACATTTCTCTCGGCCTTTTTCTTCTCGCAGAGATCTGGCGCGTTTAATTGTGAAGGCGATGTCGCCGCAGACTATAAATTGTAAATCTATGAAGGATTAATTTTACAACTGGACAGATTTTACCTCGGAGATGGGTGTAACAGGAATAAAAGTAAAATGTCTCATTAAAAATTCTCCCTACACCAGCGAAGTGTGATTTATAATAGTTTTCTGTTTTTCGACACTTCCTGAGTGTGCCTATACCATCCCTTACATGAAATGAATATTCGATGATAATTGTGACCTTCCCCCTACAGGGAAACTTCGCTGATGCATCCATAATCGTGAATGAAACATGTATATGAGAGGGTCACCTGCCTGATAATAATAAAAAACATTATTATTATTATTATTATTATTATTATTATTATTATTATTATTATTATTATTATTATTATTGCTGGTTTATCAAGTTTCCTTGGGACTTATAAAGGTTTTGTCTTGTCGTAATTTTATTTCCTTTAACATTTCGACAAGTGCTCTGTCGGCCATATATACTGATAGCACAGTGTGGAATCTTAAGAGGAAATAAAACTAAGACAAGAGAGAATCTTCATAAGTCCTTAGGAAACTTGATATATAATAATAATAATAATAATAATAATAATAATAATAATAATAATAATAATAATAATAATAATAATAATAATAATAATCTCCAAGGTCATAGTTAATAGTAAGAACGAGAAATTTTTATAGTAATATTGAGAGTAACGGTTTTGACAATATAATAACAAAAACAATGAAGTCTCAAAATTAATAAACACTTATAGAAATTAAAACAATAACTACAAAAACAGAGACAACACCGGCTTTAAAGATAAGGCAGAATTAGCTTCGAACTGAATAATAATAATAATAATAATAATAATAATAATAATAATAATAATAATAATAGCATCAGCATCAAAGCTGATATGAAAGCCATCATCACTGTAAGGTTAAATCGCCGAGAACATCAACCTGGATTGTTGTGTAAACAATTAATCTTCTTCTCCCTCCCGCCGAACAGGATTTTAACCCAAGGATAAGAGGCTCTCTCGGGACACAGGCCTCTTGGATTCACCGCGGCTGTTGAACCTTTAAGGTGTGATTAGGCCTACGGACTTCTTTATCCGGAAAAGATACGTCAAAATAAACCAGAAGATTGTGAAGCTCTCCCGATAATGGTTGTGTATCGCACAAAGAGATCCAGTCGGGTTTCGGTTGTAAGATTGTTTTAACCAAGGTCTTTAGATTAATTGTAACTATGTTGTGAGTGTTGTAGCGAACAATCGGAAGCGAAATTATCATAGTTAATTACGTGAAAGGGAAAGATAGTCTATGGGGGACCTATTTAAATGATAAATAATGCTCGGATGCATATTTAAGGATCCAAAGCAAGTGAAAGCAAGAAATTGGAAATATTATTATTTTTTTTTTCAGGAAATTTGTACAATCTAAGAAAATTATGGCCCAAATTCAAAGATGAAAGTTACGAAATAAAAGTTATGAAACGTCATATGGTATACTAGGCATGTTCCATGCTACAGAATAAAAGAAAATGTAAAAAAAAAAACCATAAATCAAAGAGAGAGAGAGAGAGAGAGAGAGAGAGAGAGAGAGAGAGAGAGAGAGAGAGAGAGAGAGAGAGCAAAGTGTTAACTGTTAACAAGCCAACAAGAACCAGGAAATAAATACTCCACAACAACAATAAAAGCTACAAAAGTCCCACAACAATACCCGTGTAACATTCTCCCTCTCTCTCTCTCTCTCTCTCTCTCTCTCTCTCTCTCTCTCTCTCTCTCTCTCTCTCTCTCTCCTCGCCCTTGTCCACTCGATCTACATTTATTCGGTCTATATCGGTCGTGTGGATGCCGTTAACGTCATTTGTTATCGGAATTATCATTCCTGTCGACATCTTACTCCCCTCTCTCTCTCTCTCTCTCTCTCTCTCTCTCGAGAGAGAGAGAGAGAGAGAGAGAGTTTGCTACGATTTGTGCACGCTTATCTATTGCAAACATTATTTTCATTATAAGGTAAAATGAACACTTCGTTAATTTGAATGATATATATATATATATATATATATATATATATATATATATATATATATATATATATATATACATATATATATATATATATATATATTATATATCTATATACATACATATATATATATATATATATATATATATATATATATATATATATATATATTGCGTGTGTGCAGGCGTGTTTGTTTAGATAGGGGATATGCAAATGTAATGTAATAATTATAATAAAAATACAAATAAAACATAACCACTATGTAAGAAAAATTTACATAAAACAGTGAAATCAAAATCGTAAAAAAAAAAACCGGAAATTTAATCTTAATCTAAAATAATAGAAAAAAAAACATACACAAAAAAAACTTACCCTTATGCGACATGTCGTCCAAAGTGGTGGGGCCCCACGGGTGGTGACCATGCGGGCCATGAGGTCCATGTGGCCCGGCGTCTACTGGGGACAACAT

>*MrIAG*_cDNA

ATGGGATACTGGAATGCCGAGATCAAGTGTGTGTTGTTCTGCTCACTCGTAGCATCGCTTCTCCCTCAACCTTCTTCGAGCTATGAGATCGAATGCCTCTCCGTTGACTTTGACTGCGGCGACATAACGAACACCCTTGCCTCCGTCTGCCTGAGACACAACAACTACATCAACCCAGGACCCACCTACGTTTCCAAAGAGCGACGATCTGCTGACATCTATACCGTTCCTTCTACGAAGTCTCCATCGCTCGCCCACCCGAGAGCTACCCACTTGACCATGGCTGACGAAGAAACTCAGAAGGTATCTAAGGTGGAGGAGGAGATTCAGCACATGACGCTGAGCAGGGAAGAAGCGAACAATATGCTGCATTCGAAGCGTCGCTTCCGGAGGGACAGCGTGAGGAGAAGTCCAAGGGAGGAATGCTGCAACAACGCCTCTTTCAGACGCTGCAACTTCGAGGAAGTCGCCGAATATTGCATCGAGCTGCGTCCCGGCGTTAACACCTGCAGTTCCAGGTAG

>*MrIAG*_DNA (Exons are indicated by yellow background shading)

ATGGGATACTGGAATGCCGAGATCAAGGTGAGTGTTTATAGTGACTGCCATTTTTGGGTTTTCAGTTGGAAAAGCTCTCGAAGCTTTCGTAACCAGCTTTCTTCTTTTATCAAGAGCACCTCCACTGGGTTCAGCTTGGGTGATTTTTCTTGAATATAGCAGTGACGAGTACACAAAACAATGCCCCTTTAGACTGTGGATTTTATTACATAATATTTCGTAGATATCACTGTTAACAATCATCGAAATTGTGGCTTTTTCTTGATATATAAAAAACCGACTGTTCTAAGGTCTTACAGGAAAGCGTGCGTGAATCAAGGTTTCATGTAAATGGTGTAGATAAGCAGTAATTTGTTTTAGCGTTTAGAATTTTCTATGCTAAAAACAAACTTTGTATTTTTATATATAAACAAAACGGCTGGTTGGCAGTAAAAGTGCGCTTTAAACAAGGGTGACTTATTTTTCAATGGATGTTTAAATCAGCAAAAGACAAGGCAACTTAAGAGTAAGTTTCTTTGATAAGAAAATGGGTCATGAATTTAATGTGGAATTGCTGATATAAACCGATGATACTGTGTTAGATGGTGATAGTGGGAGAATCCTCAATCTGTCATAAGCAATCGAAAGCATCTGTGGTAGAACAGAACTGTAAGGTTATTAGTGACATGGAAACCTGTCAGCTCGATCAACGAGAAGTGGAAACACGTGACTGCATTAAGTGTTTGGGACCACAGGTCAAGCGATTGCACGTCCAGATGTAGAGGCTTTTGAAATGAGTTTCTGCGCAGTGTGGCTAAATTCAAAGGGAGTGAAAGCATGAGAAATGGGGAGACAAGAAGACCGGACGTGTTGAGTCACATTTCGACGTGATACGGTGAAGTTATGACACATCCTAGCCACTGGGGGTGGGGGTGGTAACCCAGTTCAATTTAGTGCCGGTCCCAAGCCCGGATAAATAGGGAGGTTGAGTCAAAGAGGCATCCTTCTGTAAAATGTCTGCCGAAAGCAATTTTGAAAAGAGAGCTCATATACAGTATATATATATATATATATATATATATATATATATATATATATATATATATATATATATATATATATATATATATATATATATATATATGTATTTATATTAAGACTATATAAACCAGGGATATATATATTTATATATATATATATATATATATATATATATATATATATATATATATATTATATATATTATATAATATATATATATATAATATATATTATATATATTATATAATCATATATATATATAATATATATATATATAATATATATATATATGAAATCATATATATATATATATATATATATTCATTTTACGGACATTTGATTCGCAGGGAATTCATTTACGGACATTTGATCCCCGAGGGGCTAGTACTAAGCACAGCGAAACGGTGATTCATCTACACTGTTTCGTCGTGTTTAGTACTAGCTCCTTGGGGATCAAATGTCCCTAAGTGCATTTGATCACATTTGATCCCAGAGGGACTGGTACTAAAGACGGCAAAACAACGTAGATGAATTACTGTTTCGCCGTGTCTAGTACTAGCCCCTCGACGATCAAATTTTCCTTAGTGGATTGGTGATTTCACGAATTCCCTGAAAATTTCAGGGATTTCTGTCACATAAATCCCTGGTTATATATAATCTCTGTGTCACATATACCTATGTACATATATATATATATATATATATATATATATATATATATATATATATATATATATATATATATATATATATATATGTCTATATATGTATGTATCTATATATATACACATATGTGTAAACAGACTGAATATATATGCTTTATTTAACATCAATTTTTTAACCTCACTGGGGCAAACCATTACCAAGACTGTCCCTTTGGTTTCAGTGTGTGTTGTTCTGCTCACTCGTAGCATCGCTTCTCCCTCAACCTTCTTCGAGCTATGAGATCGAATGCCTCTCCGTTGACTTTGACTGCGGCGACATAACGAACACCCTTGCCTCCGTCTGCCTGAGACACAACAACTACATCAACCCAGGACCCACCTACGTTTCCAAAGGTATGAGACAGAGGACGATCGTCGTTCTCTCCCCCGGAATGAGTGTCAGAAGGAATCTTTTTGCGTTCGCACTTTCTCTCAAAAACAACAGGATATCAACTATATCAGAGAATAAAGGGTGGTATTCTTTGCTCTGTCAGGAACTGGAACAGATATATAGTTAGTGTTAACCCTCTGACTACATTAAATCATTTTCTAAATCAATTTTTGCGAAGTCAGGCTTATTCGGAATTTTAAAGTTTTTTTGGTTTTGTATTTTTTTTTGTCGACCACAAATTACTGCTTCGGAAAATACAACACATAGATTTTCTTCTTCTTCTTCTCTCTCTCTCTCTCTCTCTCTCTCTCTCTCTCTCTCTCTCTCTCTCTCTCTCTCTCTCTCTCTCACATACTATCACGTTAGCGCGTTTCCTTCTTCATATTTACGTTATCAACGAAAAATTTCCCGAAAATATACTTTAGTCTTTTAATACAACAGAGAATCTCTCTCCTGATACTGAGCAAAGACTGACCTCCTCTACCCATAAGCTCTCCGTTAAATTCTTTCTTATTTTCACAGAGCGACGATCTGCTGACATCTATACCGTTCCTTCTACGAAGTCTCCATCGCTCGCCCACCCGAGAGCTACCCACTTGACCATGGCTGACGAAGAAACTCAGAAGGTATCTAAGGTAAATATATTTATGGTCTTCGTCGCCTCCAGCACCGCGTGGCGCACAGGGCGTCTGTGGAATTTCGCTGCTCTTGTCTCACGTGCTTTGTCTTCCACAAATCTCCACTCATCCCAAACCTCCCTTTTCGCAGTTGTCACCCAATTAGGTTTGGATCTTCCAATCCTTCTGATGCCCACAGGAGCCCGGCTCTCACACTTTCGCCTACTATCCTCCAAAGGATTGGACGAAGTATATTTCCATGATATCTCCGTCTCCCTTTCATCATTACCCGTTCTCCACATGGAATCTCCGCAACCTCCCTTATGGGGTCACTTCTAAAATCTTTCTCACTATCTGACTCCTGATATTCTTCTTAAAGCTTTATTTTCAAATCTATAAAATCTCTTGCATATAGTTTCACTGTCATTTCGTAAGTCATGACCGTATAGCCATGCAGATCTTACCAGACTTATATAGTATAATCTTACTTTTTGCATTAAAATTGCAGTCTGATTAATTGGTTGTCAATCTTATTCAGCCAATCCATTGTTTGATTTGCCGTTTTGAGCACTGAAAAATGGCAAGCAGATATTTTTTTATATCGCCTACAACTTTTCAAATGTACTGCAACTCAACCGCGGTGATATTGCGTTAAAGAATAATTTTAAGGCTAAAATAATTATGGAAATAATTTATTTGAATCGGACATTTAGAACCAGTTAAATGTCTTGTAGTGGATAATGTTAGGCATCGCAGTGCTGACCGAATATAGTATTCATTTCTTCTAACGCAGTCGTTGTTAACCCGACCCCCCCCAACCCAGGTGGAGGAGGAGATTCAGCACATGACGCTGAGCCGGGAAGAAGCGAACAATATGCTGCATTCGAAGCGTCGCTTCCGGAGGGACAGCGTAAGGAGAAGCCCAAGGGAGGAATGCTGCAACAACGCCTCTTTCAGACGGTGCAACTTCGAGGAAGTCGCCGAATATTGCATTGAACTGGTAAGATGAGTCTCCACTGAGCTCACCGTCAATTTTGCAGAGGGCTCTCATTTTCTGACTACCTTCAGATTTACAATACAGAGTTCCCTGTATCTTAGAACTTATATTCTGATTATAGAAGAAATGAAATTTAAACACTCTTTGATCCGTTGCTGGAAACCTTCCTTGTCATTCCTGAGATTTTGTACATACAGCAAATATAAATATACTGTAAGTGTTTATGGAAGATTATATATATATATATATATATATATATATATATATATATATATATATATATATATATATATATATATATAATATATATAATATATATAATATATATATATATATATATATATATATATATGTGTGTGTGTGTGTGTGTGTGTGTGTGTGTGTGTGTGTGTGTGTGTGTGTGTGTGTGTGTGTGTACAGCAATTGTAATAGCCACAATGCCCTCTTAACTTCTCGAATTCTTCGCATTTTGTTTGGATACTCTTGTCACTACCAAGCCTCAAGATCCAAGTGCAAGAAATATAAAGATATTATGATGTCCGGTGGCGGGAAACGAACCCGGGTTCCATAATCATAACTAGTTCACGCTGCCTAAGGCTTTGTAGCGACAAGCGTATCCAAAAGAAGCACGAAGAATTCGTAAAGTTAAGAGGGCATTGTGGCTATTACAATTACATATGTATCTGGTAAAAAGTGACCAGTAGATTATATATATATATATATATATATATATATATATATATATATATATATATATATATATATATATATATATATATATATATATATATATATATATATATATATATATATATATATATATATATATATATATAGAATTTTGAACCTACATAAACTGTCCTTCCTCCACTTCTCAACTTGCTGCATCAATAAAGATAATAAACGTGTCACTCATTCCTAGAACCATAACTGTGTCTCAGCTCCATCTTTACAATGAACAAGCCACTCTCCAACCTACAGCTGTAAGACATGGCTTCACTTCCATCTCGAAAACACTGTATCTCTCCGAGCAACCTTATCTGTACCGTAAGTCCTCGAAACCTTCCACATTTCCTGTCTCCATTCTATCATAAACCCAAAATAGCTTTATAGCTTTATTTTTAACATTCACACACAGCTATTTCCTAGCAAATATATATATATATATATATATATATATATATATATATATATATATATACATACACACAGTATATACATATATATATACATATACACACATATATATAATATATATATATATATATATATATATATATATATATATATATATATATATATATATTTATCAGAAATAGCTGTGAGTGAATATTAAAAATAAAGCTATAAAGCTATTTTTGGTTTATGATAGAATGGAGACAGGAAATGTGGAAGGTTTCGAGGACTTACGGTATACATGATAAGGTTGCTCGGAGAGATACAATGTTTTCGAGATGGAAGTGAAGCCATGTGTCAAACTGTAGGTTGAAGAGTGGCTTGTTCATTGTAAAGATGGAGCTGAAACAAGGTTATGTTATGGTTCTGAGATTGAGTGACATGTTTATTATCTTTATTGATGCAGCGATGCGAGAAGTCGGAGGAAGGACAGTTTATGTAGGTTCAAAGTGTGCCAGGAATGGAGTGTAGGATGGTTGATGTTCCCAGATATTAAAGTGCAAATTGAGGATAATGAAGAGAATCTGTGCAGCCTAGCAAAGGAGTCTGAAATTCTTTGCAAGAGGAATAAGTTGGAAGCAGCTGGATGCGTGCAAAAATTTAGAAGGAAACCTGGAATATCTGTGGAAGCCAAGTTGGGAATGCATGAATGGGTTGTTGAGCCATTTCTCCTCTTTGGAATTGAAGTGTGGTTATACTGAATCAAAATAAGAGGAAAAAAGGAAGTGGAGAAGAATTATTACATAGCACATGTTCACAAAAACCACTGGAAGAATGAGACATGTAAAAAGTGGTTGTAAGAATAGATCAGAATATTTTGAGATGGTTTTGTCATGTGGAAGGAATGAAGAATGGTAAGCTGGTGAAATGATTGTAAAATTCGGAAATGTCAGGAGACAGGAGGGGAGGAAAAGCTAGGCGGTGCTGGACAGATAGCTGAAAGAGGTATCAGACTCAGGCCCGTGAACAGAAAGTGGCCTTTGACTTTTAGTGGCGAGCCACTTCTCATTTAAACAATAGAAAATACATCAAATGAATAAGTAAATAACTGGTAAAAAATAATAGGTACTATGTGGATAATTAGTGATAAATGTGATAAAAGTGATATTTCAAAAAAGCATAGAACATTAATACATTTTGATAACAAAAACATACAAAATAACCGTAAGAATACCAGTTTCTATTGCTTAGAATATTGCAGTAAAATACTAAGGTGGCCCACTTGACTTTTGGTCTAGCGGAATAAGTTAATCTGTAACAGAGATTAAATGATAATTTAGGTACAAGGCACCTAATAATGCAAAACAAAGGCTAAAAAGTAAAGTAATTCATCTAAGAGGCAAAAGTTAATGAGAGCAGTTTTTTGTGCCCAGTTCAACTTAAAATGACTCATCCATAAGCTGCAGCGAATAGCAACAGCAATTCATGATGACTGAACCACCCTCTTTTTACATTGACCATGAGAAGAAAATGGCAGTTCTGCTACAACCCCGACCAAATTCAACACTGAAACGTAGTTTCATCAGAGCACAAAATTTGCCTTTCTATGTCACTTATGAAAGTCTCTTTATGAGTATACTGCACTGCATTCTCTGAAGGAAATCATAGTTGAGTGAAGGAAGAAAGAAAAATGTTCAGTTTTGTTTTAGTGCTACAGTTCCTTATCAGATTGCAGTTTCATTCATCTGCTTTCTTGAAATATCACCAGTTTTCTGATCCATTCTTTGTAAAATGTGATACTCATTGTGCATATGACTTTCTTGCAGCGTCCCGGCGTTAACACCTGCAGTTCCAGGTAG
